# A new slider turtle (Testudines: Emydidae: Deirochelyinae: *Trachemys*) from the late Hemphillian (late Miocene/early Pliocene) of eastern Tennessee and the evolution of the deirochelyines

**DOI:** 10.7717/peerj.4338

**Published:** 2018-02-13

**Authors:** Steven E. Jasinski

**Affiliations:** 1 Department of Earth and Environmental Science, University of Pennsylvania, Philadelphia, PA, USA; 2 Section of Paleontology and Geology, State Museum of Pennsylvania, Harrisburg, PA, USA; 3 Department of Biological Sciences, Don Sundquist Center of Excellence in Paleontology, Johnson City, TN, USA

**Keywords:** *Trachemys*, Fossil turtle, Emydidae, Tennessee, New species, Gray Fossil Site, Deirochelyinae, Taxonomy, Hemphillian, Phylogeny

## Abstract

*Trachemys* (Testudines: Emydidae) represents one of the most well-known turtle genera today. The evolution of *Trachemys*, while being heavily documented with fossil representatives, is not well understood. Numerous fossils from the late Hemphillian Gray Fossil Site (GFS) in northeastern Tennessee help to elucidate its evolution. The fossil *Trachemys* at the GFS represent a new species. The new taxon, *Trachemys haugrudi*, is described, and currently represents the most thoroughly described fossil emydid species known. A phylogenetic analysis, including 31 species, focusing on the subfamily Deirochelyinae is performed that includes the new fossil species, along with numerous other modern and fossil deirochelyine species, representing the first phylogenetic analysis published that includes several fossil deirochelyines. The phylogenetic analysis, utilizing morphological evidence, provides monophyletic clades of all modern deirochelyines, including *Chrysemys*, *Deirochelys*, *Pseudemys*, *Malaclemys*, *Graptemys*, and *Trachemys*. A strict consensus tree finds the recently described fossil species *Graptemys kerneri* to be part of a clade of *Graptemys* + *Malaclemys*. Three fossil taxa, including one previously referred to *Pseudemys* (*Pseudemys caelata*) and two to *Deirochelys* (*Deirochelys carri* and *Deirochelys floridana*) are found to form a clade with modern *Deirochelys reticularia reticularia*, with *D. floridana* sister to the other members of the clade. *Chrysemys* is found to be part of a basal polytomy with *Deirochelys* in relation to other deirochelyine taxa. Two fossil taxa previously referred to *Chrysemys* (*Chrysemys timida* and *Chrysemys williamsi*) form a paraphyly with the modern *Chrysemys picta picta* and *Deirochelys*, and may be referable to distinct genera. Additionally, fossil taxa previously attributed to *Trachemys* (*Trachemys hillii*, *Trachemys idahoensis*, *Trachemys inflata*, and *Trachemys platymarginata*) and *T. haugrudi* are found to form a clade separate from clades of northern and southern *Trachemys* species, potentially suggesting a distinct lineage of *Trachemys* with no modern survivors. Hypotheses of phylogenetic relationships mostly agree between the present study and previous ones, although the inclusion of fossil taxa provides further clues to the evolution of parts of the Deirochelyinae. The inclusion of more fossil taxa and characters may help resolve the placement of some taxa, and further elucidate the evolution of these New World turtles.

## Introduction

The mainly Nearctic family Emydidae is the largest and most diverse family of extant Testudines in the New World (Ernst & Barbour, 1989; Bonin, Devaux & Dupré, 2006; Meylan, 2006; Turtle Taxonomy Working Group, 2010, 2011, 2014, 2017). Emydids are a chiefly western Hemisphere group (except for some species of *Emys*). Emydidae currently consists of two subfamilies (Emydinae and Deirochelyinae) and 10–12 extant genera (Ernst, Altenburg & Barbour, 2000; Fritz & Havaš, 2007; Turtle Taxonomy Working Group, 2017). Deirochelyines can be characterized by having stripes on their necks and limbs (except *Malaclemys*), webbed feet, and are often sexually dimorphic (with females larger than males), while emydines lack stripes, most lack webbed feet (except *Emys* sensu lato), although this is probably secondarily lost in emydines, and sexual dimorphism is less prominent. *Trachemys* is a member of the Deirochelyinae, along with *Chrysemys*, *Deirochelys*, *Graptemys*, *Malaclemys*, and *Pseudemys*. Its generic placement has varied over time (Agassiz, 1857; McDowell, 1964; Rose & Weaver, 1966; Weaver & Rose, 1967; Moll & Legler, 1971; Jackson, 1976; Holman, 1977; Vogt & McCoy, 1980; Ward, 1984; Seidel & Inchaustegui Miranda, 1984; Seidel & Smith, 1986), helping show its variability and similarities with other deirochelyine taxa, although this placement has remained consistent for the last three decades.

Jackson (1988) did the first thorough review of fossil turtles referable to *Trachemys*. He reviewed many of the previously named taxa from other genera (notably *Chrysemys* and *Pseudemys*) and reassigned those he felt were placed in the wrong genera. This was a landmark study in helping to clean up the taxonomy surrounding *Trachemys* and other related deirochelyine taxa. Not long after, Seidel & Jackson (1990) conducted a study exploring the evolutionary relationships of *Trachemys* and its closely related groups. Recently, a thesis by Jasinski (2013a) revisited fossil *Trachemys* and other fossil deirochelyines, and investigated their relationships, with this study a major product of that work. Fossils potentially referable to this genus have been found throughout the United States (Cope, 1868, 1878a, 1878b; Hay, 1908; Gilmore, 1933; Galbreath, 1948; Parmalee et al., 2002; Holman & Parmley, 2005). However, potentially the best fossil evidence comes from Florida and/or from the Pleistocene (Cope, 1868, 1878b; Leidy, 1889; Hay, 1908, 1916; Weaver & Robertson, 1967). Fossils from outside of Florida and the Pleistocene are comparatively quite rare but may help clarify the picture of *Trachemys* and deirochelyine evolution. The purpose of this study is to describe the GFS *Trachemys* species and to establish its phylogenetic relationships. Additionally, the phylogenetic relationships of several fossil emydids are discussed for the first time, allowing for a more thorough look at the evolution of these New World aquatic turtles.

## Geological Setting

**Figure 1:**
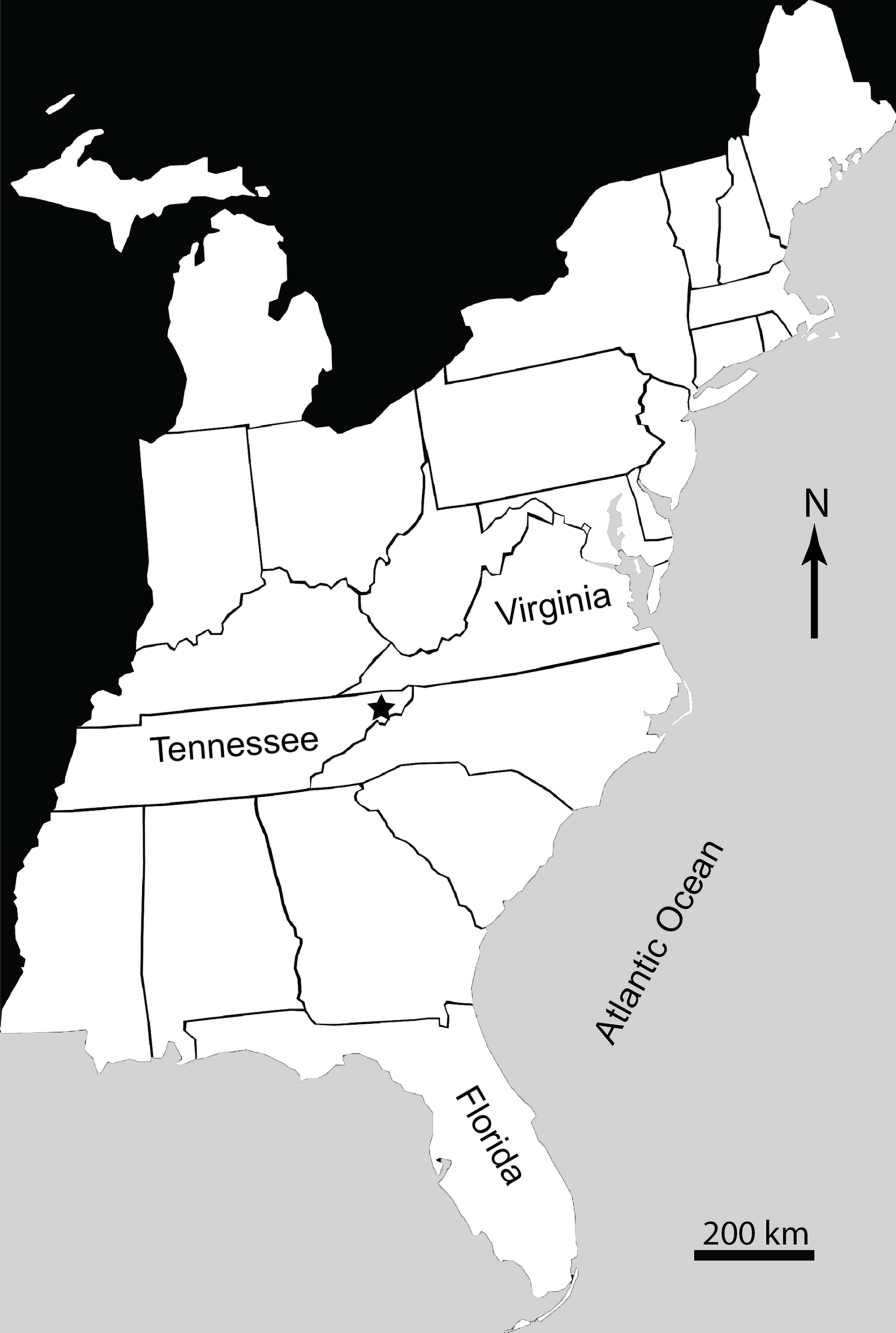
Map of Eastern United States showing location (marked by a star) of Gray Fossil Site in Washington County, East Tennessee, USA.

The Gray Fossil Site (GFS) is located in northeastern Tennessee, USA (Fig. 1). It covers an area of approximately 2.5 ha, and is up to 40 m thick (Wallace & Wang, 2004; Mead et al., 2012). New species of red panda (*Pristinailurus bristoli*) and Eurasian badger (*Arctomeles dimolodontes*) were the first named taxa from the GFS (Wallace & Wang, 2004). Bourque & Schubert (2015) named a new species of kinosternid turtle from the site, *Sternotherus palaeodorus*. More recently, Jasinski & Moscato (2017) named a new genus and species of small colubrine colubrid snake, *Zilantophis schuberti*. A new species of the plant *Sinomenium*, *Sinomenium macrocarpum* (Menispermaceae), has been named (Liu & Jacques, 2010). Gong, Karstai & Liu (2010) and Gong, Liu & Karstai (2011) also mention the presence of three new species of the grape *Vitis* (Vitaceae). Huang et al. (2015) recently described a new species of bladdernut (*Staphylea levisemia*) based on seeds collected from the GFS. Wallace & Wang (2004) discussed the stratigraphic ranges of the rhinocerotid *Teleoceros* and the ursid *Plionarctos* (both found at GFS) and used them to constrain the relative age of the locality to between 7.0 and 4.5 Ma (latest Miocene–earliest Pliocene), during the late Hemphillian North American Land Mammal Age (NALMA). The site is currently believed to be somewhere within Hh3–Hh4 Hemphillian substage (see Tedford et al., 2004 for discussion of substages). This range has not been further refined, but makes the GFS one of a limited number of Miocene–Pliocene vertebrate localities within eastern North America (Farlow et al., 2001; Tedford et al., 2004; Mead et al., 2012). Additionally, it is the only site in the Appalachian region representing the Miocene–Pliocene transition. While it has been suggested the GFS would have only had minor differences in seasonal temperatures and/or precipitation (DeSantis & Wallace, 2008), more recent data have shown that the flora and fauna at the GFS would have been subject to distinct wet–dry seasons, and belies the presence of several warm temperate—subtropical taxa that are currently found farther south such as *Alligator*, *Nyssa*, and *Pterocarya*, which are present at the site (Ochoa-Lozano, Liu & Zavada, 2010).

While it is the mammal fossils that have, thus far, made the site popular among researchers and tourists, and have continued to be relatively well-studied, excavations at the site yield a rich herpetofaunal assemblage as well. Although alligator and turtle fossils are among the most common herpetofaunal taxa recovered at the GFS, a diversity of amphibian remains have also been discovered and have received varying degrees of attention. Though several short abstracts and reports have been published (Schubert, 2006; Schubert & Wallace, 2006; Bentley et al., 2011; Boardman & Schubert, 2011a; Mead & Schubert, 2011; Schubert & Mead, 2011; Jasinski, 2012; Moscato & Jasinski, 2014; Wallace et al., 2014; Darcy, 2015; Schubert, Wallace & Mead, 2015), only a few more detailed studies have been conducted on the remainder of the herpetofauna. These studies include one by Parmalee et al. (2002) on the emydid turtle “*Trachemys* cf. *Trachemys inflate*,” one on the new kinosternid turtle *S. palaeodorus* by Bourque & Schubert (2015), one on the caudates (Caudata) by Boardman & Schubert (2011b), one on the helodermatid lizard *Heloderma* by Mead et al. (2012), and recently one on the colubrid snakes (Colubridae), including a new genus and species (*Z. schuberti*), by Jasinski & Moscato (2017).

Turtles are the most diverse group of reptiles (or amphibians) known from the site; at least seven taxa, from four families, are currently known (Bentley et al., 2011; Jasinski, 2013a; Bourque & Schubert, 2015). Known turtles include the chelydrid *Chelydra*, the recently named kinosternid *S. palaeodorus*, the testudinid *Hesperotestudo*, and the emydids *Terrapene* (or a *Terrapene*-like taxon), *Chrysemys*, *Emydoidea*/*Emys*, and *Trachemys*. A second, smaller testudinid is also present. Much of the turtle material, including the non-*Trachemys* emydid material in particular, is currently under further study. Parmalee et al. (2002) were the first to report on specimens from the GFS and, in fact, reported on turtle specimens they referred to *Trachemys* cf. *Trachemys inflata. T. inflata* is an emydid turtle from around the Mio–Pliocene boundary in Florida (Weaver & Robertson, 1967).

## Systematic Paleontology

Class **Reptilia** Laurenti, 1768
Order **Testudines** Linnaeus, 1758
Suborder **Cryptodira** Cope, 1868
Superfamily **Testudinoidea** *sensu* Gaffney & Meylan, 1988
Family **Emydidae** Bell, 1825
***Trachemys*** Agassiz, 1857
***Trachemys haugrudi*** n. sp.
(Figs. 2A–11, Appendix 4, Figs. S1–S72)

**Figure 2:**
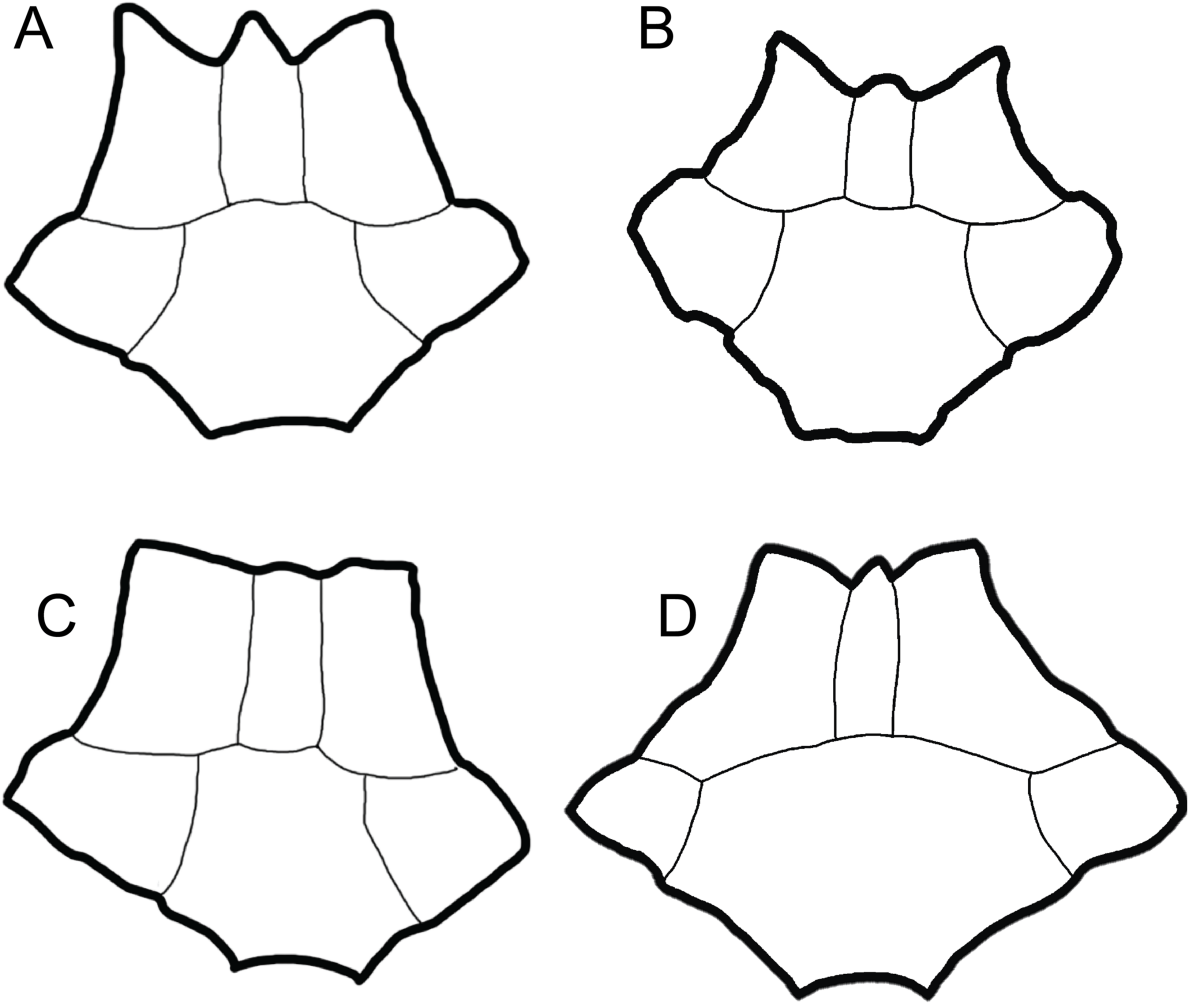
Comparison of dorsal views of nuchals of fossil *Trachemys* species. (A) *Trachemys haugrudi*, ETMNH–8549; (B) *Trachemys inflata*, UF 12460; (C) *Trachemys platymarginata*, UF 10046; (D) *Trachemys idahoensis*, USNM 12059. Nuchals scaled to approximately equal sizes for comparison.

**Figure 3:**
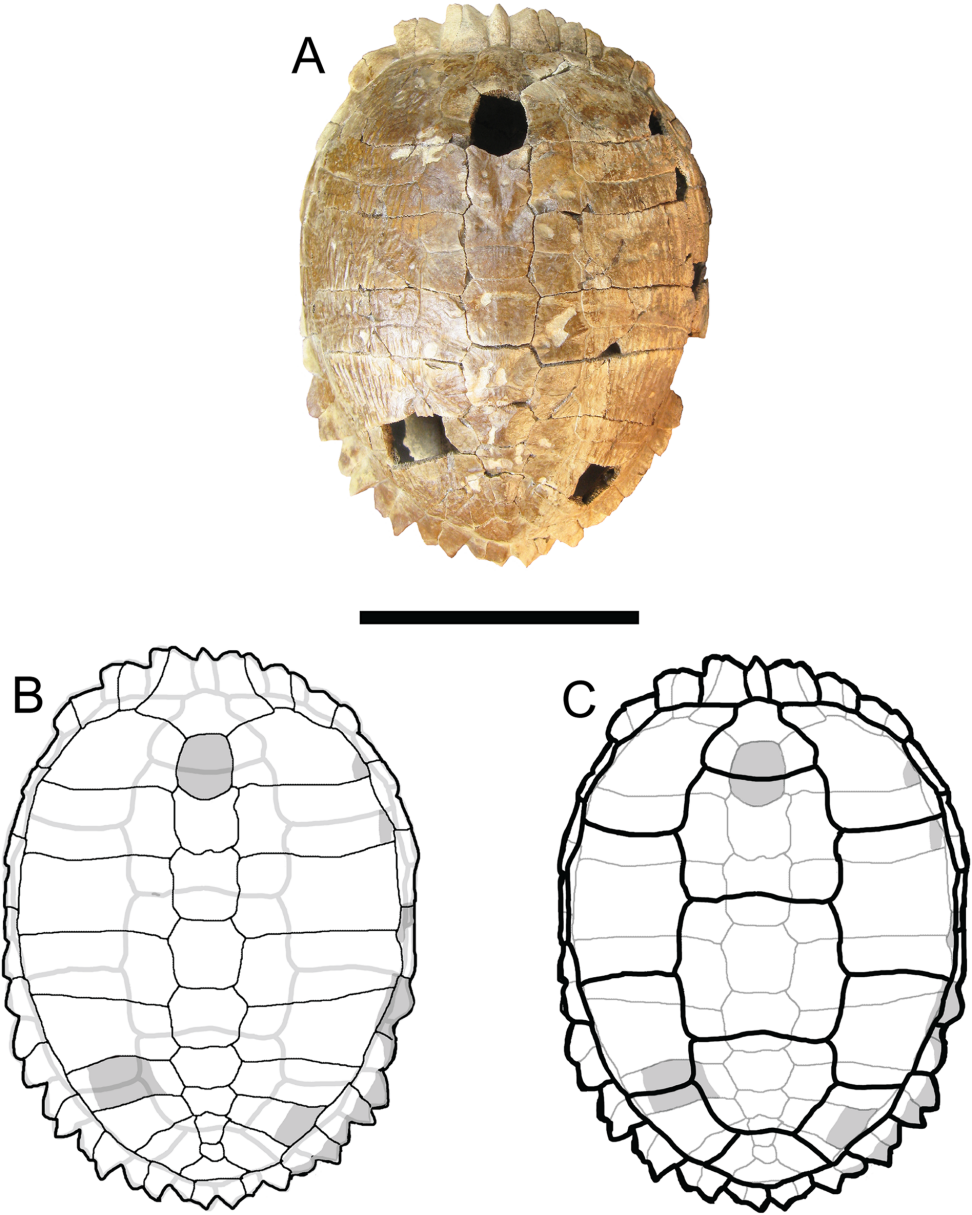
*Trachemys haugrudi*, holotype shell (ETMNH–8549). (A) Dorsal view of carapace; (B) line drawing of carapace in dorsal view, with bones outlined in black and scutes outlined in gray; and (C) with scutes outlined in black and bones outlined in gray. Missing portions are shaded in gray. Scale bar is 10 cm.

**Figure 4:**
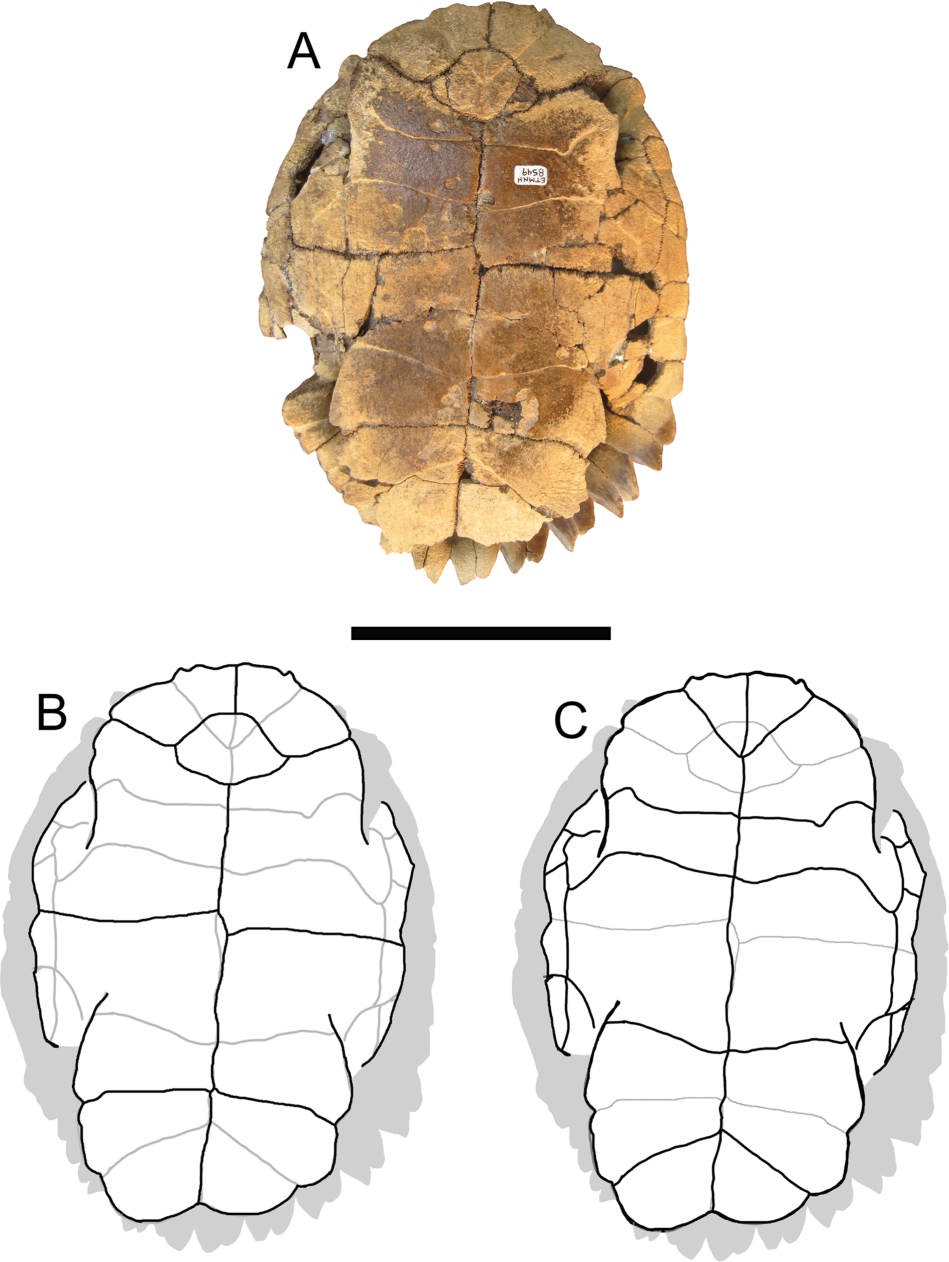
*Trachemys haugrudi*, holotype shell (ETMNH–8549). (A) Ventral view of plastron; (B) line drawing of plastron in dorsal view, with bones outlined in black and scutes outlined in gray; and (C) with scutes outlined in black and bones outlined in gray. Scale bar is 10 cm.

**Figure 5:**
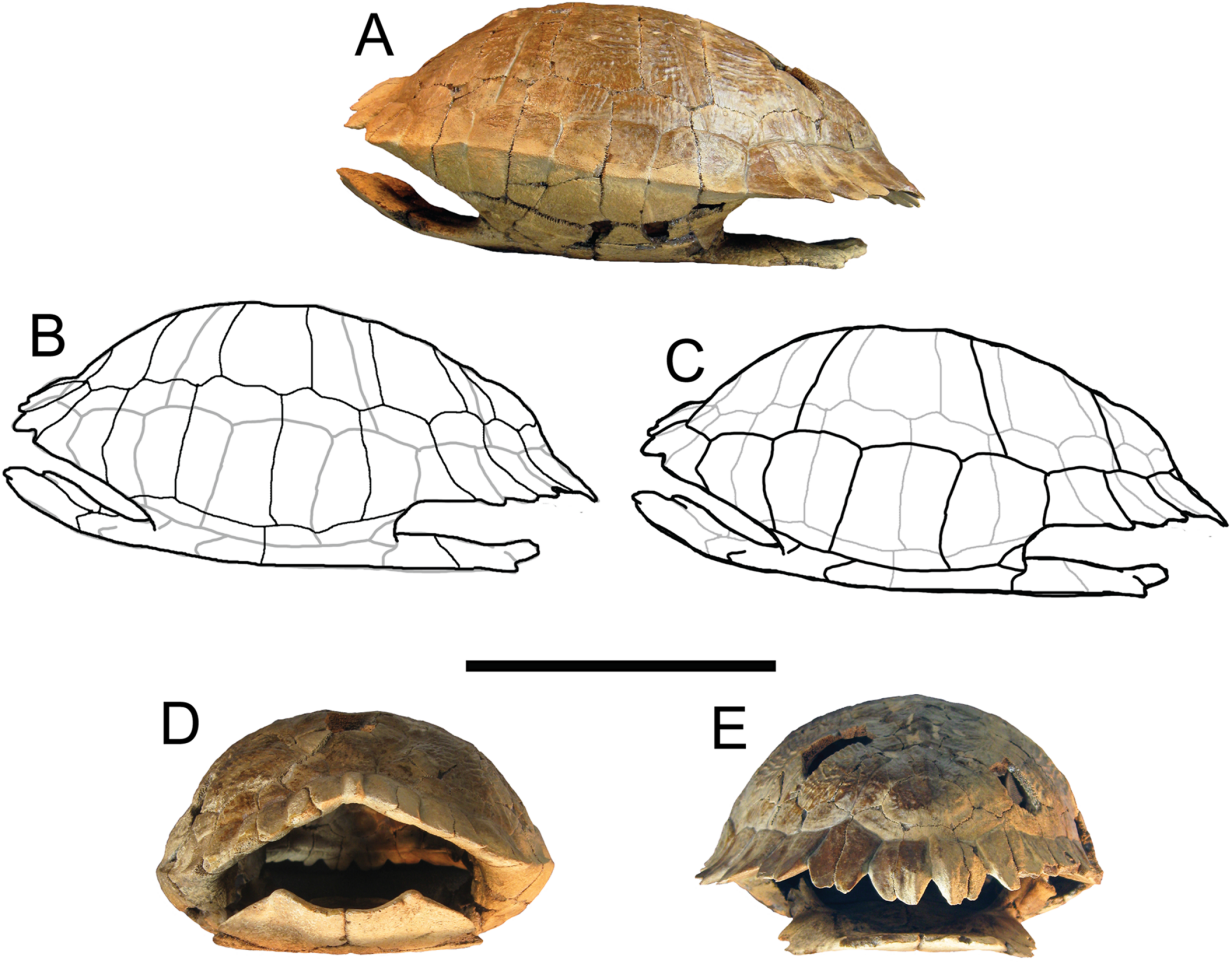
*Trachemys haugrudi*, holotype shell (ETMNH–8549). (A) Shell in left lateral view; (B) line drawing of shell in left lateral view, with bones outlined in black and scutes outlined in gray; (C) with scutes outlined in black and bones outlined in gray; (D) shell in anterior view; and (E) shell in posterior view. Scale bar is 10 cm.

**Figure 6:**
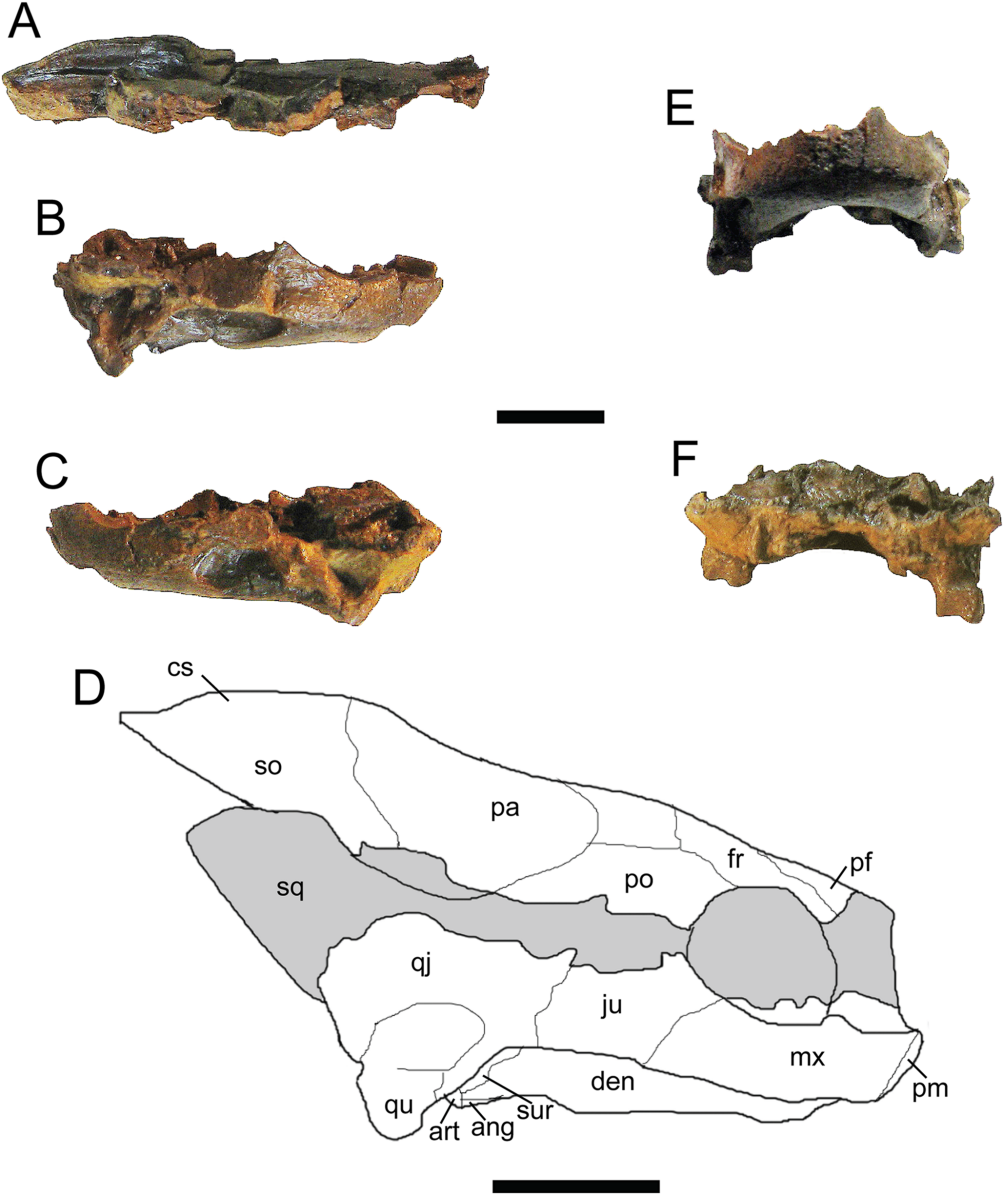
*Trachemys haugrudi*, paratype skull (ETMNH–3562). (A) Dorsal portion of skull in right lateral view; (B) ventral portion of skull in right lateral view; (C) ventral portion of skull in left lateral view; (D) reconstruction of skull in right lateral view; (E) ventral portion of skull in anterior (or rostral) view; (F) ventral portion of skull in posterior (or caudal) view. ang, angular; art, articular; cs, crista supraoccipitalis; den, dentary; fr, frontal; ju, jugal; mx, maxilla; pa, parietal; pf, prefrontal; pm, premaxilla; po, postorbital; qj, quadratojugal; qu, quadrate; so, supraoccipital; sq, squamosal; sur, surangular. Area shaded gray is not preserved and has been reconstructed. Scale bars are 1 cm.

**Figure 7:**
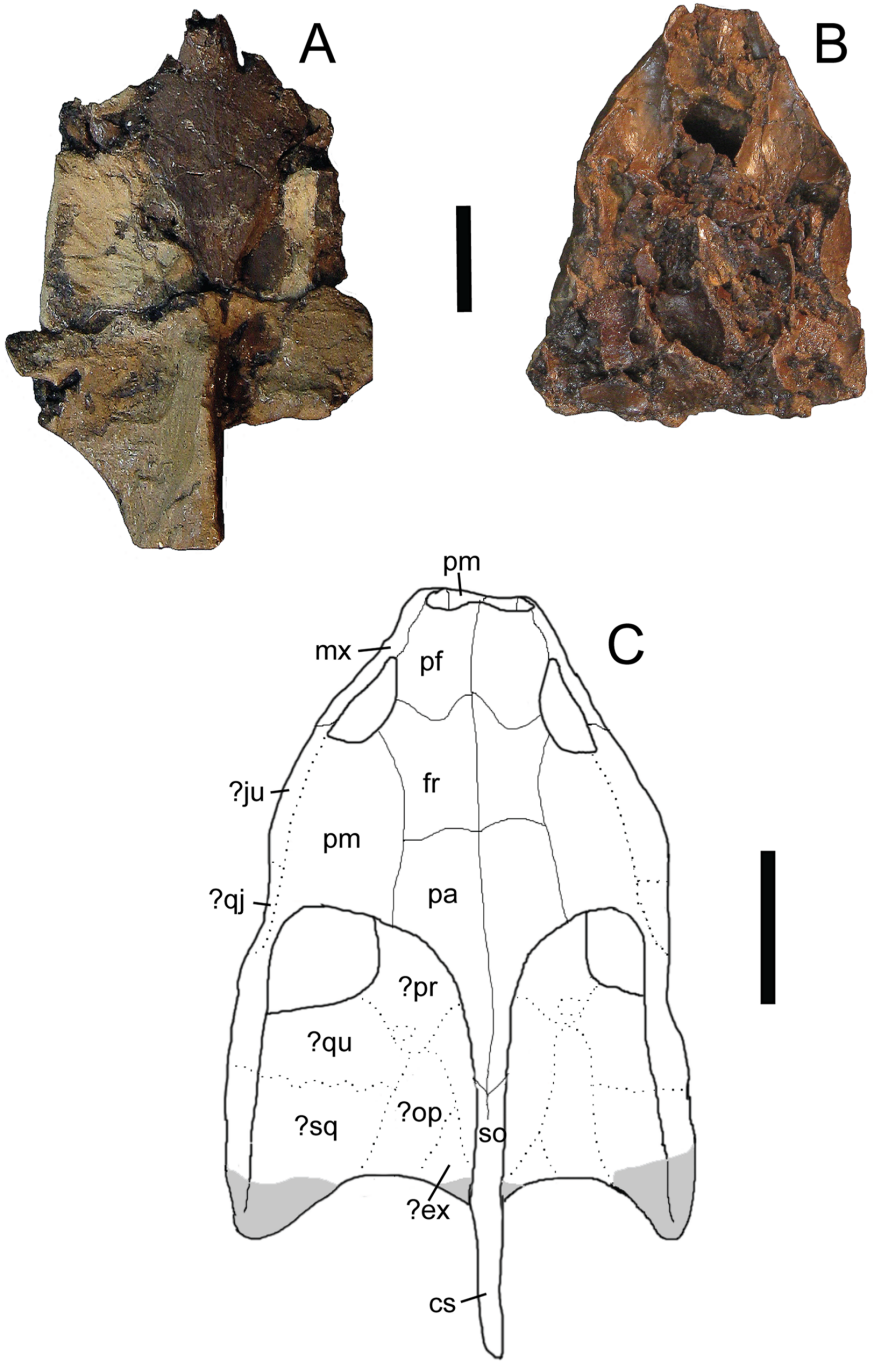
*Trachemys haugrudi*, paratype skull (ETMNH–3562) in dorsal view. (A) Dorsal portion in dorsal view; (B) ventral portion in dorsal view; (C) reconstruction of skull in dorsal view. Area shaded gray is not preserved and has been reconstructed. Dotted lines represent sutures that were not clear in the specimen. Skull has been reconstructed in the slightly deformed state the specimen is in in real life. cs, crista supraoccipitalis; den, dentary; ?ex, ?exoccipital; fr, frontal; ?ju, ?jugal; mx, maxilla; ?op, ?opisthotic; pa, parietal; pf, prefrontal; pm, premaxilla; po, postorbital; ?pr, prootic; ?qj, ?quadratojugal; ?qu, ?quadrate; so, supraoccipital; ?sq, ?squamosal. Scale bars are 1 cm.

**Figure 8:**
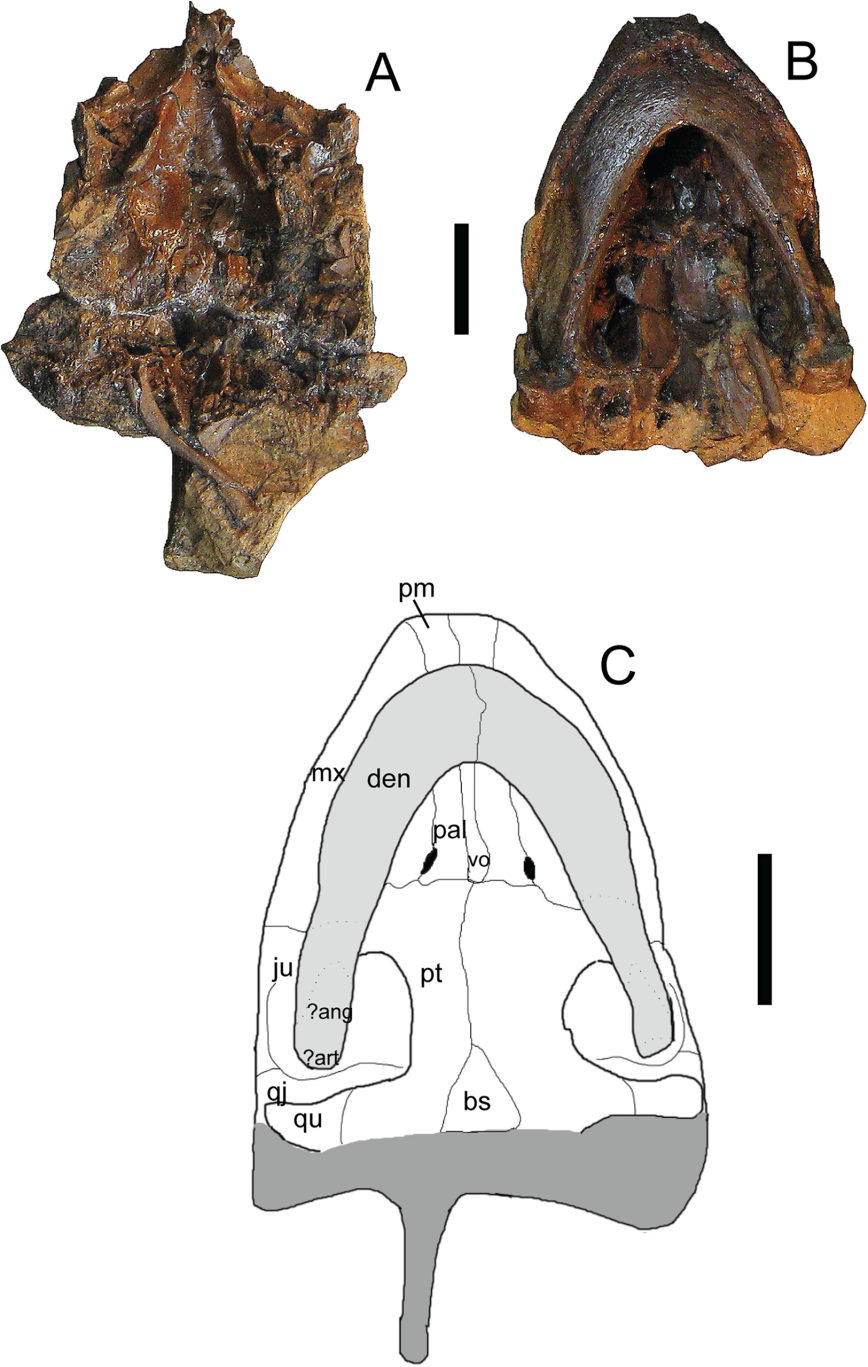
*Trachemys haugrudi*, paratype skull (ETMNH–3562) in ventral view. (A) Dorsal portion in ventral view; (B) ventral portion in ventral; (C) reconstruction of skull in ventral view. Area shaded lighter gray represents the lower jaw that is still connected to the rest of the skull. Area shaded darker gray is not preserved and has been reconstructed. Dotted lines represent sutures that were not clear in the specimen. Skull has been reconstructed in the slightly deformed state the specimen is in in real life. ?ang, ?angular; ?art, ?articular; bs, basispehnoid; den, dentary; ju, jugal; mx, maxilla; pal, palatine; pm, premaxilla; pt, pterygoid; qj, quadratojugal; qu, quadrate; vo, vomer. Scale bars are 1 cm.

**Figure 9:**
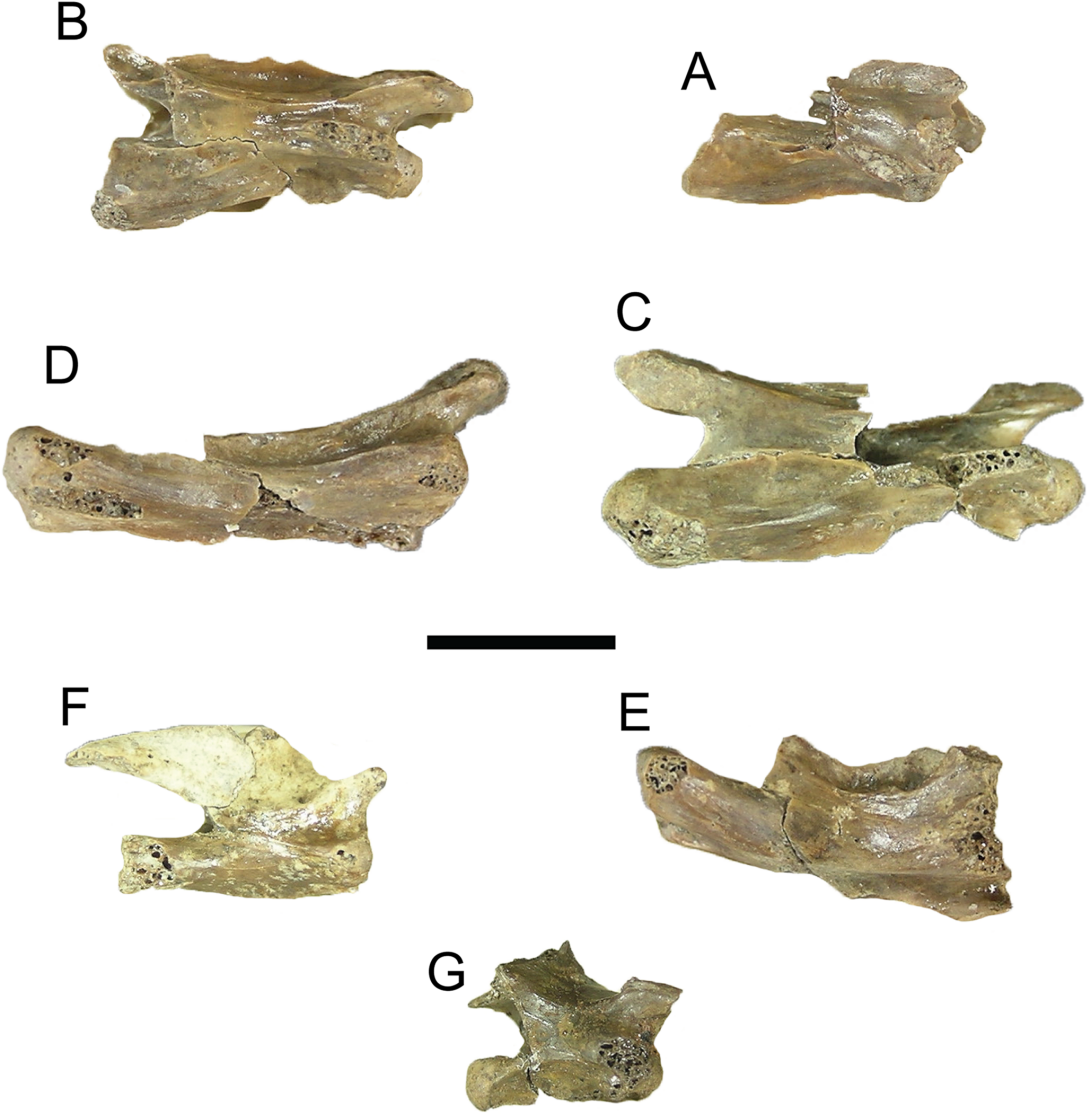
*Trachemys haugrudi*, cervical vertebrae in right lateral view. (A–F) ETMNH–8549 (holotype), (A) cervical vertebra 2 (axis) in right lateral view; (B) cervical vertebra 3; (C) cervical vertebra 4; (D) cervical vertebra 5; (E) cervical vertebra 6; (F) cervical vertebra 7. ETMNH–12832 (paratype), (G) cervical vertebra 8. Scale bar is 1 cm.

**Figure 10:**
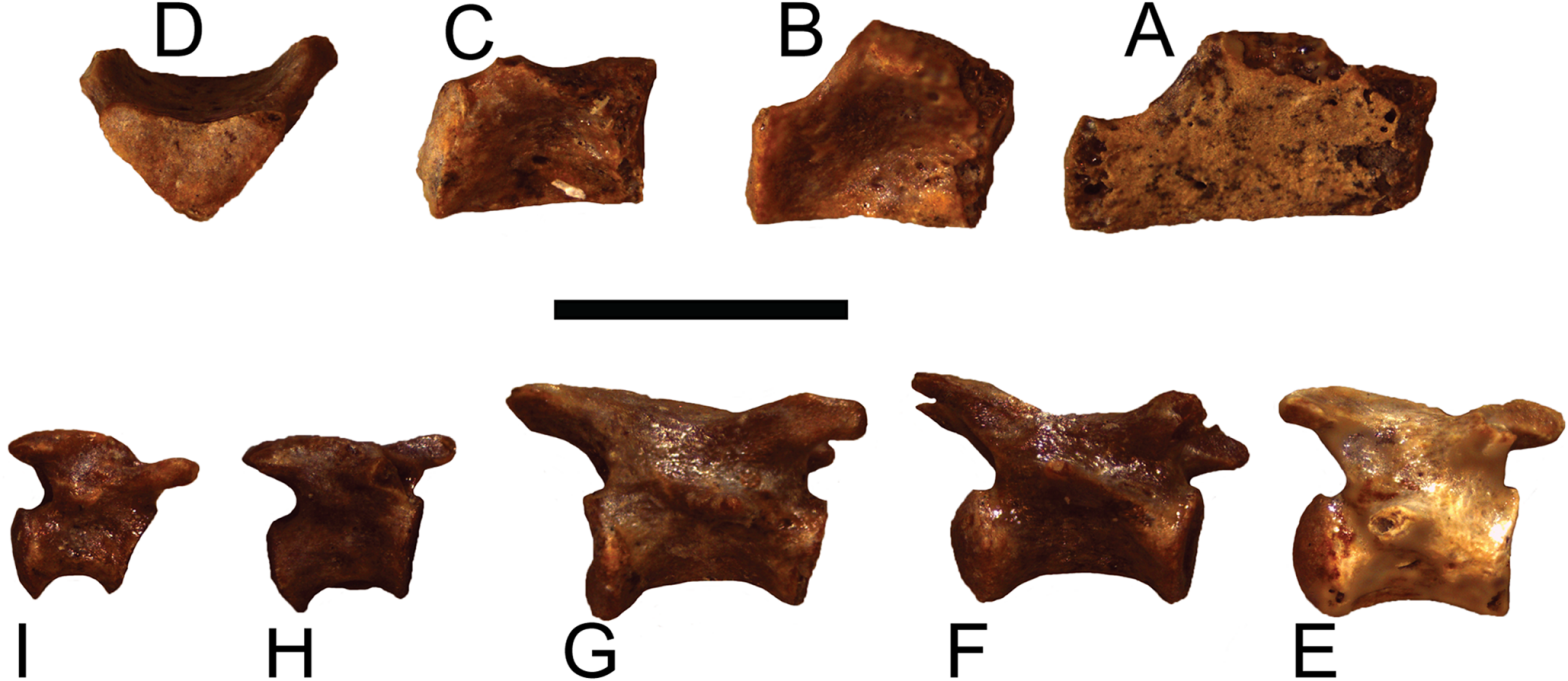
*Trachemys haugrudi*, holotype dorsal and caudal vertebrae (ETMNH–8549) in right lateral view. (A) Anterior dorsal vertebra; (B) median dorsal vertebra; (C) posterior dorsal vertebra; (D) posterior dorsal vertebra (same as in C), in posterior view; (E) proximal caudal vertebra; (F) medioproximal caudal vertebra; (G) median caudal vertebra; (H) mediodistal caudal vertebra; (I) distal caudal vertebra. Scale bar is 5 mm.

**Figure 11:**
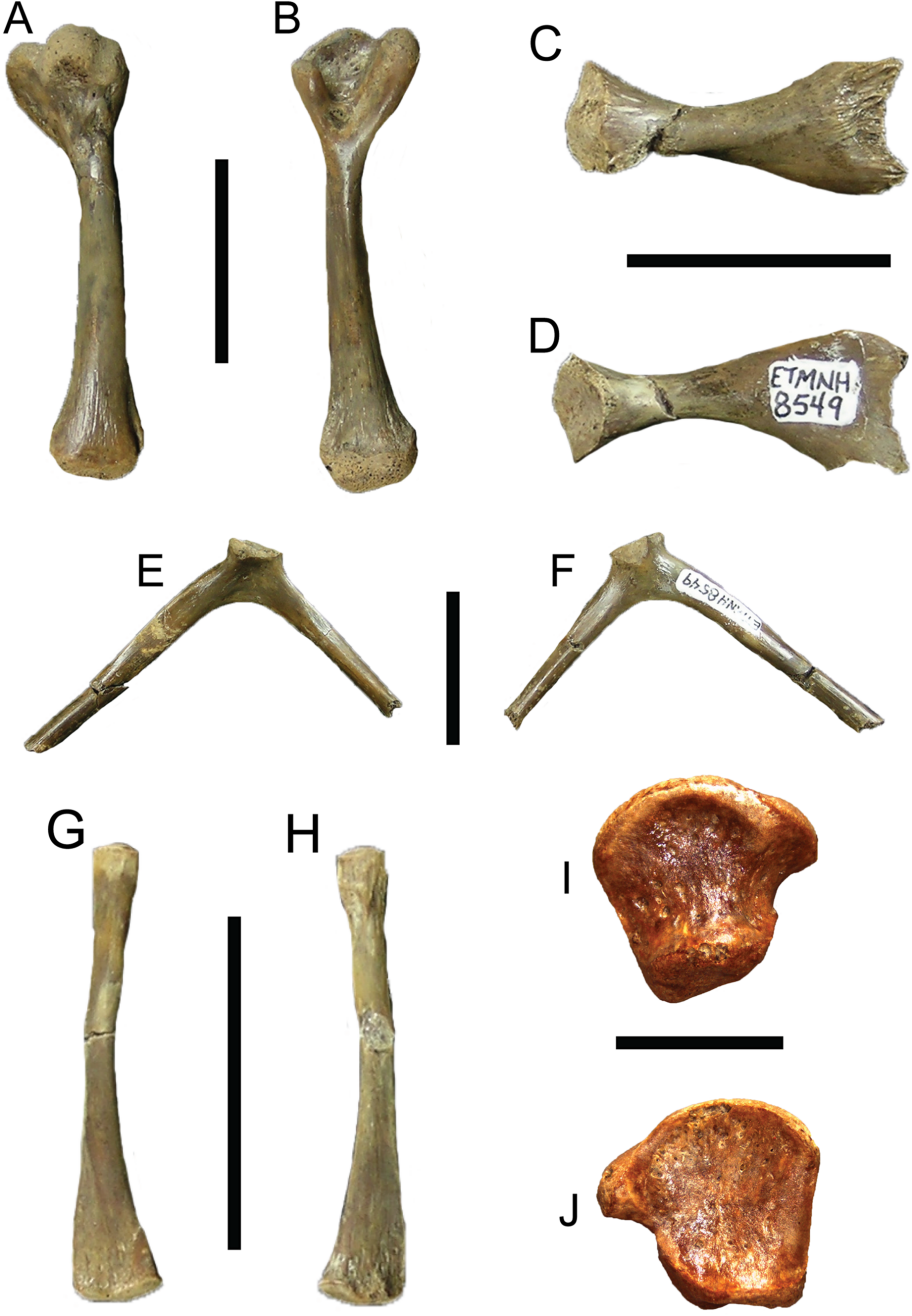
*Trachemys haugrudi*, various appendicular specimens from holotype (ETMNH–8549). Right humerus in (A) dorsal view; (B) ventral view. Left ilium in (C) medial view; (D) lateral view. Left scapula in (E), anterior (or cranial) view; (F) posterior (or caudal) view. Left fibula in (G) dorsal view; (H) ventral view. Left metatarsal V in (I) dorsal (or proximal) view; (J) ventral (or distal) view. Scale bars are 2 cm for (A–H); 5 mm for (I–J).

**Type Specimen:** ETMNH-8549. Nearly complete carapace missing portions of neural I, left costal VI, right peripherals VI and VII and a few other small carapace fragments, nearly complete plastron, cervical vertebrae 2–7, 20 incomplete dorsal vertebrae, 13 complete and nearly complete caudal vertebrae, nearly complete left and right scapulae, complete right humerus, nearly complete left femur, complete left fibula, nearly complete left ilium, nearly complete left pubis, nearly complete left and right ischia, several right metatarsals, right astragalus, right distal tarsals, at least 15 phalanges or phalangeal fragments, and other indeterminate shell and bone fragments (Figs. 3–5, 9A–9F, 10 and 11; Figs. S34–S39, S41–S48, S49A–S49D, S59A, A59B, S60A–S60D, S61A, S61B, S63G, S63H and S64–S70). Note that Figs. S1–S72 are part of the online supplemental material (Appendix 4).

**Paratypes:** ETMNH-3558, nearly complete carapace and plastron, four caudal vertebrae, incomplete right ulna, complete right radius, three right distal carpals, eight right manual phalanges, three right manual unguals, incomplete right ischium, incomplete right and left femora, incomplete right and left tibiae, nearly complete right fibula, four right and three left tarsals, four right and two left metatarsals, four right phalanges, three right pedal unguals, and other indeterminate shell and bone fragments (Figs. S1, S52, S54 and S56–S58); ETMNH-3562, incomplete carapace missing majority of neurals and left costals, nearly complete plastron missing parts of the left hyoplastron, hypoplastron and xiphiplastron, incomplete skull and lower jaws, 17 caudal vertebrae, dorsal vertebrae fragments, incomplete right and left humeri, nearly complete right radius, complete right ulna, two right metacarpals, two right distal carpals, four right manual phalanges, one right manual ungual, incomplete right and left femora, incomplete right and left tibiae, two left and four right metatarsals, two left and two right distal tarsals, six left and seven right pedal phalanges, three left and two right pedal unguals (Figs. 6–8; Figs. S2, S51C, S51D, S53C, S53D and S55); ETMNH-4686, majority of left half of shell, with at least portions of all but the nuchal and costals VII and VIII on the left side, plus portions of the right and left dentaries, incomplete left scapula, incomplete left humerus, incomplete left radius, incomplete left ulna, incomplete left femur, and other indeterminate shell and bone fragments (Fig. S3); ETMNH-6935, nearly complete carapace and plastron (Fig. S4); ETMNH-7630, nearly complete juvenile plastron missing only the left epiplastron (Fig. S5); ETMNH-7690, incomplete carapace and nearly complete plastron, skull fragment (posterior portion of dorsal half of skull), plus other indeterminate shell and bone fragments (Fig. S31); ETMNH-11642, nearly complete carapace and plastron, missing portions of the posterior, incomplete left and right dentaries, three incomplete dorsal vertebrae, several distal phalanges, and other indeterminate shell and bone fragments (Figs. S12 and S72); ETMNH-11643, nearly complete carapace and plastron, nearly complete left dentary, nearly complete left innominate (left ilium, left ischium, left pubis), nearly complete right ilium, nearly complete right ischium, nearly complete left fibula, nearly complete right fibula, nearly complete right tibia, multiple vertebrae fragments, and other indeterminate shell and bone fragments (Figs. S13, S51A and S51B); ETMNH-12456, nearly complete carapace and plastron, missing portions of the left posterior and middle posterior sections, several vertebrae fragments, incomplete left scapula and coracoid, incomplete right coracoid, nearly complete left and right humeri, complete right ulna, nearly complete left and right radii, incomplete left and right femora, complete right fibula, multiple phalanges, two unguals, and other indeterminate shell and bone fragments (Fig. S16); ETMNH-12457, nearly complete carapace and plastron, nearly complete left maxilla, incomplete left and right dentaries, incomplete left scapula and coracoid, incomplete left humerus, and other indeterminate shell and bone fragments (Figs. S17, S32 and S33A–S33E), ETMNH-12726, nearly complete carapace missing portions of the left costals and portions of the right posterior peripherals, nearly complete plastron, complete caudal vertebra, complete left tibia, incomplete left femur, multiple pedal phalanges, several pedal unguals, and other indeterminate shell and bone fragments (Fig. S18); ETMNH-12753, juvenile incomplete carapace and complete plastron, nearly complete right and left dentaries, four caudal vertebrae, nearly complete left scapula, incomplete right and left humeri, incomplete right and left ilia, incomplete right femur and nearly complete left femur, incomplete left fibula, multiple metatarsals, three distal carpals/tarsals, multiple phalanges, multiple unguals (Figs. S20 and S33F–S33I); ETMNH-12832, nearly complete carapace and plastron, nearly complete cervical vertebrae 4–8, multiple incomplete dorsal vertebrae, nearly complete left and right scapulae, nearly complete left coracoid, nearly complete left and right humeri, complete right and nearly complete left ilia, nearly complete left and right ischia, nearly complete left and right pubes, nearly complete left femur, nearly complete right fibula, complete right tibia, multiple metatarsals, multiple pedal phalanges, multiple pedal unguals, and other indeterminate shell and bone fragments (Fig. 9G; Figs. S23, S40, S49E, S49F, S59E, S59F, S60E, S60F, S61C, S61D and S63A–S63F); ETMNH-12833, nearly complete carapace and plastron, incomplete left ilium, and other indeterminate shell and bone fragments (Fig. S23); ETMNH-13443, incomplete carapace and plastron, consisting of the middle and right posterior of the shell, incomplete right scapula, nearly complete right humerus, nearly complete right ischium, nearly complete right pubis, nearly complete right femur, multiple dorsal vertebrae fragments, and other indeterminate shell and bone fragments; ETMNH-14049, incomplete carapace with incomplete nuchal, right peripherals I–III, left costals I–V, right costals I–VIII, neurals I–VIII, incomplete plastron with right and left epiplastra, entoplastron, right and left hyoplastra, incomplete right and left hypoplastra and a right xiphiplastron fragment, nearly complete cervical vertebrae 3–5, multiple incomplete to nearly complete dorsal vertebrae, plus other indeterminate shell and bone fragments; ETMNH-14362, complete carapace and plastron, multiple dorsal vertebrae fragments, and other indeterminate bone fragments (Fig. S30). All paratypes come from the type locality and are listed numerically by specimen number.

**Referred specimens:** ETMNH-8, right peripheral XI; ETMNH-102, incomplete peripheral with three indeterminate carapace fragments; ETMNH-296, incomplete right hyoplastron, right peripheral ?V, incomplete right peripheral ?VI, right posterior peripheral and other indeterminate shell fragments; ETMNH-339, anterior portion of right epiplastron; ETMNH-721, complete nuchal and left peripheral I; ETMNH-3557, incomplete pygal and left xiphiplastron; ETMNH-3560, incomplete carapace with portions posterior to neural IV, incomplete plastron with complete left xiphiplastron, incomplete right xiphiplastron, incomplete left hypoplastron, incomplete right and left femora, incomplete left ulna, plus other indeterminate shell and bone fragments; ETMNH-3568, incomplete entoplastron and right hyoplastron; ETMNH-6936, incomplete carapace and plastron, incomplete dorsal vertebra, incomplete right humerus, incomplete right ulna, incomplete pedal ungual, and other indeterminate shell and bone fragments; ETMNH-7628, three incomplete peripherals; ETMNH-7629, anterior of plastron, including incomplete right and left epiplastra and entoplastron; ETMNH-7634, incomplete left peripheral X and indeterminate carapace fragment; ETMNH-7654, right hypoplastron; ETMNH-7658, incomplete pygal, two peripherals, and incomplete right costal V; ETMNH-7659, complete neural and incomplete right hyoplastron; ETMNH-7664, left posterior peripheral and costal fragment; ETMNH-7665, right posterior peripheral and weathered suprapygal; ETMNH-7674, right posterior peripheral; ETMNH-7688, incomplete carapace; ETMNH-7689, incomplete posterior portion of carapace and plastron; ETMNH-8311, incomplete carapace and plastron, plus numerous indeterminate shell fragments (Fig. S6); ETMNH-8550, posterior portions of carapace plus right and left xiphiplastral (Fig. S7); ETMNH-8735, complete nuchal; ETMNH-10390, incomplete carapace and nearly complete plastron, nearly complete right humerus, and other indeterminate shell and bone fragments (Figs. S8 and S9); ETMNH-10391, incomplete carapace and nearly complete plastron, incomplete cervical vertebra ?V, and other indeterminate shell and bone fragments (Fig. S10); ETMNH-10547, anterior portions of the carapace and plastron, incomplete right dentary, and other indeterminate bone fragments (Figs. S11 and S71); ETMNH-12253, complete right xiphiplastron and complete right peripheral ?IX; ETMNH-12265, nearly complete carapace and plastron, right dentary fragment, multiple dorsal vertebrae fragments, complete right ilium, nearly complete left ilium, nearly complete right ischium, incomplete left ischium, nearly complete right pubis, incomplete left pubis, nearly complete left femur, incomplete right femur, and indeterminate shell and bone fragments (Figs. S14 and S62); ETMNH-12400, right peripheral III; ETMNH-12407, left peripheral ?IX; ETMNH-12409, left peripheral VIII; ETMNH-12413, left peripheral VII; ETMNH-12416, right peripherals VI and VII; ETMNH-12417, nearly complete right costal VI; ETMNH-12418, complete right costal V; ETMNH-12419, incomplete left peripheral IX; ETMNH-12420, incomplete right costal III; ETMNH-12423, right peripheral X; ETMNH-12424, incomplete plastron, including complete left and right epiplastra, complete entoplastron, complete right and incomplete left hyoplastra, and right peripherals III and IV (Fig. S15); ETMNH-12425, right peripheral XI; ETMNH-12428, incomplete right peripheral III, incomplete right epiplastron, and other indeterminate shell fragments; ETMNH-12522, right hypoplastron and posterior peripheral; ETMNH-12524, complete right peripheral X; ETMNH-12754, complete right peripheral VIII; ETMNH-12577, incomplete right and left dentaries; ETMNH-12727, incomplete carapace and nearly complete plastron, plus numerous indeterminate shell fragments (Fig. S19); ETMNH-12759, nearly complete right peripheral X; ETMNH-12772, incomplete carapace and nearly complete plastron, and other indeterminate shell fragments (Fig. S21); ETMNH-12789, suprapygal plus two other shell fragments; ETMNH-12794, complete right costal IX plus indeterminate carapace fragments; ETMNH-12834, nearly complete carapace and plastron, incomplete right and left dentaries, one caudal vertebra, and other indeterminate shell and bone fragments (Figs. S24); ETMNH-12848, nearly complete left costal V plus indeterminate shell fragments; ETMNH-12979, nearly complete carapace and plastron, missing portions of the right posterior of the shell, incomplete right coracoid, and other indeterminate shell and bone fragments (Fig. S25); ETMNH-12988, nearly complete carapace, missing portions of the right side, nearly complete plastron, and other indeterminate shell and bone fragments (Fig. S26); ETMNH-13032, posterior portions of the carapace and plastron, and other indeterminate shell fragments (Fig. S27); ETMNH-13033, fragments of a juvenile carapace and plastron, including anterior portions of the plastron, plus indeterminate shell fragments (Fig. S28); ETMNH-13036, portions of the anterior and middle of the carapace, incomplete right and left epiplastra, incomplete left ischium, nearly complete left pubis, incomplete left femur, two metatarsals, two pedal phalanges, and other indeterminate shell and bone fragments (Fig. S29). All referred specimens come from the type locality.

**Type locality:** Gray Fossil Site, Washington County, Tennessee, USA (Fig. 1).

**Type horizon and age:** Late Miocene–early Pliocene (late Hemphillian LMA, 7.0–4.5 Ma). This range means the fossil locality, and *T. haugrudi*, lies somewhere within Hh3–Hh4 (see Tedford et al., 2004 for discussion of substages).

**Etymology:** The specific name honors Shawn Haugrud, preparator at the GFS who spent countless hours working on many of the specimens cited within and who helped piece this ancient turtle back together.

**Diagnosis:** *Trachemys haugrudi* is placed in the Emydidae due to the absence of musk ducts, inframarginals reduced to two, normal hexagonal neurals 2–8 (also occurs in a few batagurids (=geoemydids); e.g., *Mauremys*), and costal-inguinal buttress confined to C5. It is placed in the Deirochelyinae due to distinct lack of pectoral overlap of the entoplastron and lack of a hingable plastral lobe with ligamentous bridge connection (also present in some emydines). Diagnosed as a member of the genus *Trachemys* by features discussed by Seidel & Jackson (1990), including the combination of: a posteriorly strongly serrated oval carapace; a vertebral keel; low longitudinal ridges (mainly on pleurals (and costals)); alternating seams of the vertebral and pleural scutes that do not align; broad plastron this is notched posteriorly, lacks plastral hinge but possesses well-developed bridge; relatively shallow cranium rostral to the basisphenoid; relatively narrow zygomatic arch and narial openings; orbits located anterolaterally; broad triturating surface of the maxilla, with a medial ridge lacking tuberculate denticles; lacks cusps or serrations on outer cutting edges of jaws; narrow triturating surface of lower jaws; and ventral (lower) surface of the dentary is rounded when viewed anteriorly. *T. haugrudi* is distinct from all other fossil emydids by possession of the following suite of characters: (1) a notch between nuchal and peripherals I, at the anterior-most point of anterolateral surfaces of nuchal; (2) cervical region of nuchal may project anterior to the anterior-most points of the marginals I region, with significant notching between cervical and marginal I; (3) anteromedial border of epiplastra form “right angle” with medial border of epiplastron where it is sutured with opposite epiplastron; (4) more pronounced rugosity in *T. haugrudi*; (5) entoplastron projects anteriorly at humeral–gular sulcus (anteroposteriorly) in *T. inflata*, while in *T. haugrudi* it remains angled; (6) more pronounced notching of carapace in *T. haugrudi* (e.g., >34% notching (*T. haugrudi*) versus 20–33% notching on posterior peripherals (*T. inflata*)). The combination of the first four characters differentiate *T. haugrudi* from all other species of *Trachemys*, while characters 5 and 6 further differentiate it from the closely related *T. inflata*. Additionally, *T. haugrudi* is further differentiated from *T. inflata* by; (1) *T. haugrudi* is less inflated (still more inflated than other *Trachemys*) than *T. inflata* (suggested by Parmalee et al., 2002); (2) anteroposterior length of nuchal under marginal I is the same length or longer than distance from posterior margin to vertebral I–pleural I–marginal I point in *T. haugrudi* versus less than to subequal to in *T. inflata*; and (3) anterior margin of pygal slightly concave posteriorly in *T. inflata*, while flat in *T. haugrudi*. Further comparisons follow in Remarks section.

**Remarks:** *Trachemys haugrudi* was once identified as *Trachemys* cf. *T. inflata* by Parmalee et al. (2002), and still appears closely related to the latter taxon. In comparing *T. haugrudi* to *T. inflata* (holotype = UF 12460), a fossil deirochelyine from the late Miocene–early Pliocene of Florida (Weaver & Robertson, 1967), namely the late Hemphillian NALMA (Webb, 1969), there are several similarities and differences (several are already listed in the diagnosis of *T. haugrudi*). The nuchal of *T. haugrudi* has the strongly indented anterior edge that is common in fossil *Trachemys*, as does *T. inflata* (Fig. 2). The portion of the nuchal under vertebral I is commonly widest at its anterior edge, and this is the case in *T. inflata* as well. In *T. haugrudi*, however, the anterior and posterior edges are approximately equal. The posteromedially directed and concavely curved edges of the region under vertebral I are also more strongly curved than in *T. inflata*, which tends to exhibit a gentle curve. Anteroposteriorly, the length of the nuchal under marginal I is the same length or longer than the length from the posterior margin to the vertebral I–pleural I–marginal I point in *T. haugrudi* versus less than, to perhaps equal to, in *T. inflata*.

*Chrysemys limnodytes* (holotype = KUVP 7676), a fossil deirochelyine from the early Pliocene of Nebraska (Galbreath, 1948), does not possess any serrations or indentations on the anterior or posterior edges of the carapace, while *T. haugrudi* does. *C. limnodytes* also does not possess indentations at the sulcus between the femoral and anal scutes, nor at the posteromedial edge of the plastron between the anals (anal notch), while *T. haugrudi* does. As in *T. inflata*, *C. limnodytes* has the anterior edge wider than the posterior edge of the vertebral I region of the nuchal, which differs from *T. haugrudi*.

*Trachemys bisornata* (holotype = ANSP 9843 + 9844), a fossil deirochelyine from the Pleistocene of Texas (Cope, 1878b), has the entire dorsal surface of the nuchal rugose (strongly textured), while only the pleural I regions of *T. haugrudi* are rugose. The dorsal surface under the vertebrals has various ridges and rugosities, while this surface is smooth in *T. haugrudi*. Recovered elements of *T. bisornata*, including the nuchal and peripherals, reveal it to be more gracile (thinner with less thickness to the elements and features of the element) than *T. haugrudi* and *T. inflata*.

The holotype of *Trachemys delicata* (holotype = USNM 8823), collected from the Pleistocene of Florida, was believed to be a right costal IV (Hay, 1916). It has longitudinal rugosity toward its lateral region. If this specimen is a fourth costal, then the sulcus between the two pleurals runs almost directly down the middle of the costal. On the fourth costal of *T. haugrudi*, however, this sulcus bends toward the lateroposterior corner. However, USNM 8823 is believed to be a right costal II instead, allowing the orientation of the sulcus to agree. If the specimen is a second costal, then *T. delicata* still differs from *T. haugrudi* in its medial edge, which is broader and more strongly angled in the former.

The holotype of *Trachemys euglypha*, collected from the Pleistocene of Florida (Leidy, 1889) belongs to the Wagner Free Institute (WFI), however its number and exact disposition are currently unknown. However, the type was illustrated, and Hay (1908) described other referred material as well. It differs from *T. haugrudi* in the former having a smaller overlap of the pleurals I on the nuchal, with its anterior border straight, rather than concave posteriorly as in *T. haugrudi*. Also different is the nuchal of *T. euglypha,* which is rugose under vertebral I. Additionally, distinct between the two taxa, the region under vertebral I projects anteriorly between the first marginals in *T. euglypha*.

*Trachemys hillii* (holotype = AMNH 2425) is known from the latest Miocene–earliest Pliocene of Kansas (Cope, 1878a), dating to the late Hemphillian NALMA (Jackson, 1988). While the anterior portion of the shell is absent in *T. hillii*, the posterior is not serrated, in contrast to *T. haugrudi*. The humerals are anteroposteriorly shorter in *T. hillii*. The shell of *T. hillii* is more sub-rectangular, with both bridges nearly parallel or sub-parallel, while the shell of *T. haugrudi* is more rounded. The shell of *T. hillii* is more flattened dorsoventrally as well. Additionally, the anterior edge of the pygal of *T. hillii* is also concave posteriorly, while that in *T. haugrudi* is straight (see character 163 (Pygal C) in Appendix 2).

*Trachemys idahoensis* (holotype = USNM 12059), originally named *Pseudemys idahoensis* and collected from the Pliocene Glenns Ferry Formation (Blancan NALMA) in Idaho (Gilmore, 1933), has various differences with *T. haugrudi*. Anteriorly, the nuchal of *T. idahoensis* is not notched (or serrated) and the cervical does not project beyond the anterolateral edges (marginal I region) of the nuchal (Fig. 2). The anterior edge of the carapace is not notched or serrated either, although there are approximately two inconspicuous indentations between the first three marginals. A more elongate and less rounded (oval) shell is present in *T. idahoensis*, compared with *T. haugrudi*, and is almost twice the length (approximately 35.0 cm for *T. idahoensis* versus 20.5 cm for *T. haugrudi*). Vertebral I is also distinct in *T. hillii*, with a constriction immediately posterior to the anterior edge, giving it a “sub-hourglass” shape, and the anterior edge flaring laterally. Posteriorly, the posterior border of the posterior suprapygal in *T. idahoensis* has two sharp angles, giving it three distinct segments, while that in *T. haugrudi* is well rounded with only a single distinct segment. *T. idahoensis* possesses only a single set of serrations (between the marginals) on the posterior edge of the carapace, while *T. haugrudi* contains two sets (between the marginals and between the peripherals). The pygal is distinct, being strongly “pinched-off” from the surrounding peripherals, elongate, and having a narrow, small indent at its posterior edge (presumably lying between the marginals XII). Anteriorly, the anterior lobe of the plastron extends beyond the anterior edge of the carapace in *T. haugrudi*, but not in *T. idahoensis*, where it is approximately even with it. Both the anterior and posterior lobes of the plastron in *T. haugrudi* are inflated (laterally), while this is not the case in *T. idahoensis*. The humeral–pectoral sulcus contacts the entoplastron in *T. idahoensis*, but not in *T. haugrudi*. A medial indent, present at the femoral–anal sulcus, is small and inconspicuous in the former taxon, but pronounced in the latter. Finally, the anal notch of the plastron is quite pronounced in *T. idahoensis*, but less conspicuous in *T. haugrudi. T. idahoensis* was identified as a potential stem *Graptemys* by Joyce et al. (2013), however the present study finds it to be a member of *Trachemys* for characters discussed below.

Since *Trachemys petrolei* (holotype = AMNH 3933), from the Pleistocene of Texas (Leidy, 1868), is only known from fragmentary material, little comparison is possible. However, *T. petrolei* appears to lack significant anterolateral gular projections, while *T. haugrudi* does exhibit them. The cervical region of the nuchal has sub-parallel lateral sides in *T. petrolei*. The marginals I region in the younger taxon (*T. petrolei*) is broad anteriorly, with some fine serrations on its edge, distinct from *T. haugrudi*. Also noted is that vertebral I in *T. petrolei* is broader anteriorly than posteriorly. A single peripheral is preserved and, while it does exhibit a small indent between the corresponding marginals, it seems that there would be no indent present between the peripherals.

*Trachemys platymarginata* (holotype = UF 10046) was collected in Pliocene strata of Florida (Weaver & Robertson, 1967), and determined to be Blancan in age (Robertson, 1976), and shows several differences from *T. haugrudi*. Distinct from *T. haugrudi*, the nuchal is not serrated, and the cervical region does not extend beyond the anterior border of the marginal I region (Fig. 2). The shell is not strongly inflated (=thickness of elements) either, which seems to be most prominent in *T. haugrudi* and *T. inflata*. Anteriorly, the anterior edge of the carapace does not exhibit serrations (indentations) between the marginals or peripherals. *T. platymarginata* would have been a longer turtle than *T. haugrudi*, and its shell is more elongate and oval. This also means that the neurals are more elongate and thinner. This is most evident on neural III, with a thin posterior border. The lateral edges of the carapace are sub-parallel, compared to the rounder carapace of *T. haugrudi*. There is little evidence of a median keel in *T. platymarginata*, which would have been relatively inconspicuous. As evidenced by the posterolateral peripherals, however, the posterior edge of the carapace would have had double serrations (between the peripherals and between the marginals). The carapace was not highly domed, although this could be sexually dimorphic. The gular region of the epiplastra projects anteriorly, although there are no anterolateral projections of the gular region.

*Trachemys sculpta* (holotype = USNM 16681), known from the Pleistocene of Florida (Hay, 1908), is distinct from *T. haugrudi* in several ways. *T. sculpta* has only small indentations on either side of the cervical region of the nuchal. The nuchal is also covered in various ridges and sculpturing, while only the pleurals I region of the nuchal of *T. haugrudi* is. The vertebral I region of the nuchal is wider anteriorly than posteriorly. Anteriorly, the edge of the carapace lacks serrations or indentations in *T. sculpta*. Dorsally, the entire carapacial surface of *T. sculpta* is textured, while in numerous others, including *T. haugrudi*, the region under the vertebrals is smooth, or relatively smooth. Posteriorly, the posterior edge of the shell has double serrations (between the marginals and between the peripherals), although these are all relatively inconspicuous. Posteriorly, the posterior border of the suprapygal has two sharp angles and three segments, similar to *T. idahoensis*. The anterior and posterior plastral lobes are not laterally inflated. Finally, the abdominal–femoral sulcus is more flattened and less curved than in *T. haugrudi*.

*Trachemys trulla* (holotype = AMNH 3934), known from the Pleistocene of Texas (Hay, 1908), is distinct from *T. haugrudi* in a few characteristics. Anteriorly, the anterior projection of the gular region of the epiplastra is prominent, although it does lack serrations and anterolateral projections of the gular region. The anterior and posterior plastral lobes are only slightly laterally inflated, not nearly to the degree present in *T. haugrudi*. The anal notch is also less conspicuous in *T. trulla*.

*Trachemys scripta*, an extant species, was originally named by Thunberg (1792) in a study by Schoepff (1792). Rhodin & Carr (2009) discussed the holotype specimen (UUZM Types 7455 = holotype of *T. scripta scripta*), and some of its complicated history. *T. scripta* is distinct from *T. haugrudi*, although the former is highly variable and can often make taxonomic comparisons difficult. In general, there are only small indentations on either side of the cervical region, and the anterior edges of the marginal I region of the nuchal is flattened rather than pointed, as in *T. haugrudi*. Dorsally, the only completely smooth region of the nuchal is the cervical region in *T. scripta*. The region of the nuchal under vertebral I is also wider anteriorly than posteriorly. Under the vertebrals, the carapacial surface is ridged and textured. While the anterior edge of the carapace is not serrated, there can be a small indent between the first and second marginals. Posteriorly, the posterior rim of the carapace does exhibit double serrations (between the peripherals and between the marginals). The anterior edge of the pygal is concave posteriorly, while it is straight in *T. haugrudi*. The plastron is covered in texturing and low ridges in *T. scripta*. Anteriorly, the anterolateral gular projections on the epiplastra are small and more inconspicuous than in *T. haugrudi*. The anterior and posterior plastral lobes are slightly inflated, albeit less so than in *T. haugrudi*. The abdominal–femoral sulcus is relatively straight as well. The medial indent at the lateral edges of the femoral–anal sulcus is less prominent, as is the anal notch at the posterior edge of the xiphiplastra between the anals.

Holman & Parmley (2005) referred several shell fragments to *Trachemys* cf. *T. inflata* from the late Hemphillian of Nebraska. This referral was based on the original identification of the GFS *Trachemys* material to *Trachemys* cf. *T. inflata* by Parmalee et al. (2002). Regarding the referred xiphiplastron (MSUVP 831) (Holman & Parmley, 2005, fig. 2), there is no, or at least no significant, anal notch present. *T. haugrudi* possesses a significant anal notch. The nuchal, with its thickness and general morphology, does suggest *Trachemys,* however. The Hemphillian Nebraska material discussed by Holman & Parmley (2005) are conservatively identified as *Trachemys* sp. until further material is recovered.

## Description

### Methods

Terminology used throughout this study follows several well-known previous studies, including Thomson (1932), Zangerl (1969), Gaffney (1972), Ernst & Barbour (1989), and Joyce (2007), among others. Measurements are all maximum lengths and/or widths unless otherwise stated. Orientations are in proper anatomical position unless otherwise stated as well. Note that Figs. S1–S72 are part of the supplemental material (Appendix 4).

The electronic version of this article in portable document format (PDF) will represent a published work according to the International Commission on Zoological Nomenclature (ICZN), and hence the new names contained in the electronic version are effectively published under that Code from the electronic edition alone. This published work and the nomenclatural acts it contains have been registered in ZooBank, the online registration system for the ICZN. The ZooBank LSIDs (Life Science Identifiers) can be resolved and the associated information viewed through any standard web browser by appending the LSID to the prefix http://zoobank.org/. The LSID for this publication is: urn:lsid:zoobank.org:pub:79D23F9D-EB5C-4CB4-8C2E-F00B63E00624. The online version of this work is archived and available from the following digital repositories: PeerJ, PubMed Central and CLOCKSS.

### Shell

(Figs. 2A–5; Figs. S1–S30)

*Trachemys haugrudi* from the GFS is represented by several well preserved and mostly three-dimensional shells (including both carapaces and plastra). While the specimens are often somewhat crushed or “deformed” while in situ, careful preparation allows them to often be re-assembled in their three-dimensional forms. While many are incomplete or single elements, a number of individuals are nearly complete, three-dimensional shells. Shells are often around or just over 20 cm in length and over two dozen ≥50% complete shells are known.

### Plastron

(Figs. 4 and 5; Figs. S1B, S2B, S3B, S4B–S4F, S5, S6C, S6D, S7C, S7D, S9, S10, S11C, S11D, S12B, S12C, S13B, S14C, S14D, S15, S16B, S17C, S17D, S18B–S18F, S19B, S20B, S21B, S22B–S22D, S23B, S24C, S24D, S25B, S25C, S26C, S26D, S27C, S27D, S28, S29E–S29G and S30C–S30F)

The plastra all follow the general emydid shape and composition. The plastron is made up of two epiplastra, an entoplastron, two hyoplastra, two hypoplastra, and two xiphiplastra. On ETMNH-8549, the entire plastron measures approximately 20.50 cm anteroposteriorly. A suture runs medially through the plastron separating the two sides and the elements of the plastron that have pairs. It does not, however, run through the entoplastron, as is the case with other emydids and turtles in general.

### Sutures of the plastron

(Fig. 4)

**Epiplastra:** The epiplastra are the anterior-most bones of the plastron and contact the entoplastron posteromedially and the hyoplastra posterolaterally. They are each 40.10 mm maximum (anteroposterior) length by 43.20 mm maximum (mediolateral) width. The anterior border is commonly “shoveled” and is curved ventrally on either side of the medial suture between the two epiplastra. The anteromedial-most border varies slightly in thickness. Serrations are commonly present lateral to the medial suture, although not in ETMNH-8549. This is generally from wear, presumably a taphonomic feature on the specimen, which has a generally slightly worn appearance. The serrations become more pronounced laterally, with the lateral-most projection on either epiplastron the largest and most pronounced. These are the only projections that can also be seen posteriorly on the visceral surface, creating two small visceral keels. At the posterior-most extent of these projections on the visceral surface, a depression lies slightly medially. Both slightly “tear-drop” shaped depressions are angled anteromedially. At the medial suture between the “tear-drop” depressions, the visceral surface rises slightly dorsally. Lateral to the most pronounced projections, the lateral edges of the plastron bend slightly ventrally, and both thin posteriorly. The epiplastra wrap around both anterolateral sides of the entoplastron. The posterior borders of the epiplastra project anterolaterally to the lateral edge. The suture between the epiplastra and hyoplastra exhibits a sigmoidal curve as well.

**Entoplastron:** The entoplastron is a subtriangular bone medioposterior to the epiplastra and contacting the epiplastra anteriorly and laterally, and the hyoplastra posteriorly and laterally. This shape in the entoplastron is common in most turtles, specifically emydids. In ETMNH-8549, the element measures 26.70 mm maximum length and 37.65 mm maximum width. All entoplastra specimens from *T. haugrudi* have a greater width than length. Ventrally the entoplastron is slightly rounded posteriorly, although it can sometimes have a small posterior projection medially. The lateral-most points are often somewhat rounded, although they do become more tapered in some specimens. The anterolateral border is often bowed medioventrally and, in fact, is also commonly a sigmoidal curve, although this bowing can vary between very slight to more pronounced. Anteriorly, the entoplastron is well rounded, although it can sometimes have a more pronounced point medially. Viscerally, the entoplastron appears wider mediolaterally and shorter anteroposteriorly. Anteriorly, there is a rounded indentation that groups closely with the two “tear-drop” indentations on the epiplastra. Posterior to the indentation, the visceral surface projects dorsally and becomes a bit wider dorsoventrally. There is a projection at the medioposterior-most point of the entoplastron on the visceral surface. This projection also exhibits a dorsal keel, which stops abruptly on the posterior-most point of the entoplastron and is not found on the hyoplastron, or any other part of the visceral surface of the plastron.

**Hyoplastra:** The hyoplastra consist of a pair of sub-rectangular bones that contact the epiplastra anterolaterally, the entoplastron anteromedially, the hypoplastra posteriorly, and the peripherals of the carapace laterally as the anterior part of the bridge. The medial border of each hyoplastron is smaller and more constricted anteroposteriorly than the lateral borders (assuming one measures the whole element and not just the bridge section that contacts the carapace). The two hyoplastra are commonly offset, as in ETMNH-8549, but this is not always the case, as in ETMNH-11642. In ETMNH-8549, the maximum width of each hyoplastron is approximately 72.10 mm, while the maximum length is 44.25 mm medially for the right hyoplastron, versus 51.22 mm for the left hyoplastron. Laterally, the maximum length is 70.65 mm for both sides. Due to the rounded and curved shapes of the entoplastron and epiplastra, the anterior border of the hyoplastra have various curves, although there is often an anteromedial projecting point of the hyoplastra found at the contact of the epiplastra and entoplastron on the anterior border. The medial and posterior borders of the hyoplastra are both relatively straight. Laterally, the hyoplastra make up the anterior portion of the bridge of the shell. The anteroposterior length on the lateral surface of the hyoplastra is comprised of approximately 31.50 mm anterior to the bridge and approximately 40.60 mm of the bridge. Comparatively, there is approximately 54.65 mm of contact between the bridge-portion of the hyoplastron and the carapace. The contact between the carapace and plastron at this location is often somewhat convexly curved. The bridge-portion itself is similar to other emydids. Viscerally, the hyoplastra have relatively few distinguishing characteristics. It is a relatively flat and smooth surface, only changing laterally to make up part of the bridge.

The anterior portion of the plastron is similar to other deirochelyines. The portion of the epiplastra covered by the gular projects farther anteriorly than any other part of the plastron. It is often somewhat inflated. The two lateral projections on the epiplastra mark a sharp contrast where the plastron cuts back posteriorly. Posterior to this, the lateral edges of the plastron are well rounded, specifically under the humeral scutes. This large convex lateral curve pinches at the contact between the humerals and pectorals. Posterior to this, the plastron expands laterally again to make up the anterior-part of the bridge. The bridge projects anterodorsally over part of the hyoplastra under the humeral scutes.

**Hypoplastra:** The hypoplastra lie posterior to the hyoplastra and contact the hyoplastra anteriorly, the xiphiplastra posteriorly, and the peripherals of the carapace laterally as the posterior part of the bridge. Unlike the medial border of the hyoplastra, the medial border of each hypoplastron is generally the same length as the lateral border. The two hypoplastra can be offset as well, as in ETMNH-8549, but this coincides with the hyoplastra, so that if the latter are offset, the former will be as well. In ETMNH-8549, the width of each hypoplastron is 67.25 mm and the maximum length is 63.10 mm medially for the right hypoplastron versus 56.64 mm medially for the left hypoplastron. Laterally, the maximum length is 64.15 mm for both sides. When the sutures are offset between the hyo- and hypoplastra (as in ETMNH-8549), the total anteroposterior width of both sets of elements should still be essentially equal. The anterior border of the hypoplastron commonly has a slightly concave posterior curve while the medial border is straight. Compared to the hyoplastron, the bridge-portion makes up a greater majority of the hypoplastron. There is approximately 21.75 mm posterior to the bridge, while the bridge-portion of the hypoplastron is approximately 43.50 mm, and the bridge contact between the hypoplastron and carapace is 50.57 mm long. The contact between the carapace and plastron at this location is often somewhat convex, although it curves ventrally in its posterior-most region. The bridge-portion itself is similar to other emydids. Posterior to the bridge, the hypoplastron flares laterally, while coming to a point lateroposteriorly. This lateroposterior point is the posterior-most point of the hypoplastron. The visceral surface is quite smooth, with little distinct morphology other than what is common among Testudines. As is also common with turtles, there is a medial “lump” that is raised dorsally. The medial “lumps” on each hypoplastron connect with each other, leaving a raised area on the visceral surface of the plastron in the pelvic region.

**Xiphiplastra:** The xiphiplastra are the posterior-most bones of the plastron and contact the hypoplastra anteriorly. These elements exhibit a significant amount of notching, especially apparent when both are present. The xiphiplastron has a maximum length of 51.20 mm and a maximum width of 46.62 mm. The medial suture between the two xiphiplastra, however, only has a maximum length of 44.30 mm. The notching is prevalent between the two xiphiplastra and on the lateral border at the contact between the femoral and anal scutes. Small serrations are common on well-preserved xiphiplastra, especially at the apex of the convex-curved surfaces. The medial sutural contact between the two xiphiplastra is straight. Viscerally, there is a lip where the anal scutes terminate. There is a dorsally raised region where this termination nears the anterior border of the xiphiplastra, which leads to the bridge on the anterior hypoplastra.

The posterior portion of the plastron is similar to other deirochelyines, specifically *Trachemys*, *Pseudemys* and *Graptemys*. However, the notching is prominent on the medioposterior border between the two xiphiplastra and the lateral point of the sulcus between the femoral and anal scutes. The posterior plastron is also convex laterally anterior to the notching and this curve is formed by the hypoplastron anteriorly and the xiphiplastron posteriorly. There is sometimes a slight mediolateral “pinching” between the hypoplastron and the xiphiplastron, but when present it is usually small. The large convex lateral curve terminates at the contact between the femoral and anal scutes (anterior-most notch). Anterior to this, the plastron expands laterally to make up the posterior portion of the bridge. The bridge projects posterodorsally over part of the hypoplastra under the femoral scute.

### Sulci of the plastron

(Fig. 4)

The surface of the plastron is covered with a number of scutes (or scales), of which the sulci (or seams) left behind can give an indication of their morphology and appearance. *T. haugrudi* is interpreted as having a pair of gular scutes, a pair of humeral scutes, a pair of pectoral scutes, a pair of abdominal scutes, a pair of femoral scutes, and a pair of anal scutes. One specimen (ETMNH-11643, Fig. S13B) had an oval scute located medially between the abdominals and the femorals. This feature was not present on any other specimens and is considered an aberrant feature or supernumerary (or extra) scute, not characteristic of *T. haugrudi*. The overall plastral formula for *T. haugrudi* is abdominal > anal > gular > femoral > pectoral > humeral.

**Gulars:** The gular scutes lie medially on the epiplastra and the entoplastron. The border between the gular and humeral scutes is located lateral to the most pronounced projections on the epiplastra. It commonly makes an angle of roughly 70° and comes to a posterior point in the middle of the entoplastron. The border between the gulars and the humerals is not completely straight, and is slightly bowed posterolaterally. On the visceral surface, the gulars terminate just anterior to the two “tear-drop” depressions. They project slightly more posterior lateral to the two depressions. This also helps give a slightly concave medial curve to this portion of the gular–humeral sulcus. The depressions, being located posterior to the gulars and medial to the humerals, are not covered by any scutes. The gulars also exhibit a long overlap on the visceral surface.

**Humerals:** The humeral scutes lie on portions of the epiplastra, the entoplastron, and the hyoplastra. They do not project anterior on the plastral lip like the gulars but do pinch down medially. They have a large convex curve laterally, giving the anterior portion of the plastron an inflated look. Posteriorly, the humerals are relatively flat, with only a slight anterolateral curve until the lateral-most portion. Laterally, on the posterior sulcus between the humerals and pectorals, there is a sharp convex anterior curve. Medially, portions of the humerals are almost evenly spaced on the epiplastra, the entoplastron and the hyoplastra, although the hyoplastra generally possess the smallest portion. The humerals exhibit a long overlap, as it is a continuation from the gular overlap.

**Pectorals:** The pectoral scutes are smaller than the humeral, abdominal, and femoral scutes and lie on the hyoplastra. They are generally anteroposteriorly short. As is the case with all the contacts, the anterior border of the pectoral coincides with the posterior border of the humeral, and, therefore, the same characteristics apply to both. There is a generally convex posterior curve to the pectoral, as is common in deirochelyines. As the femoral reaches the bridge, however, there is a relatively sharp convex anterior curve. Laterally along the bridge, the femoral contacts the axillary scute and marginals IV and V. Due to the curvature of the posterior border of the femoral, its posterior-most point lies laterally on the hyoplastra. The pectorals exhibit a long overlap, although this quickly becomes less significant as it nears the bridge posteriorly.

**Axillaries:** The axillary and inguinal scutes are located anteriorly and posteriorly on the bridge. The axillary scute is relatively small and contacts the pectorals posteriorly, marginals III anteriorly, and marginals IV laterally while lying on the hyoplastra. Its anterior and lateral surfaces are relatively straight. Its posterior border, however, is not and, instead, exhibits a more complicated sigmoidal curve. The axillary scute has a generally sub-rectangular shape.

**Inguinals:** The inguinal scute, on the other hand, is located posteriorly on the bridge and is larger than the axillary scute. It lies on the hypoplastra. The inguinal scute projects anteriorly onto the bridge while the axillary scute is almost exclusively confined anterior to the bridge. The inguinal scute barely contacts marginal VI, but more completely contacts marginals VII and VIII laterally and posteriorly, along with the abdominal medially. The inguinal scute has a generalized sub-triangular shape.

**Abdominals:** The abdominals are the largest scute set on the plastron. They cover a majority of the bridge, and up to roughly one-third of the total ventral surface of the plastron, while lying on the hyo- and hypoplastra. Just as with the posterior border of the humerals discussed above, the lateral-most portion of the anterior border is convexly curved. The lateral edges of the abdominals contact the marginals along the bridge. The abdominals somewhat pinch out laterally though, so that they only contact marginals VI. Laterally, they also contact the inguinal scutes. Due to the wedge-like (sub-triangular) shape of the inguinal scute, the posterolateral edge of each abdominal curves medially. Therefore, the anterior border of the abdominal is wider than the posterior border. The posterior border of the abdominal is similar to the posterior border of the pectoral, with a generally convex posterior curve. The abdominals exhibit virtually no overlap on the visceral surface.

**Femorals:** The femorals are located posterior to the abdominal scutes and anterior to the anal scutes, while lying on the hypo- and xiphiplastra. Similar to the humerals, the lateral edges of the bone under the femorals flare out laterally and appear inflated. The anterior notches of the posterior plastron are present at the posterolateral edges of the femorals and this gives the femorals a relatively sharp point posterolaterally. Immediately medial to the notches, the posterior border of each of the femorals angles anteriorly on both sides of the plastron and contact each other at the medial contact of the plastral elements. The femorals exhibit a long overlap, although this quickly becomes less significant as it nears the bridge anteriorly.

**Anals:** The anals are the most posterior scutes on the plastron and lie on the xiphiplastra. The medial contact is generally quite flat, and the anterior border makes a sharp anterior angle (more constricted, discussed above with the femorals). Individually the anals are sub-trapezoidal to sub-triangular, depending on how rounded or pointed the posterior-most portion of the plastron is. The prominent notching is easily visible in the anals as well, as there are notches posteromedially and anterolaterally at the lateral contact of the anal and the femoral. On the visceral surface, there is a depression that runs around the medial termination of the anal. This depression, however, is normally more prominent on the posterior plastron than on the anterior plastron. The anals exhibit a relatively long overlap on the visceral surface, although it is not as significant as the overlap exhibited by the gulars, humerals, pectorals, or femorals.

### Carapace

(Figs. 2A, 3 and 5; Figs. S1A, S2A, S3A, S4A, S4C–S4F, S6A, S6B, S7A, S7B, S8, S11A, S11B, S12A, S12C, S13A, S14A, S14B, S16A, S17A, S17B, S18A, S18C–S18F, S19A, S20A, S21A, S22A, S22C, S22D, S23A, S24A, S24B, S25A, C, S26A, S26B, S27A, S27B, S29A–S29D, S30A, S30B, S30E and S30F)

There are several well-preserved carapaces, either partial or nearly complete. These give a good indication of the general size, shape, and characteristics of *T. haugrudi*. ETMNH-8549 is a medium-sized individual, but one that has clearly reached adulthood based on fusion of the shell and carapace elements. The carapace is approximately 20.13 cm long and 17.00 cm wide. The carapace of ETMNH-8549 is nearly complete, and is only missing neural I, the medial part of the left costal VI, small fragments of right costals I and II, the lateral part of right costal VII, small fragments from right peripherals IV, V and VI, and right peripheral VII. The carapace of *T. haugrudi* comes in two different morphs; a more highly domed morph (ETMNH-8549, Figs. 3 and 5), and a somewhat flattened morph (ETMNH-6935, Fig. S4). These two morphs may represent sexual dimorphism. Previous studies have shown that females from a species may possess higher domed shells, while males can have flatter shells (Kaddour et al., 2008; Vega & Stayton, 2011). A median keel is present on parts of the carapace, including the neurals, and is specifically on the nuchal and the posterior-half of the shell. When the keel is present on the neurals, it is often immediately followed, both posteriorly and laterally, by a depression. These depressions form angles that point posteriorly and appear to end just lateral to the neural arch articulation with the neurals. Posteriorly on the shell, in lateral view, is a medioventral inflection of the shell. This inflection occurs around neural VII and deflects back posterodorsally around neurals VIII and IX. This general inflection, or depression, is present on all specimens and is not considered a taphonomic or preservational artifact, but a natural characteristic. Anteriorly, there is some double-notching of the carapace. This occurs when there is a medial concave curvature between the elements (peripherals) and the scutes (marginals). These two inward curves create two sets of notches, although the notching between the marginals is always more pronounced than that between the peripherals. Posteriorly, the double-notching becomes more prominent, with the projecting shell elements becoming thinner and the notches, specifically those between the marginals, becoming deeper.

### Sutures of the carapace

(Figs. 3 and 5)

**Nuchal:** The nuchal of *T. haugrudi* is similar in morphology to other fossil *Trachemys*, specifically *T. inflata* (Fig. 2). It is complete in ETMNH-8549 and, indeed, there are several complete nuchal bones of *T. haugrudi* preserved. They all tend to share the same general morphology. The nuchal is slightly wider (49.55 mm in ETMNH-8549) than long (48.15 mm in ETMNH-8549), and all current nuchal specimens adhere to this (Table 1). The nuchal exhibits prominent notching on the anterior border. This notching, while somewhat variable, in all instances has the medial projection (the bone under the cervical scute) protruding close to, but not quite as anteriorly, as the two lateral projections (bone under the two marginals I) in ETMNH-8549. The two anterolateral edges of the nuchal exhibit a concave curve posteromedially. The two lateral sides come to a sharp point where they meet the two costals I, and reverse direction with a slight curve anteromedially, or have an essentially straight border. The posterior-most border of the nuchal contacts neural I and has a concave curve anteriorly. The dorsal surface of the nuchal shows heavy inflation (discussed further below with the sulci of the carapace). The posterior half of the nuchal under vertebral I shows the anterior portions of a median keel. On the visceral surface, there is a slight depression around the sulcus of the cervical scute. There is also a depression around the posterior termination of the cervical scute and marginals I. Lateral to this depression are two slight costiform processes. These processes are faint and continue onto the medial portion of peripheral I. This latter depression has a sigmoidal curve, with it curving concavely anterior at the cervical and convexly anterior at the marginals I. Viscerally, there is a steep inflection at this depression, and the nuchal curves dorsally sharply at this lip. The lip itself is the most robust portion of the nuchal, and the posterior-most portion becomes increasingly thin and gracile. At the posterior-most point, the anterior-most section of the neural attachment for the thoracic vertebrae is present.

**Table 1:** Measurements of the sagittal bones and scutes of *Trachemys haugrudi* (ETMNH-8549).

	Maximum length	Maximum width
*Bones*		
Nuchal	48.15	49.55
Neural I	25.07	20.18
Neural II	22.97	21.88
Neural III	24.67	23.08
Neural IV	21.95	25.95
Neural V	21.00	26.64
Neural VI	24.65	16.51
Neural VII	16.25	19.88
Neural VIII	13.25	12.68
Suprapygal I	10.33	9.28
Suprapygal II	21.17	30.16
*Scutes*		
Cervical	19.21	7.20
Vertebral I	38.50	41.20*
Vertebral II	47.19	55.54
Vertebral III	51.16	59.05
Vertebral IV	44.62	53.50
Vertebral V	46.18	51.06

**Notes:**

Lengths correspond to the anteroposterior (or sagittal) measurement(s) and the widths correspond to the resultant perpendicular measurement(s). All measurements in mm.

* Maximum width measured posteriorly.

**Neurals:** The neurals are nearly all known from ETMNH-8549 (neural I is missing), although the entire neural column is known from the referred specimens. All specimens possess, or are inferred to possess, a series of eight neurals, and these neurals all exhibit similar neural morphology, with most being sub-hexagonal to sub-ovoid. While some are longer than wide (neurals I–III, VI, VIII), others are wider than long (neurals IV, V, VII). Measurements of the neurals are provided in Table 1. Neural I is missing in ETMNH-8549, but measurements can still be inferred, with a maximum length of 25.07 mm and a maximum width of 20.18 mm. Neural I is the longest neural in the series. It contacts the nuchal, costals I, and neural II, while having an oval shape, with all its borders slightly convex. It has a convex posteriorly curved depression on the surface where vertebrals I and II contact each other. Neural 1 projects slightly laterally at the junction of vertebral I and II and pleural I. It possesses the anterior portion of a slight median keel as continued from the posterior portion of the nuchal, but posteriorly on neural I under vertebral II this keel changes abruptly to a depression. The visceral surface of neural I, similar to that of neurals II–VIII, is essentially smooth and flat except for the neural attachment of the thoracic vertebrae.

Neural II is generally as wide as neural I, albeit a little shorter, and contacts costals I and II, along with neurals I and III. In ETMNH-8549, neural II has a maximum length of 22.97 mm and a maximum width of 21.88 mm. Anteriorly, neural II is concave congruent with the convex curve of the posterior edge of neural I. Laterally, neural II angles laterally for a short distance (approximately 3–4 mm) where it contacts the borders of costals I and II. The lateral border of neural II then angles medially until its posterior edge. In ETMNH-8549, as in other *T. haugrudi* specimens, this posterolateral border has a somewhat sigmoidal curve. Posteriorly, neural II has a convex curve. On the dorsal surface, it exhibits an axial depression. This is less prominent on some specimens that are considered taphonomically smoothed and/or weathered. Two slightly raised ridges appear lateral to the medial depression and these angle medially toward the posterior of neural II.

Neural III is prominent and contacts costals II and III, along with neurals II and IV. Its anterior edge is concave as it contacts the convex posterior border of neural II. Neural III has a maximum length of 24.67 mm and a maximum width of 23.08 mm in ETMNH-8549 (making it the second longest neural in the series). It also exhibits a slight lateral projection (approximately 4–6 mm distance from anterior edge) anteriorly as in neural II, and then angles slightly medially posterior to this lateral projection. The posterior border is generally slightly convex, although it can be nearly flat as well. Its lateral borders can also be slightly convex, opposite the condition in neural II. There is a thin depression (=sulcus) that runs laterally on the posterior portion of neural III. This sulcus coincides with the contact between vertebrals II and III and is concave anteriorly. There is a slight lateral projection of neural III at the contact between vertebrals II and III, similar to the condition in neural I. The two ridges in the posterior portion of neural II continue onto the anterior portion of neural III. This ridge pinches out and is surrounded laterally by another sulcus. This sulcus comes to a point on the anterior half of neural III.

Neural IV is also prominent in the column and contacts costals III and IV, along with neurals III and V. In ETMNH-8549 it has a maximum length of 21.95 mm and a maximum width of 25.95 mm, making it the first (or anterior-most) neural that is wider than long (neurals IV, V, VII). Similar to other neurals, the anterior edge of neural IV is concave. While it does exhibit the slight lateral projection anteriorly as in neurals II and III, this projection is now larger and situated more posteriorly on the neural (6–9 mm from the anterior edge). The posterolateral borders are also straighter and less concave. The posterior border is nearly completely flat, with only an inconspicuous concavity. On the dorsal surface, a slight median keel is barely visible.

Neural V is also prominent and contacts costals IV and V, along with neurals IV and VI. Neural V in ETMNH-8549 has a maximum length of 21.00 mm and a maximum width of 26.64 mm, making it the widest neural. The borders of neural V are similar to those of neural IV with a concave anterior border and, as is the case with neural IV, exhibits a larger lateral projection on its anterior end (7.0–9.5 mm from the anterior edge). Posterior to this projection, the lateral edges are slightly concave. The posterior edge is flat mediolaterally. The median keel is more prominent on neural V than on neural IV. There is a prominently deep, but thin, lateral depression that is concave posteriorly marking the sulcus between vertebrals III and IV. The curvature of this sulcus is more prominent than that on neural I or III.

Neural VI is the most hexagonal neural and contacts costals V and VI, along with neurals V and VII. Neural VI in ETMNH-8549 has a maximum length of 24.65 mm and a maximum width of 16.51 mm, also making it longer than wide, similar to the first three neurals (neurals I–III). The anterior portion of the lateral border to the lateral-most points measures 8.5 mm, while the posterior portion measures 9.5 mm. The anterior portion is straight, while the posterior portion is slightly concave medially. The posterior border is also straight, similar to the posterior border of neural V. The median keel is especially prominent on the dorsal surface of neural VI.

Neural VII is similar to the hexagonal neural VI, although it is smaller. It contacts costals VI and VII, along with neurals VI and VIII. In ETMNH-8549, the maximum length is 16.25 mm and the maximum width is 19.88 mm. The anterior and posterior portions of the lateral border, separated by the lateral projection, are roughly equal, although this is slightly skewed anteriorly in ETMNH-8549.

Neural VIII is a rather small bone that contacts costals VII and VIII, along with neural VII and suprapygal 1 (anterior suprapygal). In ETMNH-8549, neural VIII has a maximum length of 13.25 mm and a maximum width of 12.68 mm, making it the smallest neural. The element is sub-rectangular with two small lateral projections anteriorly. Anterior to these lateral projections measures only 2.7 mm, while posterior to these there is 10.9 mm of lateral border. The posterior lateral borders are generally slightly concave and the posterior border is straight. On the dorsal surface, the median keel is readily visible. Posteriorly on the dorsal surface of neural VIII, there is a depression that represents the sulcus between vertebrals IV and V. This depression is normally found on neural VIII and is concave posteriorly. However, the location of the depression that represents the sulcus between vertebrals IV and V can shift slightly, and is even found on the posterior-most portion of neural VII in some specimens, such as ETMNH-11643. On the visceral surface, the neural attachment decreases in size posteriorly until it is completely gone. Therefore, the neural formula is 4/6–4/6–6–6–6–6–6–4. Generally, the intervertebral sulci cross the middle of neurals I and III, the posterior of neural V and the anterior of neural VIII.

**Suprapygals:** Suprapygal I, or the anterior suprapygal, has a maximum length of 10.33 mm and a maximum width of 9.28 mm in ETMNH-8549 (Table 1). It contacts costals VII and VIII, suprapygal II (posterior suprapygal), and neural VIII. Suprapygal I is generally ovoid, with its widest point posteriorly. It is generally flat along its anterior border, and is slightly convex along its lateral and posterior borders. The anterior suprapygal still exhibits a prominent median keel on its dorsal surface. On the visceral surface, the neural attachment for the thoracic vertebrae is no longer present, but a faint, uneven surface is present medially.

Suprapygal II, or the posterior suprapygal, is similar in morphology to other deirochelyines, with a maximum length of 21.17 mm and a maximum width of 30.16 mm in ETMNH-8549 (Table 1). It contacts costal VIII, suprapygal I, peripherals XI, and the pygal. It is sub-round to sub-ovoid, while being slightly concave on its anterior end with two laterally projected points on its lateral borders. With respect to these points, the anterior portion of the lateral border is longer and more prominent than the posterior portion (16.41 versus 11.81 mm, respectively). On the dorsal surface, the median keel is still present on the anterior-half of suprapygal II. Lateral to both sides of the median keel is a depression. Posterior to the median keel, the posterior suprapygal has a kind of inflection point where it dips down ventrally and its dorsal surface becomes flatter.

**Pygal:** The pygal is the most posterior midline bone of the carapace. It contacts suprapygal II and the left and right peripherals XI and is prominently notched. In ETMNH-8549, the pygal has a maximum length of 28.18 mm to the distal point, a minimum length of 17.85 mm to the anterior-most point of the notch, and has a maximum width of 24.15 mm (Table 2). Its anterior and lateral borders are quite flat. The two posteriorly projecting points are of equal length. Dorsally, there is a pronounced depression at the sulcus between vertebral V and the right and left marginals XII. There is a second, perpendicular depression between the two marginals XII on the posterior portion of the pygal. The bone beneath the scutes on the pygal, specifically under the marginals, is heavily inflated. The posterior notch on the pygal makes an angle of approximately 50°. The pygal has a ventral curve, making it more dorsoventrally oriented than most of the surrounding peripherals except for the medial portion of peripherals XI.

**Table 2:** Measurements of the bones and scutes around the rim of *Trachemys haugrudi* (ETMNH-8549).

	Maximum length	Minimum length	Maximum width
*Bones*			
Peripheral I	28.40	24.15	20.00*
Peripheral II	28.62	26.47	25.48*
Peripheral III	27.90	–	33.92**
Peripheral IV	28.02	–	29.17
Peripheral V	30.12	–	28.30
Peripheral VI	32.34	–	29.67
Peripheral VII	33.38	–	32.00
Peripheral VIII	39.32	30.30	25.46
Peripheral IX	37.60	28.80	21.40
Peripheral X	33.64	23.79	21.18
Peripheral XI	32.60	24.40	20.47
Pygal	28.18	17.85	24.15
*Scutes*			
Marginal I	26.90	–	20.32
Marginal II	22.70	–	18.81
Marginal III	18.11	–	24.03
Marginal IV	19.04	–	27.88
Marginal V	18.02	–	29.26
Marginal VI	18.40	–	31.95
Marginal VII	23.12	–	31.68
Marginal VIII	29.10	–	28.23
Marginal IX	22.26	–	28.15
Marginal X	21.60	–	26.15
Marginal XI	21.96	–	30.42
Marginal XII	24.28	–	19.77

**Notes:**

Lengths correspond to the proximodistal measurement(s) and the widths correspond to the resultant perpendicular measurement(s). Minimum lengths are the measurement to the notch. All measurements in mm.

* Width measured distally.

** Width measured on ventral surface.

**Peripherals:** The peripherals are all present in either ETMNH-8549 or other referred specimens. There is a total of 22 peripherals (11 on each side), with many of the anterior and posterior peripherals exhibiting significant notching. The peripherals described here will be considered from the left side of the specimen unless otherwise noted. Measurements of the peripherals are listed in Table 2, with lengths corresponding to the proximodistal measurement(s) and the width corresponding to the resultant perpendicular measurement(s). Medially the peripherals exhibit a significant longitudinal wrinkled texture above which the pleurals would lie. On some specimens, these wrinkles appear relatively evenly spaced, while on others their spacing is less consistent. These wrinkles do not follow the outline and are not consistent with the geometry of the pleurals, but instead wrap longitudinally around the carapace. Peripheral I is located immediately lateral to the nuchal and contacts peripheral II and costal I. It exhibits prominent notching distally. In ETMNH-8549, peripheral I has a maximum length of 28.40 mm (proximodistally), a minimum length of 24.15 mm (proximal to notch), and a width of 20.00 mm distally (maximum width) and 9.12 mm proximally. The medial border exhibits a slightly sigmoidal curve, with the anterior (=distal) portion being concave medially, while the posterior (=proximal) portion is convex medially. It is laterally constricted along its proximal border, which is slightly convex, and its lateral border is straight. Dorsally, there are prominent depressions in the sulci between marginals I and II and pleural I. The medial distal projection is more curved and generally wider than the lateral distal projection. The bone under the carapacial scutes, specifically under the marginals, is heavily inflated. The visceral surface is quite smooth, with a steep dorsal inflection proximal to the termination of the marginals and the costiform processes, running laterally on the visceral surface of the nuchal, continue onto the medial portion of the visceral surface of peripheral I.

Peripheral II, located lateral to peripheral I and in contact with costal I and peripheral III as well, is similar to peripheral I, although the distal (=anterior) notching is not as prominent. The lateral borders are both relatively straight, although they angle medially to make the proximal border thinner than the distal border. The proximal border is still convex. In ETMNH-8549, the maximum length is 28.62 (proximal to distal projection), the minimum length is 26.47 mm (proximal to notch), and the width is 25.48 mm distally (maximum width) and 17.15 mm proximally. Anterior peripherals tend to be shorter and more robust (thicker or inflated) than those from the posterior of the carapace. The medial distal projection is smaller than the lateral distal projection. Dorsally, the depressions present in peripheral I are also present on peripheral II, although they instead now derive from the sulci of marginals II and III and pleural I. There is a slight distally projecting point of the inflated bone under pleural I. The bone under the carapacial scutes is, again, inflated, although not to the extent of peripheral I, and the visceral surface of peripheral II is similar to that of peripheral I.

Peripheral III is posterolateral to peripheral II and contacts costal I, peripheral IV, and the hyoplastron as part of the bridge. In ETMNH-8549, peripheral III has a maximum length (proximodistal) of 27.90 mm, with a width of 27.70 mm on its dorsal surface and 33.92 mm on its ventral surface. Peripheral III is similar to peripherals I and II, but the distal notching is less prominent. The anterior (~medial border of peripheral II) and posterior (~lateral border of peripheral II) borders are similar to those of peripheral II, although the element is becoming wider and the proximal border is becoming straighter. There is also a small notch distally and, while the more anterior (=medial of peripheral II) projection is still less wide than the posterior (=lateral of peripheral II) one, it also projects farther distally. Dorsally, the triad conjunction of sulci is still present, although it is now between pleural I and marginals III and IV. Inflation is less prominent than in peripherals I and II, and the anterior projection is more prominent than the posterior one. On the visceral surface, peripheral III is becoming more robust as it inflates to become the anterior portion of the bridge, with a lip that runs posteroventrally to anterodorsally and a large depression.

Peripheral IV is the first peripheral that is entirely part of the bridge and contacts costals I and II proximally, peripheral III anteriorly, peripheral V posteriorly, and the hyoplastron proximoventrally. Peripheral IV has a maximum proximodistal length of 28.02 mm and a maximum width of 29.17 mm in ETMNH-8549. Distally, it has a slight lip where the projections that make up the notching on the anterior peripherals would be present. Distodorsally, there are depressions in conjunction with the carapacial sulci as in the other peripherals. It is covered, however, by pleurals I and II and marginals IV and V, meaning it has another depression running proximodistally on the posteromedial portion of its dorsal surface. The sulci point somewhat ventrodistally at their junction. Peripheral IV is similar to other turtle bridge elements in that the entire element creates a distal (or lateral) peak (like a house roof tipped on its side).

Peripheral V is the anteromedial bridge element of the carapace and contacts costals II and III, peripherals IV and VI, the hyoplastron, and the hypoplastron. In ETMNH-8549, peripheral V has a maximum length (proximodistal) of 30.12 mm and a maximum width of 28.30 mm. Its borders and general external (dorsal and ventral) morphology are similar to that of peripheral IV. It does, however, form an angled contact with the hyoplastron and the hypoplastron, creating a proximal point on its ventral surface. The visceral surface is quite smooth with a relatively sharply angled median depression. The entire element is, similar to peripheral IV, distally (or laterally) peaked.

Peripheral VI is the posteromedial bridge element of the carapace and contacts costals III and IV, peripherals V and VII, and the hypoplastron. In ETMNH-8549, peripheral VI has a maximum length of 32.34 mm and a maximum width of 29.67 mm. Its borders (all generally straight) and general morphology are similar to that of peripheral V. Its distal (or lateral) lip, however, is more prominent than that of peripheral V. The notching again begins posteriorly, with the posterior projection being more prominent than the anterior one. Peripheral VI marks the transition from the anterior projection being more prominent (in peripheral V) to the posterior projection being more prominent, although both are equivalent as projecting distally. Dorsally, the bones under the marginals are more inflated than those under the pleurals. The visceral surface of peripheral VI is similar to that of peripheral V. The element is, as in peripheral V, distally (or laterally) peaked.

Peripheral VII is the posterior-most carapacial element of the bridge and contacts costals IV and V, peripherals VI and VIII, and the hypoplastron. In ETMNH-8549, peripheral VII has a maximum length of 33.38 mm and a maximum width of 32.00 mm. Bridge peripherals (III–VII) tend to be similar in size, so morphology is the main distinguishing feature. Peripheral VII is similar in general morphology to peripheral IV, although everything is mirrored toward the posterior-portion of the shell. The notching is, again, becoming more prominent. The posterior projection is distinctly more prominent than the anterior one, and this is where the characteristic deep posterior notches of the carapace begin anteriorly. Dorsally, its surface is nearly the same as peripheral VI, except for its more laterally projecting posterior projection. It also, however, has the sulcus between pleurals II and III anteriorly, and this sulcus can sometimes lie almost directly dorsal to the suture between peripherals VI and VII. Peripheral VII projects somewhat ventroproximally on its ventral surface, specifically anterior, to make up a portion of the bridge. Its visceral surface is smooth, but also possesses a similar lip and depression to that of peripheral III, although it is its posterior opposite. The bridge is mainly composed of peripherals IV–VII, the hyoplastron, and the hypoplastron.

Peripheral VIII is the first prominently notched peripheral on the posterior-half of the shell and contacts costals V and VI, peripherals VII and IX, and the hypoplastron. Peripheral VIII in ETMNH-8549 has a maximum length (proximal to distal projection) of 39.32 mm, a minimum length (proximal to distal notch) of 30.3 mm and a maximum width of 25.46 mm. As stated above, the deep posterior notching becomes more pronounced on peripheral VIII, while the posterodistal surface of the posterior notch is slightly concavely curved. The proximal border of the dorsal portion is convexly curved. The sulcus between marginals VIII and IX lies distal (=lateral) to the notch, as is the case with all the notches of the peripherals. The depression of the sulci between pleural III and the marginals is, again, concave distally and comes to a slight distal projecting point where all three sulci meet. The distal and/or posterior projections of the peripheral tend to sweep posteriorly, as does the angle of the notch between them. Dorsally, the bone under the scutes is again inflated, specifically under the posterior portion of the anterior marginal. Viscerally there is a slight depression (or lip) at the proximal termination of the marginals. There is also a steep dorsal inflection at this lip, and the thickest, or most robust, area of the peripheral is where this inflection and lip occur. Anteriorly, on the visceral surface, the peripheral becomes more robust as well and is slightly inflated to support a posterior portion of the bridge.

Peripheral IX is a continuation of peripheral VIII and contacts costals VI and VII and peripherals VIII and X. In ETMNH-8549 it has a maximum length (proximal to distal projection) of 37.60 mm, a minimum length (proximal to distal notch) of 28.80 mm, and a maximum width of 21.40 mm. While it is thinner and slightly longer than peripheral VIII, it is similar to the anterior peripheral in most aspects. It does, however, have a few key differences, including along the proximal border on the dorsal portion, where there is a proximal projection between costals VI and VII. The element is slightly scooped up dorsally. The visceral surface is relatively uniform with the same lip, proximal termination, and robust nature as present in peripheral VIII (discussed above).

Peripheral X is a further continuation of the posterior carapace notching found in peripherals VIII and IX. It contacts costals VII and VIII and peripherals IX and XI. Peripheral X has a maximum length (proximal to distal projection) of 33.64 mm, a minimum length (proximal to distal notch) of 23.79 mm, and a maximum width of 21.18 mm in ETMNH-8549. As stated above, the deep and prominent posterior carapacial notching is still present. While peripherals IX and X are similar, a key difference is the presence proximally on the dorsal portion of peripheral X of the sulcus between pleurals IV and V. The sulcus sweeps medially, and the sulcus between pleural IV and the marginals is well rounded (convex posteriorly). The junction between the sulci is present posteroproximally on the dorsal portion of peripheral X. The visceral surface is similar to that present in the two immediately preceding anterior peripherals, although the entire peripheral is slightly convexly curved posterodistally, which is especially apparent at the “lip” or medial (or proximal) termination on the visceral surface.

Peripheral XI is the last peripheral and contacts costal VIII, peripheral X, suprapygal II, and the pygal. It is a continuation of the deep posterior notching of the carapace, which is continued in the pygal as well. In ETMNH-8549, peripheral XI has a maximum length (proximal to distal projection) of 32.60 mm, a minimum length (proximal to distal notch) of 24.40 mm, and a maximum width of 20.47 mm. As was the case for the other posterior peripherals, the posterior portion of the bone under the anterior marginal (in this case marginal XI) is more inflated than the posterior one. Proximally it still tapers to a point or slight projection. Posteroproximally, its surface has a concave curve where it contacts the posterior suprapygal (suprapygal II). While its “anterior” (or anterolateral) projection is relatively straight, its posterior (=posteromedial) projection is more strongly curved posteroventrally. Its dorsal surface is less “scooped out” (concavely curved dorsally) than peripherals VIII–X. Similar depressions from the sulci are present on peripheral XI as on the preceding anterior peripherals, although the depression projects anterodistally toward the anterior edge of the dorsal surface. It is also similar on its visceral surface to the preceding anterior peripherals, although the posterodistal curve is not as prominent as in peripheral X.

**Costals:** The costals are similar to those of other aquatic turtles, and specifically those of other deirochelyines. *T. haugrudi* has a total of 16 costals (eight pairs). The costals described here will be considered from the left side unless otherwise noted. Proximally, the costals all exhibit a smooth surface on which the vertebral scutes would lie. Under the pleural scutes, however, there is significant surface texture. The majority of this texture is present as longitudinal wrinkles around the carapace which are situated latitudinally on each individual costal. Costal I is a distinct element that contacts the nuchal, neurals I and II, costal II, and peripherals I–IV. This many contacts mean that the outer borders of the costal have a distinct shape and tend to form a sub-half circle. In ETMNH-8549, costal I has a maximum proximodistal length (=“diameter”) of 61.66 mm and a maximum anteroposterior width (=“radius”) of 37.58 mm, with the measurements of the costals provided in Table 3. Its proximal border is slightly concave where it contacts neural I and angled where it barely contacts neural II. It has a generally convex anterior curve to its anterior and distal edges where it contacts the nuchal and peripherals I–III. Posterodistally it barely contacts peripheral IV at an angle. Its posterior edge is slightly curved convexly where it contacts costal II. There is a depression proximally on the dorsal surface where the sulci of vertebrals I and II and pleural I lie. The wrinkles on the dorsal surface are also slightly convexly curved as they wrap around the anterodistal edges of the carapace. On the visceral surface, costal I is generally smooth, although costal rib I is visible. Proximally (=medially) the rib projects ventrally where it would contact the thoracic vertebra. Along the entire length of the costal rib, however, there is a ridge running on the ventral surface of costal I. This ridge is faint proximally, but becomes more pronounced distally until it reaches the axillary buttress of the bridge and peripherals III and IV. While peripheral III is slightly deflected ventrally with the costal rib, the majority is connected to peripheral IV. The ridge and, therefore, costal rib are both concavely curved anteriorly. There is a prominent “tear-drop” depression posterior and lateral to the ridge, which works in conjunction with the rest of the depression found on peripherals III and IV to form part of the bridge.

**Table 3:** Measurements of the costal bones and pleural scutes of *Trachemys haugrudi* (ETMNH-8549).

Bones	Maximum length		Maximum width
Costal I	61.66		37.58
	**Maximum length**	**Proximal width**	**Distal width**
Costal II	71.65	22.90	27.83
Costal III	74.56	27.00	30.50
Costal IV	74.97	22.37	23.98
Costal V	68.95	21.08	32.27
Costal VI	54.50	16.07	17.25
Costal VII	44.26	11.15	19.95
Costal VIII	35.64	19.47	17.92
**Scutes**	**Maximum length**		**Maximum width**
Pleural I	64.31		59.35
	**Maximum length**	**Proximal width**	**Distal width**
Pleural II	75.10	47.14	66.48
Pleural III	65.04	41.24	44.68
Pleural IV	49.04	27.14	41.37

**Note:**

Lengths correspond to the proximodistal measurement(s) and the widths correspond to the resultant perpendicular (anteroposterior) measurement(s). All measurements in mm.

Costal II is similar to the posterior costals in form and general morphology and contacts neurals II and III, costals I and III, and peripherals IV and V. It is sub-rectangular, as is common of the middle to posterior costals. In ETMNH-8549, costal II has a maximum length of 71.65 mm, a proximal width of 22.90 mm, and a distal width of 27.83 mm. This means that the distal edge is wider than the proximal edge, similar to the more posterior costals. The entire costal is convexly curved toward the posterior of the carapace, which is translated on the anterior and posterior edges of the costal. The proximal border is relatively straight except for the posteroproximal edge which is curved where it barely contacts neural III. On the dorsal surface, there are depressions present at the sulci for vertebral II and pleurals I and II. The sulcus between the pleurals I and II runs proximodistally and approximately parallels the anterior and posterior borders of the pleural. On the lateral border, the costal has a slight concave curvature where the pleurals’ sulcus crosses and a slight convex curvature and distal projection where the costal meets the suture between peripherals IV and V. On the visceral surface, there is a slight longitudinal ridge running its length, which signifies costal rib II, and a ventroproximal (or ventromedial) projection where the rib would contact the vertebra. The ridge is slightly more visible and prominent distally on the costal.

Costal III is similar in morphology to the surrounding costals, contacts neurals III and IV, costals II and IV, and peripherals V and VI, and is sub-rectangular with a wider distal edge. In ETMNH-8549, costal III has a maximum length of 74.56 mm, a proximal width of 27.00 mm, and a distal width of 30.50 mm, making it the largest costal, although costal V is wider distally. The costal is slightly convexly curved on both its anterior and posterior borders, making it appear somewhat pinched. The proximal border of costal III is similar to that of costals I and II, with it being nearly straight except for the angled posteroproximal border with a neural (neural IV in this case). The distal border is straight to slightly convexly curved. On the dorsal surface, there is no longer a sulcus separating two pleurals distally, but now a sulcus separating two vertebrals proximally (see vertebral discussion below). The sulci form a slight angle or “arrow” that points medially (or proximally) and the wrinkles are still present on the outer surface of costal III. The visceral surface is similar to that of costal II, with a faint ridge for the costal rib and a small ventroproximal projection for articulation with the vertebra. Costal III is in the middle of the shell, with the anterior costal (costal II) being curved convexly anterior, while the posterior costals (costals IV–VIII) are curved convexly posterior.

Costal IV is similar to costal II, although it is curved convexly posterior rather than anterior and contacts neurals IV and V, costals III and V, and peripherals VI and VII. In ETMNH-8549, costal IV has a maximum length of 74.97 mm, a proximal width of 22.37 mm, and a distal width of 23.98 mm. The proximal border, similar to the other costals, is straight except for the posteroproximal edge, which is angled where it articulates with neural V. The distal border also has a slight convex curve where it contacts the peripherals, although this can be two angled surfaces, depending on how the costal and peripherals articulate. Similar to costal II, there are depressions for the carapacial scutes and one (at the contact of pleurals II and III) runs roughly parallel to the anterior and posterior borders. The sulcus in costal IV, unlike that in costal II, does not remain roughly even between the anterior and posterior borders, but trends posteriorly toward the distal edge and nearly contacts the articular points between costals IV and V and peripherals VI and VII. In dorsal view, the proximal portion is still smooth under the vertebrals and the distal portion under the pleurals remains textured. Ventrally, the surface of costal IV is essentially the same as costals II and III.

Costal V has similarities to the preceding anterior costals and contacts neurals V and VI, costals IV and VI, and peripherals VII and VIII. In ETMNH-8549, costal V has a maximum length of 68.95 mm, a proximal width of 21.08 mm, and a distal width of 32.27 mm, making costal V the widest costal present distally (as stated above). The proximal border is the same as the preceding anterior ones, with a straight anteroproximal edge and an angled posteroproximal edge where it contacts neural VI. The anterior and posterior borders are both convexly curved posteriorly, similar to that in costal IV. The distal border is far wider than the proximal border, and comes to a distal point, or slight proximodistal projection, where the costal meets the suture between peripherals VII and VIII. On its dorsal surface, costal V is similar to costal III with a sulcus between two vertebrals (III and IV in this case) rather than between two pleurals. On the visceral surface of costal V, the ventroproximal projection for articulation with the vertebra is present, although the ridge for costal rib V is more prominent. The ridge expands ventrally toward the distal edge and continues onto peripheral VII and the anterior part of peripheral VIII, along with the hypoplastron to make up part of the inguinal buttress of the bridge. There is a slight depression anterior to the visceral ridge, which contacts the depression found in peripheral VII and helps form part of the bridge.

Costal VI is similar to the anterior costals, specifically costal IV, and contacts neurals VI and VII, costals V and VII, and peripherals VIII and IX. In ETMNH-8549, costal VI has a maximum length of 54.50 mm, a proximal width of 16.07 mm, and a distal width of 17.25 mm. Its proximal and distal edges are almost equal width and its anterior and posterior borders are convexly curved posteriorly, similar to a scaled-down version of costal IV. Its proximal and distal edges are also like that of costals II–V, although the distal projecting point is less apparent. Similar to costal V, it has the sulcus between two pleurals (III and IV in this case) running distally on its dorsal surface. The dorsal surface texture of costal VI is still smooth proximally and wrinkled distally. On the visceral surface, there is still a slight ridge marking the costal rib and a ventroproximal projection marking the articulation of the costal rib with the vertebra.

Costal VII is similar to costal VI, although it is smaller and conforms less to the geometric shape of the other costals while contacting neurals VII and VIII, costals VI and VIII, and peripherals IX and X. In ETMNH-8549, costal VII has a maximum length of 44.26 mm, a proximal width of 11.15 mm, and a distal width of 19.95 mm. Its proximal edge is the same as the preceding anterior costals with straight and angled portions, and both its anterior and posterior borders are convexly curved posteriorly. The curve is sharper on the posterior edge, which allows the distal border to be wider than the proximal one. The dorsal surface still has the same proximally smooth and distally wrinkled texture mentioned for the other costals. The wrinkles are less longitudinal though, and often make a type of semi-circle that convexly curves toward the posteroproximal portion of the carapace. The visceral surface still has the weak ridge for the costal rib, but now the ventroproximal projection for articulation between the rib and vertebra is faint and gracile.

Costal VIII is a small, sub-rectangular element that contacts neurals VIII and IX, costal VII, suprapygals I and II, and peripherals X and XI. In ETMNH-8549, costal VIII has a maximum length of 35.64 mm, a proximal width of 19.47 mm, and a distal width of 17.92 mm, making it the only costal with a wider proximal border than distal border. The antero- and posteroproximal borders angle to contact each other at a proximal point (“proximal-projecting arrowhead”). The anterior edge is convexly curved, while the posterior edge is concavely curved, meaning the anterior border is slightly longer than the posterior one. The distal edge has a slight distal point similar to, but opposite, that of the proximal border. There is a depression from the sulcus of vertebral V and pleural IV that runs from the proximal portion of the anterior border, concavely curves distally, and continues to the distal edge. The bone under the pleural is dorsally raised above that under the vertebral. The dorsal surface under the pleural on costal VIII is also covered in wrinkles that continue from costal VII, while the surface is relatively smooth under the vertebral. On the visceral surface, the low ridge for the costal rib is present, but the ventroproximal projection for articulation between the rib and the vertebra are now pronounced and take up the majority of the proximal portion of costal VIII on this surface.

### Sulci of the carapace

(Figs. 3 and 5)

The surface of the turtle’s carapace is covered with a number of keratinous scutes (or scales), of which the sulci (or seams) left behind can give an indication of their morphology and appearance. *T. haugrudi* has a single cervical scute, five vertebral scutes, eight pleural scutes (four pairs) and 24 marginal scutes (12 pairs).

**Cervical:** The cervical scute (=scale) is the smallest on the carapace, lying anteromedially and contacting marginals I and vertebral I, while lying completely on (and conforming to) the anteromedial portion of the nuchal. On ETMNH-8549, the cervical has a maximum length of 19.21 mm and a maximum width of 7.20 mm posteriorly, making it almost three times as long as wide (Table 1). Additionally, the length of the nuchal underlap on the visceral surface is 13.15 mm, making the underlap only 68% the length of the maximum dorsal length. The cervical is sub-triangular, coming to a point anteriorly when the entire anteromedial portion of the nuchal is present. The bone under the cervical is highly inflated and projects higher dorsally than even the anterior projections of the nuchal that lie lateral to it (under marginals I).

**Vertebrals:** The vertebral scutes lie medially on the dorsal surface of the carapace. There are a total of five, although the anterior (vertebral I) and posterior (vertebral V) vertebrals are quite different from the others morphologically. The bone under the vertebrals has a smooth surface texture, different from that laterally under the pleurals. The vertebrals would have made the median dorsal keel more apparent, especially on the far anterior and posterior portions of the carapace. Although there are depressions under the vertebrals lateral to the median keel, these may not have been apparent in the vertebrals. The vertebral sulci all have a slight sigmoidal curve anterior and posterior to where they meet the sulci between each set of pleurals. Vertebral I contacts the cervical, both marginals I, and both pleurals I. It lies on the nuchal, neural I, and both costals I. In ETMNH-8549, vertebral I has a maximum length of 38.50 mm, an anterior width of 16.31 mm, and a posterior (=maximum) width of 41.20 mm. The measurements for all the vertebrals are provided in Table 1. Vertebral I is somewhat “hourglass”- or “soda bottle”-shaped. It has a generalized sigmoidal curve on its lateral borders and is significantly thinner anteriorly. Its anterior and posterior borders are both convexly curved, making it appear slightly round. The bone under vertebral I, specifically that of the nuchal, is inflated and implies that a small median keel was present anteriorly on the carapace.

Vertebral II contacts vertebrals I and III and pleurals I and II, while lying on neurals I–III and costals I–III (both pairs). In ETMNH-8549, vertebral II has a maximum length of 47.19 mm and a maximum width of 55.54 mm. The maximum width is taken from where the two lateral projections of the lateral edges of the vertebral occur, which are present as the sulcus curves laterally to meet the sulcus between pleurals I and II. The anterior and posterior sulci are concave, giving the vertebral a slight “pinched-in” appearance, although the anterior border is curved farther than the posterior border.

Vertebral III contacts vertebrals II and IV and pleurals II and III, while lying on neurals III–V and costals III–V (both pairs). In ETMNH-8549, vertebral III has a maximum length of 51.16 mm and a maximum width of 59.05 mm, with the maximum width again taken from the two lateral projections of the vertebral, and making it both the longest and widest vertebral present. The anterior border of vertebral III, however, is convex while the posterior sulcus is concave. Compared to vertebral II, the posterior edge of vertebral III is more strongly curved as well.

Vertebral IV contacts vertebrals III and V and pleurals III and IV, while lying on neurals V–VIII and costals V–VIII (both pairs). In ETMNH-8549, vertebral IV has a maximum length of 44.62 mm and a maximum width of 53.50 mm, with the maximum width again taken from the two lateral projections of the vertebral, thus making it the shortest vertebral among vertebrals II–V. The anterior sulcus is convex, similar to vertebral III, with the posterior sulcus concave. The posterior edge is, again, more strongly curved with a deeply depressed sulcus.

Vertebral V contacts vertebral IV, pleurals IV, and marginals XI and XII, while lying on neural VIII, both suprapygals (I and II), the pygal, and peripherals X and XI (both pairs). In ETMNH-8549, vertebral V has a maximum length of 46.18 mm and a maximum width of 51.06 mm, making it the thinnest vertebral present among vertebrals II–V, while being sub-circular to sub-hexagonal. Its anterior sulcus is convexly curved where it contacts vertebral IV. The depression at the sulcus between vertebrals IV and V is commonly quite prominent (more prominent than those between the other vertebrals), and the bone under vertebral IV is often greatly raised dorsally over the bone under vertebral V. The posterior sulcus has a slight posterior projection where it meets the sulcus between the paired marginals XII. The other sulcus borders of vertebral V are generally angled, but still straight and not curved. The bone under vertebral V is smooth and lacks the wrinkles found under the pleurals.

**Pleurals:** The pleural scutes lie lateral to the medial vertebrals on the dorsal surface of the carapace. There are a total of eight, with four bilateral pairs. The bone under the pleurals is textured by distinct wrinkles longitudinally, which is distinct from the vertebrals that lie medial to them. The wrinkles become irregular posteriorly under each corresponding pleural, especially dorsomedially (or dorsoproximally). The pleural sulci all have a slight sigmoidal curve anterior and posterior to where they meet the sulci between each set of vertebrals. All measurements are from the left pleurals unless otherwise stated (Table 3). Pleural I contacts vertebrals I and II, pleural II, and marginals I–V, while lying on the nuchal, costals I and II, and peripherals I–IV. In ETMNH-8549, pleural I has a maximum proximodistal length of 64.31 mm (measured on posterior portion) and a maximum width of 59.35 mm (measured on proximal portion). The pleural is sub-triangular with a sub-rounded anterodistal edge, like a “slice of pizza.” There is an outwardly (or distally) projecting point whenever a carapacial scute sulcus contacts the sulcus around pleural I, meaning there is a point medially or proximally (between vertebrals I and II), an anteromedial projecting point (between vertebral I and marginal I) and four points along the anterolateral edge (between marginals I–V). The sulcus between pleural I and vertebral I also has a sigmoidal shape, congruent with the shape of vertebral I.

Pleural II contacts vertebrals II and III, pleurals I and III, and marginals V–VII, while lying on costals II–IV and peripherals IV–VII. In ETMNH-8549, pleural II has a maximum length of 75.10 mm, a medial or proximal (anteroposterior) width of 47.14 mm, and a lateral or distal (anteroposterior) width of 66.48 mm, making it the largest pleural present, while being sub-rectangular with its distal edge longer than its proximal edge. The proximal sulci curve out convexly between vertebrals II and III, but curve in concavely when they get nearer the anterior (I) and posterior (III) pleurals. The anterior and posterior sulci are concave, giving the pleural a slightly “pinched-in” appearance. The distal sulci have two distally projecting points between marginals V and VI and marginals VI and VII. A tiny posterodistal-most sliver of pleural II lies on peripheral VII.

Pleural III contacts vertebrals III and IV, pleurals II and IV, and marginals VII–IX, while lying on costals IV–VI and peripherals VII–IX. In ETMNH-8549, pleural III has a maximum length of 65.04 mm, a proximal width of 41.24 mm, and a distal width of 44.68 mm, while being sub-rectangular like pleural II. The proximal sulci curve out convexly between vertebrals III and IV, but curve in concavely when they get nearer the anterior (II) and posterior (IV) pleurals. The anterior sulcus is slightly convex, while the posterior sulcus is slightly concave, giving the entire pleural III a convex anterior curve. The distal sulci have two distally projecting points between marginals VII and VIII and marginals VIII and IX. The posterodistal-most point of the sulcus between pleurals III and IV lies in close proximity to the contact between peripherals VIII and IX.

Pleural IV contacts vertebrals IV and V, pleural III, and marginals IX–XI, while lying on costals VI–VIII and peripherals IX and X. In ETMNH-8549, pleural IV has a maximum length of 49.04 mm, a proximal width of 27.14 mm, and a distal width of 41.37 mm, making it the smallest pleural present, while being sub-rectangular to sub-trapezoidal, like a smaller version of pleural III. The proximal sulci curve out convexly between vertebrals IV and V, but curve in concavely when they are situated more anteriorly (near vertebral III) and along the distal sulcus of vertebral V. The anterior sulcus is slightly convex, while the posterior sulcus is slightly concave, giving the entire pleural IV a slight convex anterior curve. The distal sulci have a distally projecting point between marginals IX and X. The posterodistal-most portion of the sulcus anterior to vertebral V and in contact with marginal XI is convexly curved.

**Marginals:** The marginal scutes lie on the lateral edges of the carapace and cover most of the rim. There are a total of 24, with 12 bilateral pairs. The bone under the marginals is generally smooth, although in some instances there are concentric rings (on the bridge marginals), but these are only semi-circles, and all convexly curve anterodorsally. Under many of the marginals, especially anteriorly and posteriorly on the carapace, the bone is inflated, and this is often pronounced. The posterior marginals often have proximodistally (or mediolaterally)-running wrinkled ridges as well. The posterodistal portions of many of the anterior and posterior marginals are angled, which results in distal points and distinct notches between the marginals. There is often a smaller notch in the middle of the marginal between the two peripherals that the marginal lies on, but this would probably not be apparent in the marginal scute in life. All measurements are from the left marginals unless otherwise stated (Table 2). The shapes of the marginals will be considered from the dorsal surface, but one must be aware that the marginal scutes do wrap onto the visceral surface of the carapace and leave a general depression, or lip, which was discussed further above with the visceral surface of the carapacial sutures and elements. Marginal I contacts the cervical scute, vertebral I, pleural I, and marginal II, while lying on the nuchal and peripheral I. In ETMNH-8549, marginal I has a maximum (proximodistal) length of 26.90 mm and a maximum width (measured along posterior edge) of 20.32 mm, while being sub-triangular, as are most of the marginals that exhibit notching anteriorly (or distally) and/or posteriorly (or proximally). The medial and lateral sulci are both relatively straight where it contacts the cervical scute and marginal II. The proximal (or posterior) sulcus is slightly convex, and makes it appear slightly round where it contacts vertebral I and pleural I. The bone under marginal I is strongly inflated. There is only a small notch distally (or anteriorly) in the bone where the nuchal and peripheral I articulate. Additionally, the bone under marginal I, as is the case with the other marginals, sweeps (or curves) toward the back or posterior of the shell (laterally in the case of the anterior-most marginals).

Marginal II contacts pleural I and marginals I and III and lies on peripherals I and II. In ETMNH-8549, marginal II has a maximum length of 22.70 mm and a maximum width (measured along posterior edge) of 18.81 mm. The notching between marginals I and II is less prominent, so the contact is stronger than that of the cervical and marginal I and the widest point is more lateral on marginal II. It is sub-triangular, as, again, are most of the marginals that exhibit notching anteriorly and posteriorly. The medial and lateral sulci are both relatively straight where it contacts marginals I and III. The posterior sulcus is slightly convex, and makes it appear slightly round where it contacts pleural I. The bone under marginal II is strongly inflated. There is only a small notch anteriorly in the bone where peripherals I and II articulate. The bone under marginal II, as is the case with the other marginals, sweeps toward the posterior of the shell (laterally in the case of the anterior-most marginals).

Marginal III contacts pleural I and marginals II and IV and lies on peripherals II and III. In ETMNH-8549, marginal III has a maximum length of 18.11 mm and a maximum width (measured along posterior edge) of 24.03 mm. As is apparent from the measurements, the marginals are becoming shorter and more squat moving posteriorly toward the bridge. The notching anterior (or distal) on marginal III is less prominent than the notches of the anterior marginals, but is almost absent posterior (or caudal) to it. Marginal III is becoming more sub-rectangular, with a slightly rounded distal edge. The medial and lateral sulci are both relatively straight where it contacts marginals II and IV. The proximal sulcus is slightly convex, and makes it appear slightly round where it contacts pleural I. The bone under marginal III is still strongly inflated and there is only a small notch distally in the bone where peripherals II and III articulate. The bone under marginal III, as is the case with the other marginals, sweeps toward the posterior of the shell (laterally in the case of the anterior-most marginals), so that the largest portion of the rounded distal edge is lateral.

Marginal IV contacts pleural I and marginals III and V while lying on peripherals III and IV. In ETMNH-8549, marginal IV has a maximum length (proximal sulcus to distal lip) of 19.04 mm and a maximum width of 27.88 mm. As is still apparent from the measurements, the marginals are becoming shorter and more squat along the bridge. The notching around marginal IV is faint and the lip is only slightly more inflated posteriorly, still providing the general posterior sweeping of the marginals. Marginal IV is now completely sub-rectangular. The anterior and posterior sulci are both relatively straight where it contacts marginals III and V. The proximal sulcus is slightly convex, and makes it appear slightly round where it contacts pleural I. The bone under marginal IV is only slightly inflated.

Marginal V contacts pleurals I and II and marginals IV and VI while lying on peripherals IV and V. In ETMNH-8549, marginal V has a maximum length of 18.02 mm and a maximum width of 29.26 mm. Marginal V is still sub-rectangular, with little inflation of the lip distally. The notching around the marginal is also faint. The anterior and posterior sulci are both relatively straight where it contacts marginals IV and VI. Additionally, the proximal sulcus is slightly convex, and still appears slightly round where it contacts pleurals I and II. The bone under marginal V is only slightly inflated and there is a slight dorsoproximal projecting point where the sulcus of marginal V contacts the sulcus between pleurals I and II.

Marginal VI contacts pleural II and marginals V and VII and lies on peripherals V and VI. In ETMNH-8549, marginal VI has a maximum length of 18.40 mm and a maximum width of 31.95 mm. Marginal VI is also sub-rectangular, and has the least amount of inflation of any of the marginals. The anterior and posterior sulci are both quite straight where it contacts marginals V and VII. The proximal sulcus is still slightly convex, and appears slightly round where it contacts pleural II. The bone under marginal VI is only slightly inflated.

Marginal VII contacts pleurals II and III and marginals VI and VIII while lying on peripherals VI and VII. In ETMNH-8549, marginal VII has a maximum length of 23.12 mm and a maximum width of 31.68 mm. Marginal VII is still sub-rectangular, with little inflation of the lip distally, although the inflation is more pronounced posteriorly. Notching around marginal VII is also faint but, again, is more prominent posteriorly between marginals VII and VIII. The anterior and posterior sulci are still relatively straight, and the proximal sulcus is slightly convex, and still appears slightly round where it contacts pleurals II and III. The bone under marginal VII is more strongly inflated than marginals VI and there is a slight dorsoproximal projecting point where the sulcus of marginal VII contacts the sulcus between pleurals II and III. On marginals VII–X (and slightly with marginal XI) there is a general distodorsal sweep of the marginals, specifically with the posterior (=lateral) projections. This sweep makes the distal edges flatter anterior to the proximoposterior-most portion of the shell. Marginals XI do not display this characteristic as prominently. Marginals XII (as discussed below) sweep medioventrally, over the tail and backside of the animal.

Marginal VIII contacts pleural III and marginals VII and IX and lies on peripherals VII and VIII. In ETMNH-8549, marginal VIII has a maximum length of 29.10 mm and a maximum width of 28.23 mm. Marginal VIII is sub-triangular to sub-trapezoidal. While there is relatively little dorsal inflation of the bone under this marginal, the distal projections (equivalent to the distal lip of marginals IV–VII), have become more pronounced and exhibit a more prominent sweep toward the posterior of the carapace. Notching around marginal VIII is also becoming more pronounced, with the notch posterior to the marginal quite prominent. The distal notch between the two articulating peripherals (in this case VII and VIII) is more pronounced than on the anterior marginals. The anterior and posterior sulci are still relatively straight, and the proximal sulcus is slightly convex, and still appears slightly round where it contacts pleural III. The bone under marginal VIII is inflated, especially apparent posteriorly.

Marginal IX contacts pleurals III and IV and marginals VIII and X while lying on peripherals VIII and IX. In ETMNH-8549, marginal IX has a maximum length of 22.26 mm and a maximum width of 28.15 mm. Marginal IX is still sub-triangular with relatively little dorsal inflation of the bone underneath. The distal projections continue to become more pronounced and exhibit a more prominent sweep toward the posterior of the carapace. The notching around marginal IX is also becoming more pronounced, including the distal notch between peripherals VIII and IX. The anterolateral and posteromedial sulci are straight, and the anteroproximal sulcus is slightly convex, making it appear slightly round where it contacts pleurals III and IV.

Marginal X contacts pleural IV and marginals IX and XI, while lying on peripherals IX and X. In ETMNH-8549, marginal X has a maximum length of 21.60 mm and a maximum width of 26.15 mm. Marginal X is still sub-triangular with little dorsal inflation of the bone underneath. The distal projections are more strongly pronounced and exhibit a more prominent sweep toward the posterior of the carapace. The notching around marginal X is prominent, including the distal notch between peripherals VIII and IX. The distal sulcus is straight, while the proximal (including the sulcus with marginal XI) is slightly convex. The anterior sulcus where marginal X contacts pleural IV is still slightly convex, making it appear slightly rounded.

Marginal XI contacts pleural IV, vertebral V, and marginals X and XII and lies on peripherals X and XI. In ETMNH-8549, marginal XI has a maximum length of 21.96 mm and a maximum width of 30.42 mm. Marginal XI is sub-triangular to sub-trapezoidal with little dorsal inflation of the bone beneath it. The distal projections are more strongly pronounced, but exhibit less of a posterior sweep as marginal XI is already at the posterior edge of the carapace. The lateral sulcus is straight, while the medial border (including the sulcus with marginal XII) is slightly convex. The proximal sulcus where marginal XI contacts pleural IV and vertebral V is angled, creating a medially pointed arrow where the sulci meet.

Marginal XII (=supracaudal of some authors) contacts vertebral V and marginal XI (and the neighboring marginal XII from the opposite side of the carapace) while lying on peripheral XI and the pygal. In ETMNH-8549, marginal XII has a maximum length of 19.77 mm and a maximum width of 24.28 mm. Marginal XII is sub-triangular with some dorsal inflation of the bone beneath and is the posterior-most carapacial scute. The distal projections are strongly pronounced, but sweep medioventrally, unlike all other marginals. The notching around marginal XII is prominent. The lateral sulcus is angled slightly outward (or laterally), while the medial sulcus is straight (anteroposteriorly). The proximal sulcus is relatively straight as well, except for the anteromedial-most (or anteroproximal-most) portion, which is often curved where the marginals XII contact each other medially.

### Skull

(Figs. 6–8; Figs. S31–S33)

Several specimens have skull material, although the vast majority of this material is comprised of lower jaws or lower jaw fragments. Only three specimens have any skull (or cephalic) material, other than jaw material, preserved (ETMNH-3562, ETMNH-7690, and ETMNH-12457). ETMNH-3562 is discussed in detail with regards to individual skull elements as it is the most complete of these (Figs. 6–8). ETMNH-7690 (Fig. S31) is compared with ETMNH-3562 and ETMNH-12457 in parallel elements and discussed further in any elements it possesses that the others do not. ETMNH-3562 has some matrix adhering to the specimen and the skull is in two separate sections due to its fragility and fear of further preparation of the specimen causing additional damage or destruction. Based on reconstruction of the incomplete skull of ETMNH-3562, an anteroposterior (or rostrocaudal) length of at least 58.47 mm is calculated. The cranial elements that were identified with visible features are noted below as being from either ETMNH-3562, ETMNH-7690, or ETMNH-12457. The incomplete skull with ETMNH-3562 has at least portions of the premaxillae, maxillae, right jugal, quadratojugals, quadrates, squamosals, left prootic, left opisthotic, left exoccipital, prefrontals, frontals, postorbitals, parietals, supraoccipital, ?pterygoids, basisphenoid, basioccipital and hyoid bones. The skull fragment that is part of ETMNH-7690 represents a portion of the posterior of the skull and has parts of the quadrates, prootics, left opisthotic, left squamosal, pterygoids, basisphenoid, ?basioccipital and ?exoccipitals. ETMNH-12457 contains a nearly complete left maxilla (Fig. S32).

ETMNH-3562 has a complete set of lower jaws preserved as well (Figs. 6B–6D and 8B–8C). Unfortunately, the lower jaws are in place under the skull and upper jaws, so little can be said of their dorsal morphology. Several other specimens, including ETMNH-4686, ETMNH-11642, ETMNH-11643, ETMNH-12265, ETMNH-12457 (Figs. S33A–S33E), ETMNH-12577, ETMNH-12753 (Figs. S33F–S33I), and ETMNH-12832, have at least partial to nearly complete sets of lower jaws preserved.

**Premaxilla:** The premaxillae are essentially complete, with a height of 4.25 mm and a width of 2.55 mm each where they form the ventral surface of the apertura narium externa. There appears to be a slight dorsal indentation to the premaxillae, similar to modern *T. scripta*, although not as pronounced. The elements are both wider at their dorsal edges (compared to their ventral edges). There is a distinct ridge running medially on the dorsal surface of the premaxillae at their sutural contact. This ridge is lower than in modern *T. scripta*, resulting in less prominent depressions lateral in the apertura narium externa.

**Maxilla:** Both maxillae are nearly complete, with a maximum anteroposterior (or rostrocaudal) length of 15.94 mm from the premaxilla to the dorsal curve at the posterior of the maxilla. The surface texture of the bone reveals that a keratinized sheath would have been present, as is found in modern *T. scripta* and other living turtles. Rostrally (or anteriorly), the maxilla is relatively gracile, specifically where it reaches dorsally to contact the prefrontals. Due to the dorsal curvature of the maxilla, the orbits would have been relatively large in ETMNH-3562, including relatively larger than modern *T. scripta* specimens. Viscerally, the maxilla has a distinct depression, probably dealing with olfactory abilities. The caudal portion of the maxilla exhibits a dorsal convex curve that is far more gradual and less pronounced than in modern *T. scripta*, with it coming to a distinct point rather than a curve in the latter. The nearly complete left maxilla present in ETMNH-12457 (Fig. S32) agrees in morphology with that in ETMNH-3562.

**Jugal:** The jugal is present in ETMNH-3562, albeit incomplete. It contacts the maxilla and fossa orbitalis rostrally, the postorbital dorsally, and the quadratojugal caudally. Its contacts with the maxilla and quadratojugal are more apparent as it is broken dorsally, and the sutural contact with the postorbital is missing. The jugal does, however, exhibit a distinct ventral projection in the fossa condylaris. This ventral projection is not seen in modern *T. scripta*.

**Quadratojugal:** The quadratojugal in ETMNH-3562 is incomplete, but some information may still be gathered from it. The element would have been sub-triangular, while contacting the jugal rostrally and the quadrate caudally. A small ventral projection of the element wraps around the quadrate and makes up a portion of its ventral border rostral to the condylaris mandibularis.

**Quadrate:** The quadrates are heavily damaged in ETMNH-3562. A small, ventral portion of the cavum tympani is present on the left quadrate. The right quadrate is more deformed and little information can be derived from it. However, the condylaris mandibularis is complete. Ventrally, the condylaris mandibularis is sub-oval with a concave curve where it would contact the area articularis mandibularis of the lower jaw. Caudally, the condylaris mandibularis has three perpendicular sides that contact each other, with a “v-shaped” concave notch on the ventral side, giving it a “flattened pacman” morphology. Modern *T. scripta*, on the other hand, has a concave curve to its medial edge in this caudal (or posterior) view. Dorsally, the quadrate is partially preserved and forms part of the foramen stapedio temporale with the prootic. The dorsal surface of the quadrate is better preserved in ETMNH-7690 (Fig. S31). The medial notch on the quadrate in the fossa temporalis inferior is slightly more pronounced than that of modern *T. scripta*. The canal running ventral to this notch is quite prominent, more so than in modern *T. scripta*. The caudal half of the condylaris mandibularis is preserved in ETMNH-7690 as well, although it is no different than that in ETMNH-3562.

**Squamosal:** Incomplete squamosals are present in ETMNH-3562, but have been badly crushed and deformed. All that can be definitively said is that they projected caudal to the quadrate and do not disagree in morphology to any modern *T. scripta* skulls from the present evidence. A small, dorsal portion of the left squamosal is preserved in ETMNH-7690 (Fig. S31). It should be noted that the dorsal ridge of the squamosal sits rather low (ventrally), and appears to be far lower than the squamosal of modern *T. scripta* specimens.

**Prootic:** The prootic is a small element that is incomplete in ETMNH-3562. It lies medial to the quadrate, rostral to the opisthotic and lateral to the parietal. It makes up part of the foramen stapedio temporale with the quadrate. This foramen is larger in ETMNH-3562 than in modern *T. scripta* specimens. The prootics are preserved and more complete in ETMNH-7690 (Fig. S31). However, little else can be said about these sub-triangular elements, as they make up part of the foramen stapedio temporale with the quadrate in ETMNH-7690 as well. Their rostral edge is well rounded, and there is a strong sutural surface present for contact with the pterygoid and/or basisphenoid.

**Opisthotic:** The opisthotic lies medial to the squamosal, caudal to the prootic, and lateral to the parietal. It is incomplete in ETMNH-3562, although the smoother caudal edge that makes up part of the caudal edge of the skull is present. A disarticulated right opisthotic is present with the specimen. The recessus labyrinthicus is pronounced and easily visible on the element, along with the smaller canalis semicircularis horizontalis. The left opisthotic is also preserved in ETMNH-7690 (Fig. S31). It makes up a mediocaudal portion of the caudal “skull shelf” and the dorsal surface has a gentle concave curve to it, similar to ETMNH-3562.

**Exoccipital:** A small fragment of the right exoccipital is present on the bone fragment of the right opisthotic discussed above in ETMNH-3562. A tiny fragment of what is thought to be the left exoccipital of ETMNH-7690 is preserved as well, although nothing distinct from other *Trachemys* can be noted for either.

**Prefrontal:** The prefrontals of ETMNH-3562 are nearly complete and make up a majority of the dorsal portion of the fossa orbitalis. They are broken rostrally, but caudally they contact the frontals. Ventrally, the prefrontals form a ridge making up the dorsomedial portion of the fossa orbitalis, although the constriction between these two ridges is less pronounced than that in similarly sized modern *T. scripta*. This leads to a thinner (mediolaterally) prefrontal, and a thinner narial region and anterior of the skull. The element extends to where the prefrontal curves ventrally and makes up a portion of the aperture narium externa, where there is breakage and deformation to the element.

**Frontal:** The frontals in ETMNH-3562 are relatively large elements and, as is the case with modern *T. scripta*, reach the fossa orbitalis rostrolaterally (or anterolaterally). They contact the prefrontals rostrally, the postorbitals laterally and the parietals caudally. The frontals project rostrally at their medial contact. Sutural contacts are hard to recognize dorsally due to fusion. They appear to project farther laterally than would normally be expected for this type of turtle. Unlike the two posterior-lying elements, the visceral (=ventral) surface of the frontals is readily visible, with the pterygoid and other skull elements that would obscure this view absent. Unsurprisingly, the element is smooth viscerally, with two ridges running along its lateral edges making up parts of the processus inferior parietalis.

**Postorbital:** The postorbitals are damaged and incomplete in ETMNH-3562. They do, however, contact the parietals caudomedially and the frontals rostromedially. They are relatively thin laterally, resulting in the fossa orbitalis and fossa temporalis inferior lying closer together than in modern *T. scripta*. A small separate skull fragment with ETMNH-3562 may be the lateral portion of the right postorbital, although it has been deformed so its identification is questionable.

**Parietal:** The parietals are nearly complete in ETMNH-3562, although the caudal portion is missing or covered by matrix. They have a minimum rostrocaudal (or anteroposterior) length of 12.97 mm along the medial sutural surface. The parietal contacts the frontal rostrally, the postorbitals rostrolaterally and the supraoccipital caudally. It makes up part of the dorsomedial edge of the fossa temporalis inferior. It thins caudally as it contacts the supraoccipital. The sutural contact between the frontals and the parietals projects farther caudally at its medial point where all four sutural surfaces meet. This is similar to the condition found in modern *T. scripta*, although not as prominent. At the lateral edges of the parietal there is a prominent laterodorsal ridge which is also present in larger specimens of modern *T. scripta*. Visceral (=ventral) portions of the parietal are preserved as well, although the only major referable region is the dorsal portion of the processus inferior parietalis, which is relatively robust.

**Supraoccipital:** The supraoccipital and crista supraoccipitalis are both nearly complete in ETMNH-3562, although only portions of its dorsal surface and right lateral side are visible due to matrix adhering to the specimen. It measures a minimum of 18.10 mm along its dorsal surface, but the rostral-most portion is not visible due to matrix adhering to the specimen and breakage. The crista supraoccipitalis is at least 6.09 mm tall dorsoventrally, although the ventral portion is broken, so it would have been taller when complete. There is a distinct rostrocaudally running ridge midway along the crest in right lateral view. This ridge appears far longer and more distinct than similar ridges found in modern *T. scripta*. It is unknown if there was a flattened mediolateral surface of the crista supraoccipitalis due to breakage, but it is unlikely due to its absence in modern *Trachemys*. Also, similar to modern *T. scripta*, it appears the supraoccipital crest would have been quite thin along its dorsal surface. Additionally, the supraoccipital contacts the parietals rostrally.

**Palatine, Vomer, Pterygoid:** The palatines and vomer are not present in any of the specimens. While they may be partially present in ETMNH-3562, the cranial material is too fragmentary and deformed to be certain. Several slivers of what may be the pterygoid can be seen in dorsal view, but other than being relatively thin and gracile bones, nothing else can be noted. A fair portion, believed to be roughly the entire caudal half, of the right and left pterygoids is preserved with ETMNH-7690. Only the visceral (=dorsal) surface can be commented on as the ventral surface is covered in a hard matrix that is unable to be removed. Rostrally, the visceral surface has a sharp concave curve, causing the rostral portion to be far higher (dorsally) than the rest, resulting in a distinct depression. Within this depression are two smaller (approximately 1.00 mm diameter) depressions laterally (foramen palatinum posterius). Toward the caudal portion of the depression along the medial suture there is a distinct, small ridge (3.21 mm anteroposterior or rostrocaudal length). This ridge lies between the fenestrae ovalis. Additionally, the left cavum tympani is preserved, although it appears to be partially crushed.

**Basisphenoid:** The braincase, including the basioccipital and basisphenoid, is present in ETMNH-3562, although the latter is very fragmentary. Part of the braincase may be present in dorsal view, but, again, little can be noted of its features. Portions of the basisphenoid are preserved in ETMNH-7690 (Fig. S31). The suture between the pterygoids and the basisphenoid is hard to distinguish, but is believed to lie near the rostrocaudally directed ridge. A concave depression is still present viscerally, as it makes up part of the braincase.

**Basioccipital:** The basioccipital is present in ETMNH-3562, although it has undergone some deformation. The condylus occipitalis is incomplete, and the caudal portion is missing. Ventrally, the element is sub-triangular, similar to the condition in modern *T. scripta*. The ventral portion of the foramen magnum is present, although breakage prevents any further notes. A small portion of the basioccipital may be present with ETMNH-7690, although nothing of morphological value can be added.

**Hyoid bones:** At least one element present in ETMNH-3562 is interpreted as a hyoid bone. It is a relatively long and thin element with a gentle curve. Its ends are larger than the shaft, with one end particularly well rounded and sub-oval. It has a minimum length of 16.22 mm, although one end is incomplete. A second, possible hyoid bone is also present. This element is broken, but has a minimum length of 11.13 mm, and lies somewhat caudal to the lower jaw, where one would expect to find the hyoid bones.

**Dentary:** Both dentaries are preserved in ETMNH-3562, although their dorsal surfaces are not visible as they are in contact with the ventral surface of the upper jaws. Ventrally, there is a gentle curve near the articulation between the left and right dentaries. On the lateral and anterior (or rostral) surfaces of the dentaries, there are numerous small depressions and foramina, presumably signifying the presence of a keratinous beak. Posteriorly on the lateral surface there is a distinct and pronounced depression. Ventrally, it projects farther caudally before it contacts the prearticular and angular. Medially, there is a distinct longitudinal depression on the majority of each dentary known as the sulcus cartilaginous meckelii. Toward the caudal (or posterior) edge of the sulcus cartilaginous meckelii lies the foramen alveolare inferius and foramen intermandibularis medius, the latter of which sits at the anterior (or rostral) border of the prearticular. The left dentary is preserved with ETMNH-11643. It is nearly complete, although the anterior-most portion where it articulates with the left dentary is missing. The sulcus cartilaginous meckelii is still distinct, although without part of the prearticular covering it, it becomes dorsoventrally expanded caudally. The foramen alveolare inferius is also clearly visible without the prearticular present. Laterally, on the anterior rim of the lateral depression lies the foramen nervi auriculotemporalis which is oriented somewhat caudally. Dorsally, there is a longitudinal depression along the alveolar surface with a gently curved medial ridge that makes up part of the tomial ridge. The lateral ridge (i.e. tomial ridge), on the other hand, is quite sharp along its length. Midway along the tomial ridge lies a distinct dorsal projection, immediately followed caudally by a slight depression. This same projection is found in modern *T. scripta*. Anteriorly (or rostrally) the tomial ridge nears the labial ridge. Portions of both the left and right dentaries are preserved in ETMNH-11642, along with a small fragment believed to be the anterior-most (or rostral-most) part of one of the dentaries. While from a smaller individual and very incomplete, both dentaries agree morphologically with those of ETMNH-3562 and ETMNH-11643. The other dentaries present with other specimens, including those with ETMNH-4686, ETMNH-12265, ETMNH-12457 (Figs. S31A–S31E), ETMNH-12577, ETMNH-12753 (Figs. S31F–S31I), and ETMNH-12832 agree morphologically with those of ETMNH-3562.

**Coronoid:** While present, both coronoids in ETMNH-3562 are covered by portions of the jugals and quadratojugals, so little can be noted of their morphology, although they appear to make up the dorsal-most portion of the lateral depressions of the lower jaws.

**Surangular:** Both surangulars in ETMNH-3562 are covered by portions of the skull and not readily visible.

**Angular:** The angulars of ETMNH-3562 are not readily visible laterally except for a small portion caudally. Ventrally, however, the angulars project farther anteriorly (or rostrally) on the lower jaw, making up just over a third of its length. Medially, the angular projects far anteriorly (or rostrally), roughly half its length until it contacts the sulcus cartilaginous meckelii. It is well rounded ventrally, and makes up a portion of the foramen intermandibularis caudalis medially along with the prearticular. It is noted that the foramen intermandibularis caudalis are anteroposteriorly (or rostrocaudally) elongate and very pronounced, more so than in modern *T. scripta*.

**Prearticular:** The right and left prearticulars are preserved in ETMNH-3562, although the majority of these elements are not visible. As noted above, the prearticulars make up part of the border of the pronounced foramen intermandibularis caudalis with the angulars. They also make up part of the foramen intermandibularis medius with the caudal (or posterior) edge of the dentaries medially.

**Articular:** ETMNH-3562 has both the left and right articulars preserved, although they are mostly covered by matrix and the rest of the skull. In ventral and medial views, the articulars possess a concave curvature where they articulate with the quadrates.

### Postcranial skeleton

(Figs. 9–11; Figs. S34–S72)

Post-crania here represents all material that is not from the shell or skull. Post-crania are present in ETMNH-8549, along with several other confidently referred specimens. Based on the number of specimens that do possess more complete post-crania, an accurate description of the non-shell and non-cranial skeleton of this turtle can be derived. Any complete, or nearly complete post-crania are noted below, with comparisons to each other, although this means that not all post-crania, or specimens with post-crania were described, dependent on the completeness of the elements and specimens. Specimens with at least some post-crania include ETMNH-3558 (Figs. S52, S54 and S56–S58) ETMNH-3560, ETMNH-3562 (Figs. S51C–S51D, S53 and S55), ETMNH-4686, ETMNH-6936, ETMNH-8549 (Figs. 9A–9F and 11; Figs. S34–S39, S41–S48, S49A–S49D, S50, S59A, S59B, S60A–S60D, S61A, S61B, S63G–S63H and S64–S70), ETMNH-10390, ETMNH-10391, ETMNH-10547 (Fig. S71), ETMNH-11642 (Fig. S72), ETMNH-11643 (Figs. S51A and S51B), ETMNH-12265 (Fig. S62), ETMNH-12456, ETMNH-12457, ETMNH-12726, ETMNH-12753, ETMNH-12832 (Fig. 9G; Figs. S40, S49E, S49F, S59C, S59D, S60E, S60F, S61C, S61D and S63A–S63F), ETMNH-12833, ETMNH-12834, ETMNH-12979, ETMNH-13036, ETMNH 13443, ETMNH-14049, and ETMNH-14362.

### Axial skeleton

**Cervical vertebrae:** Vertebrae are known from several specimens, although almost all specimens that contain vertebrae only have fragments. Cervical vertebrae are known from several specimens, including ETMNH-8549 (Figs. 9A–9F; Figs. S34–S39), ETMNH-11643, ETMNH-12832 (Fig. 9G; Fig. S40), and ETMNH-14049. Those present include cervical vertebrae II (axis) through VIII. No cervical vertebra I (atlas) is preserved. Most of these bones are nearly complete and a description is provided for what is available. Similar to modern *T. scripta*, there is no definite presence or remnants of enlarged or pronounced cervical ribs, nor have any been recovered. While the atlas is not known, its morphology would have probably been very similar, if not identical, to those of modern *Trachemys*, and emydids in general.

The axis (=cervical vertebra II) in ETMNH-8549 is nearly complete and has a maximum length of 12.94 mm craniocaudally through the centrum (Fig. 9A; Fig. S34). The cranial surface of the centrum is flat to faintly convex and sub-triangular. Its dorsal surface has a low and craniocaudally short neural spine, although the neural spine is incomplete and broken caudally. On the left lateral side of cervical II, both the articular and lateral processes are complete. The articular process is sub-rectangular and angled cranioventrally. The lateral process is sub-triangular and projects slightly ventrally. These processes are incomplete on the right side. There are several pneumatic foramina on the vertebra, notably on the lateral edges of the neural spine toward the cranial portion of cervical II, and between the lateral processes and the hypapophysis. The ventral surface of the centrum is curved so that the caudal portion projects farther ventrally than the cranial portion, and the hypapophysis projects craniocaudally through the middle. The caudal surface of the centrum is concave (making it opisthocoelous) and circular to sub-circular, and its ventral portion projects farther caudally than its dorsal portion. There are two small “knobs” on the ventral edge of the caudal centrum surface where it articulates with cervical III. There appears to be no odontoid process cranially. ETMNH-8549 appears to have a second cervical II with the specimen. While incomplete, it appears to be of similar size to the aforementioned cervical II. However, its association with the rest of the specimen is questionable as it is a duplicate element and because its preservation is different from the other cervical vertebrae in ETMHN-8549. No example can be found of a turtle possessing two cervicals II or with two anterior cervical vertebrae of identical morphology, and it is thought that the vertebra was accidently placed with the remaining cervical vertebrae from another specimen. A third cervical vertebra II is present in ETMNH-11643. While incomplete, it agrees morphologically with the cervical II of ETMNH-8549.

Cervical vertebra III is nearly complete in ETMNH-8549 and has an axial length of 16.17 mm through the centrum (Fig. 9B; Fig. S35). It is opisthocoelous and dorsally the neural spine is low and elongate. The prezygapophyses are rounded cranially, sub-oval, and project cranially and somewhat dorsally. Only one of the postzygapophyses is complete (left), but is robust, sub-triangular in cross-section, projects somewhat dorsally, and slightly concave, with a depression dorsomedially. Ventrolateral to the prezygapophyses lie the lateral processes, which are more robust than those of cervical II and are more rounded on their lateral edges. There is a distinct ridge on the ventral surface of the lateral processes. The ventral surface of cervical III is curved so that the caudal portion projects farther ventrally than the cranial portion, similar to the condition in cervical II. The hypapophysis is nearly complete and does not fully reach either the cranial or caudal surfaces of the centrum. While slightly eroded, it appears the “knobs” on the caudal surface of the centrum in cervical II are also present in cervical III as well, although not as prominent. Several foramina are found on its surface, including on the ridge on the ventral surface of the lateral processes and on the sides of the hypapophysis. The cervical vertebra III present in ETMNH-14049, while partially covered in matrix, appears to agree morphologically with that in ETMNH-8549.

Cervical vertebra IV from ETMNH-8549 is more elongate than the anterior (more cranial) cervical vertebrae, with an axial length of 20.82 mm through the centrum, making it the longest cervical vertebra (Fig. 9C; Fig. S36). Since both its cranial and caudal centrum surfaces are convex, it is a derivation of an acoelous vertebra and marks the transition from opisthocoelous anteriorly (or cranially) to procoelous posteriorly (or caudally) in the cervical series. The vertebra is nearly complete, although a portion of its dorsal half is missing cranially, and the prezygapophysis and lateral process are present on the right side only. The prezygapophysis is similar to that of cervical III, with a rounded cranial edge and generally sub-oval shape. The lateral process has become less pronounced and more robust compared to that of cervical III. The ridges on the ventral surface of the lateral processes are becoming more pronounced and robust. The postzygapophysis is similar to that of cervical III, although it projects farther ventrally than that in cervical III. The depression on the dorsomedial surface of the postzygapophysis is even more pronounced than in cervical III. The neural canal is more flattened and sub-rectangular compared to being highly rounded cranially in the more anterior cervical vertebrae. The hypapophysis is nearly complete, and does not extend to the caudal surface of the centrum. The small “knobs” on the caudal surface of the centrum are more pronounced, but have shifted somewhat cranioventrally and are more lateral than in anterior cervicals. The ventral surface of the centrum displays almost none of the ventral curve caudally that the anterior cervicals exhibited. Several foramina are present, although the most conspicuous lie lateroventrally where the hypapophysis contacts the rest of the centrum. ETMNH-12832 has a cervical vertebra IV that measures 22.69 mm craniocaudally and agrees morphologically with that of ETMNH-8549. The cervical vertebra IV present in ETMNH-14049, while partially covered in matrix, appears to agree morphologically with those described above.

Cervical vertebra V is slightly shorter than cervical IV, with a maximum craniocaudal length of 19.89 mm through the centrum in ETMNH-8549 (Fig. 9D; Fig. S37). The cranial surface of the centrum has two concave surfaces (coronal to each other), while the caudal has two convex surfaces (also coronal to each other), making it the first procoelous vertebra in the series. While the cranial surface of the centrum is sub-circular to sub-oval, the caudal surface has two convex articular surfaces that are coronal to each other. The prezygapophyses are both intact and appear approximately the same as those of cervical IV. The lateral processes have receded farther and are now robust but small lateral projections lateral to the cranial surface of the centrum. The neural spine is not preserved in ETMNH-8549. Ventrally, the low and inconspicuous hypapophysis is present and again does not extend to the caudal edge. The “knobs” or ventrocaudal projections are still present, although also inconspicuous and present ventrolateral to the caudal centrum surface. The neural canal is now sub-rectangular and is wider than high. Several foramina are present, including a set caudomedial to the prezygapophyses and a set caudoventral to the lateral processes. ETMNH-12832 has a cervical vertebra V that measures 19.78 mm craniocaudally and agrees morphologically with that of ETMNH-8549. The cervical vertebra V present in ETMNH-14049, while partially covered in matrix, agrees morphologically with that in ETMNH-8549 as well.

Cervical vertebra VI is shorter than cervical V, with a maximum craniocaudal length of 17.15 mm through the centrum in ETMNH-8549 (Fig. 9E; Fig. S38). The cranial surface of the centrum has two concave coronal surfaces, while the caudal has two convex coronal surfaces, making it procoelous. Both cranial and caudal centrum surfaces are sub-oval. The right prezygapophysis is nearly complete and is robust where it begins to project craniodorsally. The lateral processes are almost completely gone, although small lateral bumps are still preserved signifying their presence. Most of the dorsal surface is missing. Ventrally, the bottom is relatively flat except for the hypapophysis, which is only present on the cranial-half of cervical VI. There is a slight depression present caudomedially on the ventral surface of the vertebra, and a pronounced demarcation between the two “condyles” on the caudal biconvex surface of the centrum. Two ridges lie lateral to the depression on the ventral surface. The entire vertebra curves dorsally toward its caudal end. The “knobs” have now become part of the two ventral ridges toward the posterior of the vertebra. The neural canal is still sub-rectangular, similar to the condition present in cervical V. Several foramina are present, including two pronounced foramina lying caudal to the prezygapophyses on the lateral sides of the vertebra and one on either lateral side of the centrum mid-length through it. ETMNH-12832 has a cervical vertebra VI that measures 17.70 mm craniocaudally and agrees morphologically with that of ETMNH-8549.

Cervical vertebra VII is shorter than cervical VI, with a maximum craniocaudal length of 12.96 mm through the centrum in ETMNH-8549 (Fig. 9F; Fig. S39). Both the cranial and caudal surfaces of the centrum have two concave surfaces coronal to each other, making the vertebra amphicoelous. Both ends are sub-oval, although the concavities are deeper and more pronounced on the cranial surface. The prezygapophyses are less prominent than on more anterior (or cranial) cervical vertebrae and are now inconspicuous dorsal “bumps” or projections. The lateral processes have been incorporated into the lateral portion of the cranial surface of the centrum. Dorsally, the neural spine has become split. There is a point cranially where the two halves meet, located just caudal to the prezygapophyses. The two halves then project caudally onto either one of the two postzygapophyses. They form a distinct “V”-shaped ridge that appears to point cranially. There is also a significant depression within the “V” and craniomedial to the postzygapophyses. The articular surface of the postzygapophyses lies flat (anterolaterally or craniolaterally) and points ventrally. Ventrally, the hypapophysis is highly reduced and lies only on the cranial half of the vertebra. The centrum is greatly constricted at its mid-length. There is no trace of the caudal “knobs” and resultant ridges present in the more anterior (or cranial) cervical vertebrae and, specifically, in cervical VI. The neural canal is now sub-triangular. Several faint foramina are present, although the foramina located just caudal to the prezygapophyses are, by far, the most prominent. ETMNH-12832 has a cervical vertebra VII that measures 13.43 mm craniocaudally and agrees morphologically with that of ETMNH-8549.

While no cervical vertebra VIII is preserved with ETMNH-8549, ETMNH-12832 does have one preserved. It is the only representation of this element for *T. haugrudi*, and is nearly complete (Fig. 9G; Fig. S40). The element has a maximum craniocaudal length of 10.21 mm. The cranial end of the centrum is partially eroded away, while the caudal end is convex and sub-circular. The prezygapophyses are prominent, and allow for strong articulation with cervical VII. The lateral processes are still cranial, and make up even more of the lateral portion of the anterior of the centrum. Dorsally, the neural spine still appears to be somewhat split, although most of it is not preserved, and is located more posterior (or caudal) on the vertebra. While the “V”-shaped ridge is present, it is not as prominent as on cervical vertebra VII. Cranial to the ridge, the dorsal portion of the neural arch is relatively flat, in contrast to cervical VII. The majority of the hypapophysis is broken and not preserved. In ventral view, the cranial portion of the vertebra is quite broad, and becomes constricted caudally. At the cranial and caudal ends, the neural canal is oval, while the majority of the canal itself is circular, which can be seen when viewed either cranially or caudally. At least one distinct foramen is visible on the left lateral side of the hypapophysis. Compared to the other cervical vertebrae, cervical VIII is distinctly short and robust. It articulates with dorsal vertebra I, the first vertebra fused to the carapace.

**Dorsal vertebrae:** Various other vertebrae are present with ETMNH-8549 and these include numerous fragments from dorsal vertebrae (Figs. 10A–10D; Figs. S41–S43). Almost all are incomplete and provide little useful information on apomorphies or distinct morphology. A few of them show a reduced, sub-triangular centrum with a sharp, distinct ridge running ventrally. Two small centra have a maximum length of 4.75 and 4.70 mm, respectively, while a third measures a mere 3.99 mm. Various dorsal vertebrae fragments are present with other specimens, including ETMNH-11462 and ETMNH-11643, with the latter lacking any remnants of caudal vertebrae and only having dorsal vertebrae fragments. These all agree morphologically with those preserved in ETMNH-8549.

**Caudal vertebrae:** The caudal section of the vertebral column of ETMNH-8549 is represented by at least thirteen vertebrae (Figs. 10E–10H; Figs. S44–S48). While the entire column is not present, these, undoubtedly, represent the majority of the tail. The cranial (or anterior) surfaces of the centra are slightly concave and generally sub-hexagonal to sub-circular on the anterior caudals, but these become more circular moving caudally and the posterior caudals are nearly perfectly round. The caudal surfaces of the centra are convex (making the caudal vertebrae procoelous) and remain circular from the anterior to the posterior caudals. The anterior caudals are the most prominently convex on their caudal surfaces (e.g., Figs. S44–S45), but becomes less so, and nearly flat, in more posterior caudals (e.g., Figs. S47–S48). The prezygapophyses project cranially, with the articular surface with the anterior vertebra facing ventrally. The prezygapophyses and the cranial portion of the neural spine or neural process prominently project cranially beyond the cranial surface of the centrum. The transverse processes are prominent on the anterior caudals and project almost to the caudal surface of the centrum. On the anterior-most caudal there are two prominent bumps where the transverse processes come into contact, or nearly into contact, with the cranial surface of the centrum (Fig. S44). These are far less prominent posterior to this caudal, and not present on the posterior caudals. The hypapophysis is relatively prominent on the anterior caudals, and absent on the posterior caudals. On the anterior caudals with a prominent hypapophysis, there are two distinct depressions between it and the transverse processes. The postzygapophyses are relatively indistinct, being similar to those of other modern emydids. They are short and sub-columnar, with their articular surfaces facing mediodorsally. Several foramina are present, with the most prominent ones present ventrally on the centrum and, commonly, on either side of the hypapophysis. The neural canal is sub-oval in the anterior caudals, being slightly wider than high. This is reversed in the posterior caudals, with the neural canal becoming higher than wide. The two processes of the neural spine that contact the centrum are both angled slightly lateral, in contrast with those of modern *T. scripta*, which are approximately parallel. A few caudal vertebrae or caudal vertebrae fragments are preserved with ETMNH-11643, and these all agree morphologically with those preserved with ETMNH-8549. ETMNH-3562 has at least nine caudal vertebrae preserved, although most of these are only fragmentary. A few are nearly complete, however, and agree with the morphology of those in ETMNH-8549, although they are larger and a bit more robust in the former. At least four caudal vertebrae are present in ETMNH-3558. These represent more posterior caudals, due to their more pronounced transverse processes, and agree with those posterior caudal vertebrae present in ETMNH-8549.

### Appendicular skeleton

**Scapula:** The left and right scapulae of ETMNH-8549 are preserved and both are nearly complete, with only the two distal ends missing (Figs. 11E and 11F; Figs. S49A–S49D). While both are nearly complete, the right scapula is better preserved, and will be the one described here (Figs. S49C and S49D). The scapula is similar to that of modern *T. scripta*, with an anterodorsal process, a ventromedial prong (=acromial process), and a central plate containing the glenoid fossa lying laterally. The two processes are separated by an angle of approximately 90°. Both the anterodorsal scapular process and the acromial process are broken with nearly equal lengths from the glenoid fossa. The anterodorsal process is more robust than the acromial process and is more robust than that of modern *T. scripta* with a maximum preserved length of 28.80 mm from the glenoid fossa and a maximum diameter of 5.85 mm near its distal end. The acromial process is slightly more robust than that of modern *T. scripta* as well, although the difference is not as significant, and has a maximum preserved length of 31.30 mm from the glenoid fossa and a maximum diameter of 5.36 mm near its proximal end. The left scapula has a more complete anterodorsal process with a length of at least 41.15 mm, but this, too, is incomplete (Figs. 11E and 11F; Figs. S62A and S62B). The central plate of the right scapula where the two processes meet is gently curved and more pronounced than in extant *T. scripta*. There is a distinct foramen on the caudal surface of the central plate. The glenoid fossa is shaped similar to a half-circle, although it is skewed and bent caudally. The glenoid fossa is more prominent than the articular surface with the coracoid, which is in the shape of a significantly smaller half-circle. ETMNH-3562 has a partial left scapula, with only the proximal end and glenoid cavity preserved, but agrees morphologically with the scapulae of ETMNH-8549.

**Coracoid:** Neither coracoid is present with ETMNH-8549. However, a nearly complete right coracoid is preserved with ETMNH-12832 (Figs. S49E and S49F). While a portion of the distal surface is broken, the portion that is preserved and gives a maximum preserved length of 34.89 mm. The width is 6.55 mm at the articular surface around the glenoid fossa, and approximately 14.85 mm at the distal end. The coracoid is flattened and flares more ventrolaterally than dorsomedially. Directly distal to the proximal end the shaft is thinnest, with a width of 4.70 mm. The articulation with the scapula and acromial process is a “half-circle” with an inflated ridge running through the middle, while the portion of the glenoid fossa from the coracoid is sub-triangular. In the middle of the shaft, as it flares out distally, there is a slight depression that can vary in depth. In ETMNH-12832 it is not prominent. An incomplete left coracoid is preserved with ETMNH-12456 that agrees with that of ETMNH-12832, although it is a bit shorter and more robust, with a more pronounced depression. ETMNH-12979 has a nearly complete right coracoid that agrees morphologically with that of ETMNH-12832 as well.

**Humerus:** ETMNH-8549 has a nearly complete right humerus with a maximum length of 43.67 mm and a mid-shaft width of 4.25 mm (Figs. 11A and 11B; Fig. S50). The proximal end of the humerus has the head, medial process (tuberculum externum of Bojanus (1819)) and lateral process (tuberculum internum of Bojanus (1819)). The head is sub-circular, with a slight constriction across its surface toward the lateral portion. As is normal with turtle humeri, the medial process is more pronounced than the lateral process. The medial process is also more rounded, while the lateral is slightly more pointed and sub-triangular. Lying distal to the head are several small foramina. A depression is present laterodorsal to the head and between the two processes. While not as pronounced as the depression on the femur, this depression is significant and contains a large number of foramina of various sizes, with the most prominent found distally where the two processes meet and the depression ends. The shaft is greatly bowed, more prominently than the bowing of the femur. The distal end is complete and has the external condylar tubercle. Its distal end is sub-oval and shows traces of articulation with both the radius and ulna. Near the distal end on the lateral surface lies a prominent and distinct groove that travels for approximately 6.75 mm until it reaches the distal surface of the humerus. A nearly complete right and fragmentary (only distal end) left humerus are present with ETMNH-3562 and both agree with the observations made of the right humerus in ETMNH-8549.

**Radius:** While no radii are preserved with ETMNH-8549, a complete left and nearly complete right radius are preserved with ETMNH-11643. Since the right agrees with the left morphologically, the left will be described here (Figs. S51A and S51B). The left radius has a maximum length of 20.60 mm, with diameters of 4.36 mm at its proximal end, 5.57 mm at its distal end, and 2.19 mm at mid shaft. The proximal surface is flattened and semi-circular to oval. Its medial edge is flattened at the proximal surface, and there is a depression present on the medial surface near the proximal end. The distal end is twisted compared to the proximal end, with the “oval” shape wider mediolaterally (versus dorsomedially to lateroventrally) at the proximal end. The shaft flares out near both the proximal and distal ends. The distal end is wider than the proximal end, but has a “P” shape, with the wide portion of the “P” situated medially. The lateral edge projects farther distally than the medial edge. There is a slim but distinct ridge that runs on the lateral surface from the distal edge up the shaft a short distance (~2–5 mm). There is also a slight depression on the shaft near the distal end on both the dorsal and ventral surfaces. While the dorsal surface is often rounded and the ventral surface flattened in modern *T. scripta*, this condition is not present in ETMNH-11643. ETMNH-3562 has a nearly complete right radius and fragments of the left radius preserved as well. The only portions missing from the right radius are parts of the distal-most end. It agrees morphologically with the radius preserved with ETMNH-11643, with the only noticeable difference being that the proximal surface of the radius is more rounded laterally in the former. The same somewhat flattened aspect of the radius discussed for ETMNH-11643 is present in ETMNH-3562 as well. A nearly complete right radius is present in ETMNH-3558 with a maximum length of 17.00 mm, making it slightly smaller and somewhat more gracile than the radius of ETMNH-11643, but otherwise agreeing with the latter specimen morphologically.

**Ulna:** Although no unlnae are preserved with ETMNH-8549, a complete right (Figs. S51C and S51D), and fragments of a left, ulna are present in ETMNH-3562. The element closely agrees in morphology to the ulnae present in modern *T. scripta*. The complete right ulna has a maximum length of 22.48 mm, with widths of 6.14 mm proximally and 6.55 mm distally. As is common in *Trachemys*, the proximal and distal ends flare out, with the medial shaft being more constricted and thinner. The entire element is “flattened” dorsoventrally, and there is a sharp ridge running along the external lateral edge (proximodistally). On the proximal surface, there is a distinct, more distally positioned internal articular area for the external condylar tuberosity of the humerus. This means the external proximal edge reaches farther proximally than the internal edge. The proximal surface is sub-triangular, with its “three points” directed laterally (external), medially (internal), and dorsally, giving a small ridge on the proximodorsal surface of the element. Distally, there is a slight concave depression on the laterodorsal surface. Similar to the proximal surface, the lateral (external) edge projects farther distally than the medial (internal) edge where the element articulates with several proximal carpals. A key difference between modern *Trachemys* and the ulna in *T. haugrudi* is the degree of curvature of the element. When holding the manus flat, the proximal end of the ulna is curved more medially, as seen with the angle of the ventrolateral surface near the proximal end. ETMNH-3558 has the distal end of the right ulna preserved and it agrees with the ulna of ETMNH-3562.

**Carpals:** ETMNH-3558 has at least three carpals preserved. These are identified as distal carpals, and are tentatively identified as I, IV and V (Fig. S52). Two elements in ETMNH-3562 are identified as distal carpals, potentially from digits II and III, and are known to come from the right manus of the specimen (Fig. S53). They are all small elements that are rounded and somewhat flattened proximodistally.

**Manual phalanges:** Eight phalanges are preserved with ETMNH-3558, all coming from the right manus. These all follow the normal phalangeal morphology in turtles, specifically in emydids. There were also several phalanges with ETMNH-3562, all known to also be from the right manus. Six could be identified as either proximal or distal phalanges, although further distinction was not conducted. A single phalanx is identified as a proximal manual phalanx though, based on size of the element itself and size of the proximal end (Fig. S54). These agree in morphology with those of ETMNH-3558 except for being slightly larger, with the latter specimen (ETMNH-3558) known to be a smaller individual.

**Manual unguals:** A single manual ungual (=terminal phalanx) is present in ETMNH-3562, collected with the majority of the right forelimb (Fig. S55). The element is small and incomplete, with a maximum proximodistal length of 4.75 mm and a maximum proximal diameter of 1.74 mm. There is also some tuberosity around the edges of the proximal surface. Otherwise, it agrees well with the pedal unguals discussed below. ETMNH-3558 has three unguals from the right manus preserved (Figs. S56–S58). These all have maximum lengths of around 5 mm, with maximum proximal diameters of around 2 mm, and agree with the manual ungual in ETMNH-3562. It is also apparent that the ventral edge of the proximal surface is often concave.

**Ilium:** The bones of the pelvis are all nearly complete in ETMNH-8549, and those described here are from the left side. The ilium is the largest pelvic element, with a maximum length of 26.48 mm dorsoventrally (Figs. 11C and 11D; Figs. S59A and S59B). There are three main articular surfaces proximally; those for the ischium, pubis, and the femoral head (=acetabulum). All articular surfaces are sub-triangular, with the acetabulum being slightly concave. The ilium is wider in a somewhat mediolateral-plane proximally, but this twists and the element becomes wider in a more parasagittal plane distally. It flares out toward the distal end, with the lateral surface flattened, while the medial surface is more rounded. There is a significant medial depression on the medial surface at the caudal end where the ilium contacts the sacrum. It is difficult to determine whether only one sacral vertebra contacts the ilium, or two do as in modern *T. scripta*. A left ilium is nearly complete in ETMNH-11643 and agrees morphologically with that of ETMNH-8549. A complete left ilium is present in ETMNH-12832, which shows the extent to which the anterodorsal portion of the ilium tends to flare out (Figs. S59C and S59D).

**Ischium:** The left ischium is nearly complete in ETMNH-8549, with a proximodistal length of 19.73 mm (Figs. S60C and S60D), while an incomplete right ischium is also present (Figs. S60A and S60B). Proximally there are three surfaces of contact; one with the ilium, one with the ischium, and one with the femoral head (=acetabulum). The ischium has its strongest articulation with the ilium, with only a small contact with the pubis. The proximoposterior edge of the ischium also exhibits a gentle curve, as for the proximoanterior edge of the pubis, again more gracile than that in modern *T. scripta*. There is a relatively strong projection caudal to the curve where the ischium would contact, or nearly contact, the plastron. The projection is relatively wide and pronounced, more so than in modern *T. scripta*. Medial to this projection is another gentle concave edge, although the medial (or distal) edge where the two ischia would meet is not preserved. Partial left and right ischia are present with ETMNH-11643 and both agree morphologically with that of ETMNH-8549. An incomplete right ischium is present in ETMNH-3558. Other than being slightly smaller than the same element in ETMNH-8549, the only remaining difference is the shape and morphology of the articular surfaces, although this may be due to breakage along the proximoanterior surface. A nearly complete left ischium is present in ETMNH-12832 (Figs. S60E and S60F), and agrees closely with the morphology of ETMNH-8549.

**Pubis:** The left pubis is nearly complete and is smaller and more gracile than the ilium and ischium, with a proximodistal length of 16.95 mm in ETMNH-8549 (Figs. S61A and S61B). Proximally there are three surfaces of contact on the pubis; including that with the ilium, the ischium, and the femoral head (=acetabulum). While all three are sub-triangular, the contact with the ilium is, by far, the most prominent. Similar to that of other turtles, there is a gentle concave curve to the proximoanterior edge of the pubis until a projection where it contacts, or nearly contacts, the plastron. This concave curve appears to be more open and gentle than that in modern *T. scripta*, and was present in another specimen with pelvic elements (ETMNH-11643). There is another gentle concave curve medial to this contacting projection where it would contact the other pubis, although the element is incomplete and this portion is not preserved. The entire element is gracile. The left and right pubes, although not complete, are present in ETMNH-11643 and the left pubis is present in ETMNH-12832 (Figs. S61C and S61D), all of which agree morphologically with those in ETMNH-8549.

**Femur:** A nearly complete left femur is present in ETMNH-8549 with a preserved length of 38.10 mm, although it is missing the distal portion with the condyles. The element is quite gracile, with a midshaft width of 3.52 mm, and is concave medially. The femoral head is pronounced and sub-oval, with the two farthest projections pointing anteroventrally and posterodorsally. Both the trochanter minor and trochanter major are broken and incomplete. Even so, it can be deduced that neither trochanter flared out significantly from the middle of the shaft. Laterodistal to the head and between the trochanter minor and trochanter major lies a pronounced depression. This depression is sub-circular and projects slightly into the shaft distally. Distally, just proximal to the point of breakage, there is a significant caudal projection. The left femur that is part of ETMNH-3560 agrees in morphology to that of ETMNH-8549, even though it is from a smaller and, presumably, younger individual. Fragments of the left and right femora were recovered with ETMNH-3562, and these agree morphologically with the femur in ETMNH-8549. ETMNH-12265 has a complete right, and nearly complete left, femur. The complete right femur has a length of 44.30 mm and a midshaft width of 3.95 mm (Fig. S62) and agrees morphologically with those described above.

**Tibia:** Portions of both tibiae are preserved in ETMNH-8549, although this only includes the proximal and distal ends of the right tibia, and the proximal end of the left tibia. The proximal ends are sub-triangular, with a slight depression in the center. The proximal end tapers quickly into the thin, gracile shaft of the tibia. The majority of the shafts of both the left and right tibiae are missing. The distal end of the right tibia is similar to that of other *Trachemys* and is sub-trapezoidal to sub-triangular. On its distal surface, there is a small projection ventrolaterally, similar to that in modern *T. scripta*. There is also a slim, but distinct, ridge traversing the dorsal portion of the shaft that is present. The shaft of the tibia appears to be more robust distally than proximally. ETMNH-3560 has the distal part of the right tibia preserved with most of the shaft. It agrees with the right tibia in ETMNH-8549, although the distal surface is slightly more rounded and the distal surface itself is slightly convex. An incomplete left tibia is preserved with ETMNH-11643 with the proximal end and most of the shaft preserved. The proximal end is robust, with a prominent ridge lateroventrally. There is a distinct depression on the lateral surface near the proximal end. The proximal surface is somewhat rounded, although it is flattened toward the lateral edge, and is convex. The shaft is slightly bent and is sub-triangular in cross-section. A nearly complete left tibia is preserved with ETMNH-12832 (Figs. S63A–S63F) that agrees morphologically with those discussed above, and represents the only nearly complete tibia of *T. haugrudi*. It has a length of 30.44 mm with widths of 7.40 mm (proximally), 4.37 mm (midshaft), and 6.18 mm (distally).

**Fibula:** The left fibula is preserved in ETMNH-8549 and has a maximum length of 27.17 mm (Figs. 11G and 11H; Figs. S63G and S63H). It is a thin, gracile element similar to that of modern *T. scripta*, with widths of 3.00 mm at its proximal end and 5.60 mm at its distal end. Proximally, the element is round, thin and inconspicuous. However, it begins to flare toward the distal end. The fibula has a stronger curve medially, giving it a slight “boot” or “pubis”-like shape. Distally, the surface is sub-oval. There is a sharp ridge that runs laterally along its length before reaching the distal surface, where it becomes more pronounced. This contrasts with the medial portion near the distal surface, which is more flattened. The distal end also flares dorsally for muscle attachment. The distal end of a left fibula is preserved in ETMNH-11643 as well. The shaft is flattened and flares somewhat with a gentle curve, although it is more robust medially versus laterally, with a distinct ridge running along the lateral surface. Distally, the element is robust, sub-trapezoidal, and is convex. The distal surface is better preserved than that in ETMNH-8549, which may explain why they differ slightly in morphology. A nearly complete right fibula in ETMNH-3558, missing only the proximal end, and a left fibula in ETMNH-12832 both agree morphologically with those described above.

**Metatarsals:** The right metatarsals of digits ?II, III, and V are present in ETMNH-8549. Right metatarsal ?II is nearly complete, and has a steep proximodistal curve dorsally (Fig. S64). The right metatarsal III is a more distinct element, with a maximum length of 10.43 mm (Fig. S65). It has articular surfaces for metatarsal II and the proximal phalanx of digit II. It is more robust proximally versus distally. It thins out quickly from its proximal surface and becomes more blade-like distally with a gentle concave ventral curve. The articulation of the distal surface with the next more distal phalanx of digit III is only slightly more robust than the rest of the distal surface. Due to the curvature of the element, the majority of the dorsal surface has a general depression. Metatarsal V has articular surfaces for distal tarsal IV, metatarsal IV, and the proximal phalanx of digit V (Figs. 11I and 11J; Figs. S66A and S66B).

**Astragalus, calcaneum, other tarsals:** Several pedal elements are present in ETMNH-8549, including the left astragalus (Figs. S66C and S66D). While in some turtles the astragalus and calcaneum tend to fuse, the calcaneum is not preserved with the astragalus in ETMNH-8549. Dorsally, the astragalus is a sub-rounded element, with a small rim running distally in a semi-circle at its edge. There is a small bump proximomedially on its dorsal surface as well, approximately where the tibia articulates with the astragalus. Ventrally, the element is concave, with a foramen close to its center. Various articular surfaces are present around its lateral, proximal, and distal sides, including one for the fourth distal tarsal. An incomplete distal tarsal, tentatively identified as from digit IV, is identified as well (Figs. S66E and S66F). Three small, semi-circular elements are present in ETMNH-11642, and these are tentatively referred to as distal tarsals II, III and IV, respectively. A right astragalus and right distal tarsal are present with ETMNH-3562, along with a complete left astragalus and metatarsal V, which all agree with the observations in ETMNH-8549. Several pedal elements are present in ETMNH-3558. These include the astragalus, metatarsal V, and three distal tarsals, all of which agree morphologically with those discussed above.

**Pedal phalanges:** Other pedal elements are present in ETMNH-8549, although most are gracile and are considered medial and proximal pedal phalanges (Figs. S67–S69). These include a proximal phalanx that is nearly complete and sub-circular, with a maximum length of 11.03 mm (Fig. S67). Little can be said of its morphology, although both the dorsal and ventral surfaces of the element have a depression in their center. A somewhat flattened and semi-circular element is believed to represent the proximal phalanx of digit V. Its dorsal surface is concave, and its lateral edge is convex. There is a large articular surface for distal tarsal III. A key note of difference between this element and that in modern *T. scripta* is that digit V is situated more laterally based on the orientation of the articular surface for it on metatarsal V. Distal pedal phalanges are present in ETMNH-8549 as well, and all are short and relatively robust (Fig. S70). Two pedal phalanges are present in ETMNH-11462, although they are smaller and incomplete compared to those in ETMNH-8549, with measurements of approximately 5 mm each. Both elements have the same curvature of the proximal versus distal ends that is found in other proximal phalanges of *Trachemys* specimens. Six other complete to nearly complete phalanges are part of ETMNH-11642. These are all small (approximately 5 mm length), somewhat robust, especially compared to those in ETMNH-8549, and cannot be attributed confidently to the manus or pes. Several phalanges are present with ETMNH-3562 from both the left and right hindlimbs. These all agree morphologically with the phalanges in ETMNH-11462, implying that longer phalanges are present in the pes compared to the manus. Several pedal phalanges are present in ETMNH-3558 and all agree morphologically with those discussed above.

**Pedal unguals:** A single pedal ungual (=terminal phalanx) is present in ETMNH-11642 (Fig. S72). It is considered a pedal ungual because the other elements with the specimen were from the hind-, and not the fore-, foot. The ungual has a length of 5.43 mm and a diameter of 2.00 mm at its proximal end. The dorsal surface is well rounded, while the ventral surface is flattened. This same morphology is present on the flat proximal surface, giving it a “half-circle” appearance. There is a distinct constriction immediately distal to the proximal end, and the element tapers distally to a point, common in turtle unguals. ETMNH-10547 contains a single pedal ungual as well (Fig. S71). It is larger than the ungual in ETMNH-11642, with a length of 7.25 mm and a proximal diameter of 2.38 mm. There are two clear grooves on the lateral sides of this ungual though, and both travel proximally from the distal point, and gently curve toward the ventral surface. The groove connects with the ventral surface 5.35 mm from the distal point. The entire claw is gently curved, with the distal end projecting farther ventrally than the proximal end. The proximal surface agrees with that of the ungual in ETMNH-11642, although it is slightly concave and there is a ridge running medially through it from the dorsal toward the ventral surfaces in the former. The same constriction from ETMNH-11642 near the proximal surface is present in ETMNH-10547. ETMNH-11643 has a single ungual preserved with a maximum length of 8.26 mm and a maximum proximal diameter of 2.86 mm. It agrees completely in morphology with the pedal ungual of ETMNH-10547. ETMNH-3562 has a single right pedal ungual with a length of 5.87 mm and a proximal diameter of 2.70 mm. It also has three left pedal unguals, with lengths of 8.45, 8.70, and 7.60 mm and proximal diameters of 2.94, 2.83, and 2.91 mm, respectively. All pedal unguals in ETMNH-3562 agree with the other pedal unguals discussed above. At least three pedal unguals are present with ETMNH-3558, all, presumably, from the right pes. Only one is complete, but all also agree morphologically with the pedal unguals discussed above.

### Phylogenetic analysis

The phylogenetic relationships of *T. haugrudi* are tested by integrating it into a list of currently accepted extant deirochelyine taxa, along with several other suspected deirochelyine fossil representatives, using morphologic evidence. The analysis pools characters from several previous studies, including those of Bojanus (1819), Hay (1908), White (1929), Galbreath (1948), Tinkle (1962), McDowell (1964), Weaver & Robertson (1967), Weaver & Rose (1967), Adler (1968), Parsons (1968), Moll & Legler (1971), Zug (1971), Ernst & Barbour (1972, 1989), Waagen (1972), Bramble (1974), Winokur & Legler (1975), Jackson (1977, 1978, 1988), Killebrew (1979), Pritchard (1979), Dobie (1981), Bertl & Killebrew (1983), Seidel & Inchaustegui Miranda (1984), Ward (1984), Seidel & Smith (1986), Gaffney & Meylan (1988), Seidel (1988, 1994, 2002), Ernst (1990), Gibbons & Lovich (1990), Legler (1990), Seidel & Jackson (1990), Seidel & Palmer (1991), Burke, Leuteritz & Wolf (1996), Minx (1996), Seidel, Stuart & Degenhardt (1999), Ultsch et al. (2001), Stephens & Wiens (2003), Bonin, Devaux & Dupré (2006), Joyce (2007), Buhlmann, Tuberville & Gibbons (2008), Ernst & Lovich (2009), McCord et al. (2010), Sterli & de la Fuente (2011), and Jasinski (2013a). Additionally, there are 25 new characters not listed in other studies. Character scores were confirmed from previous studies, and changed or newly scored where appropriate. All specimens examined first hand are listed in Appendix 1, while all characters and character states are listed in Appendix 2. Changes to pre-existing characters and character states are also listed with Appendix 2. Finally, the taxon-character state matrix is provided in Appendix 3. *Clemmys guttata*, a member of the Emydinae, sister subfamily to the Deirochelyinae in the Emydidae, was designated as the outgroup taxon.

Species of *Trachemys* were the concentration of this analysis, as *T. haugrudi* was identified as a member of the genus by features discussed by Seidel & Jackson (1990). Current taxonomic identifications, namely species and subspecies, are taken from a combination of studies, including; Fritz et al. (2012), McCranie et al. (2013), Parham et al. (2013, 2015), and Vargas-Ramírez et al. (2017). Most modern species of *Trachemys* were included in the analysis to determine how the fossil species believed to belong to *Trachemys* were arranged. *Trachemys decussata* was excluded due to a lack of data and scorable specimens. *Trachemys taylori* was excluded as it has been found in molecular studies to be only a shallow variant of *Trachemys venusta* (see Parham et al., 2013). *Trachemys medemi* was not included as scorable specimens were not yet available to the author. The most complete fossil *Trachemys* species were included in the analysis, including *T. hillii*, *T. idahoensis*, *T. inflata*, and *T. platymarginata. T. inflata* was included as it was believed to be the most closely related species to *T. haugrudi* (see Parmalee et al., 2002). Several Pleistocene species of *Trachemys* were not included in the phylogenetic analysis because many authors consider all Pleistocene *Trachemys* to represent fossil *T. scripta* (Preston, 1966, 1971; Weaver & Robertson, 1967; Jackson, 1988; Seidel & Jackson, 1990), although this may be incorrect and these species are currently under further study. Several other fossil deirochelyine species were included to see their position on the tree and what evolutionary lineage they belonged to. Finally, all modern taxa are identified to subspecies. This is for the most accurate definition of characters within distinct taxa, and because different subspecies may score out differently. For *Trachemys*, only one subspecies was used per species, providing each species one representative. Much of the information on the accepted species was taken from several recent molecular studies, including Spinks et al. (2009, 2016), Fritz et al. (2012), McCranie et al. (2013), Parham et al. (2013, 2015), and Vargas-Ramírez et al. (2017). Recent studies by Parham et al. (2013, 2015) have further investigated the molecular relationships within *Trachemys*, particularly of Caribbean *Trachemys* and *Trachemys ornata*, respectively. Some relationships from Parham et al. (2013) were different from those presented by Fritz et al. (2012), such as the species status of *Trachemys emolli* (rather than as *Trachemys grayi emolli* in the latter). Parham et al. (2015) also disagreed with Fritz et al. (2012) on the species status of *T. venusta* (rather than as a subspecies of *T. ornata* in the latter). Spinks et al. (2009, 2016) included a few species of *Trachemys*, including *T. scripta* and *Trachemys stejnegeri* (Spinks et al., 2009 also included *T. taylori*), as their studies were focused more on intergeneric relationships with only a few species from each genus present. Several studies on molecular phylogenetics have included *Trachemys* species, including Stephens & Wiens (2003), Jackson et al. (2008), Spinks et al. (2009, 2016), Wiens, Kuczynski & Stephens (2010), Fritz et al. (2012), McCranie et al. (2013), Parham et al. (2013, 2015), and Vargas-Ramírez et al. (2017). Their resulting molecular relationships are compared to the morphological relationships found in the present study. Phylogentic analyses were run with and without molecular contraints. Constraints were based off the recent intergeneric relationships recovered by Spinks et al. (2016, fig. 5), namely *Deirochelys* + (*Chrysemys* + (*Pseudemys* + ((*Graptyemys* + *Malaclemys*) + *Trachemys*))). Species were not constrained. This was done to constrain relationships between genera and allow morphologic data to determine the interspecific relationships within genera and to determine the generic placement of the included fossil taxa.

**Figure 12:**
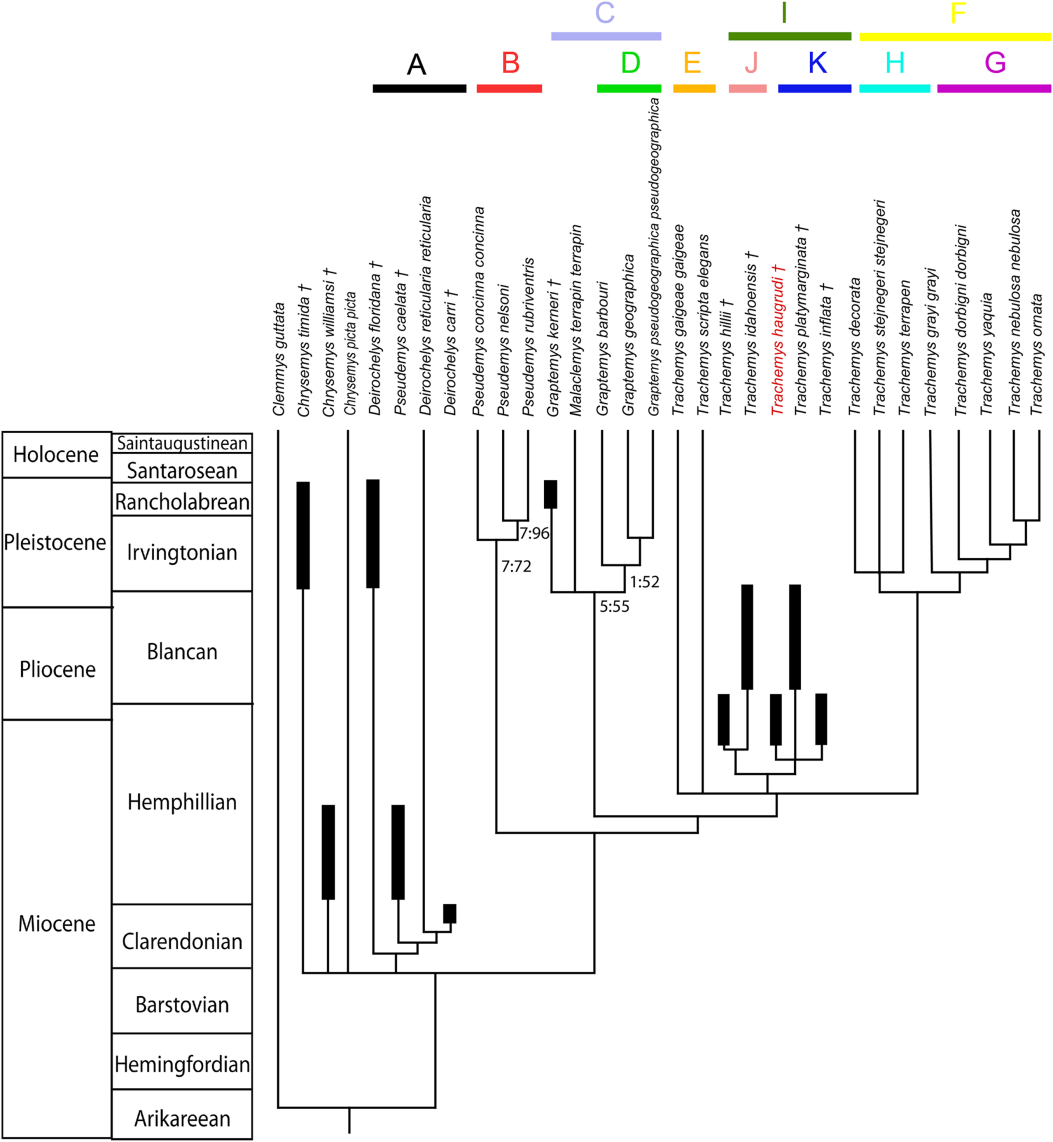
Phylogenetic relationships of deirochelyine emydids supported by this study based on morphologic data constrained by a molecular backbone. See text and appendices for details. Intergeneric relationships of modern taxa constrained based on molecular phylogenetic analysis presented by Spinks et al. (2016, fig. 5). Analysis includes the emydine *Clemmys guttata* as the outgroup. The tree presented represents the strict consensus tree of deirochelyine relationships found in the phylogenetic analysis of 243 characters within 31 taxa. Clades listed are called out and discussed in the text. Thickened bars indicate age ranges for fossil taxa. Tree length equals 882 steps, consistency index equals 0.409, retention index equals 0.475. Numbers beside branches provide the bremer supports (first) and the bootstrap values (second). Numbers are shown for branches with bootstrap values greater than 50. A, Clade “A”; B, Clade “B”; C, Clade “C”; D, Clade “D”; E, Clade “E”; F, Clade “F”; G, Clade “G”; H, Clade “H”; I, Clade “I”; J, Clade “J”; K, Clade “K.”

A chi-squared test was run using JMP 11 (Sall, Creighton & Lehman, 2005) to determine whether any characters were being duplicated. Original maximum parsimony analyses were run using PAUP 4.0b10 (Swofford, 2002), while later analyses with molecular contraints were run using TNT v1.5 (Goloboff & Catalano, 2016). There were a total of 31 taxa analyzed, including 30 deirochelyines (ingroup taxa). A total of 243 characters were scored, with 96 cranial (40% of total), 23 post-cranial (9%), 82 directly from the osteology of the shell (34%), and 42 from the scutes of the shell (17%). All characters were left unweighted, a branch-and-bound search was used with minimum branch lengths set to collapse. When run without constraints, 29 most parsimonious trees were recovered with a consistency index (CI) of 0.413, a retention index (RI) of 0.483, and a tree length of 797 steps. When run with molecular constraints on the modern genera (not on any fossil species), 21 trees were recovered with a CI of 0.409, a retention index of 0.475, and a tree length of 882 steps, and a strict consensus tree was derived (Fig. 12). The data was also run through a 50% majority rule consensus tree both with and without constraints (see Supplemental Information), which provided similar clades and relationships as those of the strict consensus phylogeny, albeit with less resolution in some areas (e.g., Caribbean *Trachemys*, southeastern fossil *Trachemys*, *Chrysemys*). Clades discussed in the text are equivalent between the phylogenetic analyses. Hypotheses of the phylogenetic relationships are discussed based on findings in the present study and then comparing these to previous studies.

## Discussion

As discussed above for the diagnosis, *T. haugrudi* was identified as a member of the genus *Trachemys* by features discussed by Seidel & Jackson (1990). For this reason, *Trachemys* was the focus of the analysis, with several modern members of the other deirochelyine genera included, along with several presumed fossil members of the subfamily. This was done to analyze the relationships among the species of *Trachemys*, to analyze the morphological relationships among the deirochelyine genera, and to assess the placement of several other fossil deirochelyines previously assigned to modern genera.

**Phylogenetic relationships among *Trachemys*:** Relationships within *Trachemys* have been discussed previously (Jackson, 1988; Seidel & Jackson, 1990), but little has been done to analyze these relationships morphologically, although a few studies have been conducted (Seidel, 2002; Stephens & Wiens, 2003). These latter studies have worked with modern members of the genus, but none of the fossil representatives. A single potential fossil representative (*T. idahoensis*) was analyzed by Joyce et al. (2013), who determined it was more accurately considered a stem *Graptemys* (although its relationships are discussed further below). An unpublished thesis investigated the relationships of fossil *Trachemys* (Jasinski, 2013a), with relevant data from that study used in the the present study.

*Trachemys* species are divided into two distinct clades (Fig. 12), with two species as sister to the two *Trachemys* clades. The first *Trachemys* group (clade “E”) consists of *Trachemys gaigeae gaigeae* and *Trachemys scripta elegans*, although they do not form a clade, and are sister to the two *Trachemys* clades. These two species represent the northernmost *Trachemys* species and the only modern *Trachemys* species found in the United States, although some of the others may cross the southern border. Although they are not monophyletic, both these northern species share several features, including; an enlarged squamosal, relatively short postorbital, a subacute termination of the supraoccipital crest, basisphenoid–basioccipital suture straight medially with lateral edges sloped posteriorly, continuous anterior midline of the alveolar suture not set apart as a ledge, very low median keel on carapace made of a single ridge, rugose surfaces beneath the marginal and first pleural scutes, deeply notched pygal, long cervical scute, diagonal rugosities on marginal surface, broad and long gulars, and the abdominal–femoral sulcus being concavely curved toward the posterior of the shell medially. Additionally, several key characters of these northern *Trachemys* species are distinct due to their plasticity within these animals, including; the anterior termination of the prefrontal process of the frontal, size and position of the foramina praepalatinum and foramen carotico-pharyngeale, contact of the pterygoid and basioccipital, basisphenoid morphology, shape of the anterior margin of the lower jaws, position of the dorsal projection of the angular, and morphology of posterior-most marginals. The plasticity of these two species may be why they are not monophyletic and form a potytomy with other *Trachemys* species. While *Trachemys terrapen* is the only *Trachemys* species not from the United States with long gulars, the United States *Trachemys* are the only members of the genus with the anterior midline of the alveolar surface continuous with the ventromedial portion of the dentary, not set apart as a ledge or shelf. When both these species are included in phylogenetic studies they are often found to be sister taxa (Jackson et al., 2008; Wiens, Kuczynski & Stephens, 2010, figs. 2–3; Fritz et al., 2012; Guillon et al., 2012; McCranie et al., 2013; Parham et al., 2013, 2015; Vargas-Ramírez et al., 2017), although this is not always the case (Seidel, 2002; Stephens & Wiens, 2003; Wiens, Kuczynski & Stephens, 2010, fig. 1). It is noted that the 50% majority rule consensus trees recovered *Trachemys scripta elegans* and *Trachemys gaigeae gaigeae* as sister taxa basal to other *Trachemys* species (see Figs. S73 and S74).

The second *Trachemys* clade includes taxa from southern North America, Central America, the Caribbean, and South America (clade “F”). Within this southern clade lies two other smaller clades, a southern mainland *Trachemys* clade (clade “G”) and a Caribbean (or Antilean) *Trachemys* clade (clade “H”). The southern clade (clade “G”), consisting of *Trachemys grayi grayi* + (*Trachemys dorbigni dorbigni* + (*Trachemys yaquia* + (*T. ornata* + *Trachemys nebulosa nebulosa*))), all represent species from the southern mainland, including Mexico, Central and South America. The derived subclade consisting of western Mexican *Trachemys* (*T. yaquia* + (*T. ornata* + *T. nebulosa nebulosa*)) are characterized by a posteriorly tapered squamosal, small foramen palatinum posterius, lack of a foramen carotico-pharyngeale, ventral slope of supraoccipital crest beginning at supraoccipital–parietal suture, rounded anterior tip of basisphenoid, lack of basioccipital process of basisphenoid, a serrated cutting edge of the lower jaw, the pygal extends beyond marginal–vertebral sulcus for its entire width, anal notch of xiphiplastron curved anteroposteriorly, and a horizontal pectoral–abdominal seam. Sister to this clade lies *T. dorbigni dorbigni,* from southeastern South America, which is characterized by a straight dorsal surface of the supraoccipital crest in lateral view, a basisphenoid–basioccipital suture that is straight medially but with lateral edges that slope posteriorly, widest portion of the posterior plastral lobe located anteriorly, and a concavely curved abdominal–femoral sulcus. Sister to *T. dorbigni dorbigni* and clade “G” is *T. grayi grayi* from southwestern Mexico, characterized by a shallow cranial depth relative to condylobasal length, pterygoid contributes to the ventral border of the foramen nervi trigemini, lateral edges of rostral projection of basisphenoid being convex posteriorly and concave anteriorly, peripherals not notched, vertebral I less than width of vertebral II, vertebral I hour-glass shaped (constricted at mid-length), marginal or marginals contacted by seam between pleural scutes II and III is the posterior of or posterior to marginal VII, gentle curve makes up the anal notch of the xiphiplastron, and the inguinal scute does not contact marginal VIII.

Sister to this southern mainland *Trachemys* clade lies a clade of Caribbean (or Antillean) *Trachemys* species (clade “H”). The Caribbean *Trachemys* clade is comprised of *Trachemys decorata* + *Trachemys stejnegeri stejnegeri* + *Trachemys terrapen* in a polytomy. This clade is distinguished by several features, including; a broadly rounded or jagged sutural contact between the pterygoids and the vomer, a ventromedial surface of the dentary that can be rounded or flattened, a short plastron relative to the carapace, the highest point of the carapace posterior to the midline, vertebral I not constricted, first marginal short and wide with the width exceeding the length, gulars may extend beyond anterior margin of the epiplastra, and inguinal scutes that do not project laterally. The 50% majority rule consensus trees recover more resolution among these Caribbean taxa, with *T. decorata* sister to *T. stejnegeri stejnegeri* + *T. terrapen* (see Supplemental Information).

A clade of fossil *Trachemys* (clade “I,” discussed further below) forms a polytomy with *T. scripta elegans*, *T. gaigeae gaigeae*, and the southern *Trachemys* clade (clade “F”).

**Phylogenetic relationships of fossil *Trachemys:*** This clade (clade “I”) consists of (*T. hillii* + *T. idahoensis*) sister to (*T. haugrudi* + *T. platymarginata* + *T. inflata*). The fossils are characterized by several features, including; a relatively wide narial opening, wide interorbital width of cranium, wide triturating (=alveolar) surfaces of the upper jaws, a large foramen palatinum posterius, a foramen carotico-pharyngeale that contacts the pterygoid–basisphenoid suture by a separate and short (pterygoid–pterygoid) suture, the presence of a depression in the pterygoid just lateral to the basisphenoid, the basisphenoid–basioccipital suture curved anteriorly, absence of a basioccipital process of the basisphenoid, presence of prominent tuberculate denticles on the alveolar surface of the lower jaws, rugose dorsal surface texture of the carapace, carapace not flared posteriorly, neural VII hexagonal, 2 suprapygals present, ribs on proximal end of costals not prominent, between 7 and 8 ridges distally and rugosity proximally on costals, seam between vertebral I and pleural I contacts anterior half of marginal I or is anterior to marginal I, anterior edge of the plastron does not extend beyond anterior edge of carapace, entoplastron broader than long, expanded xiphiplastron, long gular overlap, and lateral-most edges of the abdominal–femoral sulcus directed posteriorly.

*Trachemys hillii* and *T. idahoensis* are both from the Midwestern United States, and are found to be sister taxa in the analysis (clade “J”). Indeed, the other fossil *Trachemys* clade, made up of *T. haugrudi* + *T. platymarginata* + *T. inflata*, are from the eastern and southeastern United States (clade “K”). The Midwestern fossil *Trachemys* clade is characterized by several features, including; a short and deep cranium, absence of a prefrontal process on the frontal, jugal not reaching the orbit, short postorbital length, foramina praepalatinum exposed ventrally, subacute posterior termination of the supraoccipital crest, medial constriction of the posterior of the basisphenoid, flattened ventral surface of mandible, broad triturating surface of dentaries, oval shell with slightly concave lateral edges, shell relatively flattened with no distinct highest point in lateral view, anterior margin of carapace with notches only between marginals, nuchal barely overlapped by pleural I, neurals I and VIII hexagonal, peripherals only slightly notched, vertebral I constricted at mid-length with its anterolateral border not confined to the nuchal, posterior carapace notching to between marginals II and III, epiplastra not forming right angle at the suture, entoplastron extended posteriorly, gulars broad and long, and the posterior plastral lobe indentation at or just posterior to the lateral edge of the femoral–anal seams is absent or very faint. It is noted that since the type of *T. hillii* is missing the anterior portion of the shell and most of the cranial material, the majority of these features are derived from *T. idahoensis*.

The southeastern fossil *Trachemys* clade is made up of two taxa from Florida (*T. inflata* and *T. platymarginata*) and one from Tennessee (*T. haugrudi*). The southeastern fossil *Trachemys* clade (clade “K”) is distinguished by numerous features from other fossil *Trachemys*, including; lateral edges of the prefrontal taper anteriorly, enlarged squamosal, vomer tapers to a single point of contact with the pterygoids, anterior tip of basisphenoid rounded, posterolateral edge of basisphenoid is two-sided, sculptured dorsal surface of nuchal, posterior of pygal deeply notched, peripherals with lateral edge swollen to form a lip, vertebral I short, marginal I is long and narrow, and anterior plastral lobe with lateral sides inflated. Although the late Hemphillian *T. haugrudi* forms a polytomy with the Florida fossil taxa (*T. platymarginata* and *T. inflata*), it can still be distinguished from these by several characters, including; narrow narial opening, small orbits, narrow minimum interorbital distance, a small to absent crista praetemporalis, a smooth anteroventral border of the premaxilla, labially flared tomial edge of the maxilla, thin triturating surfaces of the upper jaws, pterygoids narrow mediolaterally, flattened ventromedial surface of the dentary, no tuberculate denticles on alveolar surface, small adult body size (less than 250 mm), highest point of the carapace posteriorly, cervical scute does not project beyond anterior extreme of first marginals, anterior and posterior margins of vertebral 1 on nuchal approximately equal, flat anterior margin of the pygal, elongate cervical underlap, vertebral 2 broader than vertebral 1, number of posterior-most marginal bearing a notched distal border between marginals 3 and 4, posterior-most marginal lies dorsal to marginals lateral to it, no projection of the entoplastron at the humeral–gular sulci, no indent at the lateral edge of the plastron at the hypoplastron–xiphiplastron sutural contact, and broad gulars. As *T. inflata* is known from mostly isolated material, cranial characters, or characters dealing with complete shells, that differentiate *T. haugrudi* from the Florida fossil taxa are taken mainly from *T. platymarginata*. Furthermore, *T. platymarginata* is distinguished from *T. inflata* by several characters, including; a serrated cutting edge of the lower jaws, presence of a median symphysial ridge on the lower alveolar surface, larger shell, lacks serrations on the anterior carapacial edge, median carapacial keel present anteriorly and posteriorly rather than just posteriorly, smooth marginal scute area on nuchal, rugose vertebral 1 area on nuchal, lack of notches between cervical scute and first marginals, longer first marginal area on nuchal, elongate cervical scute, lack of diagonal rugose lines or ridges on marginal dorsal surface, no depression between 12th marginals, serrated posterior margin of the plastron, straight anterior epiplastral margin viewed ventrally, curved anterior epiplastral margin viewed anteriorly, anterior and medial edges of epiplastra form right angle with each other, deep “V-shaped” indentation between posterior anals, flat abdominal–femoral sulcus, and anterior apex of femoral–anal sulcus acute. Four steps separate the two fossil *Trachemys* clades. The 50% majority rule consensus tree provided more resolution of the southeastern fossil *Trachemys* species, recovering *T. haugrudi* as sister to the two fossil Florida species (see Supplemental Information).

Overall, *Trachemys* taxa included in this study did have several common features linking them. These features included, a relatively narrow zygomatic arch, broad and/or wide pterygoids, an acute anterior tip of the basisphenoid, two openings in the pelvis, posterior edge of the carapace doubly notched, carapace flared posteriorly, anteroposterior length of the nuchal under the first marginal is the same length or longer than the length from the posterior margin of the nuchal to the point of intersection between vertebral I–pleural I–marginal I, pronounced notching of peripherals, posterolateral marginal serrations present, posterior plastral lobe inflated laterally, the entoplastron does not project at the gular–humeral sulci, and the distal ends of the femoral–anal seam curve anteriorly. Bootstrap values were not absolutely high for the *Trachemys* clades in the present study, although large numbers of polymorphic characters help lower these values. Membership within the clades did not vary greatly, although interrelationships among the taxa within the various clades was more variable. Additionally, less resolution was found with northern species of *Trachemys*, which formed a polytomy with fossil and southern *Trachemys* species.

*Trachemys* is monophyletic, with several fossil species previously attributed to *Trachemys* nested within. It is unsurprising that some of the clades are centered on geographic regions. Clade “E,” while not monophyletic, has the northern mainland *Trachemys* species from the United States basally in the genus. Clade “F,” contains *Trachemys* that lie south of the United States. Clade “G,” nested within “F” is made up of species from from the southern mainlaind, including Mexico and South America. This suggests that the ancestor of *T. dorbigni dorbigni* migrated from western Mexico before heading south into South America. Clade “H,” also nested within clade “F,” is made up of *Trachemys* species from the Caribbean (or Antilles). The Caribbean *Trachemys* species appear to have been isolated for a significant amount of time, helping create a clade distinct from mainland species. Recent molecular studies have found Caribbean *Trachemys* species sister to northern *Trachemys* species (Fritz et al., 2012; Parham et al., 2013; Vargas-Ramírez et al., 2017). While Fritz et al. (2012) calculated mean estimates of 8.57 and 10.21 Ma for the divergence of the Caribbean *Trachemys* from northern *Trachemys*, Vargas-Ramírez et al. (2017) calculated mean estimates of only 3.26 and 3.38 Ma, implying more work is needed to determine these divergence dates. Based on the hypothesized relationships in the present study, it is uncertain whether the Caribbean taxa evolved from a Central American or a southeastern United States ancestor. Regardless, it has been shown that *Trachemys* can be saltwater tolerant for prolonged periods (Dunson & Seidel, 1986). This may have allowed this migration from the mainland to the islands. Although *T. grayi grayi* is basal to these other southern *Trachemys* species, it is believed that the relationships of *T. grayi grayi* are not fully understood. This taxon may be found to be more derived in a clade with other southern mainland *Trachemys* species eventually. Clade “I” contains the fossil *Trachemys* species, although their monophyly may be an artifact of the characters being scored. It seems that two distinct groups of fossil *Trachemys* evolved separately, one from the Midwestern United States, and another in the southeast. Both fossil lineages would have evolved prior to the late Hemphillian. Based on morphology it seems these fossil lineages went extinct without leading directly to any of the modern lineages, although more fossil taxa are needed to more accurately infer this. Additionally, the support for this portion of the tree is not very strong, and three extra steps collapses the fossil clade. More specimens and data are needed for more clarification of the morphological relationships of *Trachemys* species.

Previous studies have also explored the relationships of modern *Trachemys* taxa using morphological and/or molecular data. Seidel (1988) was one of the first to study the interrelationships of the genus using morphological data. His data was incorporated into the present study. Seidel (1988, fig. 4) found the northern mainland *Trachemys* taxa (*T. scripta* and *T. gaigeae gaigeae*) to be basal within *Trachemys*. He found *T. stejnegeri*, and its various subspecies, to be sister to *T. decorata*. Seidel (1988) also found *T. decussata* to lie basal to other Caribbean (or Antillean) *Trachemys* taxa, similar to some molecular studies (Parham et al., 2013). Seidel (2002) conducted the most thorough study on the morphological relationships among *Trachemys* taxa, and much of his data was also incorporated into the present study and analysis. Nevertheless, not all relationships agree between his study and this one. While he did find *T. scripta* to be sister to all other *Trachemys* species (and subspecies), he found *T. gaigeae gaigeae* to be a derived member and nested within a clade also containing *T. nebulosa nebulosa*, *T. ornata*, and *T. yaquia* (Seidel, 2002, fig. 2). Additionally, he did not find a monophyletic Caribbean (or West Indies) clade of *Trachemys*, and instead found them to form a polytomy with various other *Trachemys* taxa. Stephens & Wiens (2003) presented phylogenies of emydids based on morphological and/or molecular data. They also used two different methods (between-character and between-state) scaling methods when scoring characters. Regardless of whether they used between-character (Fig. 4) or between-state (Fig. 5) scaling, *Trachemys* was paraphyletic in their analyses (Stephens & Wiens, 2003). Even when they combined morphological and molecular data into a single phylogenetic analysis, they still found *Trachemys* to be paraphyletic (Stephens & Wiens, 2003, fig. 7). Indeed, some characters previously believed to be diagnostic of *Trachemys* can be found in other emydid taxa such as *Graptemys* and *Pseudemys*, potentially leading to paraphyletic relationships of the genus. Based on molecular data, Jackson et al. (2008, figs. 1–2) found the majority of their *Trachemys* clades grouped based on biogeography. Indeed, they found the northern taxa of *T. scripta elegans* and *T. gaigeae gaigeae* to be sister taxa and basal to other *Trachemys* species (and subspecies). They also found a distinct Caribbean (or West Indies) clade including *T. decorata*, *T. stejnegeri stejnegeri*, and *T. terrapen*, along with *T. decussata* and several subspecies not included in the present study. This Caribbean clade was sister to a clade from Mexico and Central America that included *T. yaquia* and several taxa not in the current study, although they did not include *T. nebulosa nebulosa* or *T. ornata* (Jackson et al., 2008, figs. 1–2). *T. dorbigni dorbigni* formed a polytomy with two *Trachemys* clades from Mexico and Central America (Jackson et al., 2008). While some relationships between species differ, the larger clades all generally agree between their study and the present one. Spinks et al. (2009) found *Trachemys* to be polyphyletic based on nuclear data, with the included species (*T. scripta scripta*, *T. s. elegans*, *T. stejnegeri*, and *T. taylori*) grouping with members of *Malaclemys*, *Graptemys*, and *Pseudemys* in their different data sets. Thomson & Shaffer (2010, fig. 5), while including only a few species of *Trachemys*, nevertheless found them to be paraphyletic. Wiens, Kuczynski & Stephens (2010) attempted to resolve discordance between mitochondrial and nuclear gene phylogenies, but found two distinct paraphyletic clades of *Trachemys*, one with the United States species and one with other utilized species (*T. emolli*, *Trachemys nebulosa nebulosa*, and *Trachemys venusta venusta*). Even when mitochondrial DNA and nuclear DNA were combined, *Trachemys* came out as paraphyletic with *Graptemys* and *Malaclemys* species present in its clade (Wiens, Kuczynski & Stephens, 2010, fig. 3). Fritz et al. (2012) conducted a study on the relationships of several *Trachemys* species from Central and South America based on molecular data. In their study, they found the northern *Trachemys* species (*T. scripta elegans* and *T. gaigeae gaigeae*) to be basal to other *Trachemys* taxa, and sister to *T. decussata* (Fritz et al., 2012, figs. 2–4), although the latter taxon was not included in the present study. The other *Trachemys* clade consists of Central and South American taxa, with *T. dorbigni dorbigni* basal to other included taxa. This basal placement of *T. d. dorbigni* agrees with its phylogenetic placement in the current study and may suggest an early split of the clade with part quickly migrating south into South America and part remaining farther north in Mexico and Central America. Fossil evidence of *Trachemys* in South American is not currently known prior to the Pleistocene (Cabrera & Luna, 2011). Guillon et al. (2012) also found a monophyletic *Trachemys* and a clade of Caribbean species. While those findings agree with the present study, they also found *T. dorbigni* to lie sister to the northern species from the United States. This sister relationship between the most northern and southern species of *Trachemys* does not agree with the present study, or most other studies on *Trachemys* relationships. McCranie et al. (2013), who focused on *T. emolli*, found several similar relationships, including a clade of *T. scripta* and *T. gaigeae* and a clade of Caribbean species (*T. decorata*, *T. terrapen*, and *T. stejnegeri stejnegeri*). They also found *T. ornata* to be sister to *T. yaquia*, although they did not include *T. nebulosa nebulosa* in their analysis, with *T. dorbigni dorbigni* lying outside this clade (McCranie et al., 2013, figs. 3–4). Their data suggests early evolution in the southern United States followed by migrations to the Caribbean and south to Mexico and Central America. It then appears that there were multiple migrations, potentially farther south, eventually getting to Brazil, and back north into other regions of Mexico. Parham et al. (2013) analyzed the molecular relationships of *Trachemys* taxa from the Greater Antilles. While all *Trachemys* taxa were again not included in their study, the relationships presented by Parham et al. (2013, figs. 1–2) are similar to those of the present study. *T. stejnegeri* was still found to form a clade with *T. decorata* and *T. terrapen*, although they found the latter two to be sister taxa and *T. stejnegeri* to be basal within the clade. Many of the other taxa included in their study were not included in the present study, although *T. grayi grayi* was found basal to the “Antillean clade,” and the northern taxa (*T. scripta* and *T. gaigeae*) formed a clade basal to other *Trachemys* taxa (Parham et al., 2013). Parham et al. (2015, fig. 2), in focusing on *T. ornata* and Mexican *Trachemys*, again found the same relationships between the three Caribbean taxa, with *T. decussata* as sister to this clade. Conversely, while the present study found these three taxa to form a clade as well, it instead found *T. decorata* to be basal and sister to *Trachemys terrapen* + *T. stejnegeri stejnegeri*. Parham et al. (2015) found *T. nebulosa nebulosa* to be basal in a clade of southern mainland *Trachemys* taxa. After *T. nebulosa nebulosa*, they found *T. dorbigni* to be sister to all other southern mainland taxa, which follows several previous studies finding *T. dorbigni dorbigni* to lie relatively basally. Parham et al. (2015) also found *T. ornata* and *T. yaquia* to be sister taxa, similar to the present study. Spinks et al. (2016, figs. 1–5) conducted the most recent molecular phylogenetic analysis on the family Emydidae using nuclear and mitochondrial genes, although they did not include many *Trachemys* taxa (*T. scripta elegans* and *T. stejnegeri*). Vargas-Ramírez et al. (2017) reported the most recent molecular phylogeny focusing on intrageneric relationships of *Trachemys*, including more than half the currently recognized subspecies. They found *T. scripta* and *T. gaigeae* to be sister to Caribbean *Trachemys* taxa and basal to a clade of Central and South American taxa. Among southern mainland species they found *T. dorbigni* to lie basal to *T. grayi* and other southern taxa. Joyce et al. (2013, figs. 1, 3–5), while investigating divergence dating of turtles using fossils for calibration, included several emydid species, including two fossil species. One of the included fossil species, “*Pseudemys*” *idahoensis*, is also in the present study. However, Joyce et al. (2013) determined it to be most parsimoniously placed as a stem *Graptemys*. Their study altered the original data set of Joyce & Bell (2004), which focused on variation within Testudinoidea. The relationships of *T. idahoensis* are focused more within Emydidae and Deirochelyinae in the present study, allowing for its placement among the various deirochelyine genera. This study shows it is better identified as a fossil species of *Trachemys* based on several characters that unite it with this genus rather than *Graptemys*, including; narrow zygomatic arch, squamosal blunt posterodorsally, anteroventral border of the premaxilla smooth but with median notch, tomial edge of the maxilla tapered lingually, presence of a median maxillary ridge on the triturating surface of the upper jaws, broad pterygoids, angled apex of the lower jaws, hooked lower jaws, single serrations present between the marginals on the anterior margin of the carapace, anterior margin of the pygal concave posteriorly, approximately parallel lateral margins of the cervical scute, longer than broad cervical scute, and the entoplastron does project at the humeral–gular sulci. More characters unite *T. idahoensis* with *Trachemys* than *Graptemys*, and it would take 11 additional steps to be within the *Graptemys* + *Malaclemys* clade, and 12 steps to be grouped within the modern *Graptemys* clade. Therefore, it is most parsimoniously placed as a member of the central or Midwestern fossil *Trachemys* clade. The difference in placement of this fossil taxon between *Graptemys* and *Trachemys* may be due to the current data set being focused on variation in emydid turtles rather than a wider range of turtle taxa, such as all testudinoids. These studies often find a close relationship between *Graptemys* (and *Malaclemys*) and *Trachemys* as well (Stephens & Wiens, 2003; Spinks & Shaffer, 2009; Spinks et al., 2009, 2016; Wiens, Kuczynski & Stephens, 2010; Jackson, Nelson & Morris, 2012; Vargas-Ramírez et al., 2017), and so it is not unexpected to find confusion in fossil taxon placement between these genera (Jasinski, 2011, 2013b).

**Phylogenetic relationships of *Pseudemys*, *Graptemys*, and *Malaclemys*:** Similar to previous studies (Stephens & Wiens, 2003; Wiens, Kuczynski & Stephens, 2010; Fritz et al., 2012; Guillon et al., 2012; McCranie et al., 2013; Spinks et al., 2016; Vargas-Ramírez et al., 2017, fig. 3), *Graptemys* and *Malaclemys* were found to be sister taxa in a clade (clade “C”). Rarely are they found to not be sister taxa, such as by Vargas-Ramírez et al. (2017, fig. 2), where they are recovered as successive sister groups, although that study was focused on the molecular phylogenetic relationships of *Trachemys* from Central and South America rather than relationships of other emydid genera. Aside from a portion of the analyses by Stephens & Wiens (2003), all other studies on the phylogenetic relationships of *Graptemys* and *Malaclemys* have utilized exclusively molecular data. In addition to the modern species of *Graptemys* and *Malaclemys*, a recently named fossil *Graptemys* species was also included, *Graptemys kerneri* (Jackson, 1975; Ehret & Bourque, 2011). Modern *Graptemys* species, formed a clade (clade “D”) with *Malaclemys terrapin terrapin* and *G. kerneri* as sister taxa in the strict consensus phylogenetic analysis (Fig. 12). The *Graptemys* + *Malaclemys* clade is characterized by several features, including; a thick anterior border of the processus inferior parietalis, a posterodorsally tapered squamosal, a nearly flat surface medially on the triturating surface of the upper jaws, an acute to subacute posterior termination of the supraoccipital, basisphenoid–basioccipital suture that is straight medially but with sloped lateral edges, lateral edges of the basisphenoid form a simple two-sided corner with the posterior edge of the basisphenoid, strong lateral tuberosity on the basioccipital, flattened ventromedial surface of the dentary in anterior view, lower jaw not hooked, no ridge on the median lower triturating surface of the dentary, discontinuous anterior midline of the lower alveolar surface, median keel along dorsal midline of carapace, flat anterior margin of pygal, approximately square cervical scute, vertebral 1 with relatively straight lateral edges, posterior plastral lobe widest anteriorly, broader than long entoplastron, and broad gulars. The modern *Graptemys* clade is characterized by a smooth anteroventral border of the premaxilla, a lingually tapered tomial edge of the maxilla, foramen palatinum posterius occurring at the bottom of a deep furrow formed by the palatine and maxilla, pterygoid contacting basioccipital, ventral slope of the supraoccipital crest beginning at the supraoccipital–parietal suture, no notch in the basisphenoid–basioccipital suture, adult females often more than twice the carapace length of adult males, carapace smooth or possessing smooth contours, anterior margin of the carapace is doubly serrated, posterior of pygal deeply notched, peripherals somewhat notched, cervical scute widest posteriorly, posterolateral marginal serrated, anterior epiplastral margin curved and bearing a shallow medial cleft, and strong plastral buttresses. The monophyly of the *Malaclemys* + *Graptemys* clade is well-resolved in relation to other parts of the tree, although the monophyly of *Graptemys* is less well resolved, with only two steps needed to collapse *Graptemys*, *Malaclemys*, and *G. kerneri*. As the fossil species is from the Late Pleistocene, older fossil *Graptemys* and *Malaclemys* taxa are needed to better constrain when these clades split. Additionally, more fossil specimens from this clade may also help better determine the relationships of *G. kerneri*, which was found sister to extant *Malaclemys* and *Graptemys* species (Fig. 12).

As was mentioned above, *Graptemys* and *Malaclemys* are commonly considered to be sister taxa. Indeed, Stephens & Wiens (2003) found *Graptemys* and *Malaclemys* to often be sister taxa (Figs. 5 and 7), although it is noted that when using morphological data and between-character scaling (Fig. 4) these taxa were paraphyletic with *Malaclemys* being sister to all other deirochelyine taxa, and using morphological data and between-state scaling (Fig. 5) these taxa formed a clade, although *Malaclemys* was nested within *Graptemys*. Spinks et al. (2009) often found *Malaclemys* to be sister to *Trachemys*, with *Graptemys* sister to this clade, although the placement of *Malaclemys* varied in their analyses, along with *Trachemys* often not being monophyletic. Several studies find the clade of *Malaclemys* + *Graptemys* to be sister to *Trachemys* (Wiens, Kuczynski & Stephens, 2010, figs. 2, 4; Fritz et al., 2012, figs. 2–4; Guillon et al., 2012, fig. 2; McCranie et al., 2013, fig. 3; Spinks et al., 2016; figs. 1–5). In a recent study on the intrageneric phylogenetic relationships of *Graptemys* based on molecular data, Praschag et al. (2017, fig. 2) found *Graptemys geographica* as sister to two main clades, including one with mainly “broad-headed” species and one with “narrow-headed” species. Indeed, most previous studies that have looked at the intrageneric relationships of *Graptemys* have found *G. geographica* as sister to other *Graptemys* species (McKown, 1972; Lamb et al., 1994; Lamb & Osentoski, 1997; Stephens & Wiens, 2003; Wiens, Kuczynski & Stephens, 2010). Praschag et al. (2017) determined *Graptemys* was taxonomically oversplit and suggested revision of its species was needed, particularly members of their “narrow-headed” species group. The present study uses a member of their broad-headed group (*Graptemys barbouri*) and a member of their narrow-headed group (*Graptemys pseudogeographica*), along with *G. geographica*. The present study finds *G. geographica* sister to *G. pseudogeographica*, with *G. barbouri* sister to them. The disagreement in the position of *G. geographica* may be due to the morphologic characters scored in the present study, the polarity used for those characters, or the amount of morphologic variation present in this geographically widespread species. Ehret & Bourque (2011) recently named the fossil species *G. kerneri*, and felt it was most closely related to G. *barbouri*, however they did not perform a phylogenetic analysis. In the present analysis, *G. kerneri*, from the Late Pleistocene of Florida, formed a polytomy with modern *Graptemys* species, and *Malaclemys terrapin*, potentially suggesting it could be a member of either genus. The intrageneric relationships of *Graptemys* have been found difficult to determine due to apparent recent species divergence in various river drainage basins (Lindeman, 2013). The addition of more *Graptemys* taxa may help further resolve this clade along with the addition of characters focused on differentiating *Graptemys* taxa.

Sister to the *Graptemys* + *Malaclemys* clade lies a *Pseudemys* clade (clade “B”), with the three modern species all coming from the eastern United States (Fig. 12). The *Pseudemys* clade is significantly distinct from other clades, taking 14 steps to be collapsed, with high bootstrap values, and is characterized by a short and deep cranium, relatively wide narial openings, a relatively large orbit, small to absent crista praetemporalis, enlarged squamosals, triturating surface of the upper jaws not cusped, large foramen palatinum posterius, rounded ventral surface of the mandible, serrated triturating surface of the lower jaw, median symphysial ridge on lower triturating surface, prominent tuberculate denticles on triturating surfaces, adult females can grow to over 400 mm carapace length, adult males can grow to over 200 mm carapace length, highest point on carapace anterior to midpoint, median keel posterior on carapace, anterior-most suprapygal elongate, vertebral 1 thin in width anteriorly, anal notch of xiphiplastron curved anteromedially, long gulars, lateral edges of the abdominal–femoral sulcus curved posteriorly, and a wide femoral overlap. In previous phylogenetic analyses, *Pseudemys* is generally found to be monophyletic (Stephens & Wiens, 2003; Spinks et al., 2009, 2016; Wiens, Kuczynski & Stephens, 2010; Fritz et al., 2012; McCranie et al., 2013). However, its position within the Deirochelyinae seems to vary. In Stephens & Wiens (2003), *Pseudemys* is monophyletic, although its position within the subfamily is inconsistent in their analyses (Figs. 4, 5 and 7). Using between-character scaling of morphological data, Stephens & Wiens (2003, fig. 4) find *Pseudemys* to be the most derived deirochelyine and sister to a *Chrysemys* clade. However, using between-state scaling of morphological data, Stephens & Wiens (2003, fig. 5) find *Pseudemys* to form a monophyletic group nested within a paraphyletic *Trachemys*. Finally, when Stephens & Wiens (2003, fig. 7) combine morphological and molecular data, they find *Pseudemys* to be monophyletic and basal to (*Trachemys* + (*Graptemys* + *Malaclemys*)). While Spinks et al. (2009) found *Pseudemys* to be monophyletic, not all species included in their analyses were (e.g., *Pseudemys concinna*). Wiens, Kuczynski & Stephens (2010, fig. 3) included all three species of *Pseudemys* from the present study and found the same relationships presented herein, namely *Pseudemys nelsoni* as sister to a clade containing *P. concinna* + *Pseudemys rubriventris*. Fritz et al. (2012) found *Pseudemys* to be monophyletic, sister to *Chrysemys*, and basal to (*Trachemys* + (*Graptemys* + *Malaclemys*)). However, this latter study used few *Pseudemys* taxa. Guillon et al. (2012) found little resolution between *Pseudemys* taxa, although they also found *P. concinna* to be basal to other *Pseudemys* species similar to the present study. McCranie et al. (2013) also had little resolution between the *Pseudemys* species in their analyses. Vargas-Ramírez et al. (2017), while investigating intrageneric relationships of *Trachemys*, found *Pseudemys* to lie sister to *Chrysemys* and basal within Deirochelyinae. Two recent studies focused on the interrelationships of *Pseudemys* using molecular data (Jackson, Nelson & Morris, 2012; Spinks et al., 2013). Jackson, Nelson & Morris (2012) found little evidence for monophyly of numerous species within *Pseudemys*, with *P. concinna*, in particular, found throughout their phylogeny (particularly in clades 2 and 3). *Pseudemys gorzugi* formed the only monophyletic species clade with more than one specimen, although this was still only with two specimens. *P. rubriventris* was found in both clades 2 and 3, similar to numerous other taxa, and the one specimen of *P. nelsoni* was found in clade 3 (Jackson, Nelson & Morris, 2012, fig. 3). Spinks et al. (2013) also conducted a phylogenetic analysis on the molecular interrelationships of *Pseudemys*. They found little resolution among the taxa, and little to no evidence supporting the current taxonomy of *Pseudemys* species and subspecies. However, rather than conducting thorough revisions of the taxonomy of the genus, they suggested more study and analyses involving historical biogeographic and morphologic data (Spinks et al., 2013). This implies that gathering more morphologic data of more species of *Pseudemys*, such as that in this data set, will be useful for more fully understanding the interrelationships of *Pseudemys* taxa.

**Phylogenetic relationships of *Deirochelys*:** Forming a polytomy with the clade (*Pseudemys* + (*Trachemys* + (*Graptemys* + *Malaclemys*))) lies a clade with *Deirochelys*, *Chrysemys picta picta*, and two fossil species referred to *Chrysemys*. The clade containing modern *Deirochelys reticularia reticularia* also contains three fossil taxa (clade “A”) in the strict consensus phylogeny (Fig. 12). *D. reticularia reticularia*, along with the fossil species *Deirochelys carri*, *Deirochelys floridana*, and *P. caelata*, are all from the southeastern United States. This clade is characterized by numerous characters (although since the fossil species are only known from shell material, only shell characters are listed), including; thoracic rib heads that are long, slender, and bowed ventrally, sculptured nuchal dorsal surface (including rugose surfaces under the cervical, marginals, pleurals, and vertebral on the nuchal), pygal does not extend beyond the vertebral–marginal sulcus, diagonal rugose ridges on marginal dorsal surface, seam between vertebral 1 and pleural scute I contacts posterior half of marginal 1 or the seam between marginals I and I, supracaudal extends onto a suprapygal, plastron rugose, entoplastron with more than eight sides in ventral view, and the abdominal–femoral sulcus is concavely curved. Included with modern *D. r. reticularia* in clade “A” are three fossil species, two of which were originally identified as members of *Deirochelys*, including *D. carri*, named by Jackson (1978), and *D. floridana*, named by Hay (1908). *D. floridana* (Hay, 1908) is believed to be from the Pleistocene of Florida (Jackson, 1964). It was identified as conspecific with *D. reticularia* by Jackson (1964) and as a species of *Chrysemys* by Jackson (1978), however the present study implies that it is a species of *Deirochelys*, agreeing with Hay (1908) and Jackson (1964). *D. carri* (Jackson, 1978) is from the late Miocene and latest Clarendonian of Florida (Baskin, 2005). *P. caelata* (Hay, 1908) was originally believed to be from the Pleistocene of Florida, but has since been determined to be from the early late Miocene and early Hemphillian (Jackson, 1976; Prothero, 2005). Additionally, Jackson (1976) stated that *P. caelata* and another fossil species, *Chrysemys carri* (distinct from *D. carri*), named by Rose & Weaver (1966), were synonymous. Based on current results, it appears that *P. caelata* is more accurately considered a species of *Deirochelys*. Whether that means it is synonymous with *C. carri*, and the generic affinities of *C. carri*, is yet to be determined. Additionally, generic referral of the species will be addressed once a larger phylogenetic analysis of fossil emydids is conducted. *Deirochelys* is commonly found to be basal within the Deirochelyinae, usually as the sister group to all other deirochelyines (Stephens & Wiens, 2003, figs. 5, 7; Thomson & Shaffer, 2010, fig. 5; Wiens, Kuczynski & Stephens, 2010, figs. 1–3; Guillon et al., 2012, fig. 2; McCranie et al., 2013, figs. 3–4; Spinks et al., 2016, figs. 1–5). However, it is noted that Stephens & Wiens (2003, fig. 4) found another taxon, in this case *Malaclemys*, to be more basal among deirochelyines when using between-character scaling of morphological data. In the present study, *Deirochelys* is part of a polytomy basally within Deirochelyinae, with *Chrysemys picta picta* and two fossil taxa. This is not often found in many molecular studies, but is not unheard of, with *Chrysemys* the more basal member in some cases (Spinks et al., 2009, figs. 3, 8–9). The 50% majority rule consensus trees recovered *C. p. picta* as more basal when no molecular constraints were used (Fig. S73) and as part of the most basal clade of deirochelyines with the same two basal fossil species (*Chrysemys timida* and *Chrysemys williamsi*) in a polytomy when constraints were used (Fig. S74).

**Phylogenetic relationships of *Chrysemys*:** As mentioned above, *C. p. picta* is part of a polytomy basally in the Deirochelyinae with *Deirochelys* and two fossil taxa (*C. williamsi* and *C. timida*) in the strict consensus phylogenetic tree (Fig. 12). As these taxa do not form a clade, it is uncertain at this time if *C. timida* and *C. williamsi* should belong to *Chrysemys*. While finding *C. p. picta* as basal among deirochelyine taxa is not completely novel, even if it is part of a polytomy with *Deirochelys*, finding the two previously referred fossil species outside of a *Chrysemys* clade is. *C. williamsi* was believed to be from the early Pliocene (Hemphillian) of Florida (Rose & Weaver, 1966), its age was later revised to the early late Miocene and early Hemphillian (Hulbert, 2001; Tedford et al., 2004). The basal *C. timida* from Nebraska (Hay, 1908) is Pleistocene in age (Adler, 1968). This may suggest that, while somewhat superficially morphologically similar, *C. williamsi* and *C. timida* may represent separate genera that did not survive to the present day. It also suggests that some relatively recent deirochelyine lineages went extinct and did not survive to the present. The 50% majority rule consensus trees recovered both fossil *Chrysemys* species further from *C. p. picta* than in the strict consensus tree, coming out as either sister to *Deirochelys* or as successive sister groups to all deirochelyines minus *Chrysemys p. picta* (see Supplemental Information). However, no new genera are used for *C. williamsi* and *C. timida* at this time until more taxa are utilized and more analyses are run, but their inclusion in *Chrysemys* is considered questionable. *Chrysemys* is often found to be monophyletic (Stephens & Wiens, 2003; Fritz et al., 2012), and is most often found to be sister to *Pseudemys* (Stephens & Wiens, 2003; Spinks et al., 2009, fig. 4, 2016, fig. 1; Wiens, Kuczynski & Stephens, 2010, figs. 1, 3; Fritz et al., 2012; Guillon et al., 2012; McCranie et al., 2013, fig. 3; Vargas-Ramírez et al., 2017), However, its position can also vary and other studies have not found a sister relationship between these two taxa (Stephens & Wiens, 2003, fig. 5–8; Spinks et al., 2009, figs. 2–3, 5–9; Thomson & Shaffer, 2010, fig. 5; Wiens, Kuczynski & Stephens, 2010, fig. 2; Jackson, Nelson & Morris, 2012, fig. 3; McCranie et al., 2013, fig. 4; Spinks et al., 2013, fig. 4; Spinks et al., 2016, figs. 2–5). Indeed, in the study by Stephens & Wiens (2003), their *Chrysemys* clade is found to be derived when using between-character scaling of morphological data (Fig. 4), nested in the middle of the tree when using between-state scaling of morphological data (Fig. 5), or basally when combining morphological and molecular data (Fig. 7). However, Fritz et al. (2012, figs. 2–4) did find *Chrysemys* as a basal deirochelyine and sister to *Pseudemys*. Spinks et al. (2016) also recently found *Chrysemys* to be sister to all deirochelyines other than *Deirochelys*. Its position as a basal deirochelyine seems to be correct, however, whether it is more or less derived than *Deirochelys*, and whether it is sister to *Pseudemys*, is less certain.

Based on the fossil taxa and their position on the phylogenetic tree, some things can be inferred on potential divergence times and the ancestry of some deirochelyines. As several of the fossil *Trachemys* taxa are believed to be from the late Miocene and late Hemphillian, all other clades within *Trachemys* must have split before the late Hemphillian, potentially during the Clarendonian. Indeed, as *D. carri* is believed to be from the latest Clarendonian, *Deirochelys* would have evolved before then, and the last common ancestor of *Deirochelys* and (*Pseudemys* + (*Graptemys* + *Malaclemys*) + (*Trachemys*)) would have been before the late Clarendonian. Near, Meylan & Shaffer (2005) used the fossil taxa *Chrysemys antiqua* and *T. inflata* to calibrate the minimum divergence dates of the Emydidae (~34 Ma) and the split between *Trachemys* and *Graptemys* (~5 Ma). They also estimated a divergence time for the *Graptemys*–*Trachemys* clade with molecular data at approximately 15.36 Ma (during the early Barstovian, Ba1 (based on Tedford et al. (2004)), middle Miocene). Fritz et al. (2012, fig. 4) investigated divergence times based on calibrations using the fossil taxa *T. inflata* and *T. idahoensis*. They found that *Graptemys* and *Malaclemys* would have evolved around 10.0 Ma (early Hemphillian), *Pseudemys* and *Chrysemys* would have evolved around 12.0 Ma (Clarendonian), and *Trachemys* would have evolved around 13.0 Ma (early Clarendonian). In fact, the last common ancestor of *Chrysemys*, *Graptemys*, *Malaclemys*, *Pseudemys*, and *Trachemys* would have been around approximately 22.5 Ma (late Arikareean, early Miocene). These divergence times (Fritz et al., 2012, fig. 4) do not disagree with the potential times presented in this study (Fig. 12), and the late Hemphillian taxa *T. haugrudi* and *T. inflata* would not represent the basal-most *Trachemys* species (stem *Trachemys*). Lourenço et al. (2012) found a maximum divergence date of 58.9 Ma (95% confidence interval of 58.8–51.6 Ma) for the Emydidae, although they did not discuss how they arrived at this maximum age for the clade. Joyce et al. (2013) discussed divergence dates for various groups of turtles based on best practices for fossil calibrations. They used *Chrysemys antiqua* and *T. idahoensis* for calibration within the Emydidae, although they noted caution in placing *C. antiqua* in *Chrysemys* and, as noted above, felt *T. idahoensis* was more accurately considered a stem *Graptemys*. Nevertheless, they found a minimum date of 32 Ma and a maximum date of 100.5 Ma for the Emydidae. For the split of *Graptemys*–*Trachemys*, they found a minimum age of 3 Ma and a maximum age of 34 Ma. Spinks et al. (2016) conducted the most recent investigation into the divergence dates for the Emydidae and several of its internal nodes. They calculated a mean divergence date for crown Emydidae at 41.79 Ma (Duchesnean, middle Eocene), crown Deirochelyinae at 31.08 Ma (Whitneyan, early Oligocene), the split between *Chrysemys* and all other deirochelyines (minus *Deirochelys*) at 24.46 Ma (Arikareean, late Oligocene), the split between *Pseudemys* and *Trachemys* + (*Malaclemys* + *Graptemys*) at 20.91 Ma (Arikareean, early Miocene), the split between *Trachemys* and *Malaclemys* + *Graptemys* at 15.6 Ma (Barstovian, middle Miocene), and the most recent common ancestor of *Malaclemys* and *Graptemys* at 13.61 Ma (Barstovian–Clarendonian boundary, middle Miocene). For *Trachemys*, currently the oldest referable specimen not referred to a species comes from the late early Hemingfordian LMA (~18–17.5 Ma, late early Miocene, Burdigalian age) of the upper Fairhaven Member of the Calvert Formation in Virginia (Weems & George, 2013). This also suggests splits prior to the late early Hemingfordian. Indeed, these divergence date estimates from previous studies, based mainly on estimates from molecular data, generally agree with the data in the present study. While most of the bootstrap values in the present phylogenetic analysis were lower than desired, part of this is believed to be due to the number of taxa and characters used. Utilization of more taxa with characters focused on some of the other interrelationships may help strengthen portions of the phylogenetic analysis. Nevertheless, a potential problem comes from the fact that some of these turtles can be quite plastic morphologically, leading to problems in determining differentiating some characteristics. Another potential problem comes from the fact that aquatic freshwater turtle fossils during these potential divergence date ranges are rare. Fossils continue to come to light, but they are often fragmentary or have been left unstudied. Part of the current study, and the studies that will follow, are to better assess this emydid fossil record. As more fossils and material is found, more accurate divergence dates for the various nodes in the Emydidae will be determined.

**Figure 13:**
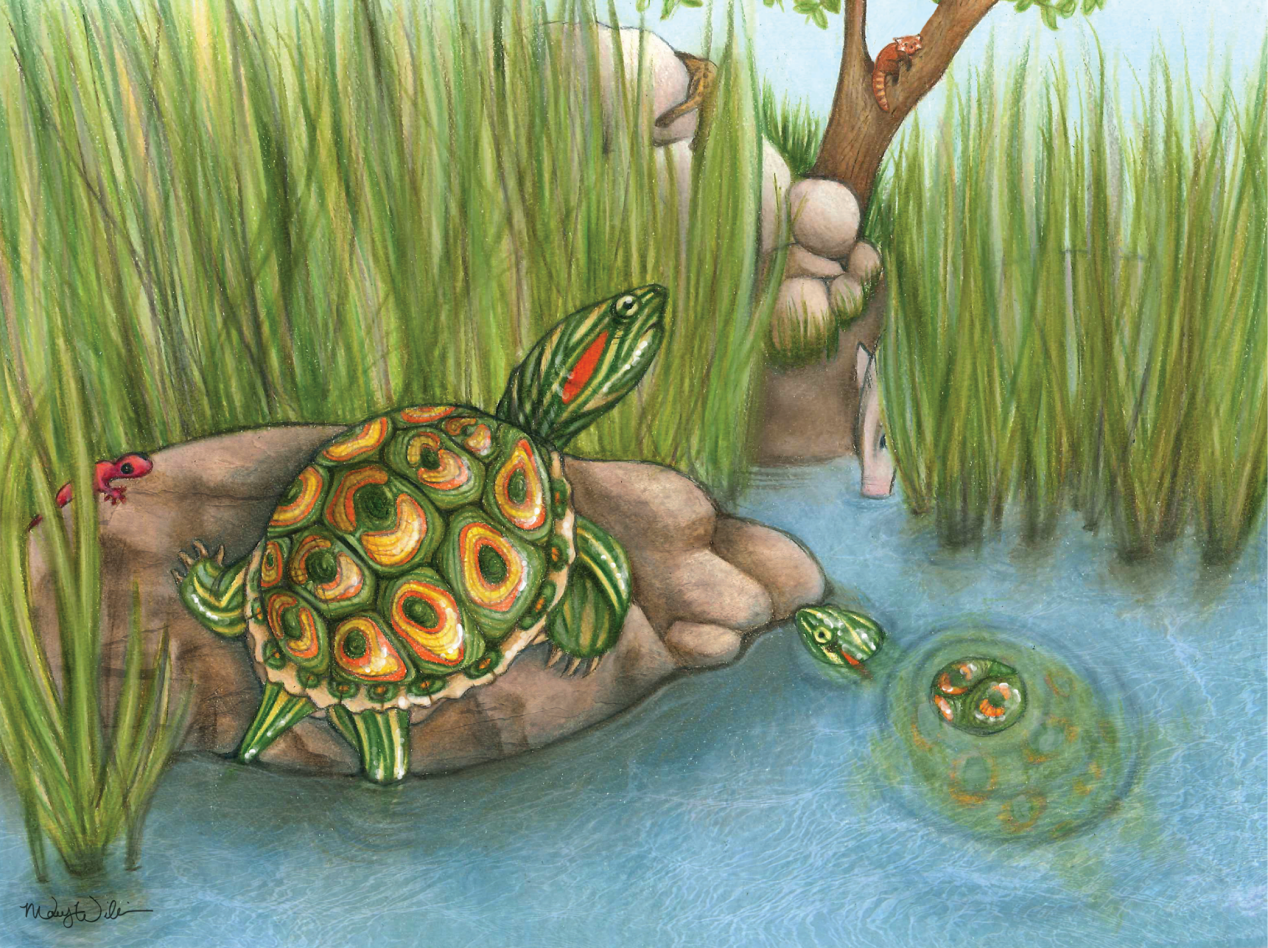
Life reconstruction of *Trachemys haugrudi* during the late Hemphillian at the Gray Fossil Site in eastern Tennessee. Several taxa that would have lived alongside *T. haugrudi* are also shown, including Caudata indeterminate, *Tapirus polkensis*, cf. *Machairodus* sp., and *Pristinailurus bristoli* (Wallace & Wang, 2004; Boardman & Schubert, 2011b; Schubert, 2011; Jasinski, 2013a). Artwork by Mary P. Williams, with permission.

In summary, hypotheses of the phylogenetic relationships between morphologic and molecular data tend to agree in most respects for *Trachemys* and the Deirochelyinae. While some recent phylogenetic analyses have found most of the genera to be monophyletic (Fritz et al., 2012; Guillon et al., 2012; Spinks et al., 2016), others have found *Trachemys* to be paraphyletic (Stephens & Wiens, 2003). Fossil *Trachemys* species form a clade that is sister to clades of northern (nonmonophyletic or paraphyletic) and southern (monophyletic) species of *Trachemys*. The presence of *Trachemys* clades based mainly on biogeography may provide clues to the evolution of these freshwater turtle clades. The saltwater tolerance of *Trachemys* for potentially prolonged periods (Dunson & Seidel, 1986), may have allowed their migration from the mainland to the islands. *Graptemys* form a clade that is sister to *Malaclemys* and a fossil taxon referred to *Graptemys* (*G. kerneri*). This clade is, in turn, sister to a *Pseudemys* clade. *Deirochelys*, *Chrysemys*, and two fossil taxa (*C. timida* and *C. williamsi*) form a polytomy near the base of Deirochelyinae. *Deirochelys* forms a clade with two fossil species previously referred to the genus (*D. carri* and *D. floridana*), and one fossil species previously referred to *Pseudemys* (*P. caelata*). This may imply that the latter belongs to *Deirochelys*. Finally, two fossil species of *Chrysemys*, *C. williamsi* and *C. timida*, and the modern *C. picta picta* form a paraphyletic group with the rest of the deirochelyines. This may suggest that the two fossil species belong to distinct genera. In regard to *T. haugrudi*, it is part of a polytomy with two fossil species from Florida (*T. inflata* and *T. platymarginata*) in a clade. While the strict consensus tree recovered a polytomy between the southeastern United States fossil *Trachemys* species, the 50% majority rule consensus trees recovered the two Florida species as sister taxa, potentially suggesting anagenesis within *Trachemys* in Florida during the late Hemphillian–Blancan. If this does represent anagenesis then it may also suggest a reversal to a less morphologically extreme, potentially more plastic and adaptable type of turtle. This may have been the beginning of *Trachemys* changing in appearance from a more extreme turtle to a more plastic species with the ability to adapt quickly and migrate. However, this is mere conjecture, and more fossil evidence is needed to refute or confirm this hypothesis. *T. haugrudi* represents one of the more derived emydids known, and is the most thoroughly described fossil emydid species, or modern emydid species in regard to osteology for that matter, known. The presence of *T. haugrudi* at the Gray Fossil Site shows the presence of a deirochelyine emydid living with a unique flora and fauna at a time when little is known about the eastern United States and a time when deirochelyine emydids were evolving into forms more closely related to those of the present day (Fig. 13).

## Supplemental Information

10.7717/peerj.4338/supp-1 Supplemental Information 1 Appendix 1: Specimens examined.

10.7717/peerj.4338/supp-2 Supplemental Information 2 Appendix 2: Character list.

10.7717/peerj.4338/supp-3 Supplemental Information 3 Appendix 3: Character–taxon data matrix.

10.7717/peerj.4338/supp-4 Supplemental Information 4 Appendix 4: *Trachemys haugrudi* osteology atlas.

10.7717/peerj.4338/supp-5 Supplemental Information 5 Appendix 5: Figure Captions for 50% majority rule consensus trees from phylogenetic analysis.

10.7717/peerj.4338/supp-6 Supplemental Information 6 Appendix 6: Geologic and temporal data for fossil taxa used in the phylogenetic analysis in the present study.

10.7717/peerj.4338/supp-7 Supplemental Information 7 *Trachemys haugrudi* osteology atlas: Supplemental Figures S1–S72. *Trachemys haugrudi* is represented by multiple individuals with many that are nearly complete. This appendix shows representative specimens and includes all known elements of *T. haugrudi* from different individuals and across the entire skeleton and represent the holotype, paratypes, and referred specimens. All the specimens figured in this atlas are referred to in the main text. The specimens also seek to show the general variation present in *T. haugrudi*. Captions are provided in Appendix 4.

10.7717/peerj.4338/supp-8 Supplemental Information 8 Supplemental Figures S73–S74–50% majority rule phylogenetic trees.

## Institutional Abbreviations

ACM: Beneski Museum of Natural History, Amherst College, Amherst, USA
AMNH: American Museum of Natural History, New York, USA
ANSP: Academy of Natural Sciences of Philadelphia (now Academy of Natural Sciences of Drexel University), Philadelphia, USA
CM: Carnegie Museum of Natural History, Pittsburgh, USA
ETMNH: East Tennessee State University and General Shale Brick Natural History Museum, Gray, USA
ETVP: East Tennessee State University, Vertebrate Paleontology Laboratory, Department of Geosciences, Johnson City, USA
KUVP: The University of Kansas Natural History Museum and Biodiversity Research Center, Lawrence, Kansas, USA
MSUVP: Michigan State University Museum, East Lansing, USA
SDSM: Museum of Geology, South Dakota School of Mines and Technology, Rapid City, USA
SEJ V: reference collection of the author, Steven E. Jasinski
UCM: University of Colorado Museum, Boulder, USA
UF: University of Florida, Florida Museum of Natural History, Gainesville, USA
UF/TRO: specimens formerly in the collection of the Timberlane Research Organization, Lake Wales, Florida, now housed at the Florida Museum of Natural History, Gainesville, USA
UNSM: University of Nebraska State Museum, Lincoln, USA
USNM: United States National Museum of Natural History, Smithsonian Institution, Washington, D.C., USA
UUZM: Uppsala University Museum of Zoology, Uppsala, Sweden
WFI: Wagner Free Institute, Philadelphia, USA
YPM–PU: Yale Peabody Museum–Princeton collection, New Haven, USA.

## Additional Information and Declarations

### Data Availability

The following information was supplied regarding data availability: The raw data has been supplied as Supplemental Dataset Files.

### New Species Registration

The following information was supplied regarding the registration of a newly described species: Publication LSID: urn:lsid:zoobank.org:pub:79D23F9D-EB5C-4CB4-8C2E-F00B63E00624; Trachemys haugrudi sp. nov. LSID urn:lsid:zoobank.org:act:E71A865B-BD3B-4B45-BBFD-CC10B5CB0C9A.
