# Supplementary material for: A new slider turtle (Testudines: Emydidae: Deirochelyinae: Trachemys) from the late Hemphillian (late Miocene/early Pliocene) of eastern Tennessee and the evolution of the deirochelyines
Source: PeerJ. 2018 Feb 13;6:e4338. doi: 10.7717/peerj.4338 (PMC5815335; doi:10.7717/peerj.4338)
Supplement: Supplemental Information 1 [file peerj-06-4338-s001.doc]

**A new slider turtle (Testudines: Emydidae: Deirochelyinae: *Trachemys*) from the late Hemphillian (late Miocene/early Pliocene) of eastern Tennessee and the evolution of the deirochelyines**

Steven E. Jasinski

**Appendix 1: Specimens examined**

Specimens listed are those utilized for this study. They were used for scoring characters and double-checking previously scored characters for various taxa. Many of the more tropical species were not rescored because specimens or individuals could not be acquired. Several of the scute characters for modern species were scored from photographs. Any time this latter method was used, a positive and confident identification was given to the turtle in each photograph beforehand. If this could not be done, then the photographs were not used. For a review of modern *Trachemys* species and their identifications, see *Jasinski* (*2013a*). Specimen numbers that are **bold and underlined** represent type specimens.

**Institutional Abbreviations**

**ACM**, Pratt Museum of Natural History, Amherst College, Amherst, USA; **AMNH**, American Museum of Natural History, New York, USA; **ANSP**, Academy of Natural Sciences, Philadelphia, USA; **CM**, Carnegie Museum of Natural History, Pittsburgh, USA; **ETMNH**, East Tennessee State University and General Shale Brick Natural History Museum, Gray, USA; **ETVP**, East Tennessee State University, Vertebrate Paleontology Laboratory, Department of Geosciences, Johnson City, USA; **KUVP,** The University of Kansas Natural History Museum and Biodiversity Research Center, Lawrence, Kansas, USA; **MSUVP**, Michigan State University Museum, East Lansing, USA; **SDSM**, Museum of Geology, South Dakota School of Mines and Technology, Rapid City, USA; **SEJ V**, reference collection of the author, Steven E. Jasinski; **UCM**, University of Colorado Museum, Boulder, USA; **UF**, University of Florida, Florida Museum of Natural History, Gainesville, USA; **UF/TRO**, specimens formerly in the collection of the Timberlane Research Organization, Lake Wales, Florida, now housed at the Florida Museum of Natural History, Gainesville, USA; **UNSM**, University of Nebraska State Museum, Lincoln, USA; **USNM**, United States National Museum of Natural History, Smithsonian Institution, Washington, D.C., USA; **UUZM**, Uppsala University Museum of Zoology, Uppsala, Sweden; **YPM-PU**, Yale Peabody Museum - Princeton collection, New Haven, USA.

**Modern EMYDIDAE**

*Chrysemys picta picta*: ETVP 9671, 9675, 9678, 9680, 9682, 9686, 9690, 9691, 9692. USNM 167537.

*Clemmys guttata*: ETVP 9538, 9539, 9540. SEJ V-10.

*Deirochelys reticularia reticularia*: ETVP 9694, 9695, 9696, 9697, 9698. SEJ V-9, 20.

*Graptemys pseudogeographica pseudogeographica*: ETVP 532, 9712, 9713.

*Malaclemys terrapin terrapin*: ETVP 9705, 9708, 9710, 9711.

*Pseudemys concinna concinna*: ETVP 576, 9634, 9642, 9643. SEJ V-1. USNM 60895, 521238.

*Pseudemys nelsoni*: USNM 310748, 310749, 310750, 310751, 310752, 310753, 310756, 310757, 310758, 328478, 328479, 335593, 335595, 335597, 335598.

*Pseudemys rubriventris*: ETVP 9639, 9640. USNM 222398, 222399.

*Trachemys decorata*: USNM 63096.

*Trachemys gaigeae gaigeae*: ETVP 9659. USNM 61216.

*Trachemys scripta elegans*: ETVP 9646, 9647, 9648, 9655, 9656, 9657, 9661, 9662, 9663, 9664, 9665, 9666. SEJ V-11, 12. USNM 245632.

*Trachemys ornata ornata*: ETVP 9654. USNM 46281.

*Trachemys terrapen*: USNM 10351, 10352, 10353, 42877, 73640, 79666, 79667, 79668, 79669, 108242, 222431, 340310.

**Fossil EMYDIDAE** (**Type specimens** are bold and underlined):

*Chrysemys timida*: **YPM-PU 10853**.

*Chrysemys williamsi*: **UF 11561**, 11089, 11562, 11563.

*Deirochelys carri*: **UF 20908**, 6485, 19204, 20887, 20890, 20891, 20899, 20900, 20903, 20906, 24100. AMNH 12995, 12997, 13000, 13777.

*Deirochelys floridana*: **USNM 16679**, 16680.

*Graptemys kerneri*: **UF 239000**, 10572, 19161, 235104, 238540, 238541, 238542, 238543, 238544, 239751, 239754, 239759, 245008, 246202, 246206, 246211, 254852, 255356. UF/TRO 100.

*Pseudemys caelata*: **USNM 2508**, 6064, 10322. AMNH 12992, 12993, 12999. UF 3410, 3415, 3416, 3417, 3418, 3420, 3421, 3423, 3425, 3426, 3427, 3537, 20870.

*Trachemys hillii*: **AMNH 2425**.

*Trachemys idahoensis*: **USNM 12059**, 12060, 12232, 15167.

*Trachemys inflata*: **UF 12460**, 11279, 11280, 11281, 11581, 13214, 16570, 16578, 16579, 18911, 32016, 53910, 55856, 55903, 55911, 55988, 55989, 55990, 55991, 57353, 58074, 58374, 58375, 58404, 58405, 58417, 62055, 65666, 65710, 65740, 65745, 65746, 67967, 67969, 68002, 68029, 90368, 91107, 91108, 91124, 97028, 97029, 101901, 101938, 101995, 103662, 103663, 103664, 103702, 117397, 123830, 130076, 130109, 131914, 131915, 133927, 133928, 208027, 208388, 220617, 220618, 220619, 220620, 220621, 220622, 220623, 220624, 220625, 220626, 220627, 220628, 220629, 220630, 220631, 220632, 220633, 220634, 220635, 220636, 220637, 220638, 220639, 220640, 220641, 220642, 220643, 220644, 220645, 220646, 220647, 220648, 220649, 220650, 220651, 220652, 220653.

*Trachemys platymarginata*: **UF 10046**, 10047, 10048, 10277, 10427, 21888, 21892, 21963, 24099, 124229, 162745, 210027, 210029, 210030, 210031, 212658, 212660, 212665, 212666, 212668, 212670, 212671, 212674, 212675, 212677, 212678, 213818, 213819, 214656, 249003, 254501, 254502, 254769, 254850, 256061. USNM 454823, 454824.

*Trachemys haugrudi*: **ETMNH–8549**, 283, 296, 721, 3522, 3558, 3560, 3562, 4686, 6935, 6936, 7502, 7629, 7630, 7654, 7665, 7688, 7689, 7690, 8311, 8550, 8735, 10390, 10391, 10547, 11642, 11643, 12265, 12424, 12456, 12457, 12522, 12726, 12727, 12753, 12772, 12832, 12833, 12834, 12979, 12988, 13032, 13033, 13036.
