# Supplementary material for: A new slider turtle (Testudines: Emydidae: Deirochelyinae: Trachemys) from the late Hemphillian (late Miocene/early Pliocene) of eastern Tennessee and the evolution of the deirochelyines
Source: PeerJ. 2018 Feb 13;6:e4338. doi: 10.7717/peerj.4338 (PMC5815335; doi:10.7717/peerj.4338)
Supplement: Supplemental Information 2 [file peerj-06-4338-s002.doc]

**A new slider turtle (Testudines: Emydidae: Deirochelyinae: *Trachemys*) from the late Hemphillian (late Miocene/early Pliocene) of eastern Tennessee and the evolution of the deirochelyines**

Steven E. Jasinski

**APPENDIX 2–Character list**

A total of 243 characters were scored for the current study. Character scores from *Stephens & Wiens* (*2003*) were transferred from their frequency bin scoring into ‘numbered’ scoring. Scoring was done with ‘a, b’ or up to 8% of a species sample changed to 0, from 9%–91% changed to 1, and ‘x,y’ or 92%–100% changed to 2. When ‘1 = mixture’, is the character occurred in 9%–91% of studied specimens. Characters where scoring was altered from scoring bins and the state of ‘1 = mixture’ is added are listed as “Added character state”. Characters who were modified by rewording to make things more clear are marked by “Reworded”. Any other changes or modifications are listed with each individual character. If nothing is listed, then the character is taken directly from the last previous citation. Changes and modifications are considered for the last previous publication but not for earlier publications, when characters may have been modified by subsequent authors and studies. Newly formed and scored characters include characters 23, 53, 68, 86, 130, 137, 140, 142, 143, 144, 145, 158, 163, 164, 165, 174, 175, 202, 205, 206, 213, 218, 219, 232, and 233. Numerous other characters were combined, rescored, and rewritten and that information can be found with each individual character. Characters that state a feature “when present” are scored as a ‘?’ if that character is absent.

References used for characters, character acquisition, and for some character scores include: *Bojanus* (*1819*) *Hay* (*1908*), *White* (*1929*), *Galbreath* (*1948*), *Tinkle* (*1962*), *McDowell* (*1964*), *Weaver & Robertson* (*1967*), *Weaver & Rose* (*1967*), *Adler* (*1968*), *Parsons* (*1968*), *Moll & Legler* (*1971*), *Zug* (*1971*), *Ernst & Barbour* (*1972*, *1989*), *Waagen* (*1972*), *Bramble* (*1974*), *Winokur & Legler* (*1975*), *Jackson* (*1977*, *1978*, *1988*), *Killebrew* (*1979*), *Pritchard* (*1979*), *Dobie* (*1981*), *Bertl & Killebrew* (*1983*), *Seidel & Inchaustegui Miranda* (*1984*), *Ward* (*1984*), *Seidel & Smith* (*1986*), *Gaffney & Meylan* (*1988*), *Seidel* (*1988*, *1994*, *2002*), *Ernst* (*1990*), *Gibbons & Lovich* (*1990*), *Legler* (*1990*), *Seidel & Jackson* (*1990*), *Seidel & Palmer* (*1991*), *Burke*, *Leuteritz & Wolf*, (*1996*), *Minx*, (*1996*), *Seidel*, *Stuart & Degenhardt* (*1999*), *Ultsch et al*. (*2001*), *Stephens & Wiens* (*2003*), *Bonin*, *Devaux & Dupré*, (*2006*), *Joyce* (*2007*), *Buhlmann*, *Tuberville & Gibbons*, (*2008*), *Ernst & Lovich* (*2009*), *McCord et al*. (*2010*), *Sterli & de la Fuente* (*2011*), and *Jasinski* (*2013a*). Character set-up is the same as used by *Joyce* (*2007*). Characters derived from previous sources are listed with the previous sources and the character numbers from those previous studies where appropriate. See Appendix 1 for newly scored and utilized specimens. See Appendix 3 for the character scores and taxon-character state matrix. References are all listed within the References section of the main document.

**Cranium**

CHARACTER 1: Skull A

**Character definition**. Cranium (*Seidel & Smith*, *1986*; *Seidel & Jackson*, *1990*, 1; Added character state): 0 = elongate and shallow; 1 = short and deep.

CHARACTER 2: Skull B

**Character definition**. Maximum cranium depth/condylobasal length (*Seidel*, *2002*, F; Reworded): 0 = less than 0.315; 1 = greater than (or equal to) 0.315.

**Nasal**

CHARACTER 3: Nasal A

**Character definition**. Narial opening of cranium (*Weaver & Rose*, *1967*; *Seidel & Smith*, *1986*; *Seidel*, *2002*, V; Reworded): 0 = relatively wide; 1 = relatively narrow.

**Orbit**

CHARACTER 4: Orbit A

**Character definition**. Orbit relative size (*Seidel & Smith*, *1986*; Modified and Split character): 0 = small; 1 = large.

**Prefrontal**

CHARACTER 5: Prefrontal A

**Character definition**. Fissura ethmoidalis (between descending processes of prefrontals) (*McDowell*, *1964*, 13; Reworded): 0 = broadly triangular; 1 = abruptly narrow beneath the small round dorsal passage for olfactory and profundus nerves, so as to be keyhole-shaped.

CHARACTER 6: Prefrontal B

**Character definition**. Lateral edges of prefrontal (*Burke et al*., *1996*, 22; *Stephens & Wiens*, *2003*, 50), in dorsal view: 0 = taper anteriorly; 1 = mixture; 2 = not tapered, lateral edges parallel or form hourglass shape.

**Frontal**

CHARACTER 7: Frontal A

**Character definition**. Minimum interorbital distance (*McDowell*, *1964*; *Stephens & Wiens*, *2003*, 51; Added character state): 0 = wider than nasal chamber; 1 = mixture of wider or narrower than nasal chamber; 2 = narrower than nasal chamber.

CHARACTER 8: Frontal B

**Character definition**. Prefrontal process of frontal (*Bertl & Killebrew*, *1983*; *Stephens & Wiens*, *2003*, 52; Added character state): 0 = absent; 1 = mixture; 2 = present.

CHARACTER 9: Frontal C

**Character definition**. Anterior termination of prefrontal process of frontal, when prefrontal process present (*Stephens & Wiens*, *2003*, 53; Added character state):0 = blunt or rounded; 1 = mixture; 2 = acute.

CHARACTER 10: Frontal D

**Character definition**. Interorbital width of cranium (*Seidel*, *1988*, 8): 0 = less than 15.0% of the condylobasal length; 1 = more than 15.0% of the condylobasal length.

CHARACTER 11: Frontal E

**Character definition**. Frontal (*McDowell*, *1964*; *Stephens & Wiens*, *2003*, 54; Added character state), in dorsal view: 0 = reaches orbital margin; 1 = mixture; 2 = does not contact orbital margin. Note that *Stephens & Wiens* (*2003*) had this scored in reverse, so their scoring had to be switched.

**Parietal**

CHARACTER 12: Parietal A

**Character definition**. Anterior border of processus inferior parietalis (*McDowell*, *1964*, 5; *Seidel & Smith*, *1986*; *Seidel & Jackson*, *1990*, 2; *Stephens & Wiens*, *2003*, 63; Added character state): 0 =thin, with single attachment to the pterygoid medial to Vidian canal, anterior end of epipterygoid equals or exceeds inferior process in anterior extent, forming lateral part of wall of the anterior end of the Vidian canal; 1 = mixture; 2 =thick, width at least one quarter the distance between posterior margins of right and left interorbital foramina, and with the anterior end of the Vidian canal between anterior border of processus inferior parietalis and epipterygoid, epipterygoid shortened anteriorly, its anterior end lying behind the thickened anterior edge of the inferior process of the parietal.

CHARACTER 13: Parietal B

**Character definition**. Crista praetemporalis, dorsoventral ridge along the posterior margin of the fossa temporalis, near parietal-prootic suture (*McDowell*, *1964*; *Stephens & Wiens*, *2003*, 62; Added character state): 0 = small to absent; 1 = mixture; 2 = present and consisting of heavy, enlarged area of spongy bone, with width 1/2 or more that of the posterior margin of fossa temporalis. After preliminary analysis the character ‘presence or absence of the crista praetemporalis’ was discarded because it showed approximately the same pattern of inter-taxon variation but was more difficult to define unambiguously.

**Jugal**

CHARACTER 14: Jugal A

**Character definition**. Jugal (*Bertl & Killebrew*, *1983*; *Stephens & Wiens*, *2003*, 55; Added character state), in lateral view: 0 = does not reach orbit; 1 = mixture; 2 = contributes to orbit.

CHARACTER 15: Jugal B

**Character definition**. Jugal (*Gaffney & Meylan*, *1988*; *Stephens & Wiens*, *2003*, 66; Added character state): 0 = does not contact palatine, jugal cut off from palatine by maxilla; 1 = mixture; 2 = contacts palatine.

CHARACTER 16: Jugal C

**Character definition**. Jugal (*McDowell*, *1964*, 12; *Burke et al*., *1996*, 23; *Stephens & Wiens*, *2003*, 68; Added character state): 0 = does not contact pterygoid; 1 = mixture; 2 = contacts pterygoid at posterior of palate in area of fossa temporalis.

**Zygomatic Arch**

CHARACTER 17: Zygomatic Arch A

**Character definition**. Zygomatic arch (*Minx*, *1996*, ZA; *Stephens & Wiens*, *2003*, 59): 0 = absent; 1 = present.

CHARACTER 18: Zygomatic Arch B

**Character definition**. Zygomatic arch (*Seidel & Smith*, *1986*; *Seidel*, *2002*, U; Reworded): 0 = relatively wide; 1 = relatively narrow.

**Quadratojugal**

CHARACTER 19: Quadratojugal A

**Character definition**. Quadratojugal (*Stephens & Wiens*, *2003*, 60; Added character state): 0 = absent; 1 = mixture; 2 = present.

CHARACTER 20: Quadratojugal B

**Character definition**. Quadratojugal, when present (*Burke et al*., *1996*, 21; *Stephens & Wiens*, *2003*, 61; Added character state): 0 = reduced, contacting quadrate but not to jugal; 1 = mixture; 2 = contacts both jugal and quadrate.

**Squamosal**

CHARACTER 21: Squamosal A

**Character definition**. Squamosal (noted by *McDowell, 1964*, 9, to be = quadratojugal of most earlier authors; Reworded): 0 = weakly joined to surrounding bones by sutures with little squamous overlap, relatively small, and apparently slightly kinetic, its anterior and nearer to the tympanic cavity than the orbit, jugal contact, if present, taking up less than half the anterior border of the squamosal; 1 = large and firmly anchored to the surrounding bones by sutures with extensive squamous overlap, its anterior end nearer to orbit than to the tympanic cavity, most of its anterior border firmly joined to jugal and most of its dorsal border firmly joined to the postorbital.

CHARACTER 22: Squamosal B

**Character definition**. Squamosal (*Seidel, 1988*; *Seidel, 2002*, H): 0 = tapered posterodorsally; 1 = intermediate; 2 = blunt posterodorsally.

CHARACTER 23: Squamosal C

**Character definition**. Squamosal (NEW): 0 = reduced, anteriorly does not contact the posterior edge of the postorbital; 1 = enlarged, anteriorly does contact the postorbital. Note that an enlarged squamosal means that the quadratojugal is reduced and does not reach the dorsal-most ridge of the zygomatic arch.

**Postorbital**

CHARACTER 24: Postorbital A

**Character definition**. Postorbital length (arch) of cranium (*Seidel, 1988*, 9): 0 = more than 15.5% of the condylobasal length; 1 = less than 15.5% of the condylobasal length.

**Premaxilla**

CHARACTER 25: Premaxilla A

**Character definition**. Posteriorly directed process of premaxilla in nasal chamber (*Killebrew, 1979*; *Stephens & Wiens, 2003*, 49; Added character state): 0 = absent (0); 1 = mixture; 2 = present. When present, the process is visible through the fossa nasalis (*Stephens & Wiens, 2003*).

CHARACTER 26: Premaxilla B

**Character definition**. Premaxillary height of cranium (*Seidel, 1988*, 10): 0 = more than 7.5% of the condylobasal length; 1 = less than 7.5% of the condylobasal length.

CHARACTER 27: Premaxilla C

**Character definition**. Anteroventral border of premaxilla (*Weaver & Rose, 1967*; *Minx, 1996*, PR; *Stephens & Wiens, 2003*, 29): 0 = smooth; 1 = smooth but with notch; 2 = notched, notch defined by two cusps; 3 = hooked beak.

**Maxilla**

CHARACTER 28: Maxilla A

**Character definition**. Maxilla (*McDowell, 1964*; *Stephens & Wiens, 2003*, 65; Added character state): 0 = separated from quadratojugal by jugal; 1 = mixture; 2 = has posterior process that contacts quadratojugal. The description of this character follows *McDowell* (*1964*) except that the element referred to here is the quadratojugal (following *Gaffney, 1979*), which was described and figured as the squamosal by *McDowell* (*1964*).

CHARACTER 29: Maxilla B

**Character definition**. Maxilla and squamosal united in (*Weaver & Rose, 1967*): 0 = less than or equal to 50% of specimens; 1 = more than 50% of specimens.

CHARACTER 30: Maxilla C

**Character definition**. Tomial edge of the maxilla (*Seidel, 1988*, 12): 0 = tapered inward (lingually); 1 = flared laterally (labially), not tapered inward (lingually).

CHARACTER 31: Maxilla D

**Character definition**. Posteroventral surface of upper jaw (*Stephens & Wiens, 2003*, 31; Added character state): 0 = not serrated; 1 = mixture; 2 = serrated.

CHARACTER 32: Maxilla E

**Character definition**. Cutting surface of upper jaw (*Weaver & Rose, 1967*; *Seidel & Smith, 1986*; *Seidel & Jackson, 1990*, 7; *Seidel, 2002*, T; Modified and Added character state): 0 = anterior cusp present on median ridge; 1 = not cusped but medially lacks a shallow notch or angle; 2 = not cusped but medially forms an angle or shallow notch.

CHARACTER 33: Maxilla F

**Character definition**. Median maxillary ridge on triturating surface of upper jaws (*Weaver & Rose, 1967*; *Seidel & Smith, 1986*; *Gaffney & Meylan, 1988*; *Seidel & Jackson, 1990*, 5; *Stephens & Wiens, 2003*, 26; Added character state and Modified wording): 0 = absent, nearly flat surface; 1 = mixture; 2 = present.

CHARACTER 34: Maxilla G

**Character definition**. Concavities medial to maxillary ridge (*Gaffney & Meylan, 1988*; *Stephens & Wiens, 2003*, 27; Added character state): 0 = absent; 1 = mixture; 2 = present. Note this character is scored as unknown (‘?’) if maxillary ridge is absent (scored ‘0’ for character 33).

CHARACTER 35: Maxilla H

**Character definition**. Number of concavities medial to maxillary ridge (*Gaffney & Meylan, 1988*; *Stephens & Wiens, 2003*, 28): 0 = one; 1 =two. Note this character is scored as unknown (‘?’) if maxillary ridge is absent (scored ‘0’ for character 33).

CHARACTER 36: Maxilla I

**Character definition**. Triturating surfaces of upper jaws (*Weaver & Rose, 1967*; Modified and Split character): 0 = thin; 1 = wide.

CHARACTER 37: Maxilla J

**Character definition**. Foramen orbito-nasal (*Stephens & Wiens, 2003*, 19; Added character state): 0 = equal in size to or smaller than palatine process of vomer; 1 = mixture; 2 = large opening with width greater than or equal to that of palatine process of vomer.

CHARACTER 38: Maxilla K

**Character definition**. Foramen orbito-nasal (*Stephens & Wiens, 2003*, 20; Added character state): 0 = not bisected, only one opening present; 1 = mixture; 2 = bisected partially or completely by thin process of palatine.

CHARACTER 39: Maxilla L

**Character definition**. Shape of foramen orbito-nasal (*Stephens & Wiens, 2003*, 21; Added character state): 0 = elongate anteriorly and posteriorly, diameter of longitudinal axis two or more times diameter of perpendicular axis; 1 = mixture; 2 = rounded with length and width subequal.

**Vomer**

CHARACTER 40: Vomer A

**Character definition**. Contact of vomer with pterygoids (*Stephens & Wiens, 2003*, 15): 0 = vomer does not come to a distinct point at contact with pterygoids on ventral surface of palate, suture is broadly rounded at contact or forms jagged horizontal line; 1 = vomer tapers to a single point at contact with pterygoids, and often flared just anterior to contact; 2 = vomer bifurcate at contact with pterygoids; 3 = vomer trifurcate; 4 = vomer with four distinct points; 5 = vomer with five distinct points.

CHARACTER 41: Vomer B

**Character definition**. Vomer–pterygoid contact occurs (*Stephens & Wiens, 2003*, 16; Added character state): 0 = at posterior border of palate; 1 = mixture; 2 = anterior to posterior border of palate.

CHARACTER 42: Vomer C

**Character definition**. Vomer (*McDowell, 1964*; *Stephens & Wiens, 2003*, 25; Added character state): 0 = does not contribute to triturating surface; 1 = mixture; 2 = contributes to triturating surface.

**Palatine**

CHARACTER 43: Palatine A

**Character definition**. Ventromedial surface of palate (*Stephens & Wiens, 2003*, 14; Added character state): 0 = flat; 1 = mixture; 2 = depressed.

CHARACTER 44: Palatine B

**Character definition**. Foramen palatinum posterius size (*McDowell, 1964*; *Burke et al., 1996*, 18; Reworded): 0 = presence of a small fenestra; 1 = expanded into a large fenestra.

CHARACTER 45: Palatine C

**Character definition**. Size of foramen palatinum posterius relative to apertura narium interna (*Seidel & Smith, 1986*; *Seidel & Jackson, 1990*; 8; Reworded and Reordered characters): 0 = large; 1 = small.

CHARACTER 46: Palatine D

**Character definition**. Foramen palatinum posterius (*Burke et al., 1996*, 18; *Stephens & Wiens, 2003*, 17; Added character state): 0 = narrower than or equal to palatine process of vomer; 1 = mixture; 2 = wider than palatine process of vomer. In the latter case the foramen usually consists of a large opening defined by a thin membranous palatine.

CHARACTER 47: Palatine E

**Character definition**. Foramen palatinum posterius (*Stephens & Wiens, 2003*, 18; Added character state): 0 = occurs at bottom of deep furrow formed by posteroventral projections of palatine and maxilla; 1 = mixture; 2 = is clearly visible. In the latter case the foramen occurs in a flat surface of bone or in a shallow depression (Stephens & Wiens, 2003).

CHARACTER 48: Palatine F

**Character definition**. Foramen palatinum posterius (*Gaffney & Meylan, 1988*; *Stephens & Wiens, 2003*, 22; Added character state): 0 = larger than size of foramen orbito-nasal; 1 = mixture; 2 = smaller or same size as foramen orbito-nasal.

CHARACTER 49: Palatine G

**Character definition**. Palatine (*Gaffney & Meylan, 1988*; *Stephens & Wiens, 2003*, 24; Added character state): 0 = excluded entirely from triturating surface of skull; 1 = mixture; 2 = not excluded from triturating surface.

CHARACTER 50: Palatine H

**Character definition**. Foramina praepalatinum (*McDowell, 1964*; *Stephens & Wiens, 2003*, 30; Added character state): 0 = exposed ventrally; 1 = mixture; 2 = not visible ventrally.

CHARACTER 51: Palatine I

**Character definition**. Parietal-palatine contact (*McDowell, 1964*; *Burke et al., 1996*, 19; *Stephens & Wiens*, *2003*, 64; Added character state): 0 = absent, elements separated by pterygoid; 1 = mixture; 2 = contact occurs.

**Epipterygoid**

CHARACTER 52: Epipterygoid A

**Character definition**. Epipterygoid (*Gaffney & Meylan, 1988*; *Stephens & Wiens, 2003*, 67; Added character state): 0 = does not contact jugal; 1 = mixture; 2 = contacts jugal at posterior of palate.

**Pterygoid**

CHARACTER 53: Pterygoid A

**Character definition**. Pterygoids (mediolaterally) (NEW): 0 = narrow and/or thin; 1 = broad and/or wide.

CHARACTER 54: Pterygoid B

**Character definition**. Contact of pterygoid with exoccipital (*McDowell, 1964*, 10; *Seidel & Smith, 1986*; *Seidel & Jackson, 1990*; 9; *Stephens & Wiens, 2003*, 48; Added character state): 0 = absent; 1 = mixture; 2 = present.

CHARACTER 55: Pterygoid C

**Character definition**. Pterygoid (*Gaffney & Meylan, 1988*; *Stephens & Wiens, 2003*, 23; Added character state): 0 = does not contact foramen palatinum posterius; 1 = mixture; 2 = contacts foramen palatinum posterius and forms part of posterior border.

CHARACTER 56: Pterygoid D

**Character definition**. Foramen carotico-pharyngeale (*Gaffney & Meylan, 1988*; *Stephens & Wiens, 2003*, 32; Added character state), foramen located on the ventromedial pterygoid surface: 0 = absent; 1 = mixture; 2 = present.

CHARACTER 57: Pterygoid E

**Character definition**. Foramen carotico-pharyngeale (*Stephens & Wiens, 2003*, 33; Added character state): 0 = large, easily seen with naked eye, subdivisions of foramen visible just below surface; 1 = mixture; 2 = small, barely visible to naked eye, subdivisions not visible.

CHARACTER 58: Pterygoid F

**Character definition**. Foramen carotico-pharyngeale (*McDowell, 1964*, 11; *Stephens & Wiens, 2003*, 34; Added character state): 0 = contacts pterygoid-basisphenoid suture; 1 = mixture; 2 = does not contact pterygoid-basisphenoid suture.

CHARACTER 59: Pterygoid G

**Character definition**. Foramen carotico-pharyngeale, when not contacting pterygoid-basisphenoid suture (*Stephens & Wiens, 2003*, 35; Added character state and Reordered characters for basal versus derived states): 0 = connected to pterygoid-basisphenoid suture by separate, short (pterygoid-pterygoid) suture; 1 = mixture; 2 = is not connected to pterygoid-basisphenoid suture, or directly contacts pterygoid-basisphenoid suture.

CHARACTER 60: Pterygoid H

**Character definition**. Depression in pterygoid just lateral to basisphenoid (*Stephens & Wiens, 2003*, 36; Added character state): 0 = absent; 1 = mixture; 2 = present. When such a depression is present the foramen carotico-pharyngeale usually occurs in the wall of the depression (*Stephens & Wiens, 2003*).

CHARACTER 61: Pterygoid I

**Character definition**. Contact of pterygoid with basioccipital (*Gaffney & Meylan, 1988*; *Stephens & Wiens, 2003*, 47; Added character state): 0 = absent; 1 = mixture; 2 = present.

CHARACTER 62: Pterygoid J

**Character definition**. Pterygoid (*Bertl & Killebrew*, *1983*; *Stephens & Wiens, 2003*, 69; Added character state): 0 = does not contribute to ventral border of foramen nervi trigemini (f.n.t.), dorsal projection of pterygoid separated from f.n.t. by anterior projection of quadrate (processus epipterygoideus) and posterior projection of epipterygoid and/or parietal; 1 = mixture; 2 = contributes to ventral border of f.n.t..

**Supraoccipital**

CHARACTER 63: Supraoccipital A

**Character definition**. Dorsal surface of supraoccipital crest in lateral view (*Stephens & Wiens, 2003*, 56; Added character state): 0 = rounded; 1 = straight (forming continuous line) along more than 3/4 of length; 2 = peaked such that both anterior and posterior halves of the supraoccipital crest are straight, but the posterior half slopes ventrally at an angle from anterior half.

CHARACTER 64: Supraoccipital B

**Character definition**. Ventral slope of supraoccipital crest, when crest is sloped (*Stephens & Wiens, 2003*, 57; Added character state): 0 = begins anterior to supraoccipital-parietal suture; 1 = begins at supraoccipital-parietal suture; 2 = begins posterior to supraoccipital-parietal suture.

CHARACTER 65: Supraoccipital C

**Character definition**. Posterior termination of supraoccipital crest (*Stephens & Wiens, 2003*, 58): 0 = rounded; 1 = acute to subacute.

**Basisphenoid**

CHARACTER 66: Basisphenoid A

**Character definition**. Lateral edges of rostral projection of basisphenoid (*Killebrew, 1979*; *Stephens & Wiens, 2003*, 37), in ventral view: 0 = convex; 1 = concave; 2 = convex posteriorly and concave anteriorly; 3 = straight.

CHARACTER 67: Basisphenoid B

**Character definition**. ‘Wings’ on rostral projection of basisphenoid, anterolateral processes of basisphenoid that often contact foramen carotico-pharyngeale (*Stephens & Wiens, 2003*, 38; Added character state): 0 = absent; 1 = mixture; 2 = present.

CHARACTER 68: Basisphenoid C

**Character definition**. Medial constriction of posterior of basisphenoid, in ventral view (NEW): 0 = absent; 1 = present.

CHARACTER 69: Basisphenoid D

**Character definition**. Anterior tip of basisphenoid (*Stephens & Wiens, 2003*, 39; Added character state): 0 = acute; 1 = mixture; 2 = rounded.

CHARACTER 70: Basisphenoid E

**Character definition**. Foramen at anterior tip of basisphenoid (*Stephens & Wiens, 2003*, 40; Added character state): 0 = absent; 1 = mixture; 2 = present. When such a foramen is present, the previous character (i.e. 69) usually cannot be scored due to the tip of the basisphenoid being not fully ossified.

CHARACTER 71: Basisphenoid F

**Character definition**. Basisphenoid-basioccipital suture (*Bertl & Killebrew*, *1983*; *Stephens & Wiens, 2003*, 41): 0 = straight; 1 = curved anteriorly; 2 = straight medially, but lateral edges sloped posteriorly.

CHARACTER 72: Basisphenoid G

**Character definition**. Basisphenoid-basioccipital suture (*Stephens & Wiens, 2003*, 42; Added character state): 0 = not notched; 1 = mixture; 2 = medial notch present.

CHARACTER 73: Basisphenoid H

**Character definition**. Basioccipital process of basisphenoid, small posteromedial projection of basisphenoid (*Stephens & Wiens, 2003*, 43; Added character state): 0 = absent; 1= mixture; 2 = present.

CHARACTER 74: Basisphenoid I

**Character definition**. Lateral edge of basisphenoid (*Stephens & Wiens, 2003*, 44; Added character state): 0 = forms simple two-sided corner with posterior edge of basisphenoid; 1 = mixture; 2 = three-sided corner with posterior edge of basisphenoid.

CHARACTER 75: Basisphenoid J

**Character definition**. Cranium at basisphenoid (*Seidel, 1988*, 11; Reworded): 0 = deep, with depth at the anterior apex of the basisphenoid more than 31.5% of the condylobasal length; 1 = mixture; 2 = shallow, with depth at the anterior apex of the basisphenoid less than 31.5% of the condylobasal length.

**Basioccipital**

CHARACTER 76: Basioccipital A

**Character definition**. Tuberculum basioccipitale (*Stephens & Wiens, 2003*, 45): 0 = absent; 1 = present.

CHARACTER 77: Basioccipital B

**Character definition**. Tuberculum basioccipitale, when present (*Stephens & Wiens, 2003*, 46; Added character state): 0 = directed posteriorly; 1 = mixture; 2 = directed ventrally.

CHARACTER 78: Basioccipital C

**Character definition**. Basioccipital (*McDowell, 1964*, 2): 0 = with strong lateral tuberosity that extends lateral to lagena and forms floor of the recessus scalae tympani; 1 = without strong lateral tuberosity, not extending lateral to lagena, contributing to the medial wall of the recessus scalae tympani, but not to the floor of that recess, instead the exoccipital curves downward and forward to form the floor, as well as the posterior wall of that recess.

CHARACTER 79: Basioccipital D

**Character definition**. Postlagenar hiatus (a gap in the suture between basioccipital and processus interfenestralis of opisthotic, filled with connective tissue in life, ventral to the perilymphatic foramen, and immediately posterior to the lagena of the membranous labyrinth) (*McDowell, 1964*, 7): 0 = a large, round hole, more than half as big as perilymphatic foramen; 1 = a small, vertical slit.

**Dentary**

CHARACTER 80: Dentary A

**Character definition**. Apex of lower jaw (*Seidel & Palmer*, *1991*; *Stephens & Wiens, 2003*, 70; Added character state): 0 = angled; 1 = mixture; 2 = rounded.

CHARACTER 81: Dentary B

**Character definition**. Anterior margin of dentary (*McDowell, 1964*; *Stephens & Wiens, 2003*, 71; Added character state), in lateral view: 0 = rounded; 1 = mixture; 2 = forms 90 degree angle ventrally with ventral margin of dentary.

CHARACTER 82: Dentary C

**Character definition**. Ventromedial surface of dentary in anterior view (*McDowell, 1964*; *Stephens & Wiens, 2003*, 72; Added character state): 0 = rounded; 1 = mixture; 2 = flattened.

CHARACTER 83: Dentary D

**Character definition**. Ventral surface of mandible (*Weaver & Rose, 1967*; *Seidel & Smith, 1986*; *Seidel & Jackson, 1990*, 3; *Seidel, 2002*, R): 0 = flattened; 1 = rounded.

CHARACTER 84: Dentary E

**Character definition**. Cutting edge of lower jaw (= mandibular tomium) (*Seidel, 1988*, 14; *Seidel, 2002*, J; *Stephens & Wiens, 2003*, 73; Added character state): 0 = not serrated; 1 = mixture; 2 = serrated.

CHARACTER 85: Dentary F

**Character definition**. Lower jaw (*Stephens & Wiens, 2003*, 74; Added character state): 0 = not hooked; 1 = mixture; 2 = hooked.

CHARACTER 86: Dentary G

**Character definition**. Dentary notch (NEW): 0 = absent; 1 = present.

CHARACTER 87: Dentary H

**Character definition**. Triturating (or alveolar) surface of dentary (lower jaws) (*Weaver & Rose, 1967*; *Seidel & Smith, 1986*; *Seidel & Jackson, 1990*, 6; *Seidel, 2002*, S; *Stephens & Wiens, 2003*, 75; Added character state and Altered wording): 0 = narrow, not spatulate; 1 = mixture; 2 = broad, commonly spatulate.

CHARACTER 88: Dentary I

**Character definition**. Edge of triturating surface of dentary (*McDowell, 1964*; *Stephens & Wiens, 2003*, 76; Added character state): 0 = with distinct lingual border, a sharp angularity setting off the horizontal triturating surface from the medial surface of the dentary; 1 = mixture; 2 = lacks distinct lingual border, and slopes gradually towards the vertical medial face of the dentary.

CHARACTER 89: Dentary J

**Character definition**. Lower triturating surface of dentary (*McDowell, 1964*; *Stephens & Wiens, 2003*, 77; Added character state): 0 = in dorsal view not sharply defined anteromedially, width of anteromedial and lateral triturating surfaces roughly equal; 1 = mixture; 2 = sharply defined anteromedially, width of anteromedial triturating surface at least twice width of lateral triturating surface.

CHARACTER 90: Dentary K

**Character definition**. Ridge of median lower triturating surface of dentary (*McDowell, 1964*; *Weaver & Rose, 1967*; *Seidel & Smith, 1986*; *Seidel & Jackson, 1990*, 5; *Stephens & Wiens, 2003*, 78; Added character state and Altered wording): 0 = absent, surface nearly flat; 1 = mixture; 2 = present, raised, separate from and lateral to lingual ridge.

CHARACTER 91: Dentary L

**Character definition**. Anterior midline of the alveolar surface (*Seidel, 1988*, 15): 0 = discontinuous with ventral median portion of the dentary set apart as a ledge or shelf; 1 = continuous with the ventral median portion of the dentary, not set apart as a ledge or shelf.

CHARACTER 92: Dentary M

**Character definition**. Median symphysial ridge on lower alveolar surface (*Weaver & Rose, 1967*): 0 = absent; 1 = present.

**Angular**

CHARACTER 93: Angular A

**Character definition**. Dorsal projection of angular (*McDowell, 1964*, 1; *Gaffney & Meylan, 1988*; *Stephens & Wiens, 2003*, 79; Added character state and Altered wording): 0 = contacts; 1 = mixture; 2 = does not contact Meckel’s cartilage (separated by the prearticular, but does contact articular).

CHARACTER 94: Angular B

**Character definition**. Angular bone (*McDowell, 1964*, 6): 0 = reduced in length and conspicuously exceeded in anterior extent by prearticular; 1 = unreduced in length, equals or exceeds prearticular in anterior extent.

**Coronoid**

CHARACTER 95: Coronoid A

**Character definition**. Processus coronoideus (*Bertl & Killebrew*, *1983*; *Stephens & Wiens, 2003*, 80; Added character state): 0 = not hooked; 1 = mixture; 2 = hooked.

**Jaws**

CHARACTER 96: Jaws A

**Character definition**. Tuberculate denticles on alveolar surface (*Seidel & Smith, 1986*; *Seidel & Jackson, 1990*, 4): 0 = inconspicuous or absent; 1 = prominent.

**Cervical Vertebra**

CHARACTER 97: Cervical Vertebra A

**Character definition**. Cervical vertebrae (*McDowell, 1964*; *Stephens & Wiens, 2003*, 117; Added character state): 0 = not elongate; 1 = mixture; 2 = elongate.

CHARACTER 98: Cervical Vertebra B

**Character definition**. Articulation of cervical vertebrae V and VI (*McDowell, 1964*, 3; *Gaffney & Meylan, 1988*; *Stephens & Wiens, 2003*, 119; Added character state): 0 = double; 1 = mixture; 2 = single.

CHARACTER 99: Cervical Vertebra C

**Character definition**. Cervical vertebra VIII (*McDowell, 1964*; *Stephens & Wiens, 2003*, 118; Added character state): 0 = shorter than cervical vertebrae II–VII; 1 = mixture; 2 = cervical vertebrae all equal in length.

CHARACTER 100: Cervical Vertebra D

**Character definition**. Cervical vertebra IX (*Adler*, *1968*; Modified wording): 0 = haemal spine projects ventrally, with distal end approximately directly ventral to midpoint between connection of haemal spine and vertebral centrum; 1 = haemal spine projects anteroventrally, with distal end anterior to midpoint at connection between haemel spine and vertebral centrum.

**Ribs**

CHARACTER 101: Ribs A

**Character definition**. Thoracic rib heads (*McDowell, 1964*; *Stephens & Wiens, 2003*, 120): 0 = straight, relatively short and thick; 1 = long, slender, and bowed ventrally; 2 = long and slender but not bowed ventrally.

CHARACTER 102: Ribs B

**Character definition**. Furthest lateral rib projections (free rib segment) from neurals (*Adler*, *1968*; Modified wording): 0 = rib III; 1 = rib II.

**Scapula**

CHARACTER 103: Scapula A

**Character definition**. Suprascapula (*Bojanus*, *1819*; *White*, *1929*; *Bramble*, 1974; *Burke et al., 1996*, 11; *Stephens & Wiens, 2003*, 115; Added character state): 0 = absent; 1 = mixture; 2 = present.

CHARACTER 104: Scapula B

**Character definition**. Episcapula (*White*, *1929*; *Bramble*, *1974*; *Burke et al., 1996*, 12; *Stephens & Wiens, 2003*, 116; Added character state): 0 = absent; 1 = mixture; 2 = present.

CHARACTER 105: Scapula C

**Character definition**. Length of acromion process of scapula (*Minx, 1996*, SC; *Stephens & Wiens, 2003*, 13; Provided distinct character states): 0 = shorter than length of scapula; 1 = approximately equal in length; 2 = longer than length of scapula.

**Pelvis**

CHARACTER 106: Pelvis A

**Character definition**. Opening in pelvis (*Stephens & Wiens, 2003*, 122; Added character state): 0 = single; 1 = mixture; 2 = two openings present, anterior and posterior halves of pelvis contact medially.

CHARACTER 107: Pelvis B

**Character definition**. Sutures between right and left halves of pelvis (*Stephens & Wiens, 2003*, 123; Added character state): 0 = visible; 1 = mixture; 2 = right and left half of pelvis completely fused, sutures no longer visible ventrally.

**Pubes**

CHARACTER 108: Pubes A

**Character definition**. Epipubes (*Gaffney & Meylan, 1988*; *Stephens & Wiens, 2003*, 121; Added character state): 0 = not ossified, cartilaginous; 1 = mixture; 2 = at least partially ossified.

**Manus**

CHARACTER 109: Manus A

**Character definition**. Number of phalanges of manus digit I (*Stephens & Wiens, 2003*, 105; Added character state and Provided distinct character states): 0 = 2 or less; 1 = mixture; 2 = 3 or more.

CHARACTER 110: Manus B

**Character definition**. Number of phalanges of manus digit II (*Stephens & Wiens, 2003*, 106; Added character state and Provided distinct character states): 0 = 2 or less; 1 = mixture; 2 = 3 or more.

CHARACTER 111: Manus C

**Character definition**. Number of phalanges of manus digit III (*Stephens & Wiens, 2003*, 107; Added character state and Provided distinct character states): 0 = 2 or less; 1 = mixture; 2 = 3 or more.

CHARACTER 112: Manus D

**Character definition**. Number of phalanges of manus digit IV (*Stephens & Wiens, 2003*, 108; Added character state and Provided distinct character states): 0 = 2 or less; 1 = mixture; 2 = 3 or more.

CHARACTER 113: Manus E

**Character definition**. Number of phalanges of manus digit V (*Stephens & Wiens, 2003*, 109; Added character state and Provided distinct character states): 0 = 2 or less; 1 = mixture; 2 = 3 or more.

**Pes**

CHARACTER 114: Pes A

**Character definition**. Number of phalanges of pes digit I (*Stephens & Wiens, 2003*, 110; Added character state and Provided distinct character states): 0 = 2 or less; 1 = mixture; 2 = 3 or more.

CHARACTER 115: Pes B

**Character definition**. Number of phalanges of pes digit II (*Stephens & Wiens, 2003*, 111; Added character state and Provided distinct character states): 0 = 2 or less; 1 = mixture; 2 = 3 or more.

CHARACTER 116: Pes C

**Character definition**. Number of phalanges of pes digit III (*Stephens & Wiens, 2003*, 112; Added character state and Provided distinct character states): 0 = 2 or less; 1 = mixture; 2 = 3 or more.

CHARACTER 117: Pes D

**Character definition**. Number of phalanges of pes digit IV (*Stephens & Wiens, 2003*, 113; Added character state and Provided distinct character states): 0 = 2 or less; 1 = mixture; 2 = 3 or more.

CHARACTER 118: Pes E

**Character definition**. Number of phalanges of pes digit V (*Stephens & Wiens, 2003*, 114; Added character state and Provided distinct character states): 0 = 2 or less; 1 = mixture; 2 = 3 or more.

**Phalanges**

CHARACTER 119: Phalanges A

**Character definition**. Fifth digit (*Seidel & Smith, 1986*; *Seidel & Jackson, 1990*; 18): 0 = never more than three phalanges present; 1 = can have more than three phalanges present.

**Shell**

CHARACTER 120: Shell A

**Character definition**. Plastron length/carapace length (*Seidel, 2002*, A): 0 = less than or equal to 0.89; 1 = greater than 0.89.

CHARACTER 121: Shell B

**Character definition**. Carapace-plastral connection (*Burke et al., 1996*, 14; *Stephens & Wiens, 2003*, 81; Added character state and Reordered characters for basal versus derived states): 0 = bony; 1 = mixture; 2 = ligamentous.

**Carapace**

CHARACTER 122: Carapace A

**Character definition**. Sexual size dimorphism (*Seidel & Smith, 1986*; *Gibbons & Lovich*, *1990*; *Seidel & Jackson, 1990*, 17; *Burke et al., 1996*, 37; Modified and Reordered character states): 0 = adult females tend to have greater carapace lengths than males, but less than 2x that of the males; 1 = adult females often larger than 2x the size of the males; 2 = adult males and females with approximately the same carapace lengths; 3 = adult males tend to have greater carapace lengths. Character states 1 and 2 were switched from *Burke et al*. (1996) to make scoring ‘linear’.

CHARACTER 123: Carapace B

**Character definition**. Maximum female carapace length (*Seidel & Smith, 1986*; *Seidel, 1988*, 16; *Seidel & Jackson, 1990*; 16; *Seidel, 2002*, G; Modified values for character states): 0 = less than or equal to 250 mm; 1 = between 250 mm and 400 mm; 2 = greater than or equal to 400 mm. This size is considered the maximum size for the turtles, so if a specimen is not sexed, then a maximum size can be used for this character, but not for the maximum size of males (Character 124: Carapace C).

CHARACTER 124: Carapace C

**Character definition**. Old adult males (*Seidel, 1988*, 17): 0 = frequently less than 200 mm carapace length; 1 = frequently larger than 200 mm carapace length.

CHARACTER 125: Carapace D

**Character definition**. Adult body size (*Ernst & Barbour*, *1989*; *Burke et al., 1996*, 4; Altered character states): 0 = approximately greater than or equal to (≥) 140 mm carapace length; 1 = approximately less than (<) 140 mm carapace length.

CHARACTER 126: Carapace E

**Character definition**. Overall shape of carapace, in dorsal external view (*Minx*, *1996*, EC; *Stephens & Wiens, 2003*, 156): 0 = circular; 1 = oval, sometimes expanded posteriorly, with lateral edges around bridge parallel to sub-parallel; 2 = with slightly concave lateral edges.

CHARACTER 127: Carapace F

**Character definition**. Carapace shape in lateral view (*Galbreath*, *1948*; *Seidel & Inchaustegui Miranda*, *1984*; *Stephens & Wiens, 2003*, 157; Added character state): 0 = domed, having distinct ‘highest point’; 1 = mixture; 2 = flattened, no distinct highest point.

CHARACTER 128: Carapace G

**Character definition**. Location of highest point of carapace, when present (*Stephens & Wiens, 2003*, 158): 0 = anterior to midline; 1 = at midline; 2 = posterior to midline.

CHARACTER 129: Carapace H

**Character definition**. Carapace dorsal surface texture (*Galbreath*, *1948*; *Weaver & Rose, 1967*; *Burke et al., 1996*, 7; Reworded): 0 = smooth or with smooth contours; 1 = rugose, with depressions and/or elevations.

CHARACTER 130: Carapace I

**Character definition**. Anterior margin of carapace (NEW): 0 = not serrated/indented; 1 = singly serrated/indented (usually at the distal end of sulcus between marginals); 2 = doubly serrated/indented (at distal end of sulcus between marginals and at sutures between peripherals).

CHARACTER 131: Carapace J

**Character definition**. Median keel along dorsal midline of carapace (*Galbreath*, *1948*; *Seidel & Smith, 1986*; *Seidel & Jackson, 1990*, 10; *Burke et al., 1996*, 5; *Stephens & Wiens, 2003*, 174; Added character state): 0 = absent; 1 = mixture; 2 = present.

CHARACTER 132: Carapace K

**Character definition**. Keel, when present (*Stephens & Wiens, 2003*, 175): 0 = primarily located on anterior half of carapace; 1 = distributed equally on anterior and posterior halves of carapace; 2 = primarily located on posterior half of carapace.

CHARACTER 133: Carapace L

**Character definition**. Keel, when present, consists of (*Stephens & Wiens, 2003*, 176): 0 = single ridge; 1 = apically blunt knobs; 2 = apically acute serrations.

CHARACTER 134: Carapace M

**Character definition**. Posterior edge of carapace (*Adler*, *1968*; Modified wording): 0 = not serrated/indented; 1 = singly serrated/indented (usually at the distal end of sulcus between marginals); 2 = doubly serrated/indented (at distal end of sulcus between marginals and at sutures between peripherals).

CHARACTER 135: Carapace N

**Character definition**. Carapace (*Seidel, 1988*, 18): 0 = not flared posteriorly, width at marginal sulcus VII–VIII usually less than 75% of the carapace length; 1 = flared posteriorly, width at marginal sulcus VII–VIII usually greater than 75% of the carapace length.

**Nuchal**

CHARACTER 136: Nuchal A

**Character definition**. Nuchal dorsal surface (*Galbreath, 1948*; *Weaver & Rose, 1967*; Modified wording): 0 = smooth; 1 = sculptured, with ridges.

CHARACTER 137: Nuchal B

**Character definition**. Cervical scute region of nuchal (NEW): 0 = smooth; 1 = rugose.

CHARACTER 138: Nuchal C

**Character definition**. Marginal scute region of nuchal (*Weaver & Robertson, 1967*; Modified wording): 0 = smooth; 1 = anterior smooth; 2 = rugose.

CHARACTER 139: Nuchal D

**Character definition**. Pleural scute region of nuchal (*Weaver & Robertson, 1967*; Modified wording): 0 = smooth; 1 = rugose.

CHARACTER 140: Nuchal E

**Character definition**. Vertebral scute region of nuchal (NEW): 0 = smooth; 1 = rugose.

CHARACTER 141: Nuchal F

**Character definition**. Nuchal with broad shallow notch at anterior end, between first marginals (*Galbreath, 1948*; *Weaver & Robertson, 1967*; Modified wording): 0 = absent, not notched, or insignificant; 1 = shallow notched; 2 = deeply notched.

CHARACTER 142: Nuchal G

**Character definition**. The region of the nuchal under the cervical scute (NEW): 0 = does not project anterior to the anterior-most point of the first marginal region; 1 = does project anterior to the anterior-most point of the first marginal region.

CHARACTER 143: Nuchal H

**Character definition**. The region of the nuchal under vertebral 1 (NEW): 0 = anterior margin wider than posterior margin; 1 = anterior and posterior margins roughly equal or anterior narrower than posterior.

CHARACTER 144: Nuchal I

**Character definition**. The anterior edge of the region of the nuchal under vertebral 1 (NEW): 0 = with roughly flat anterior edge, or gently convexly-curved; 1 = projects anteromedially between the region of marginals 1, also leading to a shortened (anteroposteriorly) cervical (or cervical region).

CHARACTER 145: Nuchal J

**Character definition**. Anteroposterior length of nuchal under first marginal (NEW): 0 = less than (<), to perhaps equal to, the length from posterior margin to vertebral 1 – first pleural – first marginal point; 1 = same length or longer (≥) than. If only equal to, then scored as (0,1). This character may end up being separated into a third character state to better show variations when the two values are close to equal.

CHARACTER 146: Nuchal K

**Character definition**. Nuchal (*Adler, 1968*; *Seidel & Jackson, 1990*, 14; Added character state and Modified): 0 = Not overlapped by pleural 1; 1 = barely overlapped by pleural 1; 2 = broad overlap by pleural 1.

**Neural**

CHARACTER 147: Neural A

**Character definition**. Number of sides of neural I (*Minx, 1996*, NC; *Stephens & Wiens, 2003*, 84; Provided discrete character states): 0 = 5 or less; 1 = 6 (hexagonal); 2 = 7 or more.

CHARACTER 148: Neural B

**Character definition**. Number of sides of neural II (*Minx, 1996*, NC; *Stephens & Wiens, 2003*, 85; Provided discrete character states): 0 = 5 or less; 1 = 6 (hexagonal); 2 = 7 or more.

CHARACTER 149: Neural C

**Character definition**. Number of sides of neural III (*Minx, 1996*, NC; *Stephens & Wiens, 2003*, 86; Provided discrete character states): 0 = 5 or less; 1 = 6 (hexagonal); 2 = 7 or more.

CHARACTER 150: Neural D

**Character definition**. Number of sides of neural IV (*Minx, 1996*, NC; *Stephens & Wiens, 2003*, 87; Provided discrete character states): 0 = 5 or less; 1 = 6 (hexagonal); 2 = 7 or more.

CHARACTER 151: Neural E

**Character definition**. Number of sides of neural V (*Minx, 1996*, NC; *Stephens & Wiens, 2003*, 88; Provided discrete character states): 0 = 5 or less; 1 = 6 (hexagonal); 2 = 7 or more.

CHARACTER 152: Neural F

**Character definition**. Number of sides of neural VI (*Minx, 1996*, NC; *Stephens & Wiens, 2003*, 89; Provided discrete character states): 0 = 5 or less; 1 = 6 (hexagonal); 2 = 7 or more.

CHARACTER 153: Neural G

**Character definition**. Neural VII (*Stephens & Wiens, 2003*, 90; Added character state): 0 = absent; 1 = mixture; 2 = present.

CHARACTER 154: Neural H

**Character definition**. Number of sides of neural VII (*Minx, 1996*, NC; *Stephens & Wiens, 2003*, 91; Provided discrete character states): 0 = 5 or less; 1 = 6 (hexagonal); 2 = 7 or more.

CHARACTER 155: Neural I

**Character definition**. Neural VIII (*Stephens & Wiens, 2003*, 92; Added character state): 0 = absent; 1 = mixture; 2 = present.

CHARACTER 156: Neural J

**Character definition**. Number of sides of neural VIII (*Minx, 1996*, NC; *Stephens & Wiens, 2003*, 93; Provided discrete character states): 0 = 5 or less; 1 = 6 (hexagonal); 2 = 7 or more.

**Suprapygal**

CHARACTER 157: Suprapygal A

**Character definition**. Number of suprapygals (*Stephens & Wiens, 2003*, 97; Provided discrete character states): 0 = 0; 1 = 1; 2 = 2; 3 = 3.

CHARACTER 158: Suprapygal B

**Character definition**. Shape of anterior-most suprapygal (NEW): 0 = shortened; 1 = elongate. Note this character scored as unknown (‘?’) if less than two suprapygals present.

CHARACTER 159: Suprapygal C

**Character definition**. Number of sides of posterior-most suprapygal (*Minx, 1996*, SP; *Stephens & Wiens, 2003*, 95; Provided discrete character states): 0 = 5 or less; 1 = 6 (hexagonal); 2 = 7 or more. Note: To get 6 or more sides, there must be distinct angles present, commonly on the posterior of this suprapygal. To get more than 6 sides, commonly the anterior suprapygal (or posterior-most neural) is posteriorly-angled into the anterior edge of the posterior suprapygal and/or peripherals XI form sharp angles toward its anterolateral edges. Note this character scored as unknown (‘?’) is no suprapygals present.

CHARACTER 160: Suprapygal D

**Character definition**. Suprapygals (*Stephens & Wiens, 2003*, 96; Added character state): 0 = separated from neurals by last pair of costals; 1 = mixture; 2 = contacting neurals.

**Pygal**

CHARACTER 161: Pygal A

**Character definition**. Ventral projection of pygal bone (*Weaver & Rose, 1967*): 0 = absent; 1 = present and acute.

CHARACTER 162: Pygal B

**Character definition**. Pygal (*Hay*, *1908*; *Seidel, 1988*, 24; *Seidel, 2002*, I; Added character state and Modified wording): 0 = does not extend beyond marginal-vertebral sulcus; 1 = extends beyond marginal-vertebral sulcus, but not for its entire width; 2 = extends beyond marginal-vertebral sulcus for its entire width.

CHARACTER 163: Pygal C

**Character definition**. Anterior margin of pygal (NEW): 0 = concave posteriorly; 1 = flat.

CHARACTER 164: Pygal D

**Character definition**. Lateral edges of pygal (NEW): 0 = parallel; 1 = posterior edge wider; 2 = anterior edge wider.

CHARACTER 165: Pygal E

**Character definition**. Posterior notch of pygal (NEW): 0 = absent; 1 = slight notch; 2 = pronounced or deeply notched.

NOTE: For medial depression of pygal, see Character Marginal O (Character 189).

**Costal**

CHARACTER 166: Costal A

**Character definition**. Ribs on proximal end of costal (*Galbreath, 1948*; Modified wording): 0 = not prominent; 1 = prominent.

CHARACTER 167: Costal B

**Character definition**. Longitudinal rugosities of costals (*Adler, 1968*; *Seidel & Smith, 1986*; *Seidel & Jackson, 1990*, 11; Modified wording): 0 = absent (or in less than 10% of individuals); 1 = present (or in at least 90% of individuals).

CHARACTER 168: Costal C

**Character definition**. Costal dorsal surface (*Galbreath, 1948*; Modified wording): 0 = with ≤ 6 ridges on distal edge; 1 = between 7–8 ridges on distal edge, rugosity on proximal portion; 2 = with ≥ 9 ridges (covered with ridges).

CHARACTER 169: Costal D

**Character definition**. Inward depression in the posterior half of the fourth costal (*Minx, 1996*, PB; *Stephens & Wiens, 2003*, 94; Added character state): 0 = absent; 1 = mixture; 2 = present.

**Peripheral**

CHARACTER 170: Peripheral A

**Character definition**. Peripheral bone notching at lateral edges (*Weaver & Rose, 1967*; *Seidel & Smith, 1986*; *Seidel & Jackson, 1990*, 12; *Stephens & Wiens, 2003*, 168; Modified to include all peripherals rather than just posterior peripherals): 0 = not notched; 1 = somewhat notched; 2 = pronounced notching.

CHARACTER 171: Peripheral B

**Character definition**. Peripherals (*Minx, 1996*, TP; *Stephens & Wiens, 2003*, 83; Added character state): 0 = not thickened; 1 = mixture; 2 = with lateral edges swollen to form lip.

**Cervical**

CHARACTER 172: Cervical A

**Character definition**. Cervical (= nuchal scute) underlap (ventral length)/carapace length (*Weaver & Rose, 1967*; *Seidel, 2002*, B; Modified character states): 0 = greater than 0.055; 1 = between (or equal to) 0.037–0.055; 2 = less than 0.037.

CHARACTER 173: Cervical B

**Character definition**. Cervical scute (= nuchal scute) dorsal length (*Seidel, 1988*, 22): 0 = long, dorsal length more than 7.3% of the carapace length; 1 = short, dorsal length less than 7.3% of the carapace length.

CHARACTER 174: Cervical C

**Character definition**. Cervical scute (= nuchal scute) (NEW): 0 = widest along its posterior edge; 1 = widest approximately midway through scute; 2 = approximately parallel along its length (at least along the length where it contacts the first marginal).

CHARACTER 175: Cervical D

**Character definition**. Cervical scute (= nuchal scute) shape, in dorsal view (NEW): 0 = longer (anteroposteriorly) than broad (mediolaterally); 1 = approximately even sides (square); 2 = broader than long.

**Vertebral**

CHARACTER 176: Vertebral A

**Character definition**. Vertebral 1 anterior width/carapace length (*Seidel, 1988*, 23; *Seidel, 2002*, C; Modified character states): 0 = less than 0.150; 1 = between (or equal to) 0.150–0.175; 2 = greater than 0.175.

CHARACTER 177: Vertebral B

**Character definition**. Anterior and posterior widths of vertebral 1 and vertebral 2 (*Galbreath, 1948*; Added and Modified character states): 0 = vertebral 1 approximately equal to width of vertebral 2, or vertebral 1 wider than vertebral 2; 1 = vertebral 1 less than width of vertebral 2; 2 = anterior width of vertebral 1 less than posterior width of vertebral 1 and widths of vertebral 2, while posterior width of vertebral 1 roughly equal to widths of vertebral 2; 3 = anterior width of vertebral 1 wider than posterior width of vertebral 1 and width of vertebral 2.

CHARACTER 178: Vertebral C

**Character definition**. Vertebral 1 (*Galbreath, 1948*; *Seidel & Smith, 1986*; *Seidel & Jackson, 1990*, 13; *Seidel, 1994*; *Stephens & Wiens, 2003*, 179; Added and Modified character state): 0 = not constricted, edges relatively straight; 1 = constricted anteriorly; 2 = constricted at mid-length, forming hour-glass shape; 3 = not constricted with posterior border narrower than anterior border.

CHARACTER 179: Vertebral D

**Character definition**. Anterolateral border of vertebral 1 (*Seidel & Jackson, 1990*, 15): 0 = not confined to nuchal; 1 = confined to nuchal.

**Marginal**

CHARACTER 180: Marginal A

**Character definition**. Diagonal rugose lines or ridges on marginal dorsal surface (*Galbreath, 1948*; *Weaver & Robertson, 1967*; Modified wording): 0 = absent; 1 = present on lateral half only; 2 = present over whole region.

CHARACTER 181: Marginal B

**Character definition**. First marginal (*Minx, 1996*, MS; *Stephens & Wiens, 2003*, 180): 0 = long and narrow, maximum length exceeds maximum width; 1 = square, width and length roughly equal; 2 = short and wide, width exceeds length.

CHARACTER 182: Marginal C

**Character definition**. Anterior marginals of carapace (*Stephens & Wiens, 2003*, 164; Added character state): 0 = not serrate; 1 – mixture; 2 = serrate.

CHARACTER 183: Marginal D

**Character definition**. Number of most posterior marginal bearing a notched distal border among marginals anterior of bridge. Marginals were numbered from anterior to posterior, following *Zangerl* (*1969*) (*Stephens & Wiens, 2003*, 167; Provided discrete character states): 0 = absent or only notching present between cervical and marginal 1; 1 = between marginal 1 and marginal 2; 2 = between marginal 2 and marginal 3; 3 = between marginal 3 and marginal 4; 4 = between marginal 4 and marginal 5.

CHARACTER 184: Marginal E

**Character definition**. Posterolateral marginal serrations (*Galbreath, 1948*; Modified wording): 0 = not present; 1 = present.

CHARACTER 185: Marginal F

**Character definition**. Posterior marginal serrations (*Galbreath, 1948*; Modified wording): 0 = absent; 1 = present.

CHARACTER 186: Marginal G

**Character definition**. Posterior-most marginal (*Stephens & Wiens, 2003*, 181; Added character state): 0 = form smooth horizontal line with marginals lateral to them; 1 = mixture; 2 = higher than marginals just lateral to them.

CHARACTER 187: Marginal H

**Character definition**. Orientation of edge of posterior row of marginal (*Stephens & Wiens, 2003*, 182; Added character state): 0 = posteroventral, not flared; 1 = mixture; 2 = flared posteriorly or posterodorsally out and up to form lip.

CHARACTER 188: Marginal I

**Character definition**. Marginal or marginals contacted by sulcus A, sulcus between vertebral I and pleural scute I (*Tinkle*, *1962*; *Burke et al., 1996*, 8; *Stephens & Wiens, 2003*, 183; Provided discrete character states): 0 = anterior to marginal 1 or anterior half of marginal 1; 1 = middle of marginal 1; 2 = posterior half of marginal 1 or to the sulcus between marginal 1 and marginal 2; 3 = completely on marginal 2.

CHARACTER 189: Marginal J

**Character definition**. Marginal or marginals contacted by sulcus B, sulcus between pleural scutes I and II (*Tinkle*, *1962*; *Stephens & Wiens, 2003*, 184; Provided discrete character states): 0 = marginal 4 or sulcus between marginal 4 and marginal 5; 1 = marginal 5; 2 = sulcus of marginal 5 and marginal 6 or posterior to marginal 5.

CHARACTER 190: Marginal K

**Character definition**. Marginal or marginals contacted by sulcus C, sulcus between pleural scutes II and III (*Tinkle*, 1962; *Stephens & Wiens, 2003*, 185; Provided discrete character states): 0 = marginal 6; 1 = anterior or middle of marginal 7; 2 = posterior of or posterior to marginal 7.

CHARACTER 191: Marginal L

**Character definition**. Marginal or marginals contacted by sulcus D, sulcus between pleural scutes III and IV (*Tinkle*, *1962*; *Stephens & Wiens, 2003*, 186; Provided discrete character states): 0 = marginal 8; 1 = sulcus between marginal 8 and marginal 9, or anterior portion of marginal 9; 2 = middle of marginal 9 and more posteriorly.

CHARACTER 192: Marginal M

**Character definition**. Marginal or marginals contacted by sulcus E, sulcus between pleural scutes IV and V (*Tinkle*, *1962*; *Stephens & Wiens, 2003*, 187; Provided discrete character states): 0 = anterior to or at sulcus between marginal 10 and marginal 11; 1 = anterior to middle of marginal 11; 2 = posterior of or posterior to marginal 11.

CHARACTER 193: Marginal N

**Character definition**. Number of marginals on each side of carapace (*Tinkle*, *1962*; *Stephens & Wiens, 2003*, 188; Provided discrete character states): 0 = 11; 1 = 12; 2 = 13. Note that this assumes the two posterior-most scutes are supracaudals and not marginals.

CHARACTER 194: Marginal O

**Character definition**. Depression between 12th marginals, also known as the supracaudals, (*Minx, 1996*, IM; Modified wording): 0 = absent; 1 = present.

CHARACTER 195: Marginal P

**Character definition**. Supracaudal (posterior-most marginal) scutes (*McDowell, 1964*, 4): 0 = extend forward onto suprapygal; 1 = fall short of suture between pygal and suprapygal.

**Supracaudal**

See posterior-most marginal characters (Characters 189–190).

**Plastron**

CHARACTER 196: Plastron A

**Character definition**. Plastron (*Weaver & Rose, 1967*): 0 = smooth; 1 = rugose.

CHARACTER 197: Plastron B

**Character definition**. Number of plastral hinges (*Stephens & Wiens, 2003*, 189): 0 = none; 1 = one; 2 = two. It is noted that in all specimens that bore a single plastral hinge, the hinge was present in the anterior half of the carapace (*Stephens & Wiens, 2003*).

CHARACTER 198: Plastron C

**Character definition**. Anterior plastron (*Burke et al., 1996*, 16; *Stephens & Wiens, 2003*, 191): 0 = not closeable; 1 = closeable.

CHARACTER 199: Plastron D

**Character definition**. Posterior plastron (*Burke et al., 1996*, 15; *Stephens & Wiens, 2003*, 190): 0 = not closeable; 1 = closeable.

CHARACTER 200: Plastron E

**Character definition**. Male plastron (*Minx, 1996*, CP; *Stephens & Wiens, 2003*, 198): 0 = flat; 1 = bearing concavity.

CHARACTER 201: Plastron F

**Character definition**. Concavity of male plastron, when present (*Minx, 1996*, CP; *Stephens & Wiens, 2003*, 199; Added character state): 0 = restricted to posterior half of plastron; 1 = mixture; 2 = extends along entire length of plastron.

CHARACTER 202: Plastron G

**Character definition**. Anterior edge of plastron, in dorsal view (NEW): 0 = does not extend beyond anterior edge of carapace; 1 = even with, or extends beyond, anterior edge of carapace.

CHARACTER 203: Plastron H

**Character definition**. Anterior margin of plastron (*Stephens & Wiens, 2003*, 206; Added character state): 0 = not serrate; 1 = mixture; 2 = serrate.

CHARACTER 204: Plastron I

**Character definition**. Posterior margin of plastron (*Stephens & Wiens, 2003*, 207; Added character state): 0 = not serrate; 1 = mixture; 2 = serrate.

CHARACTER 205: Plastron J

**Character definition**. Anterior plastral lobe (NEW): 0 = not inflated laterally, lateral sides approximately parallel; 1 = inflated laterally.

CHARACTER 206: Plastron K

**Character definition**. Posterior plastral lobe (NEW): 0 = not inflated laterally, lateral sides approximately parallel; 1 = inflated laterally.

CHARACTER 207: Plastron L

**Character definition**. Cervico-plastral ligament (*Bramble*, *1974*; *Burke et al., 1996*, 13): 0 = absent; 1 = present.

CHARACTER 208: Plastron M

**Character definition**. Widest location of the posterior plastral lobe (*Galbreath, 1948*; *Minx, 1996*, WP; Modified wording): 0 = anteriorly toward the inguinal buttresses; 1 = medially, around the femoral scutes or the femural scutes are as wide as the anterior near the inguinal buttresses; 2 = posteriorly, at or posterior to the femoral-anal sulcus.

**Epiplastron**

CHARACTER 209: Epiplastron A

**Character definition**. Anterior epiplastral margin (*Weaver & Rose, 1967*; *Seidel, 1994*; *Stephens & Wiens, 2003*, 98) 0 = underlying gular scutes: not swollen, short and inconspicuous or absent; 1 = mixture; 2 = swollen to form lip, wide and thick or pronounced.

CHARACTER 210: Epiplastron B

**Character definition**. Epiplastra (*Stephens & Wiens, 2003*, 99; Added character state and Reworded): 0 = with smooth anterolateral margins; 1 = mixture; 2 = each bearing a tooth-like swelling on dorsal surface at margin of gular and humeral scutes.

CHARACTER 211: Epiplastron C

**Character definition**. Anterior epiplastral margin, in ventral view (*Seidel*, *1994*; *Stephens & Wiens, 2003*, 100): 0 = straight; 1 = curved anteromedially and usually forming smooth, slightly convexly-curved line with rest of epiplastral margin; 2 = curved and bearing shallow medial cleft, having an appearance similar to the top of a ‘heart’ symbol.

CHARACTER 212: Epiplastron D

**Character definition**. Anterior epiplastral margin underlying gular scutes, in anterior view (*Stephens & Wiens, 2003*, 101; Added character state): 0 = straight and flat; 1 = mixture; 2 = curved dorsally at margins.

CHARACTER 213: Epiplastron E

**Character definition**. Epiplastron (NEW): 0 = anterior and medial edges form right angle; 1 = do not form right angle.

**Entoplastron**

CHARACTER 214: Entoplastron A

**Character definition**. Entoplastron (*Stephens & Wiens, 2003*, 102): 0 = absent; 1 = present.

CHARACTER 215: Entoplastron B

**Character definition**. Entoplastron (*Jackson*, *1988*; *Seidel, 2002*, W; Reworded and Reordered characters for basal versus derived states): 0 = elongate, longer (anteroposteriorly) than broad (mediolaterally); 1 = roughly equally broad (mediolaterally) as long (anteroposteriorly); 2 = broader (mediolaterally) than long (anteroposteriorly).

CHARACTER 216: Entoplastron C

**Character definition**. Number of sides of entoplastron, in ventral view (*Seidel & Inchaustegui Miranda*, *1984*; *Stephens & Wiens, 2003*, 103; Provided discrete character states): 0 = ≤ 5; 1 = 6–7; 2 = ≥ 8.

CHARACTER 217: Entoplastron D

**Character definition**. Entoplastron (*Stephens & Wiens, 2003*, 104): 0 = extended anteriorly, majority of element is anterior to point of greatest width; 1 = anterior and posterior halves of entoplastron equal; 2 = entoplastron extended posteriorly, majority of element posterior to point of greatest width.

CHARACTER 218: Entoplastron E

**Character definition**. Entoplastron: 0 = does not project at humeral-gular sulci, keeping a gently angled edge; 1 = projects anteriorly at humeral-gular sulcus (anteroposteriorly), creating a pronounced, sharp angle.

**Hypoplastron**

CHARACTER 219: Hypoplastron A

**Character definition**. Indent at lateral edge of plastron at the hypoplastron-xiphiplastron sutural contact (NEW): 0 = absent; 1 = present.

**Xiphiplastron**

CHARACTER 220: Xiphiplastron A

**Character definition**. Xiphiplastron (*Weaver & Rose, 1967*; Modified): 0 = expanded; 1 = abbreviated. *Weaver & Rose* (*1967*) defined xiphiplastron width as the width at the hypoplastral-xiphiplastral suture divided by the anteroposterior length from the posterior edge of the plastron to the posterior border of the abdominals (along the midline). For this character, *Weaver & Rose* (*1967*) seem to have used values above 1.35 to be ‘expanded’, and those below 1.35 to be ‘abbreviated’. These are the values used in this study to check old scores and score new taxa and specimens.

CHARACTER 221: Xiphiplastron B

**Character definition**. Posteromedial margin of plastron, anal notch of xiphiplastra (*Weaver & Rose, 1967*; *Adler, 1968*; *Seidel, 1994*; *Stephens & Wiens, 2003*, 205; Reworded and Reordered characters for basal versus derived states): 0 = forms a horizontal line, may have tiny, inconspicuous median notch present; 1 = curved anteromedially, 2 = consists of deep ‘V-shaped’ indentation between posterior anals; 3 = rounded posteriorly (convexly-curved).

**Bridge**

CHARACTER 222: Bridge A

**Character definition**. Bridge of plastron, a dorsal extension of the plastron that is visible externally and contacts the carapace (*Stephens & Wiens, 2003*, 216; Added character state): 0 = absent; 1 = mixture; 2 = present. Note that when the plastral bridge is absent, reduced plastral buttresses may or may not be present internally (*Stephens & Wiens, 2003*).

CHARACTER 223: Bridge B

**Character definition**. Plastral buttresses (*Burke et al., 1996*, 17; *Stephens & Wiens, 2003*, 82): 0 = absent; 1 = present, with axillary and inguinal buttresses (dorsal processes of plastron) contacting carapace.

CHARACTER 224: Bridge C

**Character definition**. Plastral buttresses, if present (*McDowell, 1964*, 15; *Burke et al., 1996*. 17; Modified wording and Reordered character states): 0 = rather weak, with the axillary buttress extending only a short distance onto the first costal, the inguinal buttress extending a short distance onto the fifth costal; 1 = relatively strong, the axillary buttress extending well on the first costal, the inguinal buttress extending well on to fifth costal, sometimes contacting sixth costal.

CHARACTER 225: Bridge D

**Character definition**. Musk glands (*Waagen*, *1972*; *Burke et al., 1996*, 20): 0 = one pair of axillary and one pair of inguinal glands; 1 = one pair of axillary glands only; 2 = no glands.

**Gular**

CHARACTER 226: Gular A

**Character definition**. Gular overlap (*Weaver & Rose, 1967*): 0 = short; 1 = long.

CHARACTER 227: Gular B

**Character definition**. Gulars, in ventral view (*Stephens & Wiens, 2003*, 200; Added character state): 0 = flush with anterior margin of epiplastra; 1 = mixture; 2 = extend anteriorly to margin of epiplastra. *Stephens & Wiens* (*2003*) had mistakenly listed them as humerals rather than epiplastra.

CHARACTER 228: Gular C

**Character definition**. Gulars width (*Seidel, 1988*, 20; Reworded): 0 = narrow, plastron width at gular-humeral sulcus less than or equal to 23% of carapace length; 1 = broad, plastron width at gular-humeral sulcus greater than 23% of the carapace length. Note width is measured at anterior edge of gulars.

CHARACTER 229: Gular D

**Character definition**. Gular length (*Seidel, 1988*, 21; Reworded): 0 = gulars long, median length greater than or equal to 15% of carapace length; 1 = scutes short, median length less than 15% of carapace length. Note, length is measured down gular-gular sulcus.

NOTE: When talking about sulci between two different plastral scutes (i.e. humeral and pectoral), the character is listed with the more anterior scute.

**Humeral**

CHARACTER 230: Humeral A

**Character definition**. Humoral-pectoral sulcus (*Weaver & Rose, 1967*; *Gaffney & Meylan, 1988*; *Stephens & Wiens, 2003*, 201; Added character state): 0 = does not contact and/or is posterior to entoplastron; 1 = mixture; 2 = contacts entoplastron.

**Pectoral**

CHARACTER 231: Pectoral A

**Character definition**. Contour of pectoral-abdominal sulcus (*Gaffney & Meylan, 1988*; *Stephens & Wiens, 2003*, 202): 0 = horizontal; 1 = sloped posteromedially to approach abdominal-femoral sulcus.

**Abdominal**

CHARACTER 232: Abdominal A

**Character definition**. Abdominal-femoral sulcus (NEW): 0 = concavely-curved towards the posterior of the shell medially; 1 = flat, or only inconspicuously concavely curved posteriorly.

CHARACTER 233: Abdominal B

**Character definition**. Lateral edges of the abdominal-femoral sulcus (NEW): 0 = laterally curved with the lateral-most edge oriented posteriorly; 1 = flat, or very inconspicuous curve; 2 = laterally curved with lateral-most edge oriented anteriorly.

**Femoral**

CHARACTER 234: Femoral A

**Character definition**. Femoral overlap (*Weaver & Rose, 1967*Modified and Reordered character states): 0 = wide; 1 = thin.

CHARACTER 235: Femoral B

**Character definition**. Contour of anterior apex of femoral-anal sulcus (*Stephens & Wiens, 2003*, 203; Added character state and Reworded): 0 = acute; 1 = mixture; 2 = smooth curving line.

CHARACTER 236: Femoral C

**Character definition**. Posterior plastral lobe indent at (or just posterior to) lateral edge of femoral-anal sulcus (*Galbreath, 1948*; *Minx, 1996*, IL; *Stephens & Wiens, 2003*, 204; Added character state and Reworded): 0 = absent or very faint; 1 = mixture; 2 = present and commonly pronounced or distinct.

CHARACTER 237: Femoral D

**Character definition**. Femoral-anal sulcus (*Minx, 1996*, CS; Modified wording): 0 = with distal ends curved anteriorly; 1 = straight or with slight curve at its midpoint.

**Axillary**

CHARACTER 238: Axillary A

**Character definition**. Axillary scute (*Minx, 1996*, AS; *Stephens & Wiens, 2003*, 214; Added character state): 0 = absent; 1 = mixture; 2 = present.

CHARACTER 239: Axillary B

**Character definition**. Anterior extent of axillary scute (*Minx, 1996*, AS; *Stephens & Wiens, 2003*, 215; Modified wording and Provided discrete character states): 0 = contacts marginal 4 or sulcus between marginal 3 and marginal 4; 1 = contacts posterior of marginal 3; 2 = contacts well into (at least middle of) marginal 3.

**Apical**

CHARACTER 240: Apical A

**Character definition**. Apical scale (*Minx, 1996*, AP; *Stephens & Wiens, 2003*; 212; Added character state): 0 = absent; 1 = mixture; 2 = present.

**Inguinal**

CHARACTER 241: Inguinal A

**Character definition**. Inguinal scute (*Minx, 1996*, IS; *Stephens & Wiens, 2003*, 208; Added character state): 0 = absent; 1 = mixture; 2 = present.

CHARACTER 242: Inguinal B

**Character definition**. Inguinal scute (*Seidel, 1988*, 19): 0 = does not project laterally; 1 = projected laterally to a point (angle).

CHARACTER 243: Inguinal C

**Character definition**. Posterior extent of inguinal scute (*Seidel, 1994*; *Stephens & Wiens, 2003*, 215; Modified wording and Provided discrete character states): 0 = contacts marginal 7; 1 = contacts sulcus between marginal 7 and marginal 8, or anterior of marginal 8; 2 = extends well onto (at least middle of) marginal 8.
