# Supplementary material for: A new slider turtle (Testudines: Emydidae: Deirochelyinae: Trachemys) from the late Hemphillian (late Miocene/early Pliocene) of eastern Tennessee and the evolution of the deirochelyines
Source: PeerJ. 2018 Feb 13;6:e4338. doi: 10.7717/peerj.4338 (PMC5815335; doi:10.7717/peerj.4338)
Supplement: Supplemental Information 3 [file peerj-06-4338-s003.doc]

**A new slider turtle (Testudines: Emydidae: Deirochelyinae: *Trachemys*) from the late Hemphillian (late Miocene/early Pliocene) of eastern Tennessee and the evolution of the deirochelyines**

Steven E. Jasinski

**APPENDIX 3**

**Character - taxon matrix**

Character – taxon matrix used for the phylogenetic study in the present study. Abbreviations: a = states 0 & 1; b = 1 & 2; c = 0 & 2; d = 0 & 3; e = 1 & 3.

**Modern Taxa**

*Clemmys guttata*

110110021100120010221210101001020??011112001002201000022111200021110100021010110021020022010011000010000121102220000210a00001211000??01000000000??0a1aa120211?a20???10000010020210??20?000111111100?000120000101210011?12?0022101111021010111100201

*Chrysemys picta picta*

0110?20110111222112201a010200002220011112001010120110022222001021200100021010111011020000200110000000000110002220000210100001211000??0a000000000110aaaa120211?a2120010001000020100a002100001111110100000?011010a12020101101002102100011110111110211

*Deirochelys reticularia reticularia*

0110?22200001222102202001010000220?0000220210022101200122210011200102000212111100110200112101000200?1100110102220?????0000100101100??00112110000000a1aa1200?1?10000011120a011202000210000002111110010000?0010a0a02120112011121102010a11001111211211

*Graptemys barbouri*

0?10?200??0202221022?0??2001??020???0100100?12002?21?102222021b1000?102020?10??20210001010??0?10000?0?00120002220000221101100100022111?000001000?20a1aa120211?a2????2?10010?2012101?1011111111111??00000?100000000c201?10?a1221?2?1??0111?121210211

*Graptemys geographica*

0?10?1021?0222221022?0??1001??020???0002220112102021?10222201101000?101121?10??20210002020??0?10000?0?0010000222000021110010021a022111?000001000?20a1aa120211?a2????1?10010?2c12101?1011111111111??00000?1000000012111?10?a1221?2?1??0111?111210211

*Graptemys pseudogeographica pseudogeographica*

0110?112110212221022100000000a020??1001e21001210202101012220210b00101010210100120210001010000100000?0100110002210000221101100101022112000000100a020a1aa120211?a2?2102010120220121b1010111111111110100000?100000a0121a12101a122112010101111121210211

*Malaclemys terrapin terrapin*

0110?2022112122210221100101101020??1000e2000122022210101221012a20000002120000a120210101000000110000?010011000222000021110000011110201aa10101100001010aa120212022121a1?100002121210a2201010111111101000010100000001021121100122102011101111111100101

*Pseudemys concinna concinna*

1101?1020100022210221?1010111?1122100011200?12202221001222200102100?100121010??20102200002?10?01000?0?00110102220000221100210100001211000000a00002aa1aa1202a21a201002010010212001210b0001211111110100000?1000001022111010111b1112010a1110012121021b

*Pseudemys nelsoni*

1101?1021100112210221??010210?1122110011210?122022200002222011010d0?101011010??12102201002?10?11000?0?001000????0????21?00110200100??1?000001000?20a1aa120211?a21???10101000020?211?1000100111111??10000?110110110c201?10?00111?211??00100121210211

*Pseudemys rubriventris*

1101?2121100022210221210002101112211001121011221222100022220102c1c10101111210111110220101211110100010100120002220000221100210201100??1a10011a000120a1aa120111?a11200b0100000a2002110110a1000111110110000?110110110020101010012102110000100121210211

*Trachemys decorata*

0110?10210012122112212?02010?002220?0020200?11202221100222202002000?000022010??001102000020?0?00000?0?00?20202220000220000100202??2?120?????10?0?20a1aa120211?a2?0??1?1202011202001?20?1110111111??00000?1000a01202001110?0?c11?2?10100?1??20210201

*Trachemys dorbigni dorbigni*

0110?00200002222112212?02010?002220?0001200?12202?20100222202012030?002022110??000102000020?0?00??????????????????????0100110?????2??21?????10?0?2???????????????0??b?12???11202211?b0?11??11111???0000??1000000?1020111??0?221?2??01?101?12021?21?

*Trachemys gaigeae gaigeae*

0110?????0?0????11??10?1?01?00?22??0????????1???????10????????????????????0?????0?10200??210???0????????????????0????2010000020b0?0??210????10?0?2??????????????02??2?12?2?20202211?b011112111111??00000?100010102020111??01121?2021001011020210211

*Trachemys grayi grayi*

0010?20200000222112212?01010?002220?000e200?12202221100222202202120?00a020110??000112000020?0?000?0?0?001200??????????0100110101??21121?????a0?0?20a1aa120211?a2?0??1?1200011202121?2001100112111??00000?1000a0100220111??0?121?2?2010111?020211210

*Trachemys iversoni*

0110???????0????11??1?????1????22???????????1???????10??????????????????????????0?1?20???2?????0??????????????????????0?00000?????2??2??????10?0?2??????????????????1?12?2???20?011?10?11??111111??0000??1000000?10201?1??0?b21?2?????aa1?02021?21?

*Trachemys nebulosa nebulosa*

0110?????0?0????11??10?0?0???0?22???????????1???????10????????????????????1?????0?10200??20????0??????????????????????0000110101??22021?????10?0?2aa0aa1200?1?a0?2??1?1202021201a11?2001100111101??00000?1000000?1020111??0?121?2?201?111?220211211

*Trachemys ornata ornata*

0110?002000022221122100020100002220000012000122222201000222020010300200002110110001220000200010000????00?20202220000220000110201000??210000010000ba11111202121a2020c111202021c0201a020?11001111110100000?100010101020111100112112000100111220210211

*Trachemys scripta elegans*

0110?20210011222112212111010000222000011200012202121100221101102101000201101011101102000021011000001010012010222000022010000010b101102110210100012aa1aa120211?a21200211202000201121211011111111110100000?100010112020111000022102111001010120210211

*Trachemys stejnegeri stejnegeri*

0110?20200000200112212?02010?002220?000?200?122022201000222210020b0?02b222010??002102000020?0?00??????????????????????0000000b02??2b121?????10?0?20a1aa120211?a2?0??2?1201011c01001?2011100111111??00000?1000101000201110?0?211?2?00101a1?220210201

*Trachemys taylori*

0110?????0?0????11??12?0?0???0?22???????????1???????10????????????????????1?????0?12200??20????0??????????????????????0000110101??21021?????10?0?2???????????????0??1?12?1?21c02011?2011101111111??0000101000101?2020111??0?221?2?1010111?120210211

*Trachemys terrapen*

0110?20220002222112212?0?010?002220?0023200?12202220100222200012000?002022010??002102000020?0?00??????????????????????0000000201??21121?????10?0?2???????????????0??2?1221010202001?1011100111111??00000?1000a0122220111??0?211?2?1100111?020210201

*Trachemys yaquia*

0110?????0?0????11??10?0?0???0?22???????????1???????10????????????????????1?????0?12200??20????0??????????????????????0100110?????2??21?????10?0?2???????????????2??1?12?2?11202011?10?11??111111??0000??1000?01?1020111??0?121?2??01?011?02021?21?

**Fossil Taxa**

*Chrysemys timida*

???????????????????????????????????????????????????????????????????????????????????????????????????????????????????????10?0?0121000??00000000000?001111121203002?200?00??00????203002000000311211?10000???00?????2???11?????021?2??????11????2??2??

*Chrysemys williamsi*

???????????????????????????????????????????????????????????????????????????????????????????????????????????????????????10?1?0100000??000000000????01111?????????0??00?0?0002000????0?0000001????1??0000??00000?00222?11?1001121?20??101111?012??2??

*Deirochelys carri*

????????????????????????????????????????????????????????????????????????????????????????????????2??????????????????????10?1?0101100??001121100000001111121212012??????12000?10?200021000000311111??1000???0?00?a0?1?01?????1221?2????0110?0202??2??

*Deirochelys floridana*

??????????????????????????????????????????????????????????????????????????????????????????????????????????????????????????1?0???1?0????11211000011????????????????????12???10000?0?2???????????????????????????????????????????????????????????????

*Pseudemys caelata*

???????????????????????????????????????????????????????????????????????????????????????????????????????????????????????a0?1?0???100??0?11211000002?????????????????01111?0021100??12?0?00??????????1000???0?????22020?????0??????120???011?????????

*Graptemys kerneri*

1110?200?10202221022??0?200000020??1000020001202222?022222202????001002000?0???2021000200001??00???????????????????????10?0?0?????2111?00000200002??121??????????????????20?1222?011???1???????????000???10?0???022201???????????021101????????????

*Trachemys haugrudi*

0?10?0220?0?0222112?12??2?00010?2200??????0?????2???0?????????020??????????????00210?0000200010000??010012?????????????100000002122b1201001021101201111121212012121a201102200200111002311220111011100000?12011?1222201210010221?2121101000221200212

*Trachemy hillii*

??????????????????2??1?????????????????????????????????????????????????????????????????????????????????????????????????00?0?02211?0??20??????????????11121??????02001?11010?????2??2???0102?11111011000????001?1?????1????10221?2????010102012??2??

*Trachemys idahoensis*

1100?200?10?20?211221201?01?00022201002120011220202?1?0?????2?021001001002010??00?0020200???0?0????????????????????????10?1?02201121?100000010001111111121202012?2021?10?10?120202001020102212211?10000??00000?0?22?11212000221?2?2102100?0012??2??

*Trachemys inflata*

???????????????????????????????????????????????????????????????????????????????00010200002000?01??????00???????????????00?1?0???122212?101a02000020111112121??1?02002?11?2211200?11202?11????????110000???20?1?12220112101??021?2120?01000221??????

*Trachemys platymarginata*

0101?012010?222211221210201000022201002220011220222?1?0220022?020000201000010??00012200002010?01???????????????????????10?1?0101102112010011000012011111212b20a2?2002?110221020021100010102011111010000??01211?1220201210100221?21201011000212??2??
