# Supplementary material for: A new slider turtle (Testudines: Emydidae: Deirochelyinae: Trachemys) from the late Hemphillian (late Miocene/early Pliocene) of eastern Tennessee and the evolution of the deirochelyines
Source: PeerJ. 2018 Feb 13;6:e4338. doi: 10.7717/peerj.4338 (PMC5815335; doi:10.7717/peerj.4338)
Supplement: Supplemental Information 4 [file peerj-06-4338-s004.doc]

**A new slider turtle (Testudines: Emydidae: Deirochelyinae: *Trachemys*) from the late Hemphillian (late Miocene/early Pliocene) of eastern Tennessee and the evolution of the deirochelyines**

Steven E. Jasinski

**Appendix 4: *Trachemys haugrudi* osteology atlas**

*Trachemys haugrudi* is represented by multiple individuals with many that are nearly complete. This appendix shows representative specimens and includes all known elements of *T*. *haugrudi* from different individuals and across the entire skeleton and represent the holotype, paratypes, and referred specimens. All the specimens figured in this atlas are referred to in the main text. The specimens also seek to show the general variation present in *T*. *haugrudi*.

**Figure captions**

**Figure S1.** ***Trachemys* *haugrudi*, paratype nearly complete shell (ETMNH–3558).** (A) nearly complete carapace in dorsal view; (B) nearly complete plastron in ventral view. Scale bar is 10 cm. [intended for page width]

**Figure S2.** ***Trachemys haugrudi*, paratype incomplete shell (ETMNH–3562).** (A) incomplete carapace in dorsal view; (B) incomplete plastron in ventral view. Scale bar is 10 cm. [intended for page width]

**Figure S3.** ***Trachemys haugrudi*, paratype incomplete shell (ETMNH–4686).** (A) incomplete carapace in dorsal view; (B) incomplete plastron in ventral view. Scale bar is 10 cm. [intended for page width]

**Figure S4.** ***Trachemys haugrudi*, paratype nearly complete shell (ETMNH–6935).** (A) carapace in dorsal view; (B) plastron in ventral view. Shell in (C) left lateral, (D) right lateral, (E) anterior and (F) posterior views. Scale bar is 10 cm. [intended for page width]

**Figure S5.** ***Trachemys haugrudi*, paratype nearly complete juvenile plastron (ETMNH–7630)** in (A) ventral (= external) view; (B) dorsal (= internal) view. Scale bar is 10 cm. [intended for page width]

**Figure S6.** ***Trachemys haugrudi*, referred fragmentary carapace and plastron (ETMNH–8311).** Partial posterior carapace in (A) dorsal (= external) view; (B) ventral (= internal) view. Posterior plastron in (C) ventral (= external) view; (D) dorsal (= internal) view. Scale bar is 10 cm. [intended for page width]

**Figure S7.** ***Trachemys haugrudi*, referred partial carapace and plastron (ETMNH–8550).** Posterior carapace in (A) dorsal (= external) view; (B) ventral (= internal) view. Posterior plastron in (C) ventral (= external) view; (D) dorsal (= internal) view. Scale bar is 10 cm. [intended for page width]

**Figure S8.** ***Trachemys haugrudi*, referred incomplete carapace (ETMNH–10390)** in (A) dorsal (= external) view; (B) ventral (= internal) view. Scale bar is 10 cm. [intended for page width]

**Figure S9.** ***Trachemys haugrudi*, referred nearly complete plastron (ETMNH–10390)** in (A) exploded ventral (= external) view with elements in ‘life-placement’ relative to each other; (B) in ventral (= external) view; (C) exploded dorsal (= internal) view with elements in ‘life-placement’ relative to each other; (D) dorsal (= internal) view. Scale bar is 10 cm. [intended for page width]

**Figure S10.** ***Trachemys haugrudi*, referred incomplete juvenile plastron (ETMNH–10391)** in (A) ventral (= external) view; (B) dorsal (= internal) view. Scale bar is 10 cm. [intended for page width]

**Figure S11.** ***Trachemys haugrudi*, referred partial carapace and plastron (ETMNH–10547).** Partial anterior carapace in (A) dorsal (= external) view; (B) ventral (= internal) view. Anterior plastron in (C) ventral (= external) view; (D) dorsal (= internal) view. Scale bar is 10 cm. [intended for page width]

**Figure S12.** ***Trachemys haugrudi*, paratype nearly complete shell (ETMNH–11642)** in (A) dorsal view; (B) ventral view; (C) left lateral view. Scale bar is 10 cm. [intended for page width]

**Figure S13.** ***Trachemys haugrudi*, paratype nearly complete shell (ETMNH–11643)** in (A) dorsal view; (B) ventral view; (C) left lateral view. Scale bar is 10 cm. [intended for page width]

**Figure S14.** ***Trachemys haugrudi*, referred incomplete carapace and nearly complete plastron (ETMNH–12265).** Incomplete carapace in (A) dorsal (= external) view; (B) ventral (= internal) view. Nearly complete plastron in (C) ventral (= external) view; (D) dorsal (= internal) view. Scale bar is 10 cm. [intended for page width]

**Figure S15.** ***Trachemys haugrudi*, referred anterior plastron (ETMNH–12424)** in (A) ventral (= external) view; (B) dorsal (= internal) view. Scale bar is 10 cm. [intended for page width]

**Figure S16.** ***Trachemys haugrudi*, paratype nearly complete shell (ETMNH–12456)** in (A) dorsal view; (B) ventral view. Scale bar is 10 cm. [intended for page width]

**Figure S17.** ***Trachemys haugrudi*, paratype nearly complete carapace and plastron (ETMNH–12457).** Nearly complete carapace in (A) dorsal (= external) view; (B) ventral (= internal) view. Nearly complete plastron in (C) ventral (= external) view; (D) dorsal (= internal) view. Scale bar is 10 cm. [intended for page width]

**Figure S18.** ***Trachemys haugrudi*, paratype nearly complete shell (ETMNH–12726)** in (A) dorsal; (B) ventral; (C) left lateral; (D) right lateral; (E) anterior; and (F) posterior views. Scale bar is 10 cm. [intended for page width]

**Figure S19.** ***Trachemys haugrudi*, referred incomplete shell (ETMNH–12727)** in (A) dorsal view; (B) ventral view. Scale bar is 10 cm. [intended for page width]

**Figure S20.** ***Trachemys haugrudi*, paratype incomplete juvenile shell (ETMNH–12753)** in (A) dorsal view; (B) ventral view. Scale bar is 10 cm. [intended for page width]

**Figure S21.** ***Trachemys haugrudi*, referred nearly complete shell (ETMNH–12772)** in (A) dorsal view; (B) ventral view. Scale bar is 10 cm. [intended for page width]

**Figure S22.** ***Trachemys haugrudi*, paratype nearly complete shell (ETMNH–12832)** in (A) dorsal; (B) ventral; (C) left lateral; and (D) right lateral views. Scale bar is 10 cm. [intended for page width]

**Figure S23.** ***Trachemys haugrudi*, paratype nearly complete shell (ETMNH–12833)** in (A) dorsal view; (B) ventral view. Scale bar is 10 cm. [intended for page width]

**Figure S24.** ***Trachemys haugrudi*, referred incomplete carapace and plastron (ETMNH–12834).** Incomplete carapace in (A) dorsal (= external) view; (B) ventral (= internal) view. Incomplete plastron in (C) ventral (= external) view; (D) dorsal (= internal) view. Scale bar is 10 cm. [intended for page width]

**Figure S25.** ***Trachemys haugrudi*, referred incomplete shell (ETMNH–12979)** in (A) dorsal; (B) ventral; and (C) right lateral views. Scale bar is 10 cm. [intended for page width]

**Figure S26.** ***Trachemys haugrudi*, referred incomplete carapace and plastron (ETMNH–12988).** Incomplete carapace in (A) dorsal (= external) view; (B) ventral (= internal) view. Incomplete plastron in (C) ventral (= external) view; (D) dorsal (= internal) view. Scale bar is 10 cm. [intended for page width]

**Figure S27.** ***Trachemys haugrudi*, referred incomplete carapace and plastron (ETMNH–13032).** Posterior carapace in (A) dorsal (= external) view; (B) ventral (= internal) view. Posterior plastron in (C) ventral (= external) view; (D) dorsal (= internal) view. Scale bar is 10 cm. [intended for page width]

**Figure S28.** ***Trachemys haugrudi*, referred incomplete plastron (ETMNH–13033)** in (A) dorsal (= internal) view; (B) ventral (= external) view. Scale bar is 10 cm. [intended for page width]

**Figure S29.** ***Trachemys haugrudi*, referred incomplete carapace and plastron (ETMNH–13036).** Anterior portion of carapace in (A) dorsal (= external) view; (B) ventral (= internal) view. Medial portion of carapace in (C) dorsal (= external) view; (D) ventral (= internal) view. Articulated right and left epiplastra in (E) dorsal (= internal) view; (F) anterior view; (G) ventral (= external) view. Scale bar is 10 cm. [intended for page width]

**Figure S30.** ***Trachemys haugrudi*, paratype complete carapace and plastron (ETMNH–14362).** Complete carapace in (A) dorsal (= external) view; (B) ventral (= internal) view. Complete carapace in (C) ventral (= external) view; (D) dorsal (= internal) view. Shell in (E) left lateral view; (F) right lateral view. Scale bar is 10 cm. [intended for page width]

**Figure S31.** ***Trachemys haugrudi*, paratype incomplete skull (ETMNH–7690)** in (A) ventral; (B) left lateral; (C) right lateral; (D) anterior (or rostral); and (E) posterior (or caudal) views. Note that dorsal view is not shown because it is covered by a concreted matrix. Scale bar is 2 cm. [intended for page width]

**Figure S32.** ***Trachemys haugrudi*, paratype left maxilla (ETMNH–12457)** in (A) dorsal; (B) ventral; (C) lateral (with anterior to the left); and (D) medial (with anterior to the right) views. Scale bar is 2 cm. [intended for page width]

**Figure S33.** ***Trachemys haugrudi*, lower jaws.** (A–E) paratype nearly complete set of lower jaws (ETMNH–12457) in (A) anterior (or rostral); (B) posterior (or caudal); (C) right posterolateral (focusing on medial surface of left lower jaw); (D) dorsal; and (E) ventral views. (F–I) Paratype nearly complete set of lower jaws (ETMNH–12753) in (F) anterior (or rostral); (G) left posterolateral (or left caudolateral, focusing on medial surface of right lower jaw); (H) dorsal; and (I) ventral views. Scale bar is 2 cm. [intended for page width]

**Figure S34.** ***Trachemys haugrudi*, holotype nearly complete cervical vertebra II (= axis, ETMNH–8549)** in (A) dorsal (cranial to right); (B) ventral (cranial to right); (C) left lateral; (D) right lateral; (E) cranial (or anterior); and (F) caudal (or posterior) views. Scale bar is 1 cm. [intended for page width]

**Figure S35.** ***Trachemys haugrudi*, holotype nearly complete cervical vertebra III (ETMNH–8549)** in (A) dorsal (cranial to right); (B) ventral (cranial to right); (C) left lateral; (D) right lateral; (E) cranial (or anterior); and (F) caudal (or posterior) views. Scale bar is 1 cm. [intended for page width]

**Figure S36.** ***Trachemys haugrudi*, holotype nearly complete cervical vertebra IV (ETMNH–8549)** in (A) dorsal (cranial to right); (B) ventral (cranial to right); (C) left lateral; (D) right lateral; (E) cranial (or anterior); and (F) caudal (or posterior) views. Scale bar is 1 cm. [intended for page width]

**Figure S37.** ***Trachemys haugrudi*, holotype nearly complete cervical vertebra V (ETMNH–8549)** in (A) dorsal (cranial to right); (B) ventral (cranial to right); (C) left lateral; (D) right lateral; (E) cranial (or anterior); and (F) caudal (or posterior) views. Scale bar is 1 cm. [intended for page width]

**Figure S38.** ***Trachemys haugrudi*, holotype nearly complete cervical vertebra VI (ETMNH–8549)** in (A) dorsal (cranial to right); (B) ventral (cranial to right); (C) left lateral; (D) right lateral; (E) cranial (or anterior); and (F) caudal (or posterior) views. Scale bar: 1 cm. [intended for page width]

**Figure S39.** ***Trachemys haugrudi*, holotype nearly complete cervical vertebra VII (ETMNH–8549)** in (A) dorsal (cranial to right); (B) ventral (cranial to right); (C) left lateral; (D) right lateral; (E) cranial (or anterior); and (F) caudal (or posterior) views. Scale bar is 1 cm. [intended for page width]

**Figure S40.** ***Trachemys haugrudi*, paratype nearly complete cervical vertebra VIII (ETMNH–12832)** in (A) dorsal (cranial to right); (B) ventral (cranial to right); (C) left lateral; (D) right lateral; (E) cranial (or anterior); and (F) caudal (or posterior) views. Scale bar is 1 cm. [intended for page width]

**Figure S41.** ***Trachemys haugrudi*, holotype anterior dorsal vertebra (ETMNH–8549)** in (A) dorsal (cranial to right); (B) ventral (cranial to right); (C) left lateral; (D) right lateral; (E) cranial (or anterior); and (F) caudal (or posterior) views. Scale bar is 5 mm. [intended for page width]

**Figure S42.** ***Trachemys haugrudi*, holotype posterior dorsal vertebra (ETMNH–8549)** in (A) dorsal (cranial to right); (B) ventral (c); (C) left lateral; (D) right lateral; (E) cranial (or anterior); and (F) caudal (or posterior) views. Scale bar is 5 mm. [intended for page width]

**Figure S43.** ***Trachemys haugrudi*, holotype posterior dorsal vertebra (posterior to dorsal vertebra in Figure S42) (ETMNH–8549)** in (A) dorsal (cranial to right); (B) ventral (cranial to right); (C) left lateral; (D) right lateral; (E) cranial (or anterior); and (F) caudal (or posterior) views. Scale bar is 5 mm. [intended for page width]

**Figure S44.** ***Trachemys haugrudi*, holotype proximal caudal vertebra (ETMNH–8549)** in (A) dorsal (cranial to right); (B) ventral (cranial to right); (C) left lateral; (D) right lateral; (E) cranial (or anterior); and (F) caudal (or posterior) views. Scale bar is 5 mm. [intended for page width]

**Figure S45.** ***Trachemys haugrudi*, holotype medioproximal caudal vertebra (posterior to caudal vertebra in Figure S44) (ETMNH–8549)** in (A) dorsal (cranial to right); (B) ventral (cranial to right); (C) left lateral; (D) right lateral; (E) cranial (or anterior); and (F) caudal (or posterior) views. Scale bar is 5 mm. [intended for page width]

**Figure S46.** ***Trachemys haugrudi*, holotype median caudal vertebra (ETMNH–8549)** in (A) dorsal (cranial to right); (B) ventral (cranial to right); (C) left lateral; (D) right lateral; (E) cranial (or anterior); and (F) caudal (or posterior) views. Scale bar is 5 mm. [intended for page width]

**Figure S47.** ***Trachemys haugrudi*, holotype mediodistal caudal vertebra (ETMNH–8549)** in (A) dorsal (cranial to right); (B) ventral (cranial to right); (C) left lateral; (D) right lateral; (E) cranial (or anterior); and (F) caudal (or posterior) views. Scale bar is 5 mm. [intended for page width]

**Figure S48.** ***Trachemys haugrudi*, holotype distal caudal vertebra (posterior to caudal vertebra in Figure S47) (ETMNH–8549)** in (A) dorsal (cranial to right); (B) ventral (cranial to right); (C) left lateral; (D) right lateral; (E) cranial (or anterior); and (F) caudal (or posterior) views. Scale bar is 5 mm. [intended for page width]

**Figure S49.** ***Trachemys haugrudi*, scapula and coracoid.** (A–B) holotype nearly complete left scapula (ETMNH–8549) in (A) cranial (or anterior) view; (B) caudal (or posterior) view. (C–D) Holotype nearly complete right scapula (ETMNH–8549) in (C) cranial (or anterior) view; (D) caudal (or posterior) view. (E–F) Paratype nearly complete right coracoid (ETMNH–12832) in (E) dorsocranial (or dorsoanterior) view; (F) ventrocaudal (or ventroposterior) view. Scale bar is 2 cm. [intended for page width]

**Figure S50.** ***Trachemys haugrudi*, holotype complete right humerus (ETMNH–8549)** in (A) cranial (or anterior); (B) caudal (or posterior); (C) dorsal; (D) ventral; (E) proximal; and (F) distal views. Scale bar is 2 cm. [intended for page width]

**Figure S51.** ***Trachemys haugrudi*, radius and ulna.** (A–B) paratype complete left radius (ETMNH–11643) in (A) dorsal view; (B) ventral view. (C–D) Paratype complete right ulna (ETMNH–3562) in (C) dorsal view; (D) ventral view. Scale bar is 2 cm. [intended for page width]

**Figure S52.** ***Trachemys haugrudi*, carpals.** (A–B) paratype right distal carpal ?I (ETMNH–3558) in (A) anterior (or proximal) and (B) posterior (or caudal) view. (C–D) distal carpal ?IV (ETMNH–3558) in (C) anterior (or proximal) view; (D) posterior (or caudal) view; distal carpal ?V (ETMNH–3558) in (E) anterior (or proximal) view; (F) posterior (or caudal) view. Scale bar is 5 mm. [intended for page width]

**Figure S53.** ***Trachemys haugrudi*, distal carpals.** (A–B) paratype right distal carpal ?II (ETMNH–3562) in (A) anterior (or proximal) view; (B) posterior (or caudal) view. (C–D) distal carpal ?III (ETMNH–3562) in (C) anterior (or proximal) view; (D) posterior (or caudal) view. Scale bar is 5 mm. [intended for page width]

**Figure S54.** ***Trachemys haugrudi*, paratype right proximal phalanx ?IV (ETMNH–3558)** in (A) dorsal (cranial to right); (B) ventral (cranial to right); (C) left lateral; (D) right lateral; (E) distal (or anterior); and (F) proximal (or posterior) views. Scale bar is 5 mm. [intended for page width]

**Figure S55.** ***Trachemys haugrudi*, paratype right manual ungual ?I (ETMNH–3562)** in (A) dorsal (anterior to right); (B) ventral (anterior to right); (C) left lateral; (D) right lateral; (E) anterior; and (F) proximal (or posterior) views. Scale bar is 5 mm. [intended for page width]

**Figure S56.** ***Trachemys haugrudi*, paratype right manual ungual ?II (ETMNH–3558)** in (A) dorsal (anterior to right); (B) ventral (anterior to right); (C) left lateral; (D) right lateral; (E) distal (or anterior); and (F) proximal (or posterior) views. Scale bar is 5 mm. [intended for page width]

**Figure S57.** ***Trachemys haugrudi*, paratype right manual ungual ?III (ETMNH–3558)** in (A) dorsal (anterior to right); (B) ventral (anterior to right); (C) left lateral; (D) right lateral; (E) distal (or anterior); and (F) proximal (or posterior) views. Scale bar is 5 mm. [intended for page width]

**Figure S58.** ***Trachemys haugrudi*, paratype right manual ungual ?IV (ETMNH–3558)** in (A) dorsal (anterior to right); (B) ventral (anterior to right); (C) left lateral; (D) right lateral; (E) distal (or anterior); and (F) proximal (or posterior) views. Scale bar is 5 mm. [intended for page width]

**Figure S59.** ***Trachemys haugrudi*, holotype nearly complete left ilium (ETMNH–8549)** in (A) lateral view, (B) medial view. Paratype complete left ilium (ETMNH–12832) in (C) lateral view; (D) medial view. Scale bar is 2 cm. [intended for page width]

**Figure S60.** ***Trachemys haugrudi*, ischium.** (A–B) holotype nearly complete right ischium (ETMNH–8549) in (A) anteroventral view; (B) posterodorsal view. (C–D) Holotype nearly complete left ischium (ETMNH–8549) in (C) anteroventral view; (D) posterodorsal view. (E–F) Paratype nearly complete left ischium (ETMNH–12832) in (E) anteroventral view; (F) posterodorsal view. Scale bar is 2 cm. [intended for page width]

**Figure S61.** ***Trachemys haugrudi*, pubis.** (A–B) holotype nearly complete left pubis (ETMNH–8549) in (A) dorsal view; (B) ventral view. (C–D) Paratype nearly complete left pubis (ETMNH–12832) in (C) dorsal view; (D) ventral view. Scale bar is 2 cm. [intended for page width]

**Figure S62.** ***Trachemys haugrudi*, referred complete right femur (ETMNH–12265)** in (A) anterior; (B) posterior; (C) dorsal; (D) ventral; (E) proximal (or medial); and (F) distal (or lateral) views. Scale bar is 2 cm. [intended for page width]

**Figure S63.** ***Trachemys haugrudi*, tibia and fibula.** (A–F) paratype complete left tibia (ETMNH–12832) in (A) dorsal (proximal end to right); (B) medioventral (proximal end to right); (C) lateroventral; (D) mediodorsal; (E) proximal; and (F) distal views. (G–H) Holotype complete left fibula (ETMNH–8549) in (G) dorsal view; (H) ventral view. Scale bar is 2 cm. [intended for page width]

**Figure S64.** ***Trachemys haugrudi*, holotype right metatarsal ?II (ETMNH–8549)** in (A) dorsal (cranial to right); (B) ventral (cranial to right); (C) left lateral; (D) right lateral; (E) distal (or anterior); and (F) proximal (or posterior) views. Scale bar is 5 mm. [intended for page width]

**Figure S65.** ***Trachemys haugrudi*, holotype right metatarsal III (ETMNH–8549)** in (A) dorsal (cranial to right); (B) ventral (cranial to right); (C) left lateral; (D) right lateral; (E) distal (or anterior); and (F) proximal (or posterior) views. Scale bar is 5 mm. [intended for page width]

**Figure S66.** ***Trachemys haugrudi*, holotype right metatarsal V, right astragalus, and incomplete right distal tarsal ?IV (ETMNH–8549).** Holotype right metatarsal V (ETMNH–8549) in (A) dorsal (or proximal) view; (B) ventral (or distal) view. Holotype right astragalus (ETMNH–8549) in (C) dorsal (or proximal) view; (D) ventral (or distal) view. Holotype incomplete right distal tarsal ?IV (ETMNH–8549) in (E) dorsal (or proximal view; (F) ventral (or distal) view. Scale bar is 5 mm. [intended for page width]

**Figure S67.** ***Trachemys haugrudi*, holotype right proximal phalanx ?III (ETMNH–8549)** in (A) dorsal (cranial to right); (B) ventral (cranial to right); (C) left lateral; (D) right lateral; (E) distal (or anterior); and (F) proximal (or posterior) views. Scale bar is 5 mm. [intended for page width]

**Figure S68.** ***Trachemys haugrudi*, holotype right proximal phalanx V (ETMNH–8549)** in (A) dorsal (cranial to right); (B) ventral (cranial to right); (C) left lateral; (D) right lateral; (E) distal (or anterior); and (F) proximal (or posterior) views. Scale bar is 5 mm. [intended for page width]

**Figure S69.** ***Trachemys haugrudi*, holotype right medial phalanx ?III (ETMNH–8549)** in (A) dorsal (cranial to right); (B) ventral (cranial to right); (C) left lateral; (D) right lateral; (E) distal (or anterior); and (F) proximal (or posterior) views. Scale bar is 5 mm. [intended for page width]

**Figure S70.** ***Trachemys haugrudi*, holotype right distal phalanx ?II (ETMNH–8549)** in (A) dorsal (cranial to right); (B) ventral (cranial to right); (C) left lateral; (D) right lateral; (E) distal (or anterior); and (F) proximal (or posterior) views. Scale bar is 5 mm. [intended for page width]

**Figure S71.** ***Trachemys haugrudi*, referred pedal ungual ?III (ETMNH–10547)** in (A) dorsal (cranial to right); (B) ventral (cranial to right); (C) left lateral; (D) right lateral; (E) distal (or anterior); and (F) proximal (or posterior) views. Scale bar is 5 mm. [intended for page width]

**Figure S72.** ***Trachemys haugrudi*, paratype pedal ungual ?IV (ETMNH–11642)** in (A) dorsal (cranial to right); (B) ventral (cranial to right); (C) left lateral; (D) right lateral; (E) distal (or anterior); and (F) proximal (or posterior) views. Scale bar is 5 mm. [intended for page width]
