# Supplementary material for: A new slider turtle (Testudines: Emydidae: Deirochelyinae: Trachemys) from the late Hemphillian (late Miocene/early Pliocene) of eastern Tennessee and the evolution of the deirochelyines
Source: PeerJ. 2018 Feb 13;6:e4338. doi: 10.7717/peerj.4338 (PMC5815335; doi:10.7717/peerj.4338)
Supplement: Supplemental Information 5 [file peerj-06-4338-s005.docx]

**A new slider turtle (Testudines: Emydidae: Deirochelyinae: *Trachemys*) from the late Hemphillian (late Miocene/early Pliocene) of eastern Tennessee and the evolution of the deirochelyines**

Steven E. Jasinski

**Appendix 5:** Supplemental Figures S73–S74 providing 50% majority rule consensus trees from the phylogenetic analyses. Age ranges of fossil taxa discussed in Appendix 6.

**Figure S73.** **Phylogenetic relationships of deirochelyine emydids supported by this study based on morphologic data.** See text and appendices for details. Analysis includes the emydine *Clemmys guttata* as the outgroup. The tree presented represents the 50% majority rule consensus tree of deirochelyine relationships found in the phylogenetic analysis of 243 characters within 31 taxa. Clades listed are called out and discussed in the text. Thickened bars indicate age ranges for fossil taxa. Tree length equals 874 steps, consistency index equals 0.413, retention index equals 0.483. Numbers beside branches provide the bremer supports (first) and the bootstrap values (second). Numbers are shown for branches with bootstrap values greater than 33. Abbreviations: A, Clade ‘A’; B, Clade ‘B’; C, Clade ‘C’; D, Clade ‘D’; E, Clade ‘E’; F, Clade ‘F’; G, Clade ‘G’; H, Clade ‘H’; I, Clade ‘I’; J, Clade ‘J’; K, Clade ‘K’. [intended for page width]

**Figure S74.** **Phylogenetic relationships of deirochelyine emydids supported by this study based on morphologic data constrained by a molecular backbone.** See text and appendices for details. Intergeneric relationships of modern taxa constrained based on molecular phylogenetic analysis presented by Spinks et al. (2016, fig. 5). Analysis includes the emydine *Clemmys guttata* as the outgroup. The tree presented represents the 50% majority rule consensus tree of deirochelyine relationships found in the phylogenetic analysis of 243 characters within 31 taxa. Clades listed are called out and discussed in the text. Thickened bars indicate age ranges for fossil taxa. Tree length equals 882 steps, consistency index equals 0.409, retention index equals 0.475. Numbers beside branches provide the bremer supports (first) and the bootstrap values (second). Numbers are shown for branches with bootstrap values greater than 33. Abbreviations: A, Clade ‘A’; B, Clade ‘B’; C, Clade ‘C’; D, Clade ‘D’; E, Clade ‘E’; F, Clade ‘F’; G, Clade ‘G’; H, Clade ‘H’; I, Clade ‘I’; J, Clade ‘J’; K, Clade ‘K’. [intended for page width]
