# Supplementary material for: A new slider turtle (Testudines: Emydidae: Deirochelyinae: Trachemys) from the late Hemphillian (late Miocene/early Pliocene) of eastern Tennessee and the evolution of the deirochelyines
Source: PeerJ. 2018 Feb 13;6:e4338. doi: 10.7717/peerj.4338 (PMC5815335; doi:10.7717/peerj.4338)
Supplement: Supplemental Information 6 [file peerj-06-4338-s006.docx]

**A new slider turtle (Testudines: Emydidae: Deirochelyinae: *Trachemys*) from the late Hemphillian (late Miocene/early Pliocene) of eastern Tennessee and the evolution of the deirochelyines**

Steven E. Jasinski

**Appendix 6**: Temporal and geologic ranges of fossil taxa in the present study. These ranges are used in Figure 12 and Supplemental Figure 74. References helping to derive the geologic strata and ages of these taxa are also provided.

***Chrysemys* *timida*.** The holotype specimen (YPM PU-10853) was collected from “Equus beds” near the Niobrara River in Sheridan County, Nebraska. These are known to now be Pleistocene in age (Adler, 1968).

**Adler K. 1968.** Synonymy of the Pliocene turtles *Pseudemys hilli* Cope and *Chrysemys limnodytes* Galbreath. *Journal of Herpetology*, **1:**32–38.

**Hay OP.** **1908.** *The Fossil Turtles of North America*. Washington, D.C.: Carnegie Institute of Washington, Publication No. 75, 568 pp.

***Chrysemys* *williamsi*.** The holotype specimen (UF 11561) was collected from McGehee Farm near Newberry, Alachua County, northcentral Florida (Rose & Weaver, 1966). While originally thought to be early Pliocene and late Hemphillian in age (Rose & Weaver, 1966), this has since been revised and considered to be from the early Hemphillian (Hh1) and early late Miocene based on biostratigraphy (Hulbert, 2001; Tedford et al., 2004).

**Hulbert RC**, **Jr**. **2001**. *The Fossil Vertebrates of Florida*. Gainesville: University Press, 350 pp.

**Rose FL, Weaver Jr. WG.** **1966.** Two new species of *Chrysemys* (= *Pseudemys*) from the Florida Pliocene. *Tulane Studies in Geology*, **5:**41–48.

**Tedford RH, Albright LB, Barnosky AD, Ferrusquia-Villafranca I, Hunt RM, Storer JE, Swisher CC, Voorhies MR, Webb SD, Whistler DP.** **2004.** Chapter 6. Mammalian Biochronology of the Arikareean through Hemphillian interval (late Oligocene through early Pliocene epochs) In: Woodburne MO, ed. *Late Cretaceous and Cenozoic Mammals of North America. Biostratigraphy and Geochronology*. New York: Columbia University Press, 169–231.

***Deirochelys carri*.** The holotype specimen (UF 20908) was collected from the Love Bone Bed (or Love Site) near Archer in Alachua County, northcentral Florida (Jackson, 1978). Based on vertebrate biochronology, the site is dated at latest Clarendonian and early late Miocene (Baskin, 2005).

**Baskin JA. 2005.** Carnivora from the Late Miocene Love Bone Bed of Florida. *Bulletin of the Florida Museum of Natural History*, **45:**413–434.

**Jackson DR.** **1978.** Evolution and fossil record of the chicken turtle *Deirochelys*, with a re-evaluation of the genus. *Tulane Studies of Zoology and Botany*, **20:**35–55.

***Deirochelys floridana*.** The holotype specimen (USNM 16679) was collected along Peace Creek in Hillsborough County, Florida (Hay, 1908). This is now believed to be from the Pleistocene (Jackson, 1964).

**Hay OP.** **1908.** *The Fossil Turtles of North America*. Washington, D.C.: Carnegie Institute of Washington, Publication No. 75, 568 pp.

**Jackson CG.** **1964.** The status of *Deirochelys floridana* Hay with comments on the fossil history of the genus. *Tulane Studies in Geology*, **2:**103–106.

***Graptemys kerneri*.** The holotype specimen (UF 239000) was collected on the Suwanee River, at the boundary of the Dixie and Gilchrist Counties, northcentral Florida (Ehret & Bourque, 2011). While the specimen was collected from unconsolidated sediments in the river bed, it is believed to be Rancholabrean, late Pleistocene in age (approximately 15 ka) (Ehret & Bourque, 2011).

**Ehret DJ, Bourque JR.** **2011.** An extinct map turtle *Graptemys* (Testudines: Emydidae) from the late Pleistocene of Florida. *Journal of Vertebrate Paleontology*, **31:**575–587.

***Pseudemys caelata*.** The holotype specimen (USNM 2508) was originally cited as being collected from Mixson’s Bone Bed in Levy County, Florida (Hay, 1908; Jackson, 1976) and hypothesized to be from the Pleistocene (Hay, 1908). This age was revised and more recently has been considered to be from the early Hemphillian (Hh1) and early late Miocene based on biostratigraphy (Jackson, 1976; Prothero, 2005).

**Hay OP.** **1908.** *The Fossil Turtles of North America*. Washington, D.C.: Carnegie Institute of Washington, Publication No. 75, 568 pp.

**Jackson DR.** **1976.** The status of the Pliocene turtle *Pseudemys caelata* Hay and *Chrysemys carri* Rose and Weaver. *Copeia*, **1976:**655–659.

**Prothero DR**. **2005**. *The Evolution of North American Rhinoceroses*. Cambridge: Cambridge University Press, 218 pp.

***Trachemys hillii*.** The holotype specimen (AMNH 2425) is believed to come from the Loup Fork Beds of Decatur County, Kansas (Cope, 1878; Hay, 1908). These are dated to the late Hemphillian and the latest Miocene–earliest Pliocene (Jackson, 1988).

**Cope ED.** **1878.** Descriptions of new extinct Vertebrata from the Upper Tertiary and Dakota formations. *Bulletin of the United States Geological Survey Territories*, **4:**379–396.

**Hay OP.** **1908.** *The Fossil Turtles of North America*. Washington, D.C.: Carnegie Institute of Washington, Publication No. 75, 568 pp.

**Jackson DR.** **1988.** A re-examination of fossil turtles of the genus *Trachemys* (Testudines: Emydidae). *Herpetologica*, **44:**317–325.

***Trachemys idahoensis*.** The holotype (USNM 12059) and paratype (USNM 12060) specimens were collected from the Hagerman lake beds (=*Plesippus* Quarry), near Hagerman, Gooding County, southwestern Idaho (Gilmore, 1933). These strata are from the Glenns Ferry Formation. The specimens were collected from the Pliocene Glenns Ferry Formation (Blancan NALMA) (Gilmore, 1933; Jackson, 1988).

**Gilmore CW.** **1933.** A new species of extinct turtle from the Upper Pliocene of Idaho. *Proceedings of the United States National Museum*, **82:**1–7.

**Jackson DR.** **1988.** A re-examination of fossil turtles of the genus *Trachemys* (Testudines: Emydidae). *Herpetologica*, **44:**317–325.

***Trachemys inflata*.** The holotype specimen (UF 12460) was collected in a phosphate mining area called Palmetto Washer in Polk County, Florida (Weaver & Robertson, 1967). These deposits are known to usually be from the Bone Valley Formation (Hulbert, 2001), and is believed to be from near the upper Miocene-lower Pliocene boundary and late Hemphillian in age (Weaver & Robertson, 1967; Webb, 1969).

**Hulbert RC**, **Jr**. **2001**. *The Fossil Vertebrates of Florida*. Gainesville: University Press, 350 pp.

**Weaver Jr. WG, Robertson JS.** **1967.** A re-evaluation of fossil turtles of the *Chrysemys scripta* group. *Tulane Studies in Geology*, **5:**53–66.

**Webb SD**. **1969**. The Pliocene Canidae of Florida. *Bulletin of the Florida State Museum. Biological Sciences* **14**:273–308.

***Trachemys platymarginata*.** The holotype specimen (UF 11046) was collected from Haile XV A in Alachua County, Florida. Weaver and Robertson (1967) originally thought the site was of Irvingtonian age (Pleistocene), but this was later reinterpreted to be from the Blancan (Pliocene) (Robertson, 1976).

**Weaver Jr. WG, Robertson JS.** **1967.** A re-evaluation of fossil turtles of the *Chrysemys scripta* group. *Tulane Studies in Geology*, **5:**53–66.

**Robertson JS**. **1976**. Latest Pliocene mammals from Haile XV A, Alachua County, Florida. *Bulletin of the Florida State Museum. Biological Sciences*, **20**:111–186.

***Trachemys haugrudi*.** The holotype specimen (ETMNH 8549) and all other material referred to this taxon currently come from the Gray Fossil Site near Gray, Washington County, northeastern Tennessee (Parmalee et al., 2002). The site represents an ancient sinkhole, and based on biostratigraphic ranges of *Teleoceros* (Rhinocerotidae) and *Plionarctos* (Ursidae), has been dated between 7.0 and 4.5 Ma, making it late Hemphillian in age (upper Miocene–lower Pliocene) (Wallace & Wang, 2004).

**Parmalee PW, Klippel WE, Meylan PA, Holman JA.** **2002.** A late Miocene-early Pliocene population of *Trachemys* (Testudines: Emydidae) from east Tennessee. *Annals of Carnegie Museum*, **71:**233–239.

**Wallace SC, Wang X.** **2004.** Two new carnivores from an unusual late Tertiary forest biota in eastern North America. *Nature*, **431:**556–559.

**Institutional Abbreviations**

**AMNH**, American Museum of Natural History, New York, USA; **ETMNH**, East Tennessee State University and General Shale Brick Natural History Museum, Gray, USA; **UF**, University of Florida, Florida Museum of Natural History, Gainesville, USA; **USNM**, United States National Museum of Natural History, Smithsonian Institution, Washington, D.C., USA; **YPM**–**PU**, Yale Peabody Museum–Princeton collection, New Haven, USA.
