# Supplementary material for: A new slider turtle (Testudines: Emydidae: Deirochelyinae: Trachemys) from the late Hemphillian (late Miocene/early Pliocene) of eastern Tennessee and the evolution of the deirochelyines
Source: PeerJ. 2018 Feb 13;6:e4338. doi: 10.7717/peerj.4338 (PMC5815335; doi:10.7717/peerj.4338)
Supplement: Supplemental Information 7 — Trachemys haugrudi is represented by multiple individuals with many that are nearly complete. This appendix shows representative specimens and includes all known elements of T. haugrudi from different individuals and across the entire skeleton and represent the holotype, paratypes, and referred specimens. All the specimens figured in this atlas are referred to in the main text. The specimens also seek to show the general variation present in T. haugrudi. Captions are provided in Appendix 4. [file peerj-06-4338-s007.pdf]

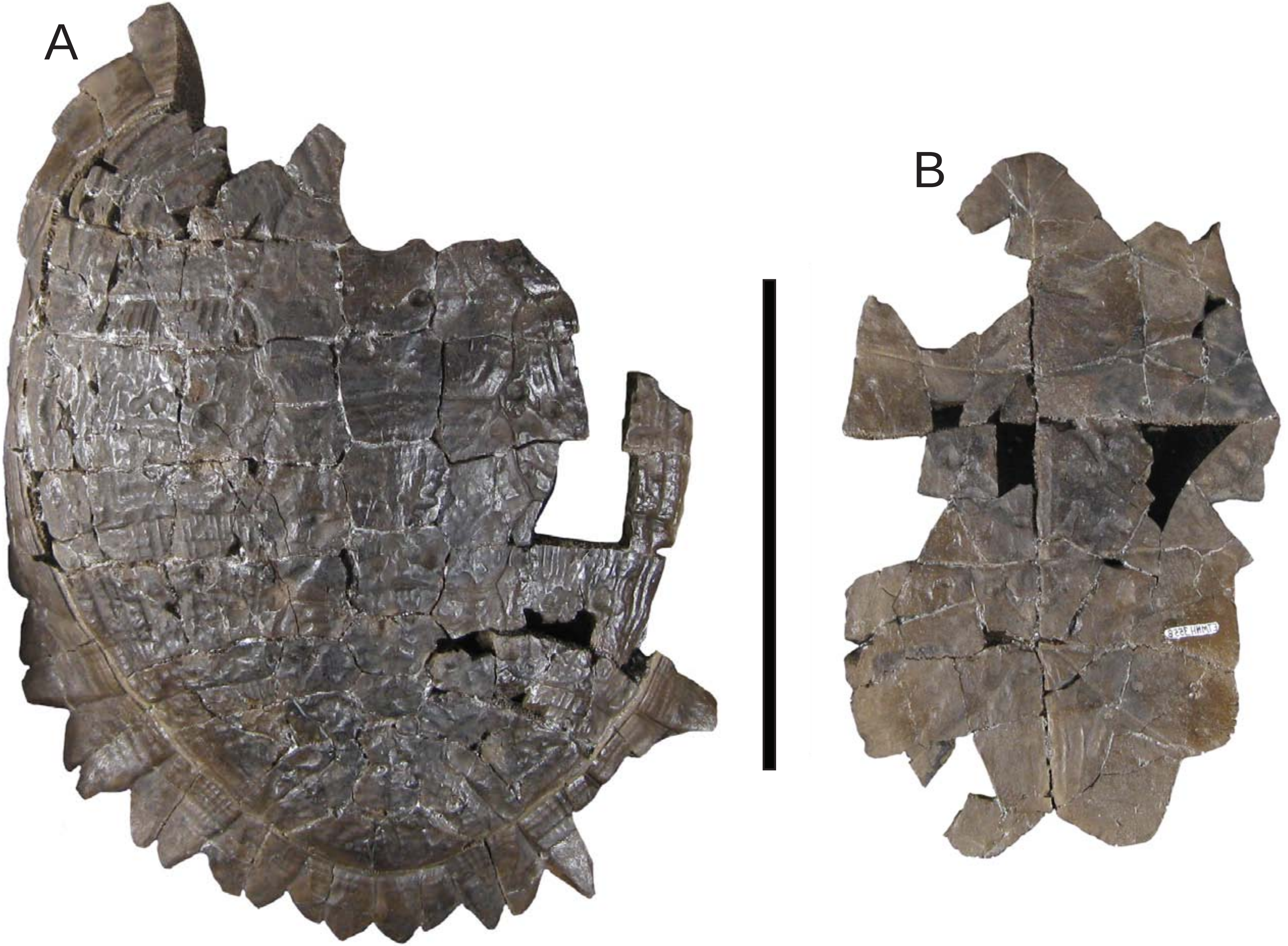

Figure S1. ***Trachemys haugrudi*, paratype nearly complete shell (ETMNH–3558).**

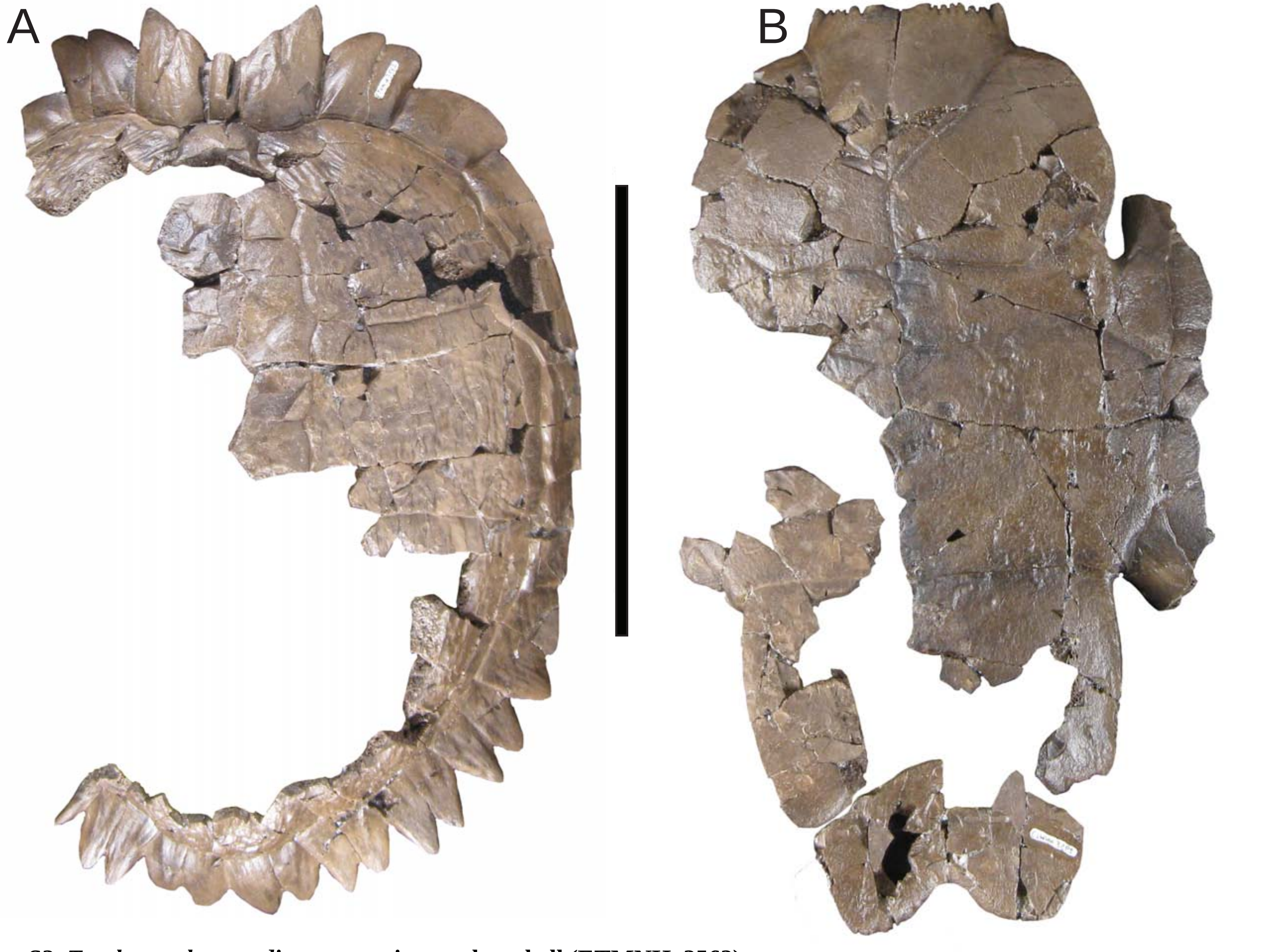

Figure S2. *Trachemys haugrudi*, paratype incomplete shell (ETMNH–3562).

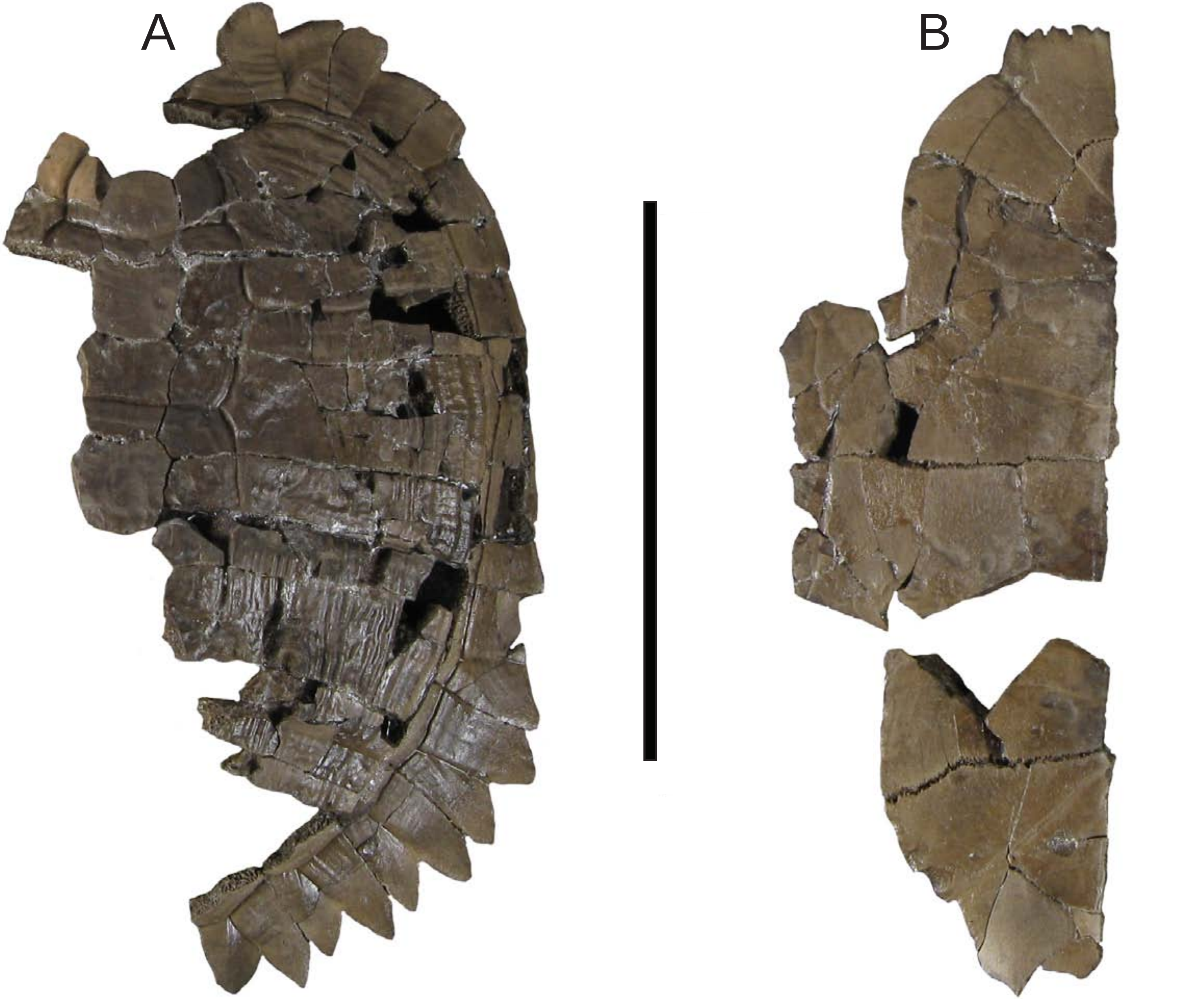

Figure S3. ***Trachemys haugrudi*, paratype incomplete shell (ETMNH–4686).**

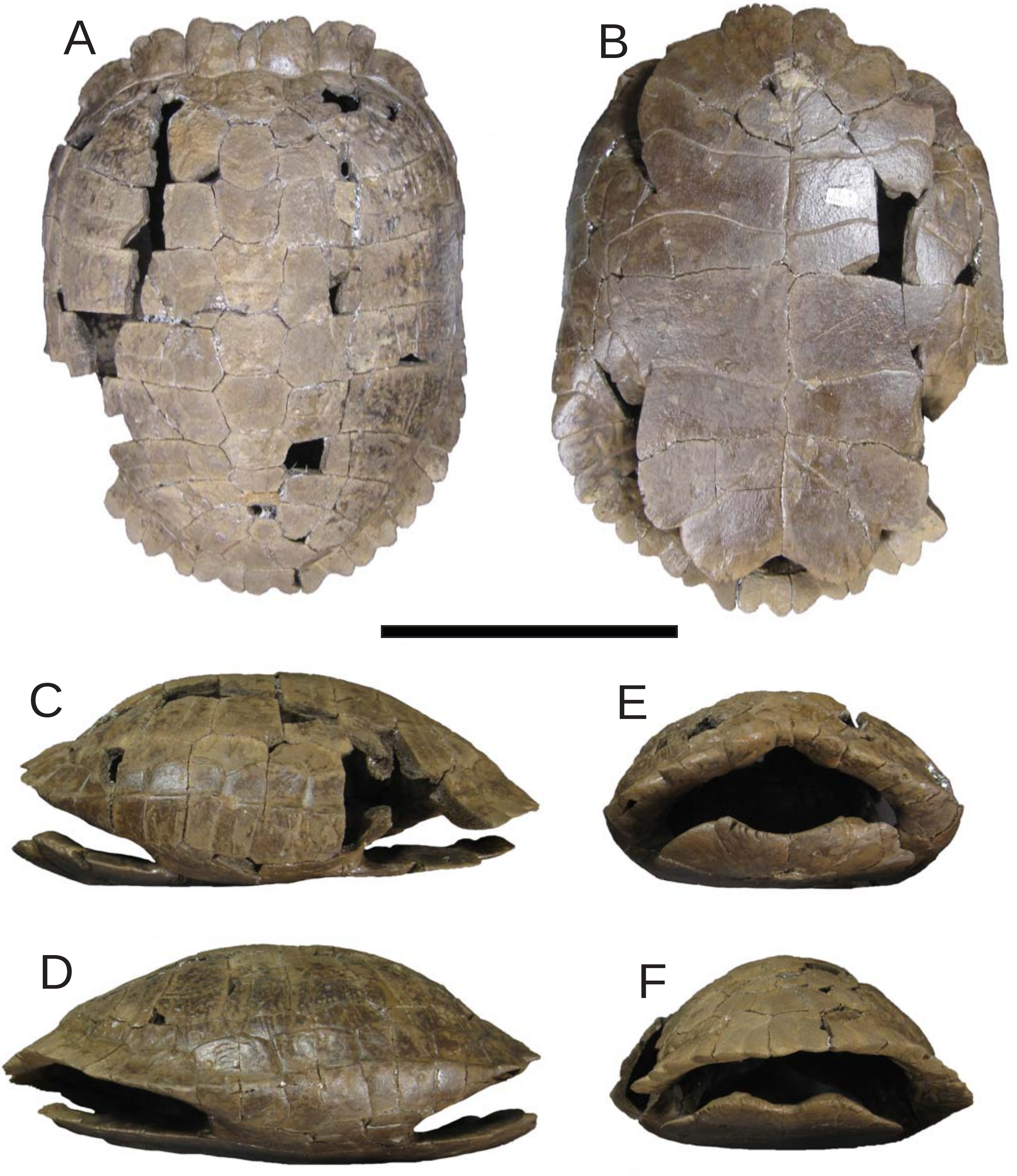

Figure S4. ***Trachemys haugrudi*****, paratype nearly complete shell (ETMNH–6935).**

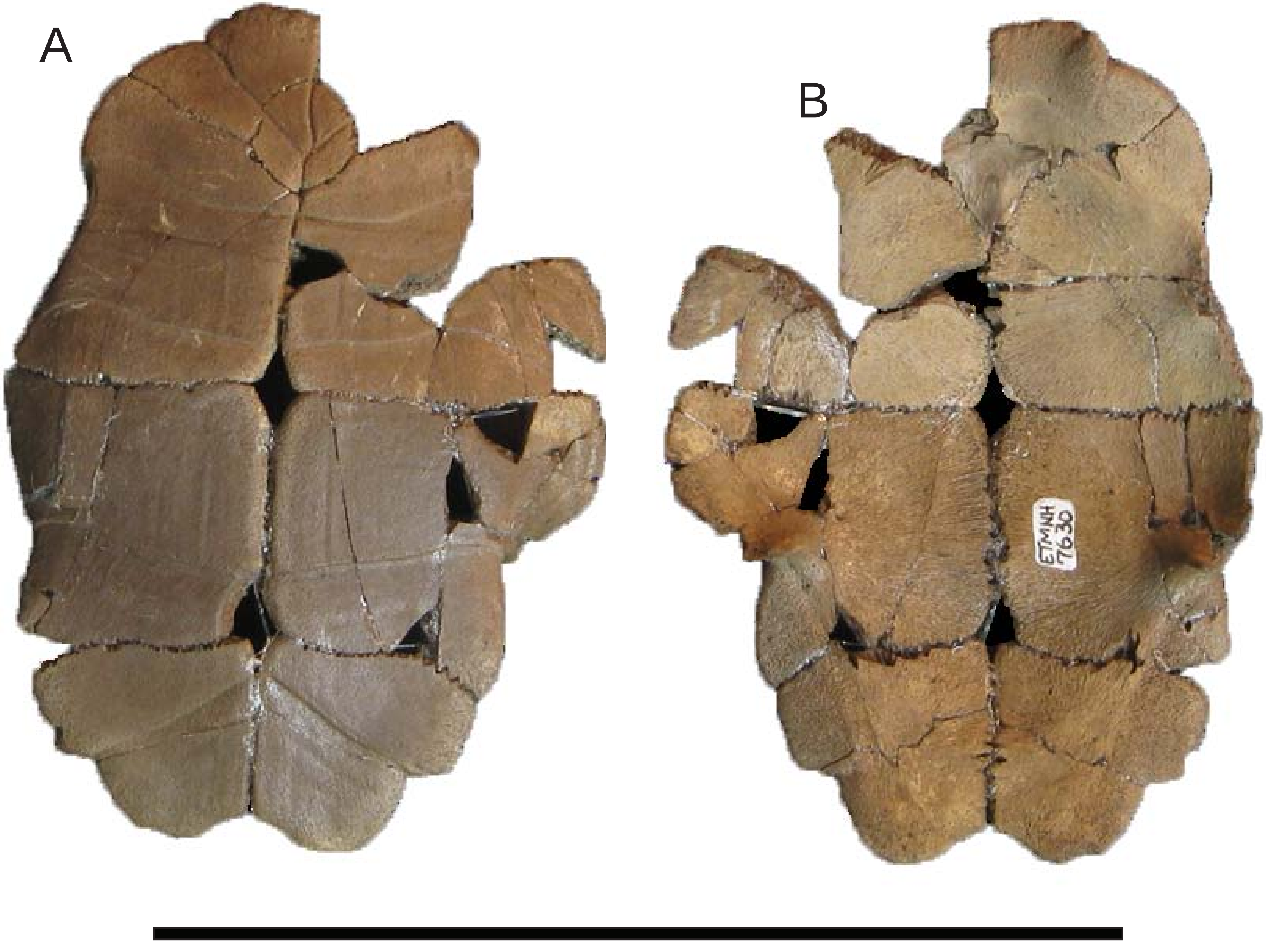

Figure S5. ***Trachemys haugrudi*, paratype nearly complete juvenile plastron (ETMNH–7630).**

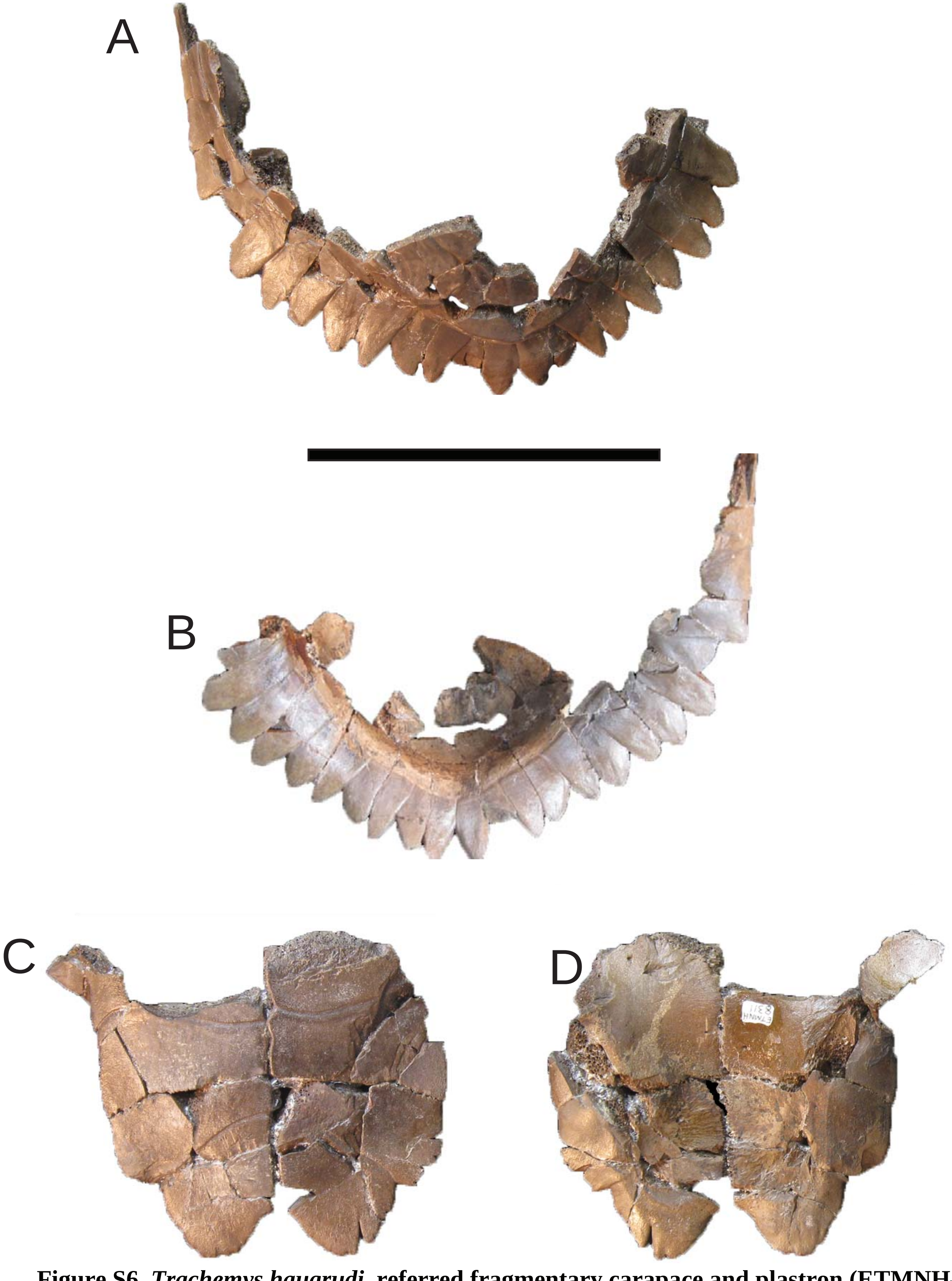

Figure S6. ***Trachemys haugrudi*, referred fragmentary carapace and plastron (ETMNH–8311).**

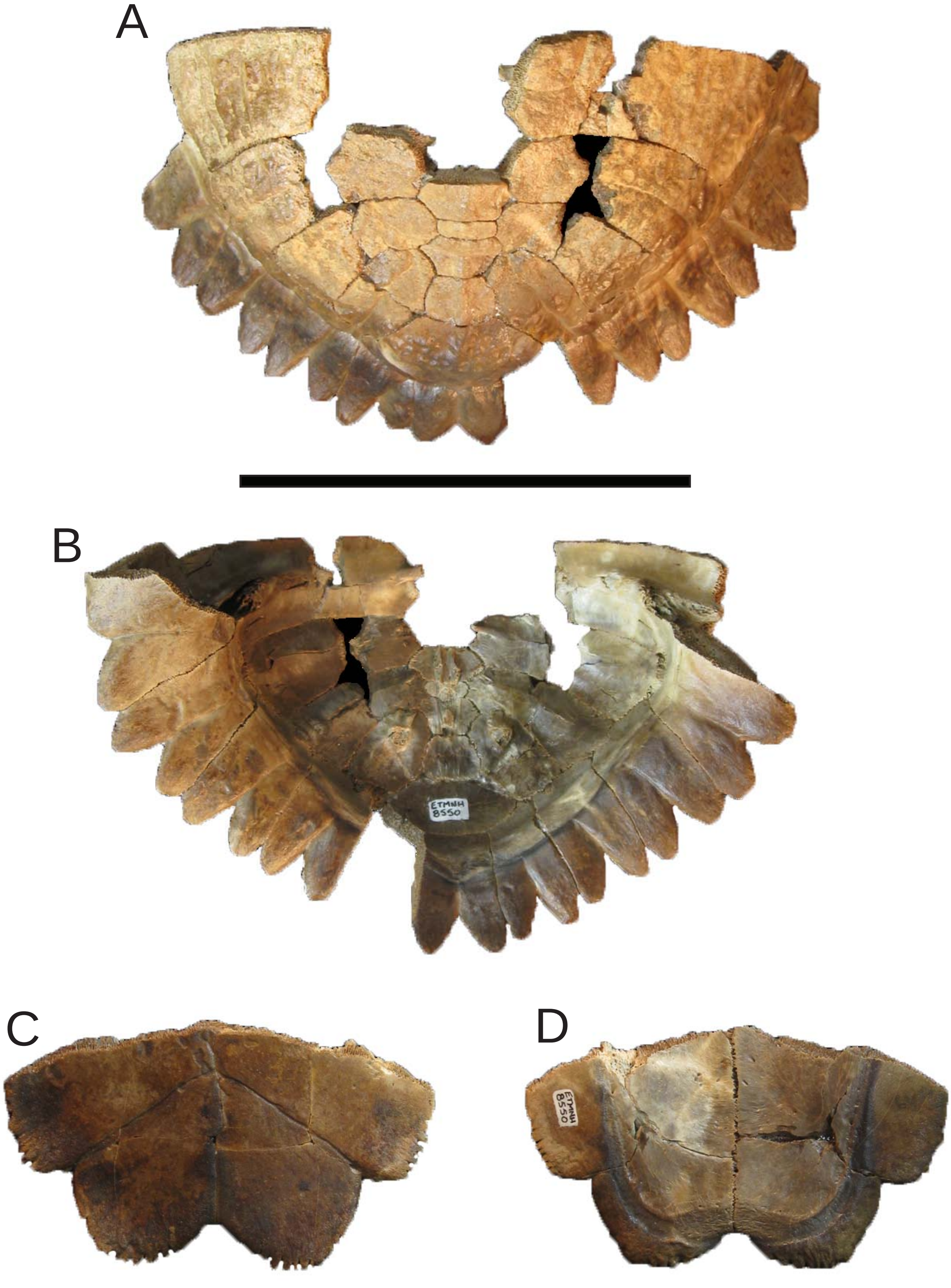

Figure S7. ***Trachemys haugrudi*, referred partial carapace and plastron (ETMNH–8550).**

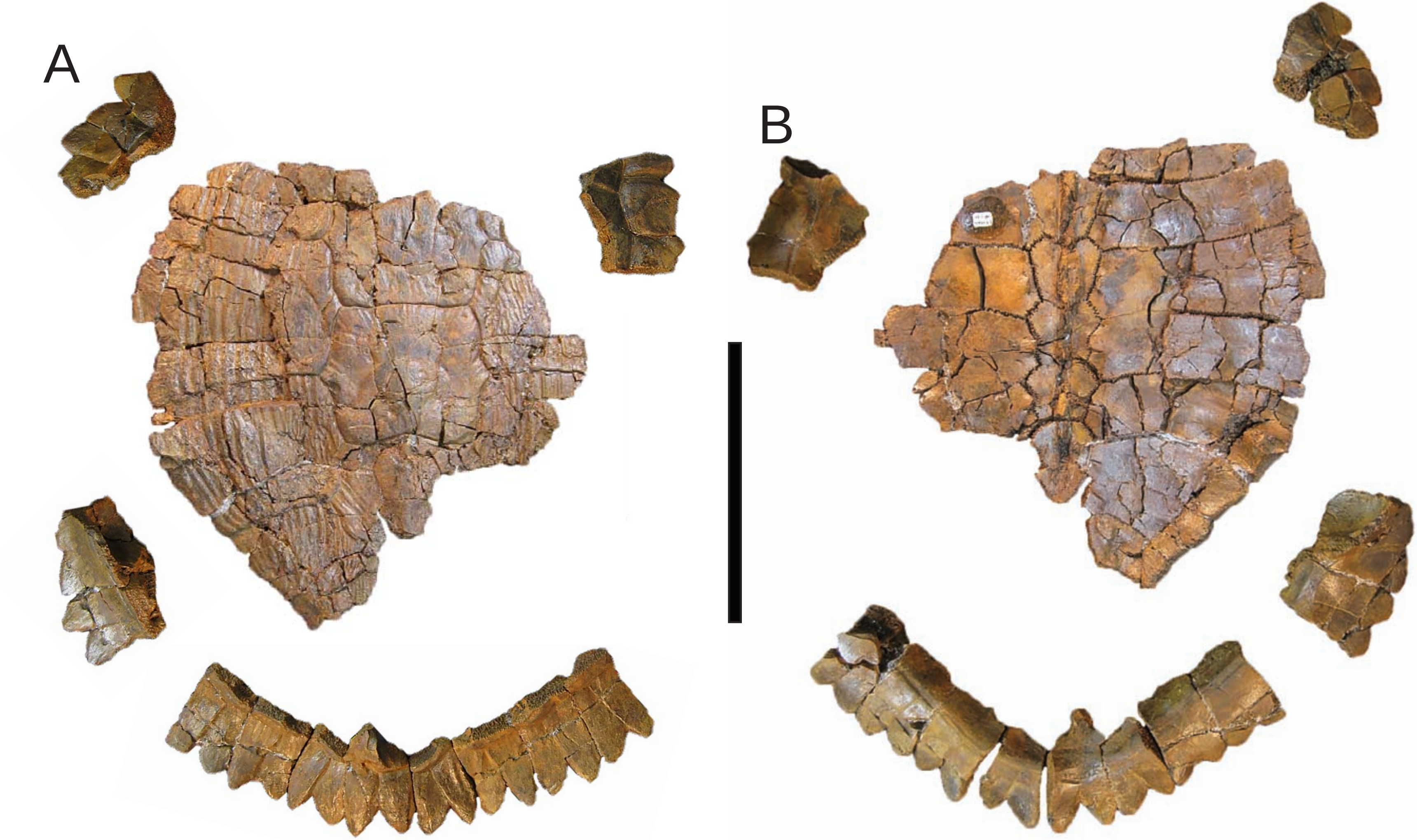

Figure S8. ***Trachemys haugrudi*****, referred incomplete carapace (ETMNH–10390).**

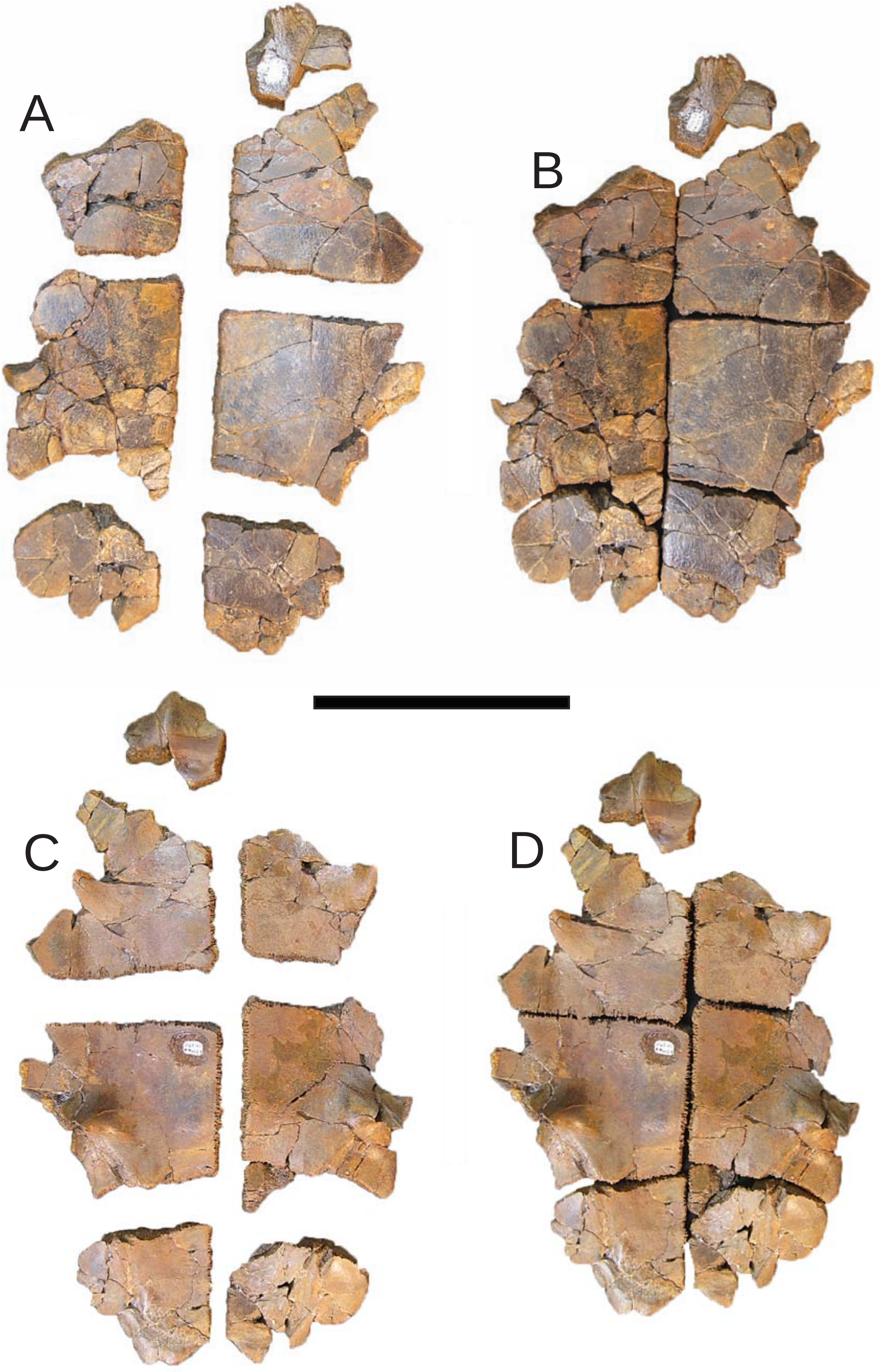

Figure S9. ***Trachemys haugrudi*, referred nearly complete plastron (ETMNH–10390).**

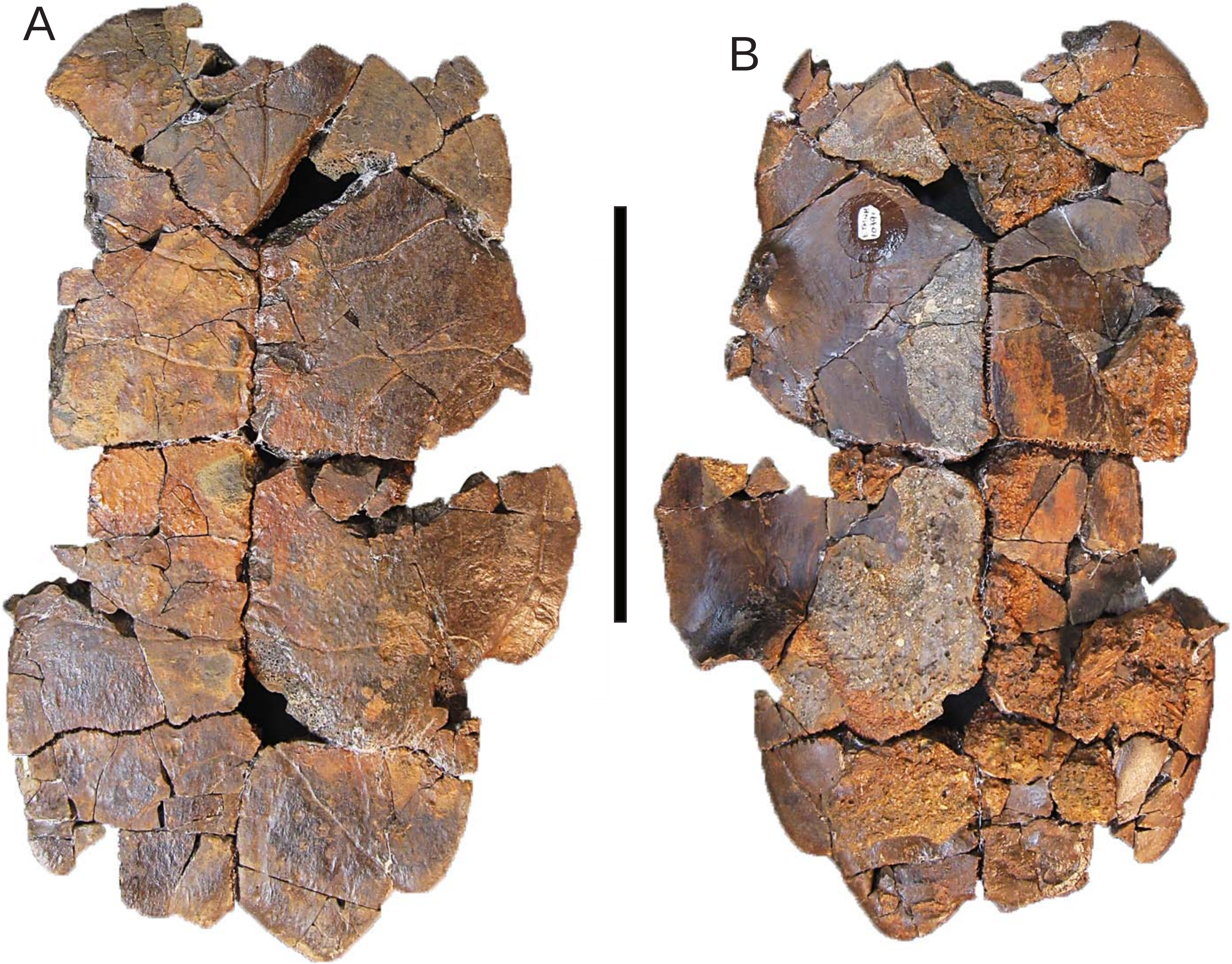

Figure S10. ***Trachemys haugrudi*****, referred incomplete juvenile plastron (ETMNH–10391).**

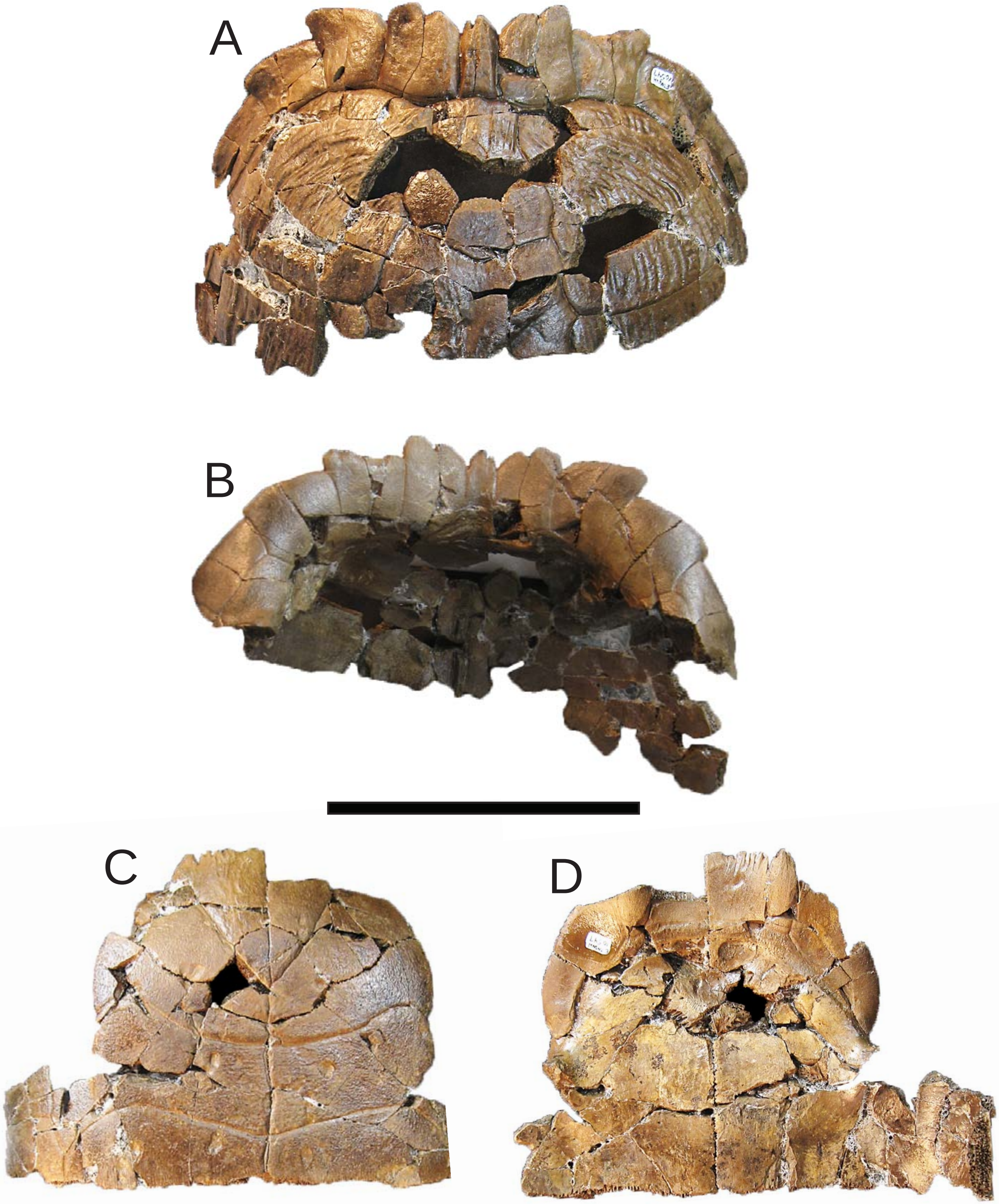

Figure S11. ***Trachemys haugrudi*, referred partial carapace and plastron (ETMNH–10547).**

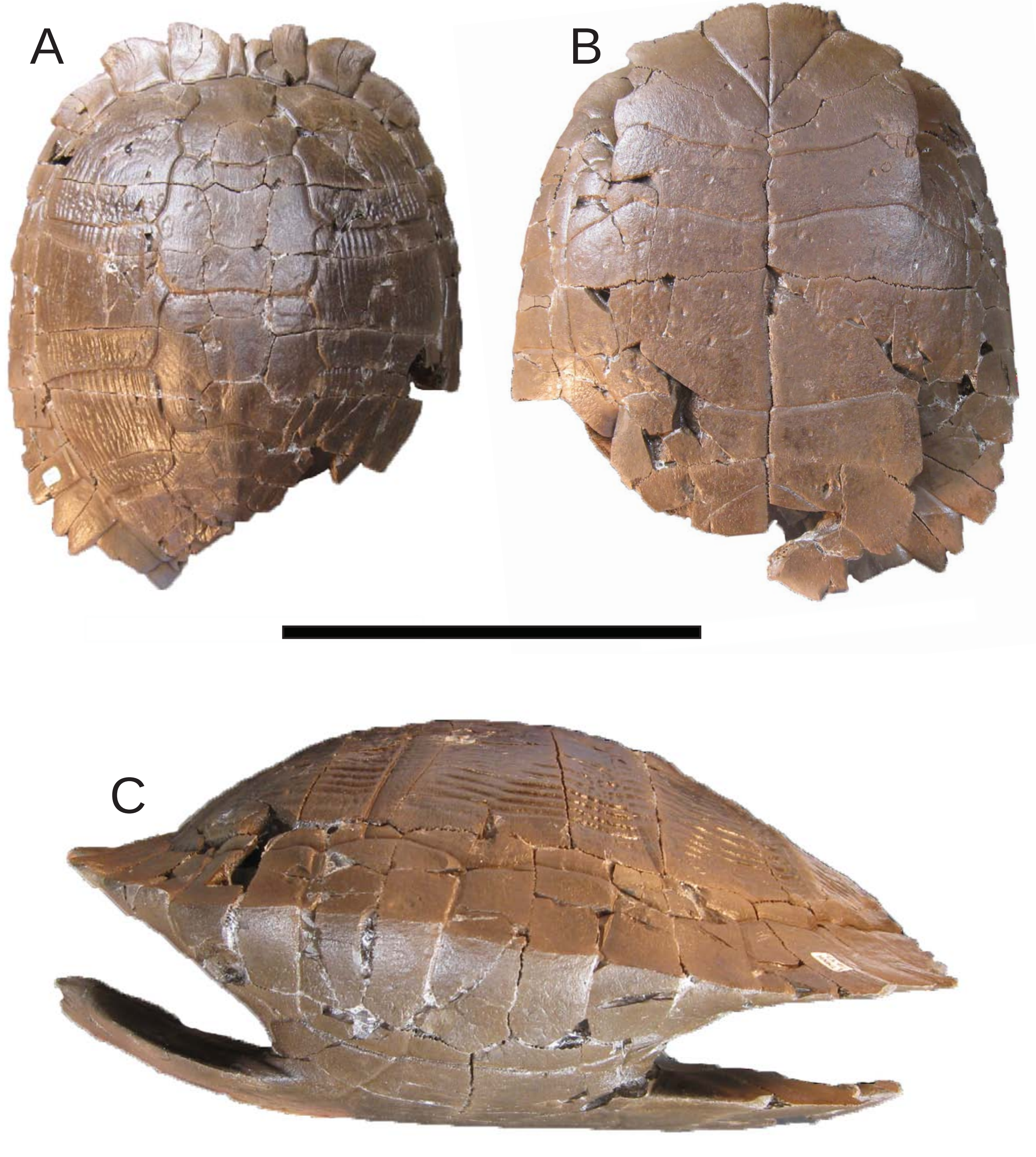

Figure S12. ***Trachemys haugrudi*****, paratype nearly complete shell (ETMNH–11642).**

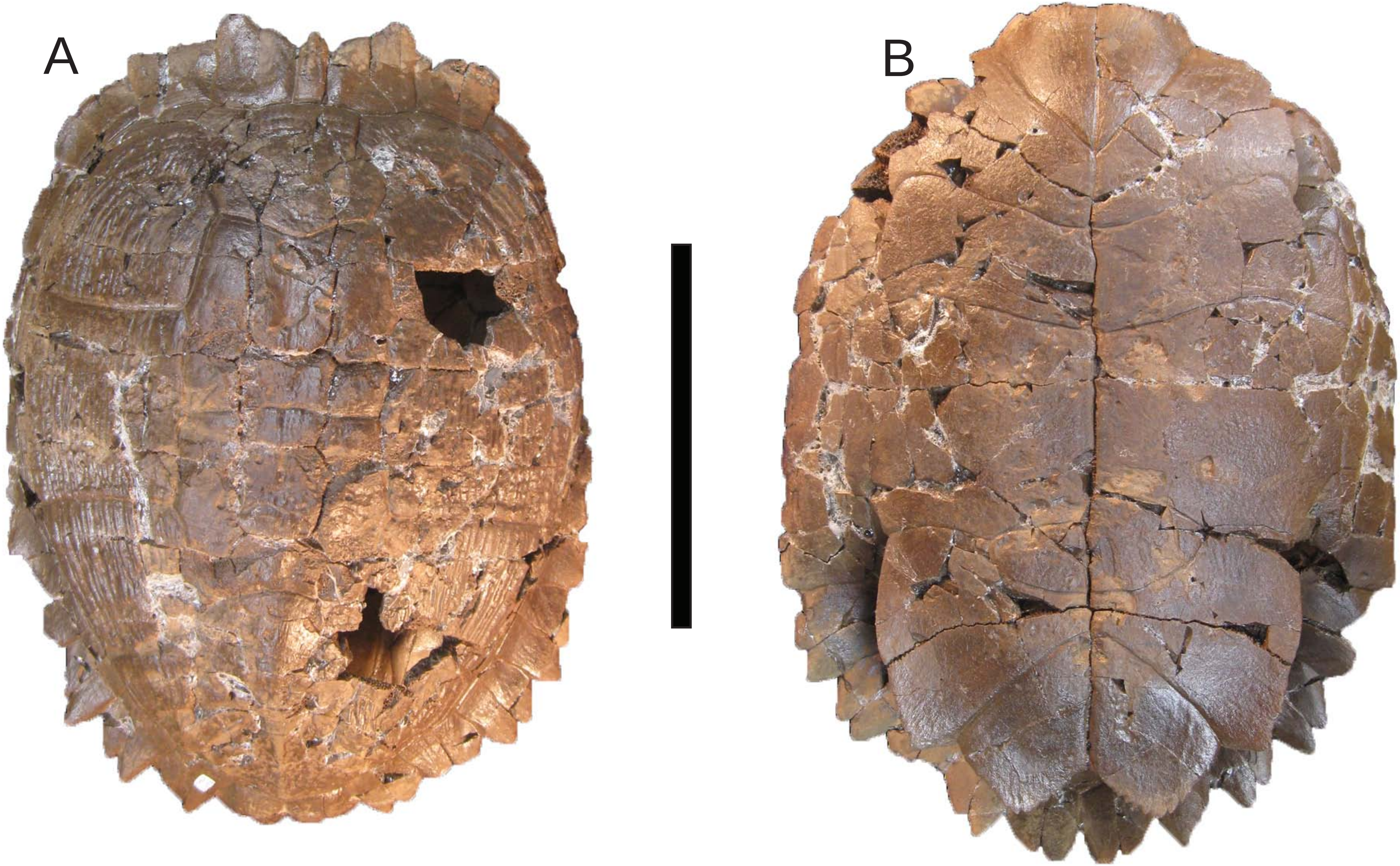

Figure S13. ***Trachemys haugrudi*, paratype nearly complete shell (ETMNH–11643).**

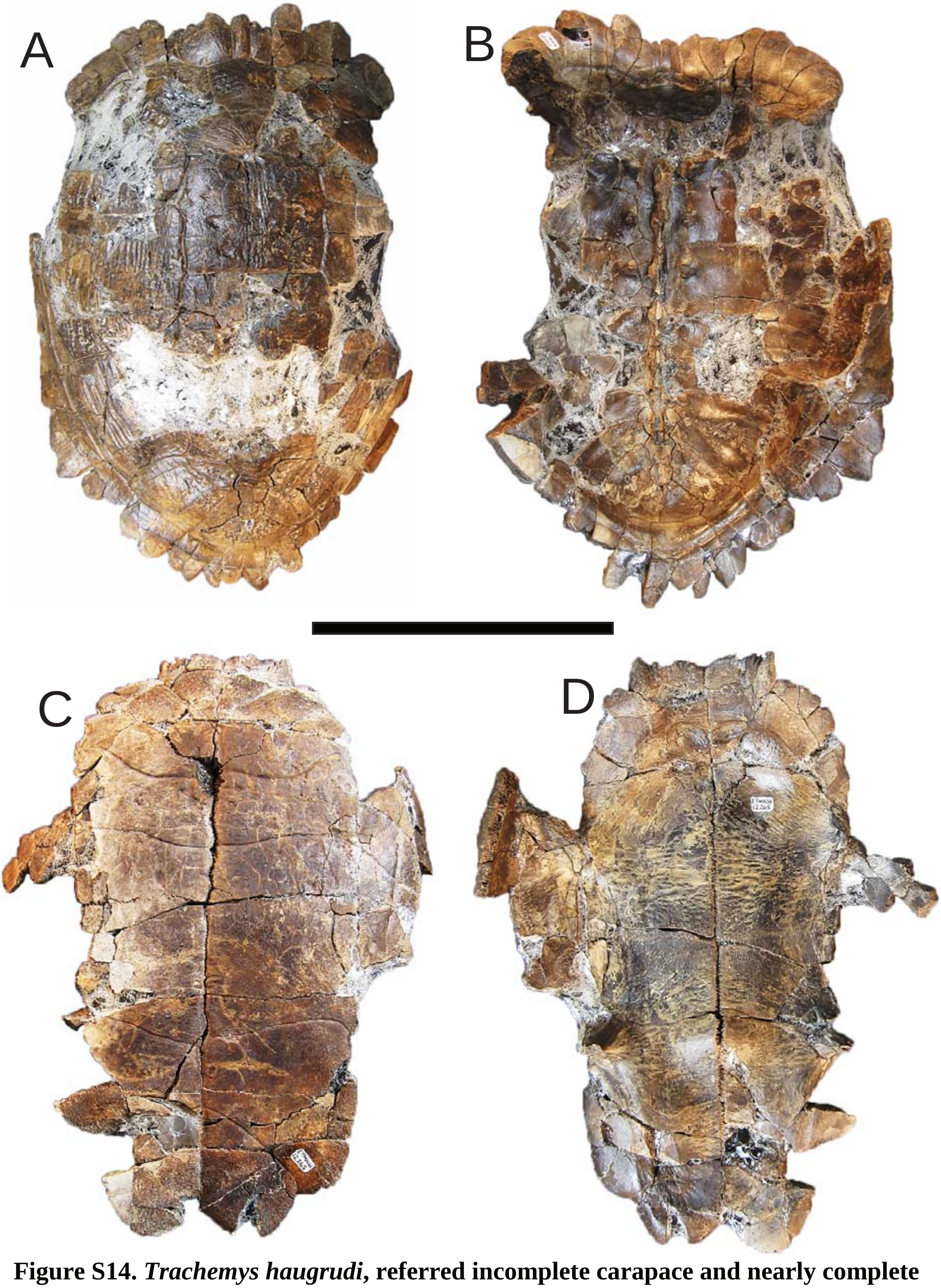

Figure S14. ***Trachemys haugrudi*, referred incomplete carapace and nearly complete plastron (ETMNH–12265).**

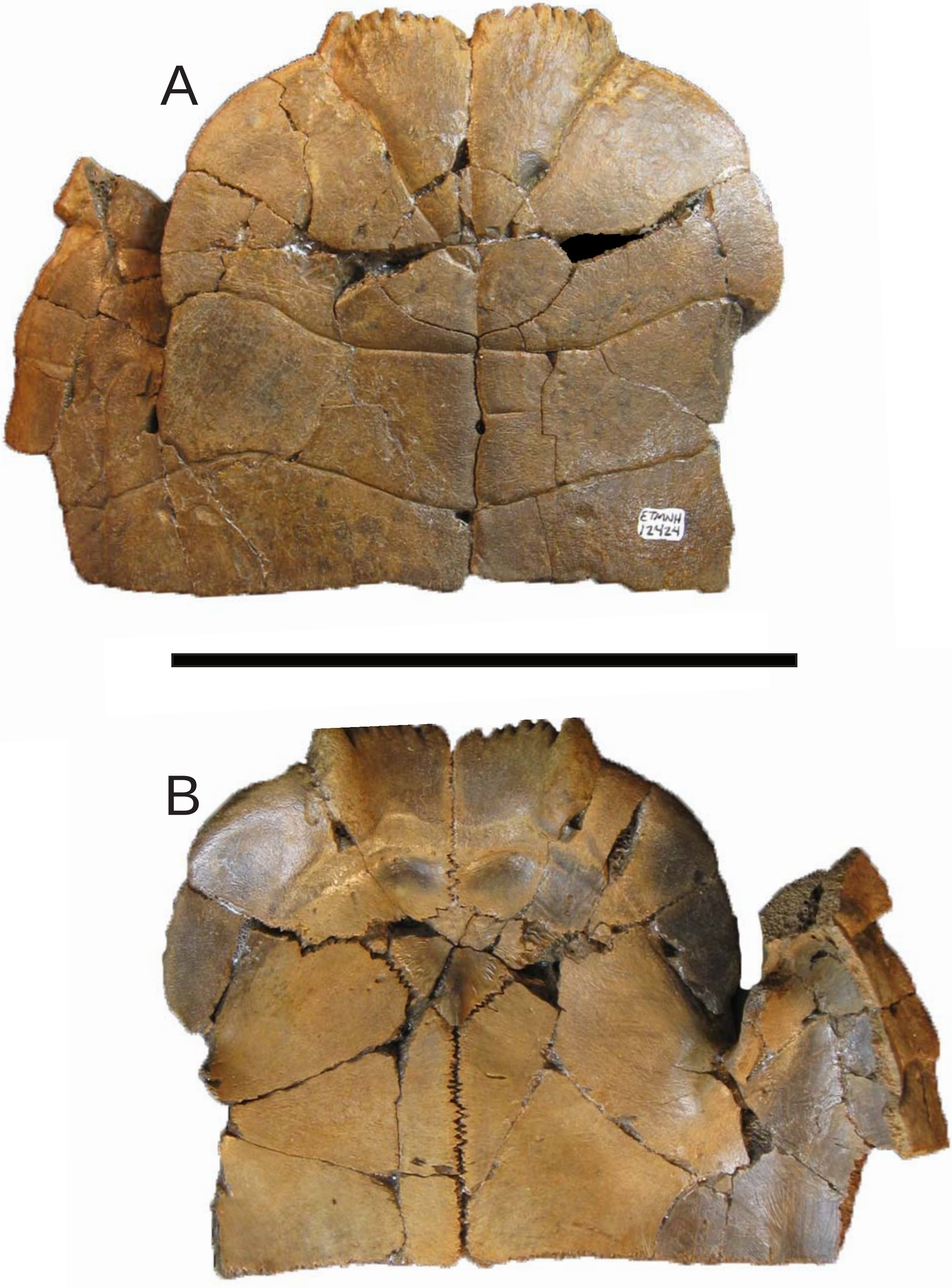

Figure S15. ***Trachemys haugrudi*, referred anterior plastron (ETMNH–12424).**

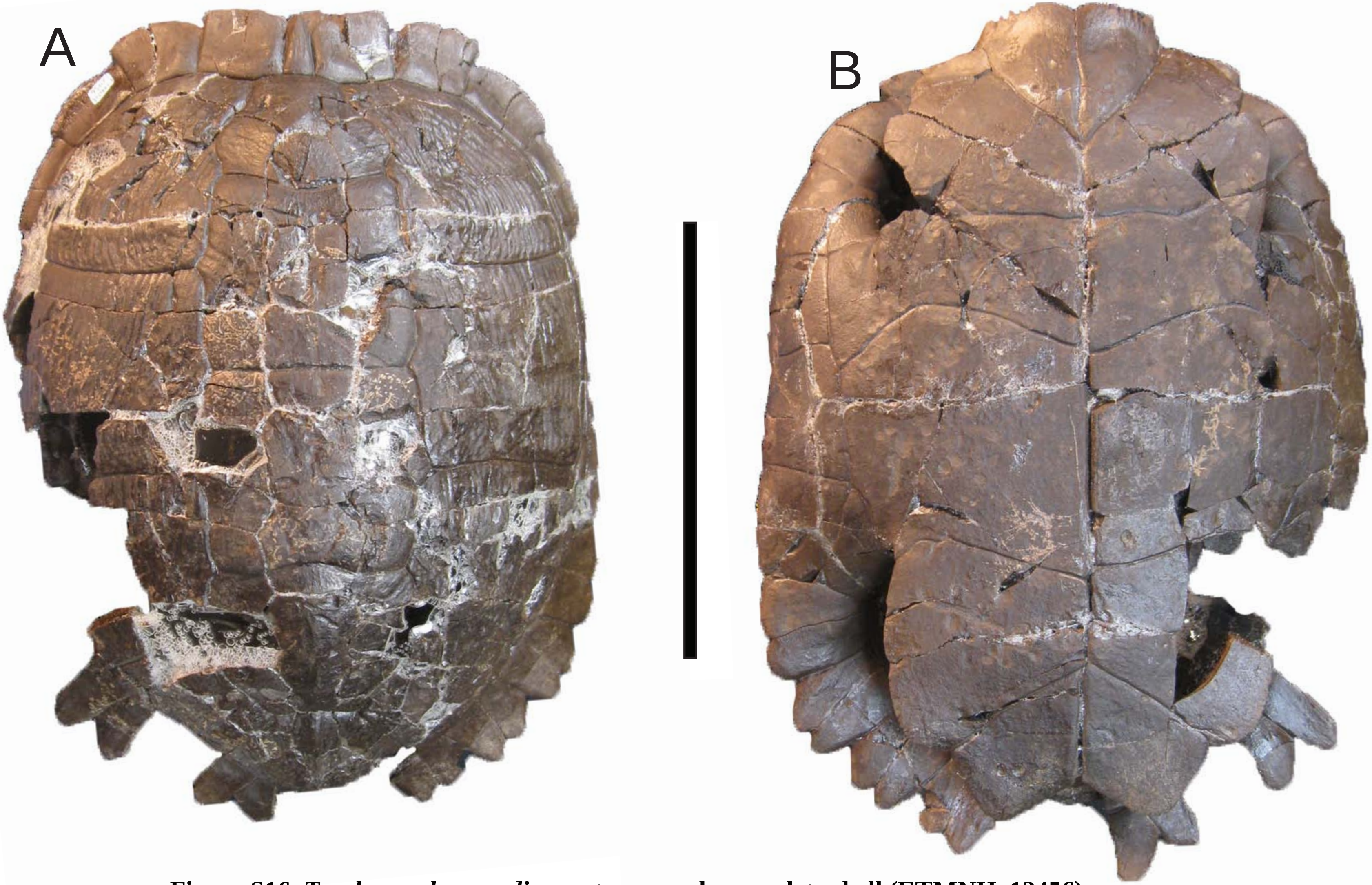

Figure S16. ***Trachemys haugrudi*****, paratype nearly complete shell (ETMNH–12456).**

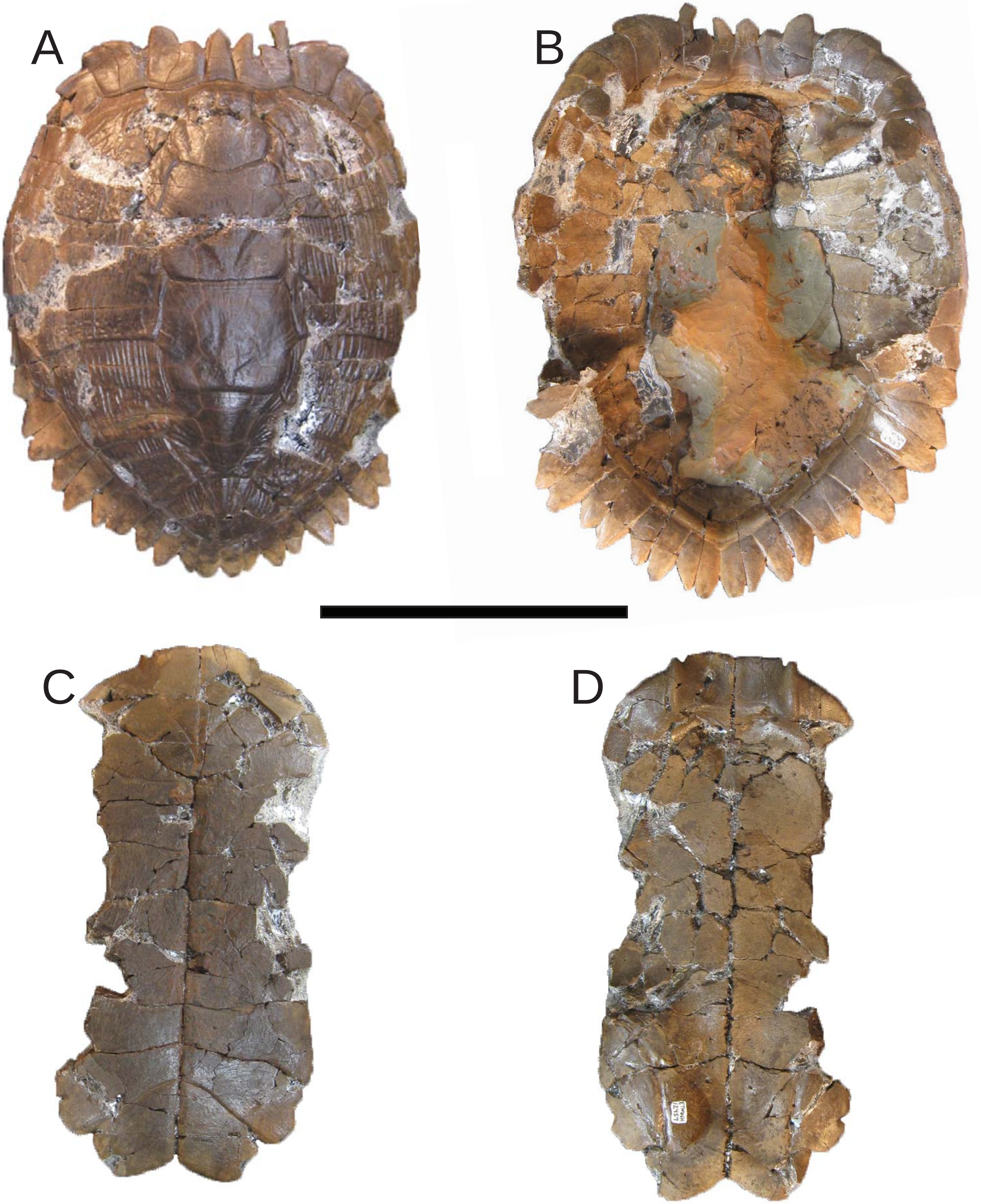

Figure S17. ***Trachemys haugrudi*, paratype nearly complete carapace and plastron (ETMNH–12457).**

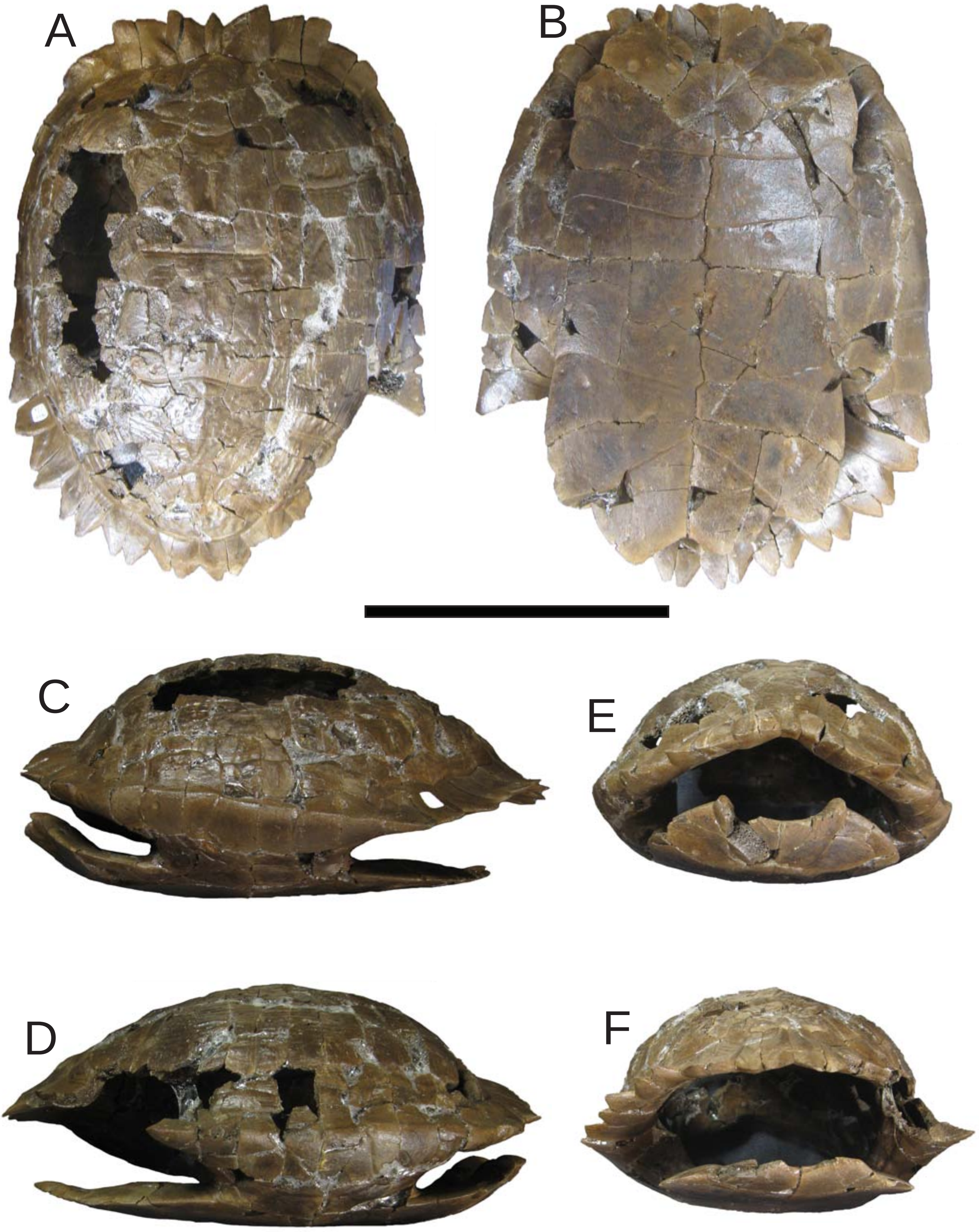

Figure S18. ***Trachemys haugrudi*, paratype nearly complete shell (ETMNH–12726).**

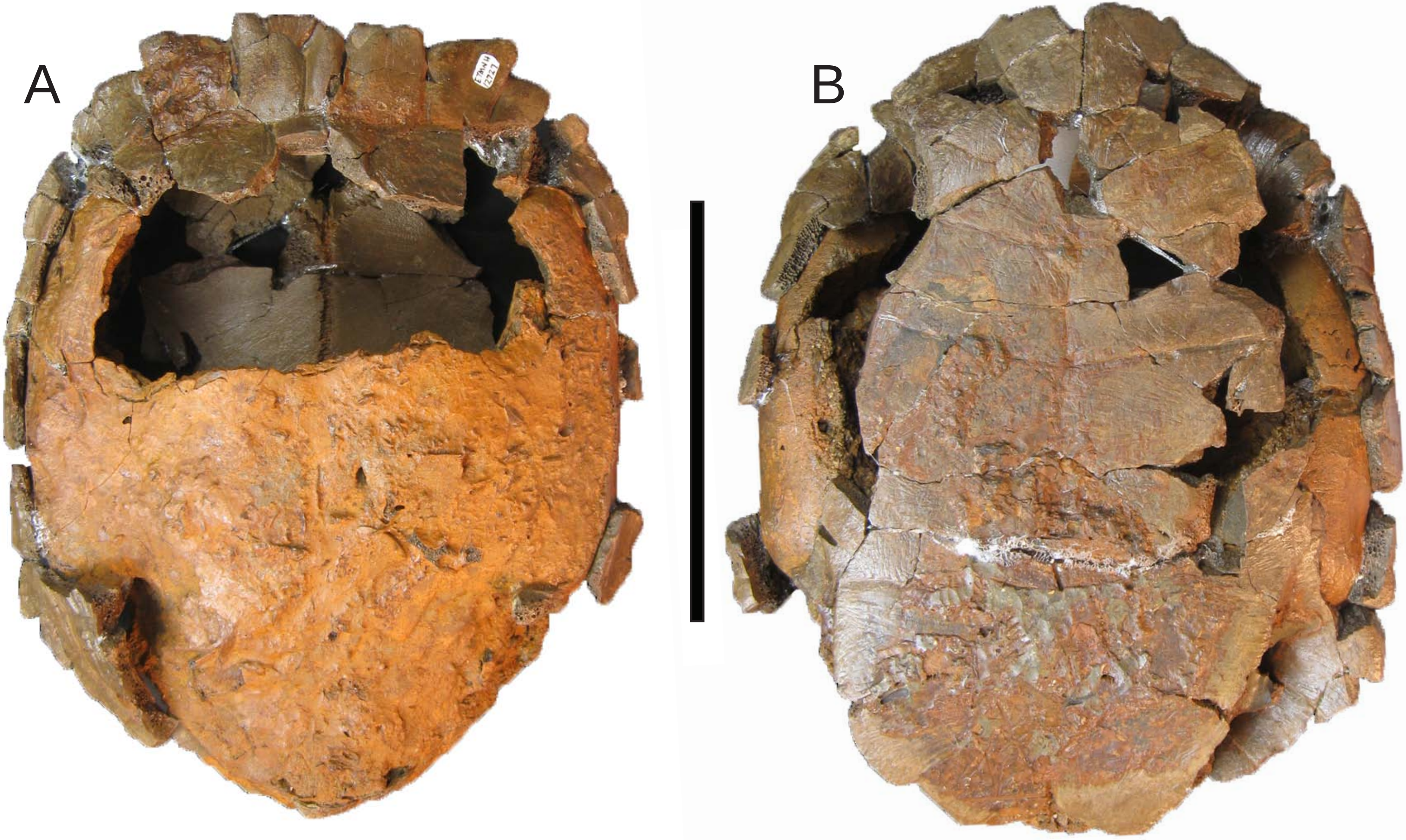

Figure S19. ***Trachemys haugrudi*****, referred incomplete shell (ETMNH–12727).**

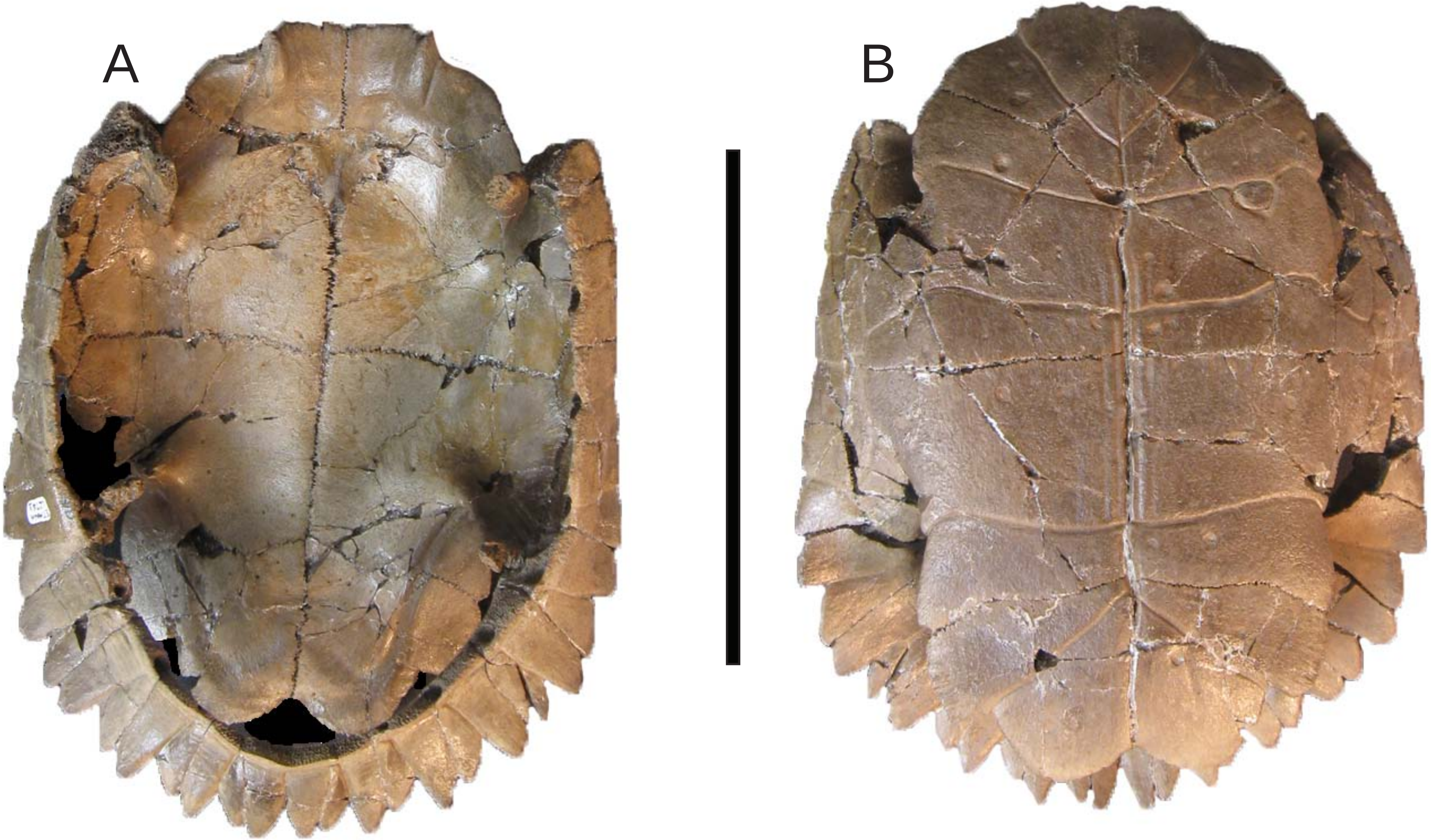

Figure S20. ***Trachemys haugrudi*****, paratype incomplete juvenile shell (ETMNH–12753).**

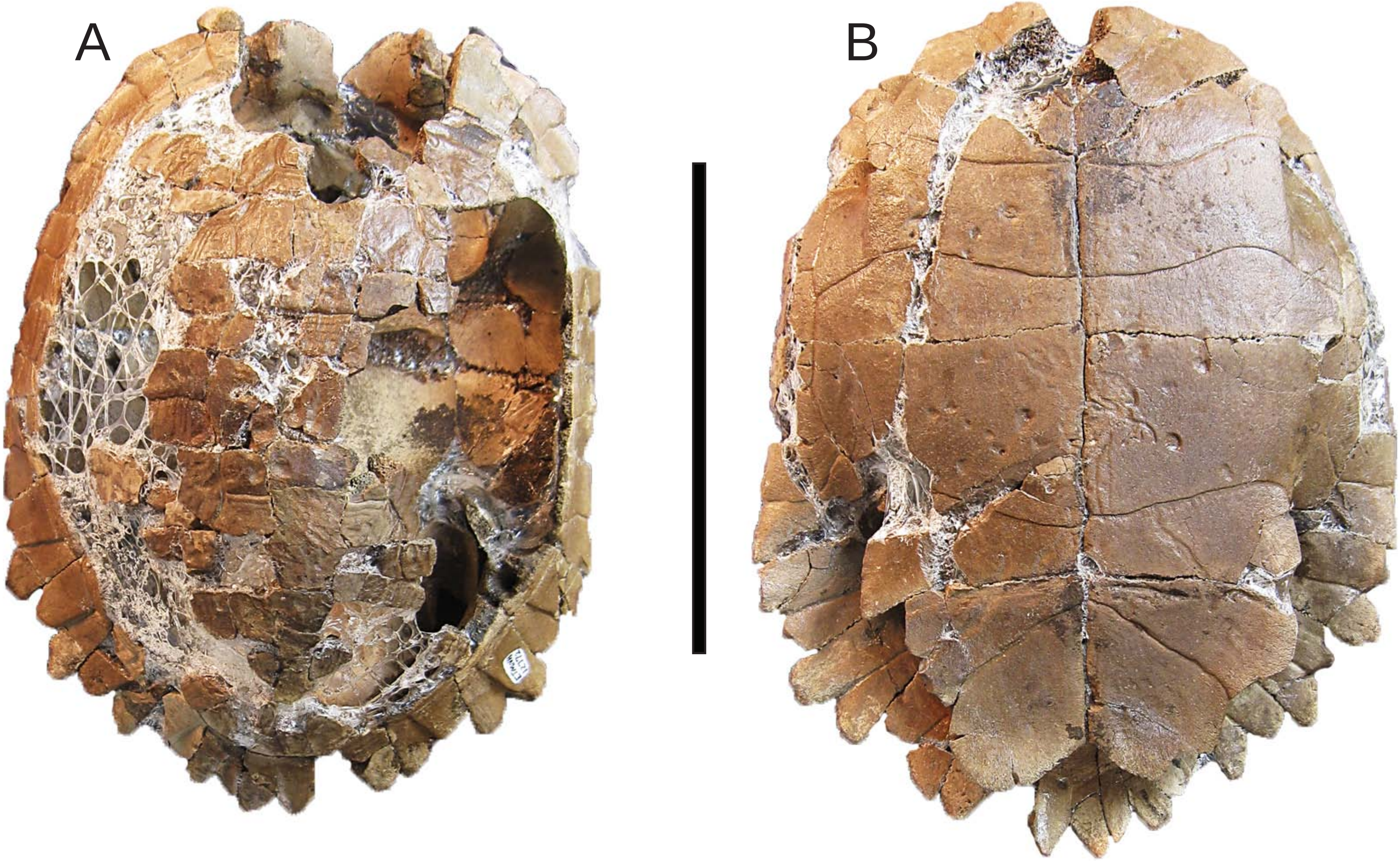

Figure S21. ***Trachemys haugrudi*, referred nearly complete shell (ETMNH–12772).**

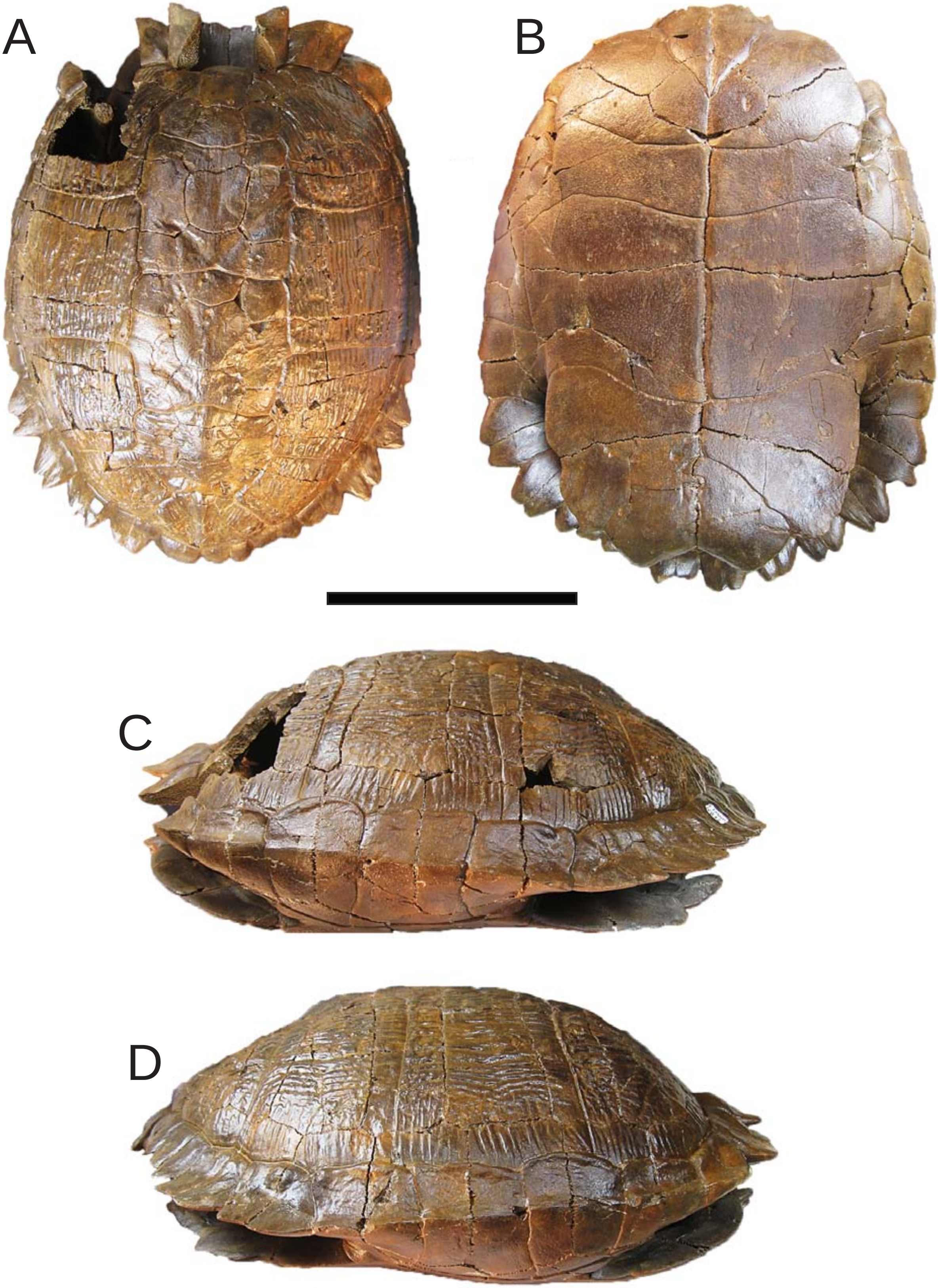

Figure S22. ***Trachemys haugrudi*, paratype nearly complete shell (ETMNH–12832).**

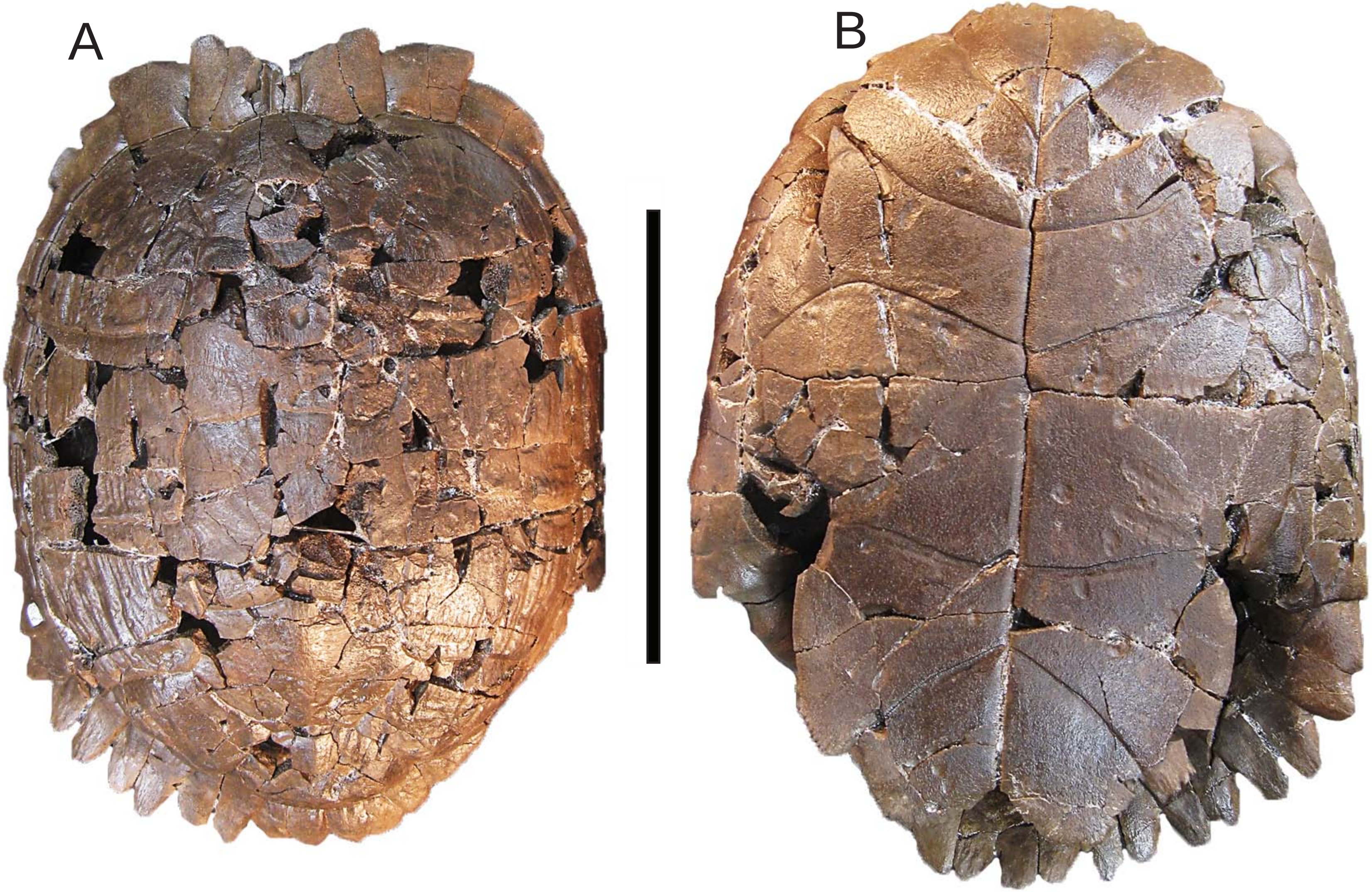

Figure S23. ***Trachemys haugrudi*****, paratype nearly complete shell (ETMNH–12833).**

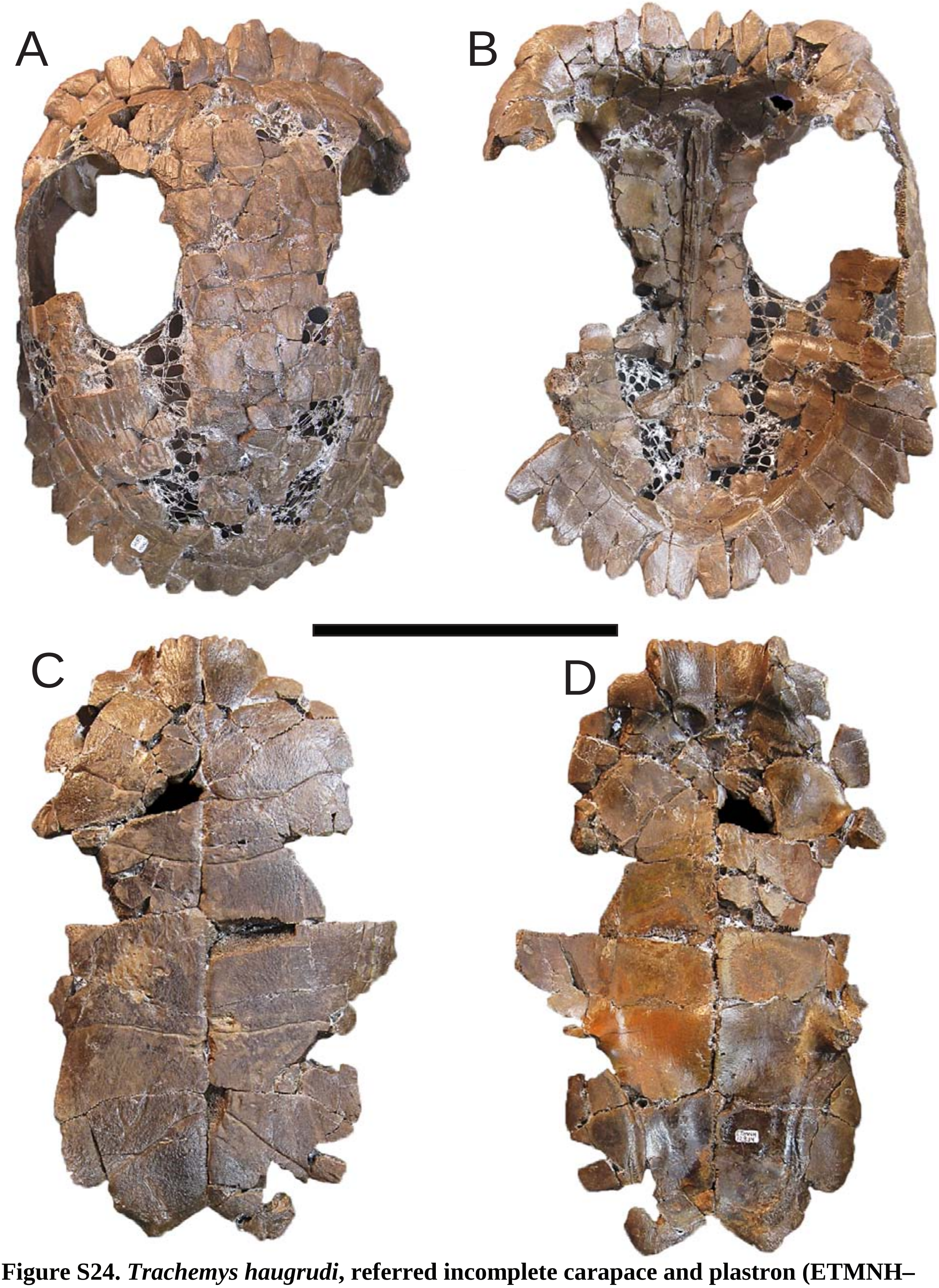

Figure S24. ***Trachemys haugrudi*, referred incomplete carapace and plastron (ETMNH–12834).**

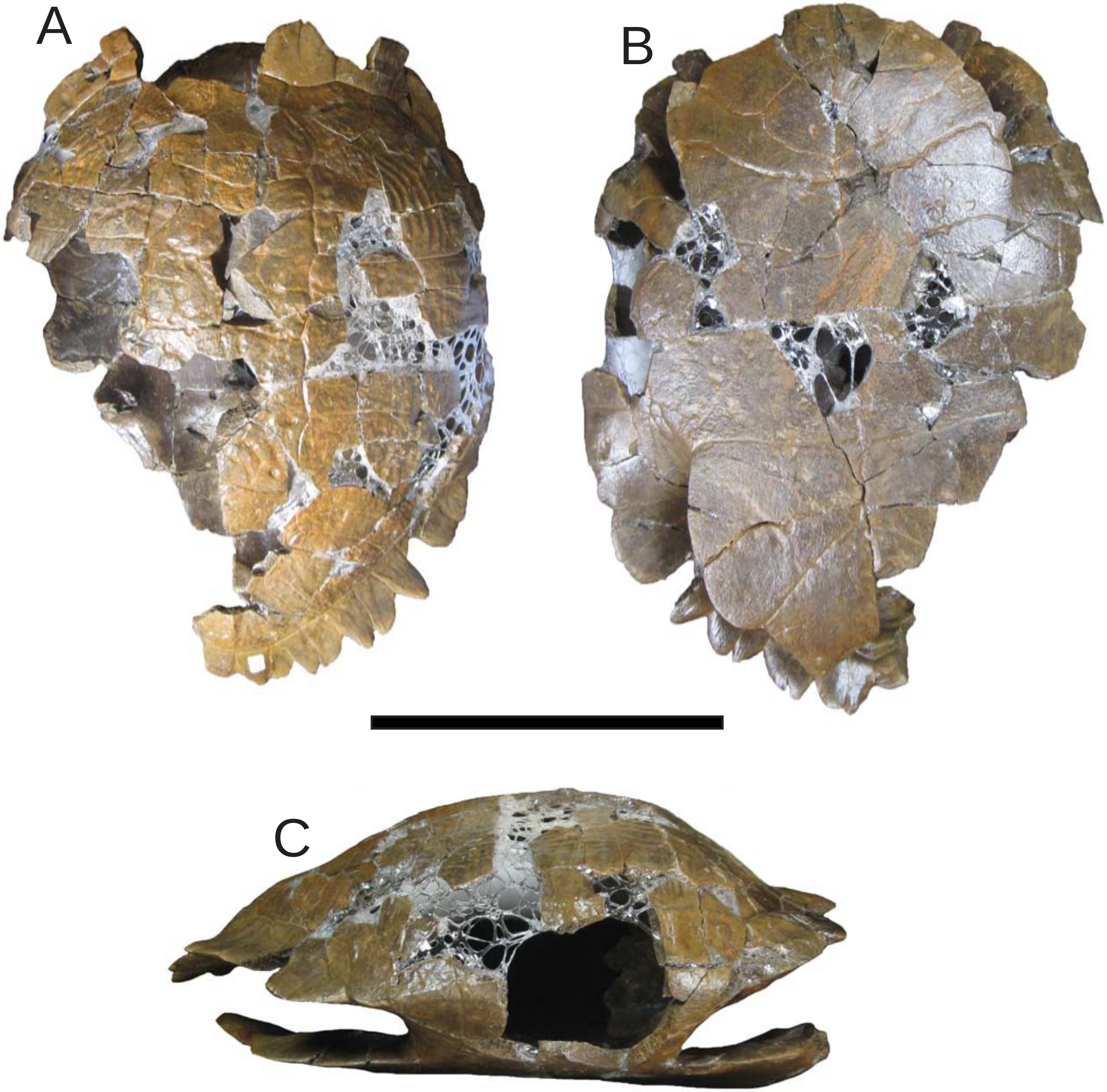

Figure S25. ***Trachemys haugrudi*****, referred incomplete shell (ETMNH–12979).**

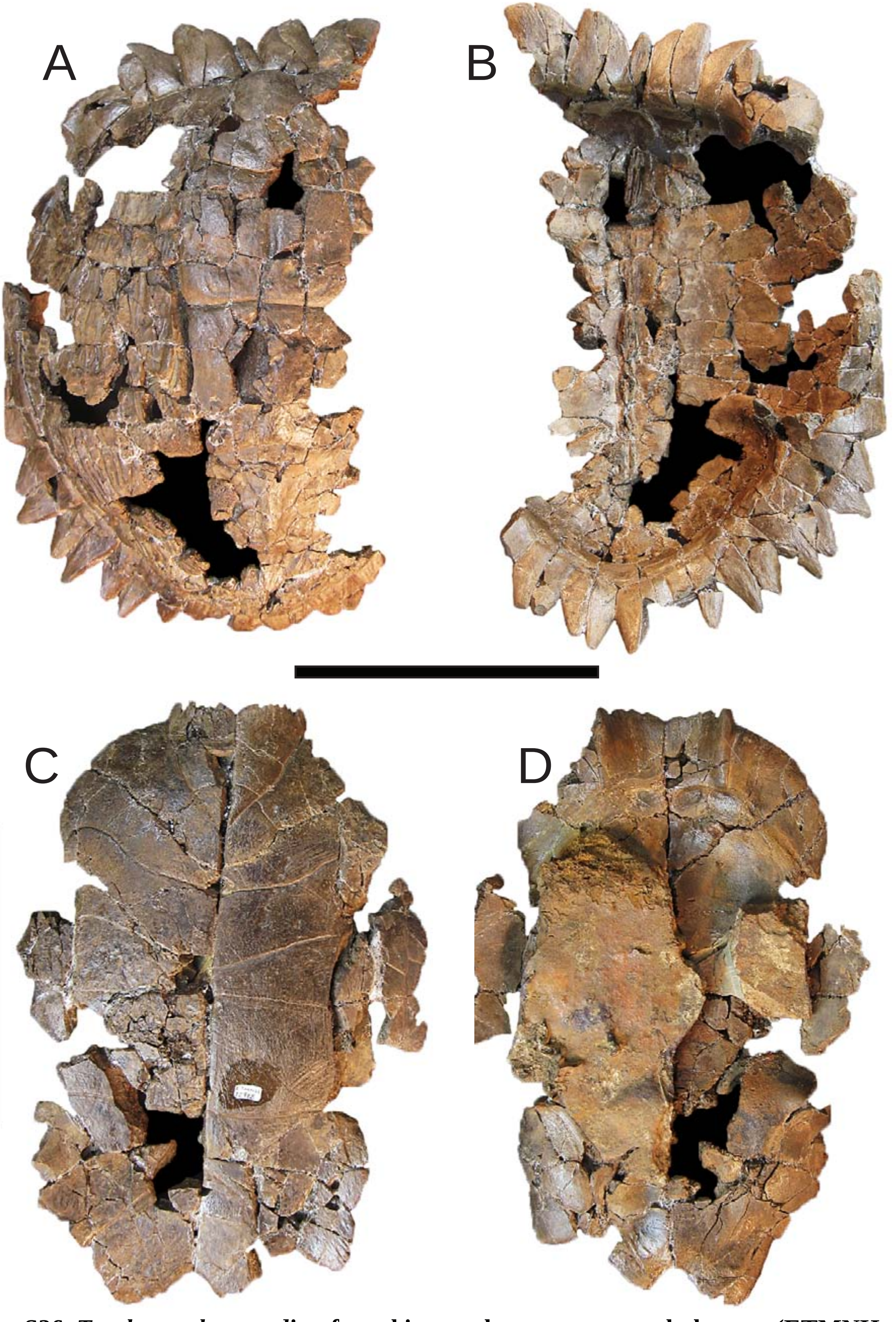

Figure S26. ***Trachemys haugrudi*****, referred incomplete carapace and plastron (ETMNH–12988).**

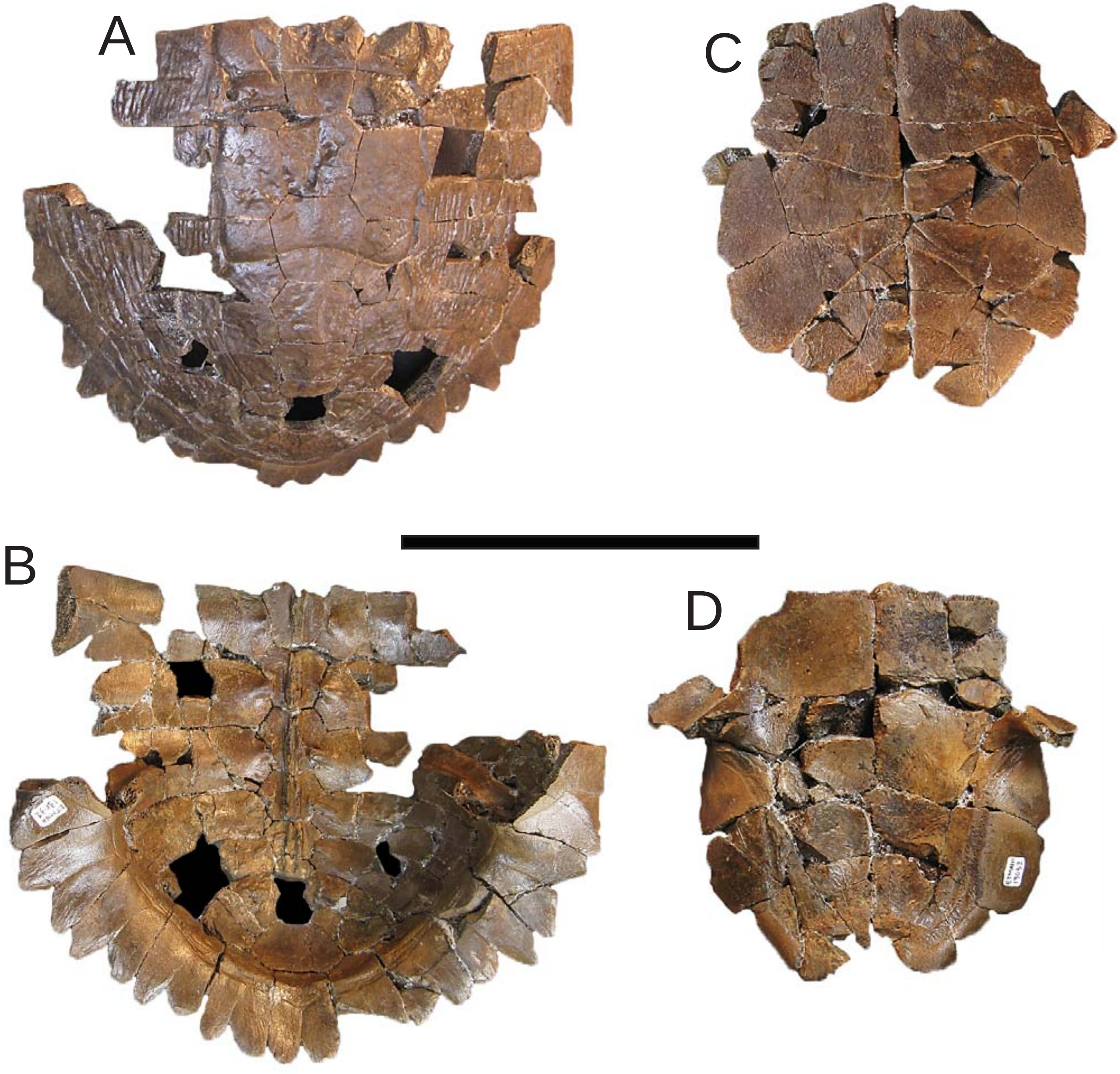

Figure S27. ***Trachemys haugrudi*, referred incomplete carapace and plastron (ETMNH–13032).**

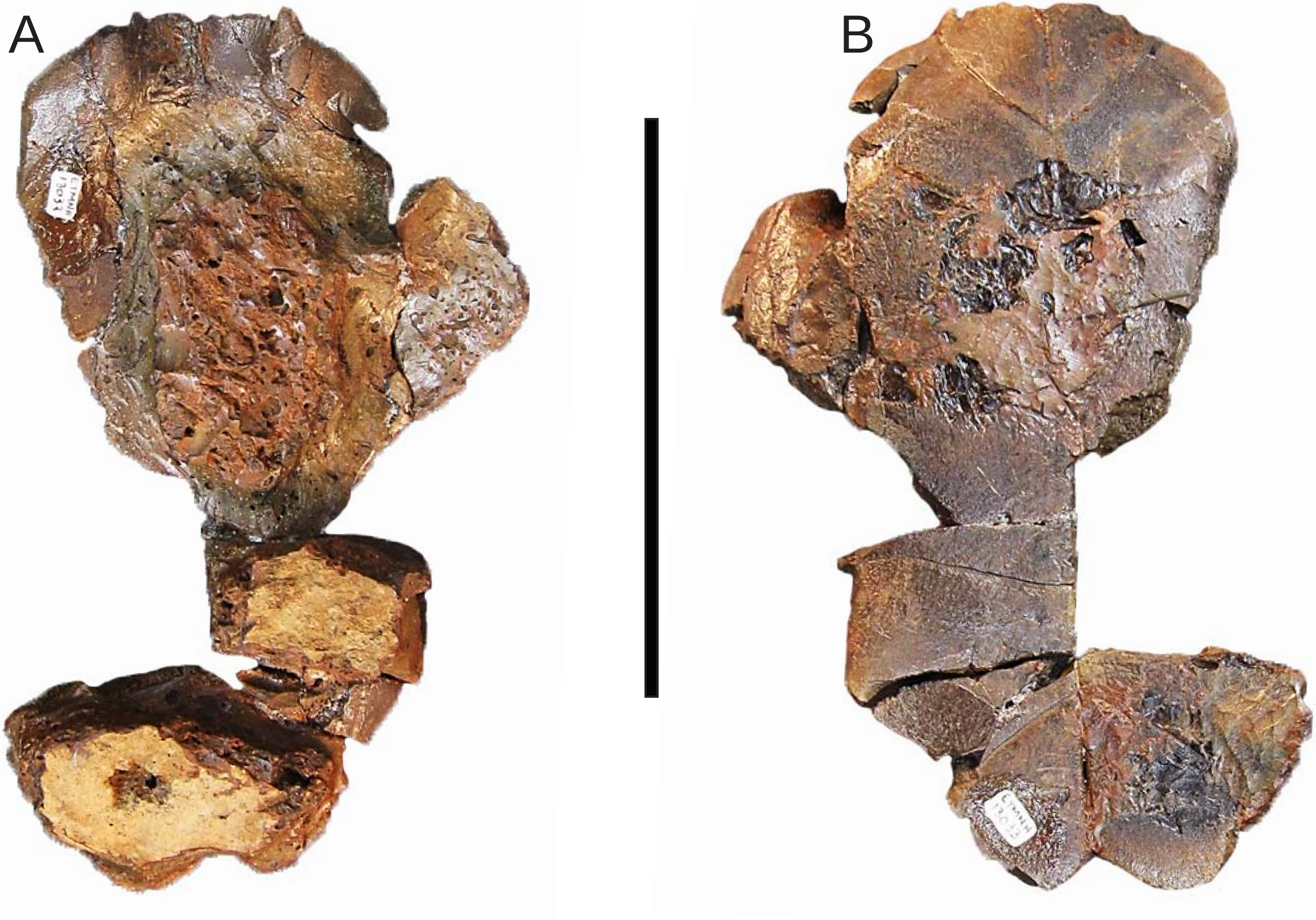

Figure S28. ***Trachemys haugrudi*, referred incomplete plastron (ETMNH–13033).**

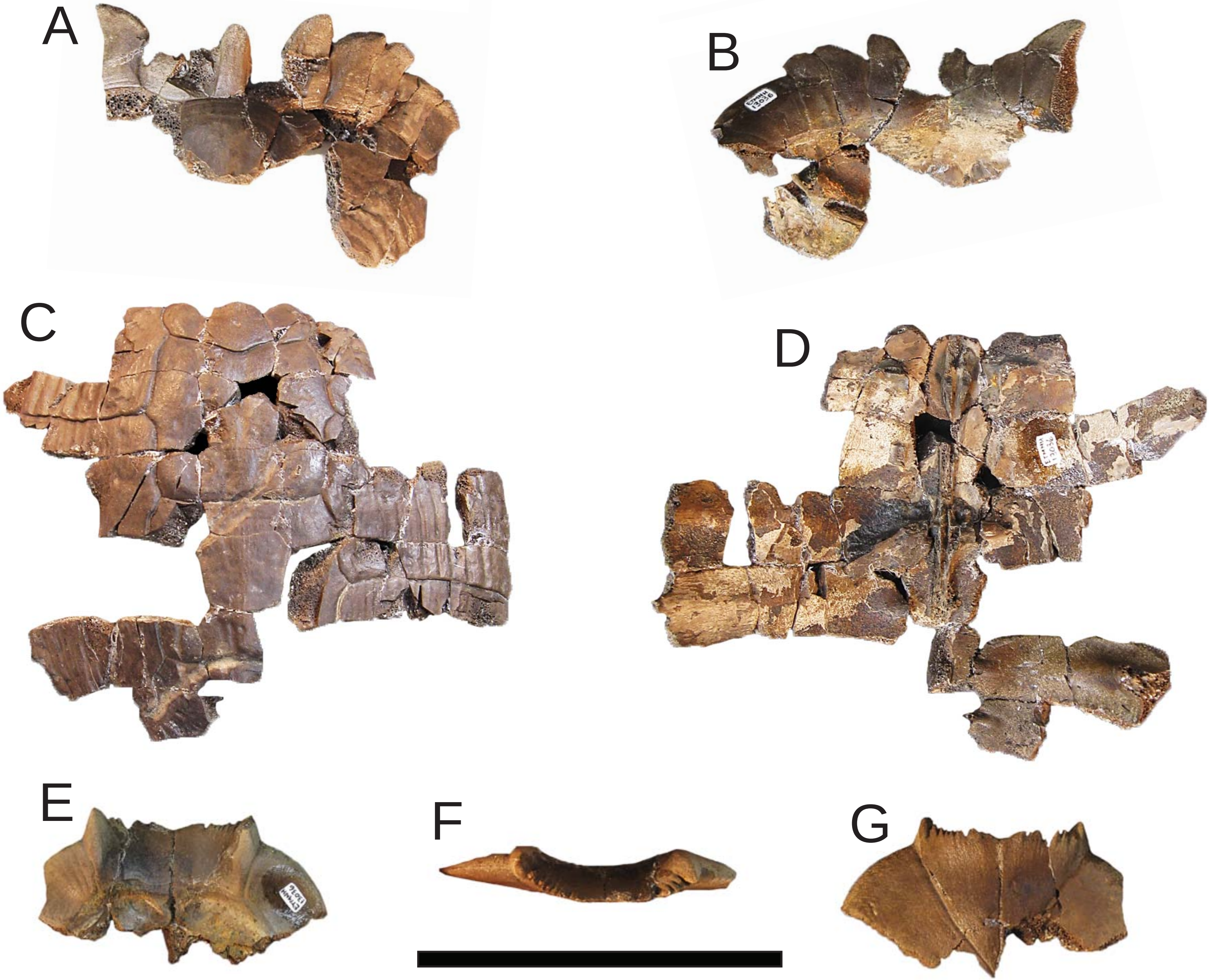

Figure S29. ***Trachemys haugrudi*, referred incomplete carapace and plastron (ETMNH–13036).**

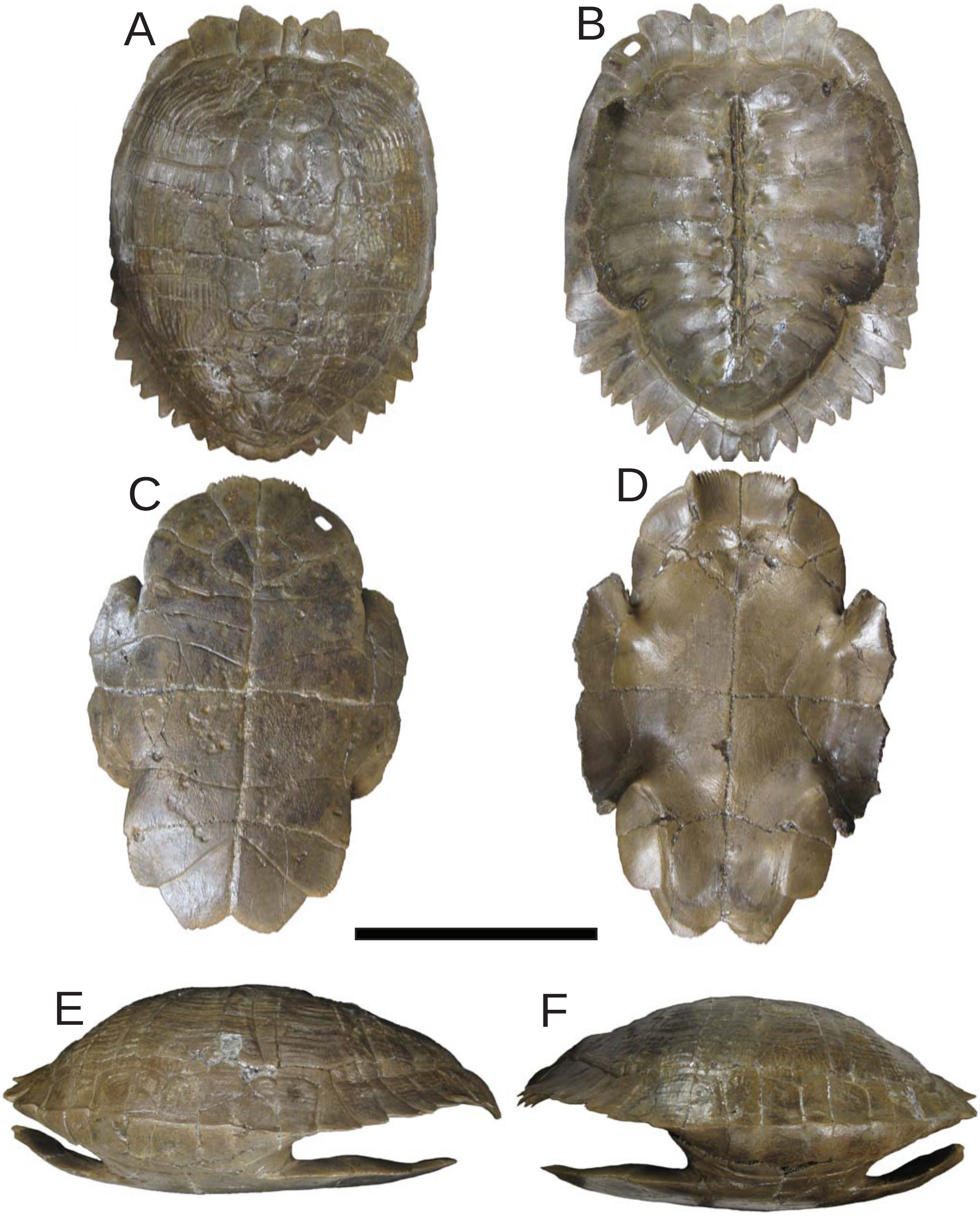

Figure S30. ***Trachemys haugrudi*****, paratype complete carapace and plastron (ETMNH–14362).**

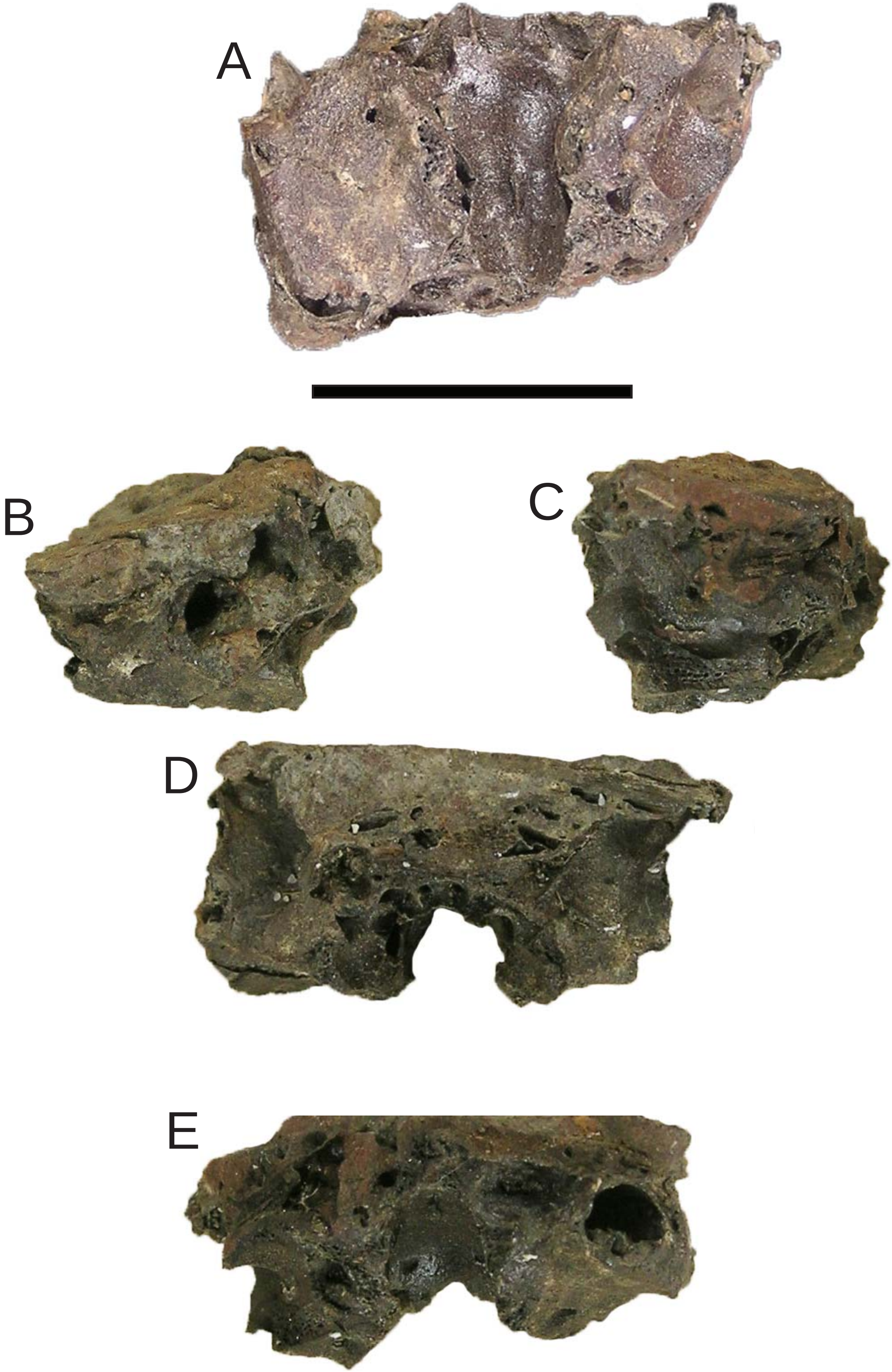

Figure S31. ***Trachemys haugrudi*, paratype incomplete skull (ETMNH–7690).**

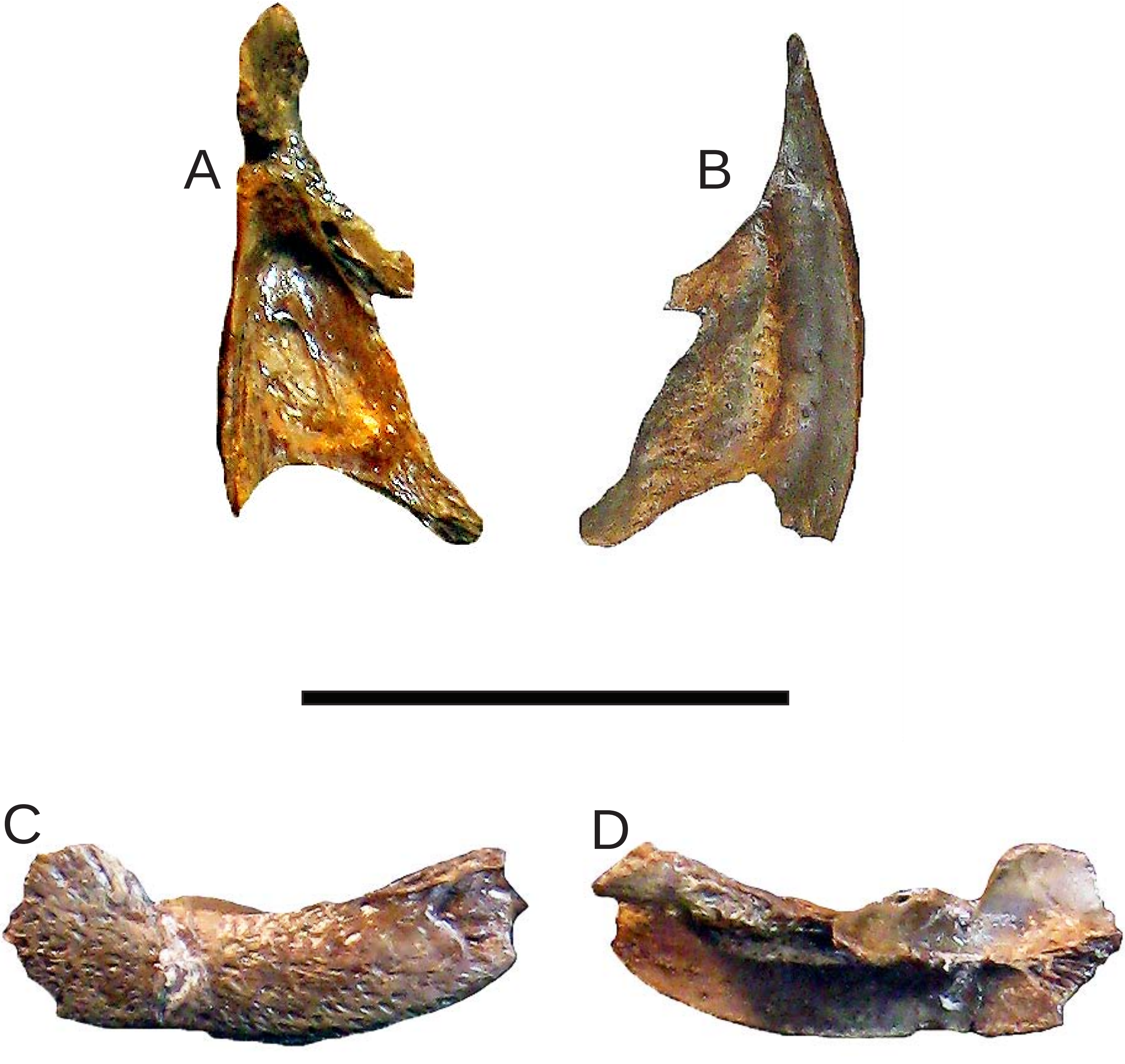

Figure S32. ***Trachemys haugrudi*****, paratype left maxilla (ETMNH–12457).**

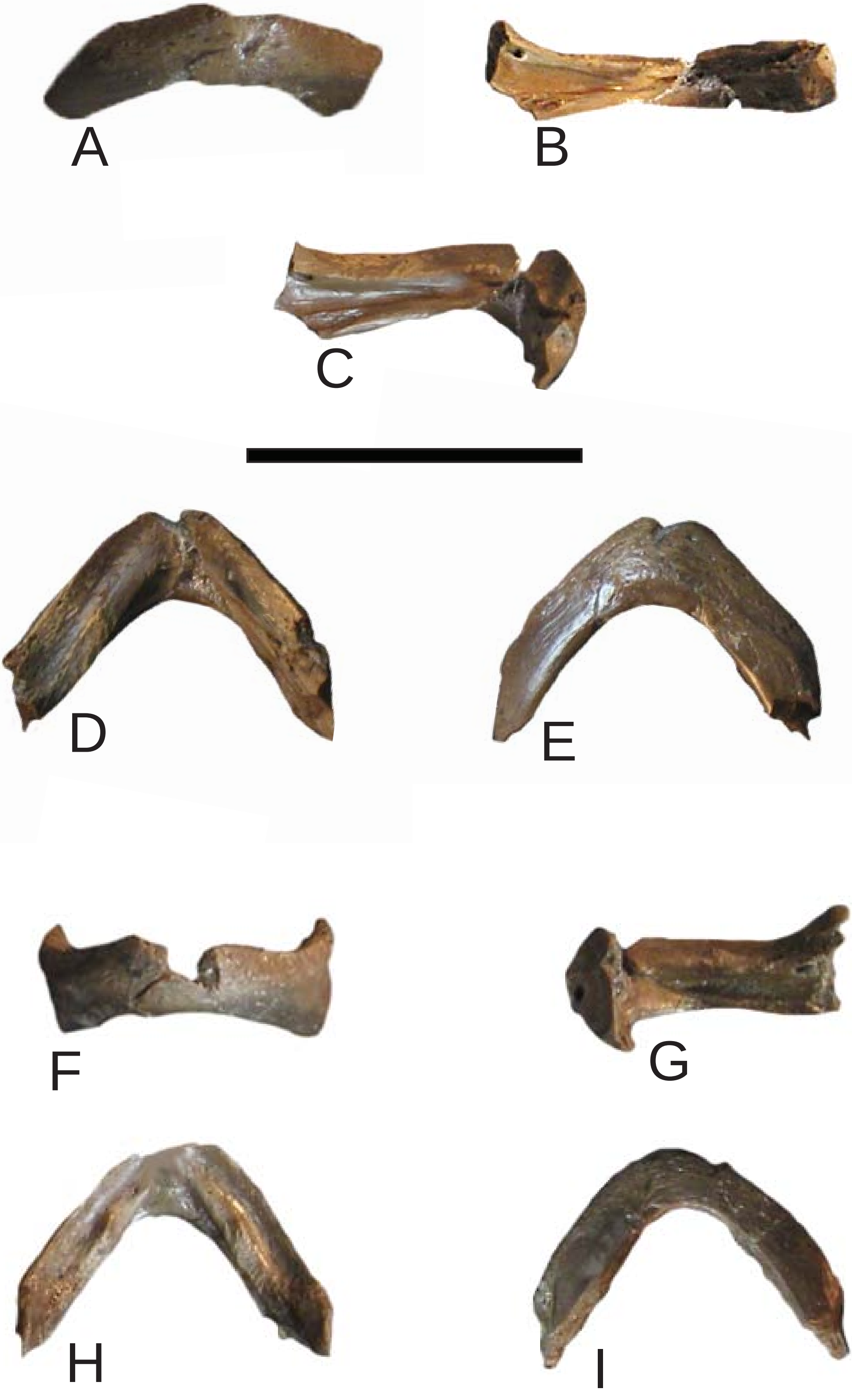

Figure S33. ***Trachemys haugrudi*****, lower jaws.**

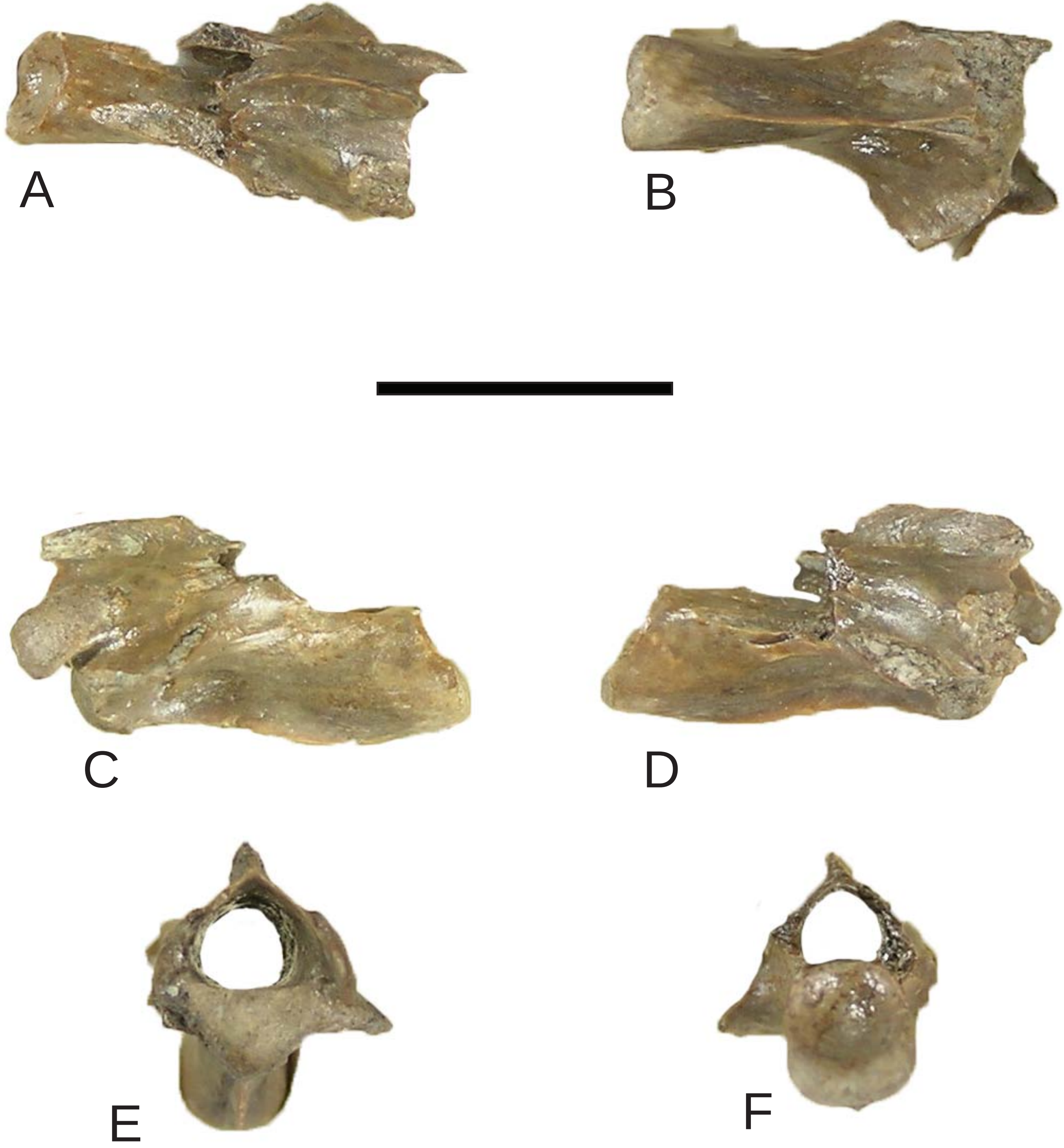

Figure S34. ***Trachemys haugrudi*, holotype nearly complete cervical vertebra II (= axis, ETMNH–8549).**

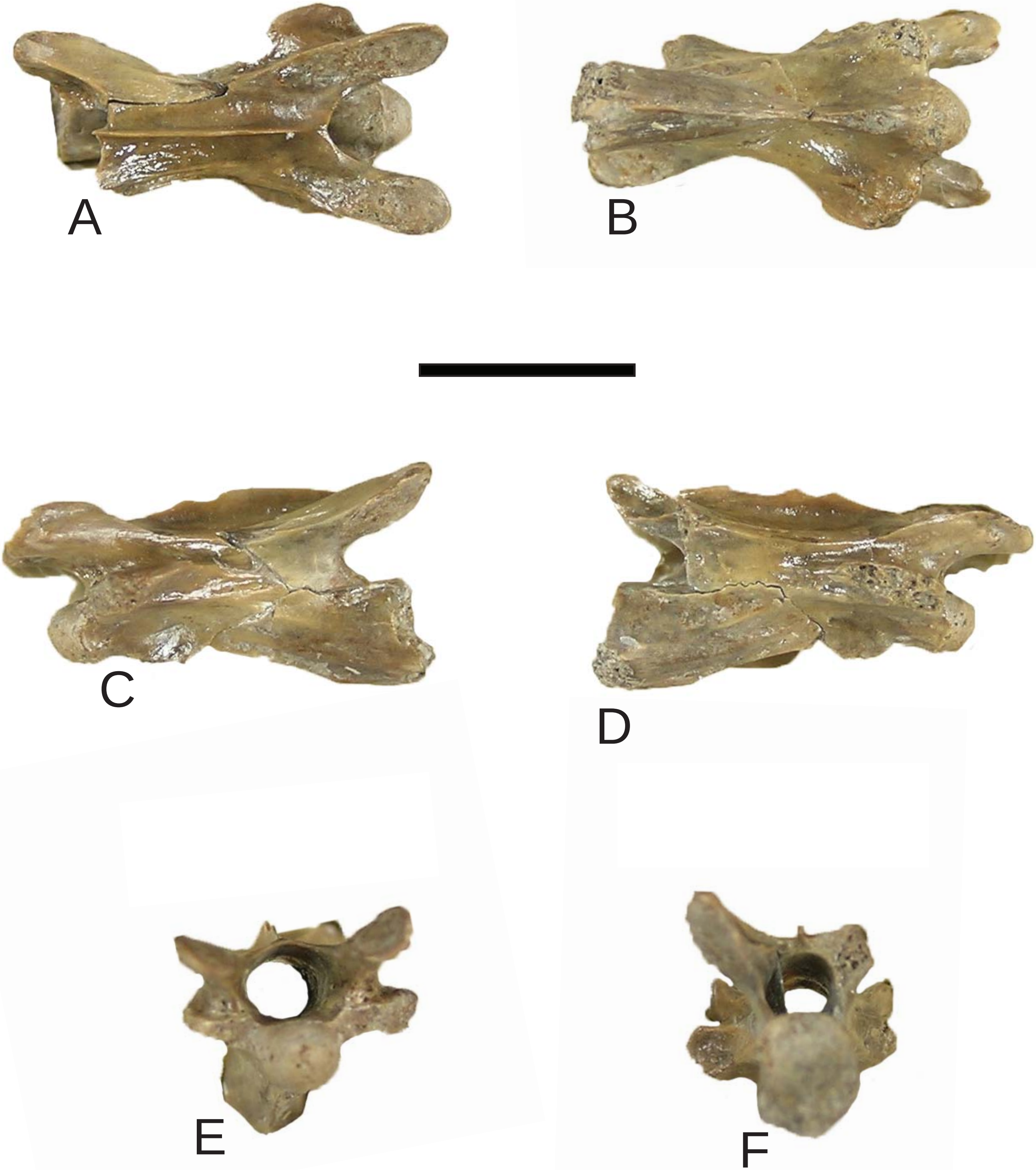

Figure S35. ***Trachemys haugrudi*****, holotype nearly complete cervical vertebra III (ETMNH–8549).**

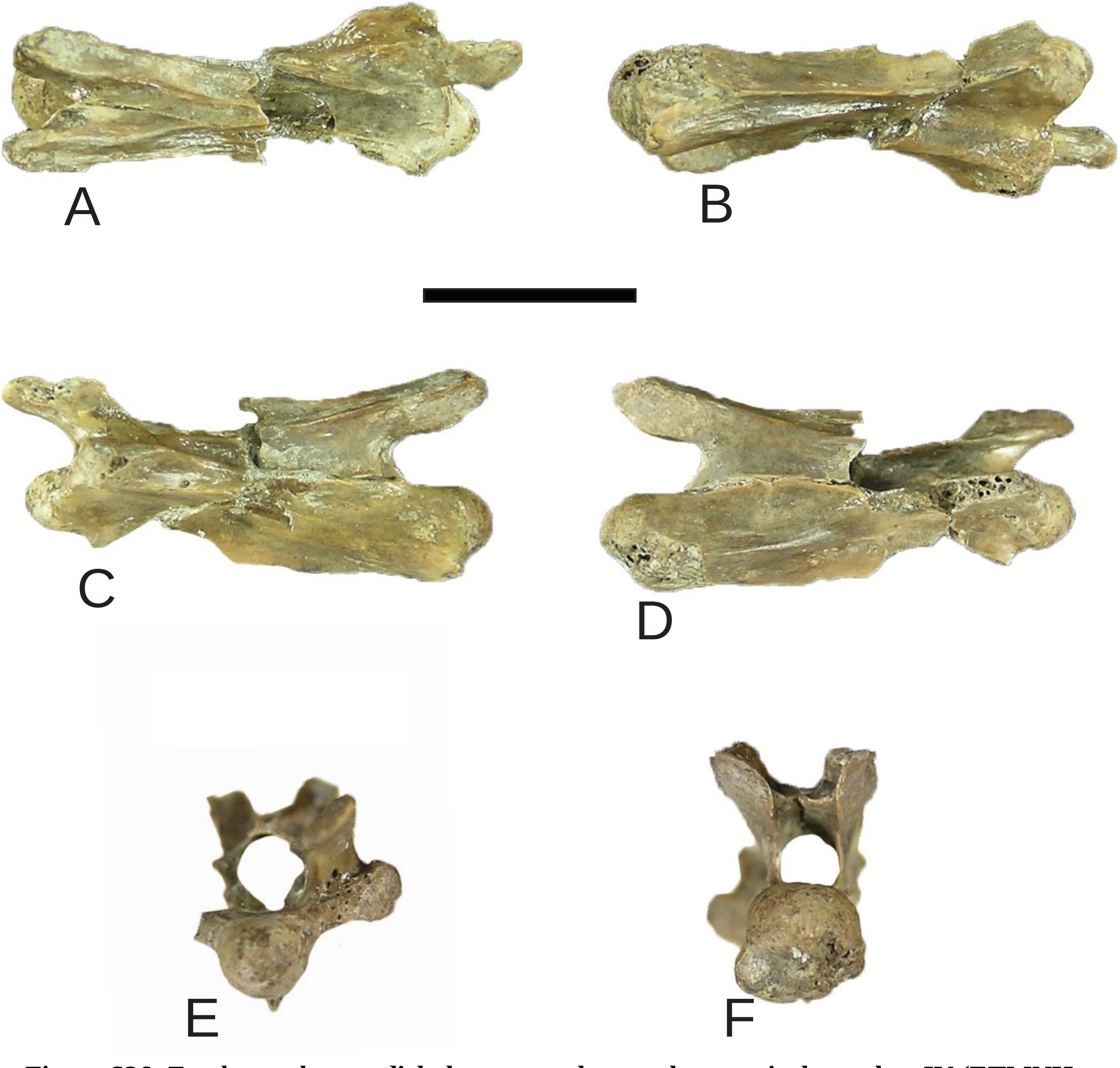

Figure S36. ***Trachemys haugrudi*, holotype nearly complete cervical vertebra IV (ETMNH–8549).**

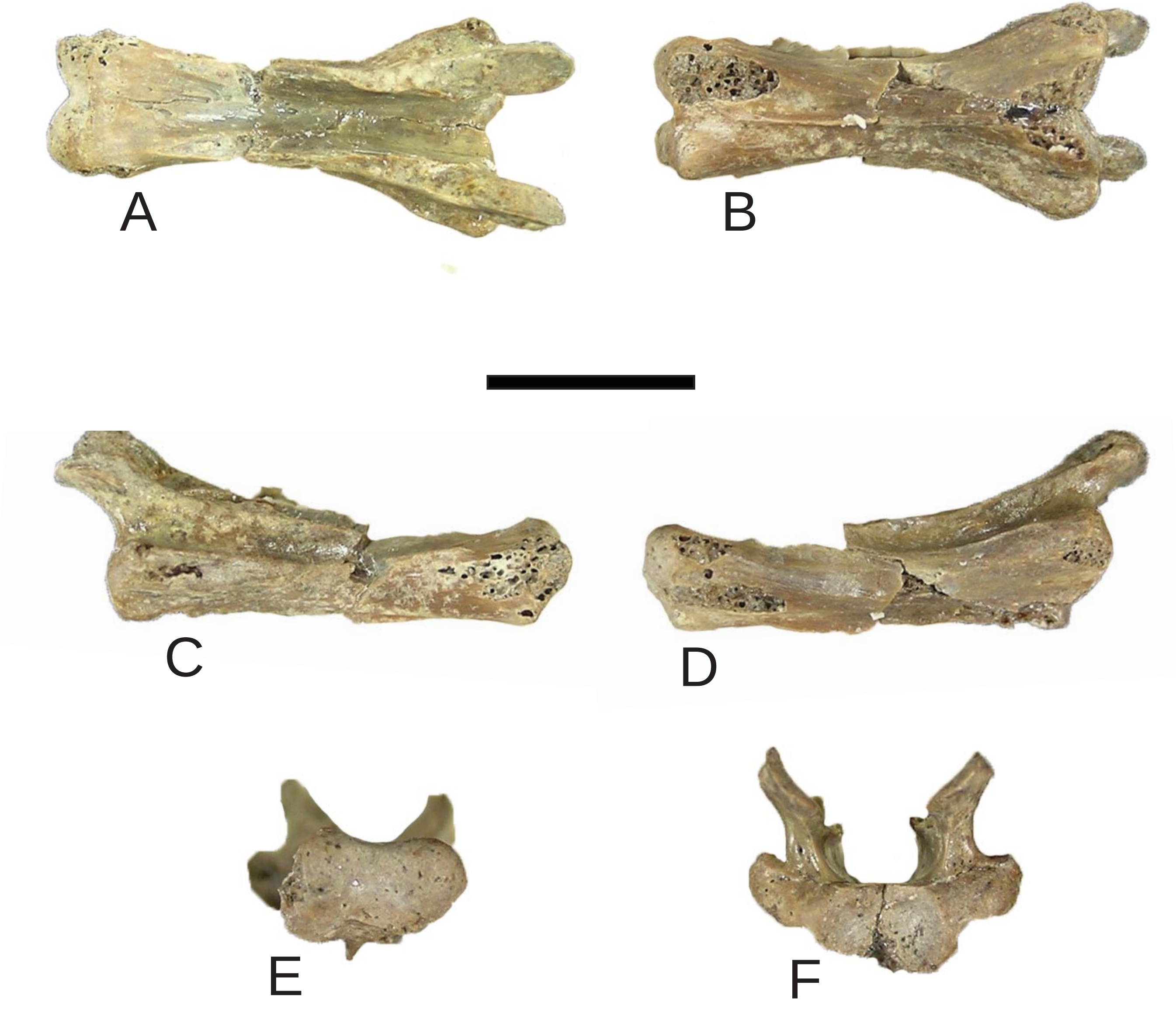

Figure S37. ***Trachemys haugrudi*, holotype nearly complete cervical vertebra V (ETMNH–8549).**

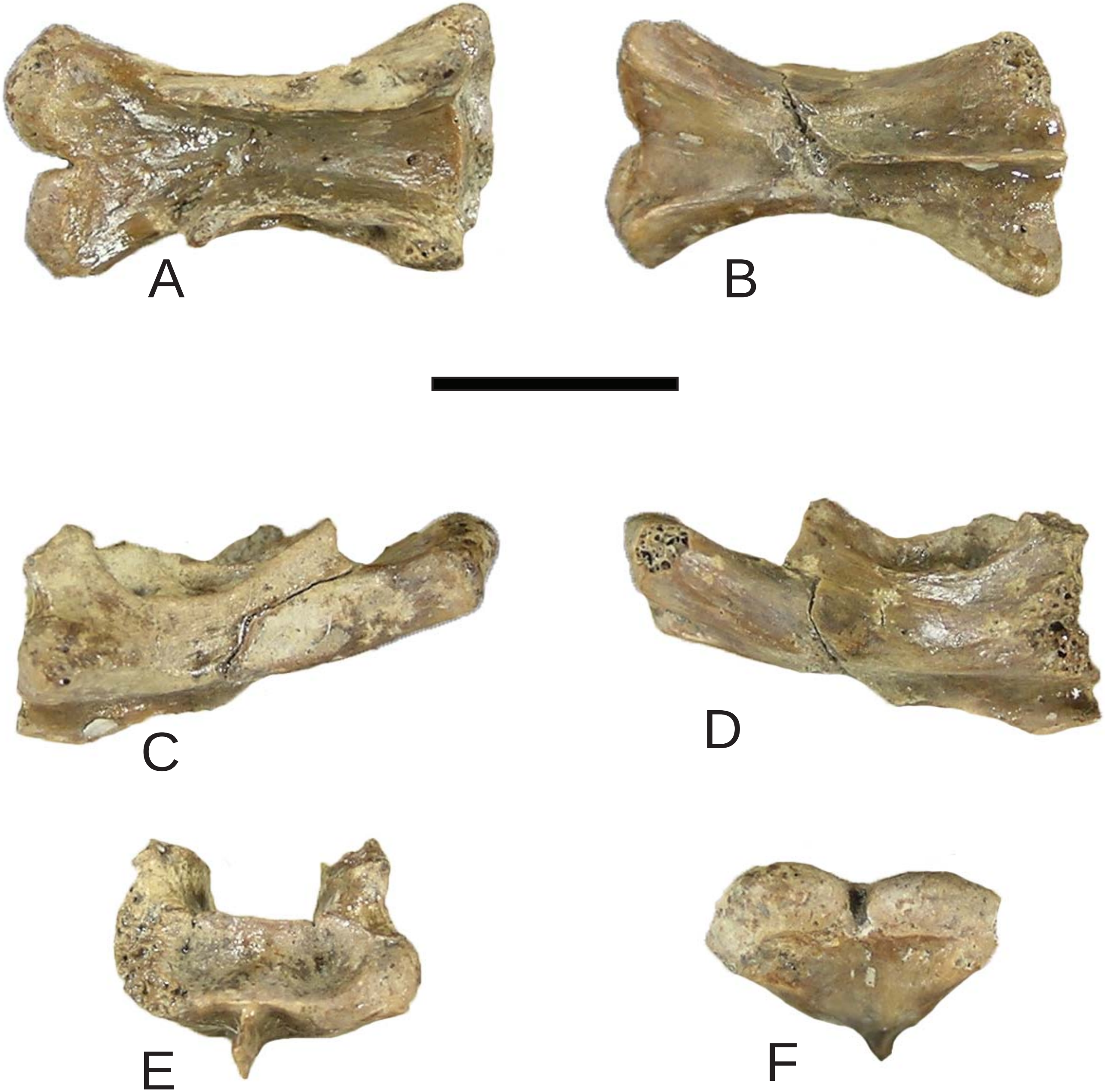

Figure S38. ***Trachemys haugrudi*, holotype nearly complete cervical vertebra VI (ETMNH–8549).**

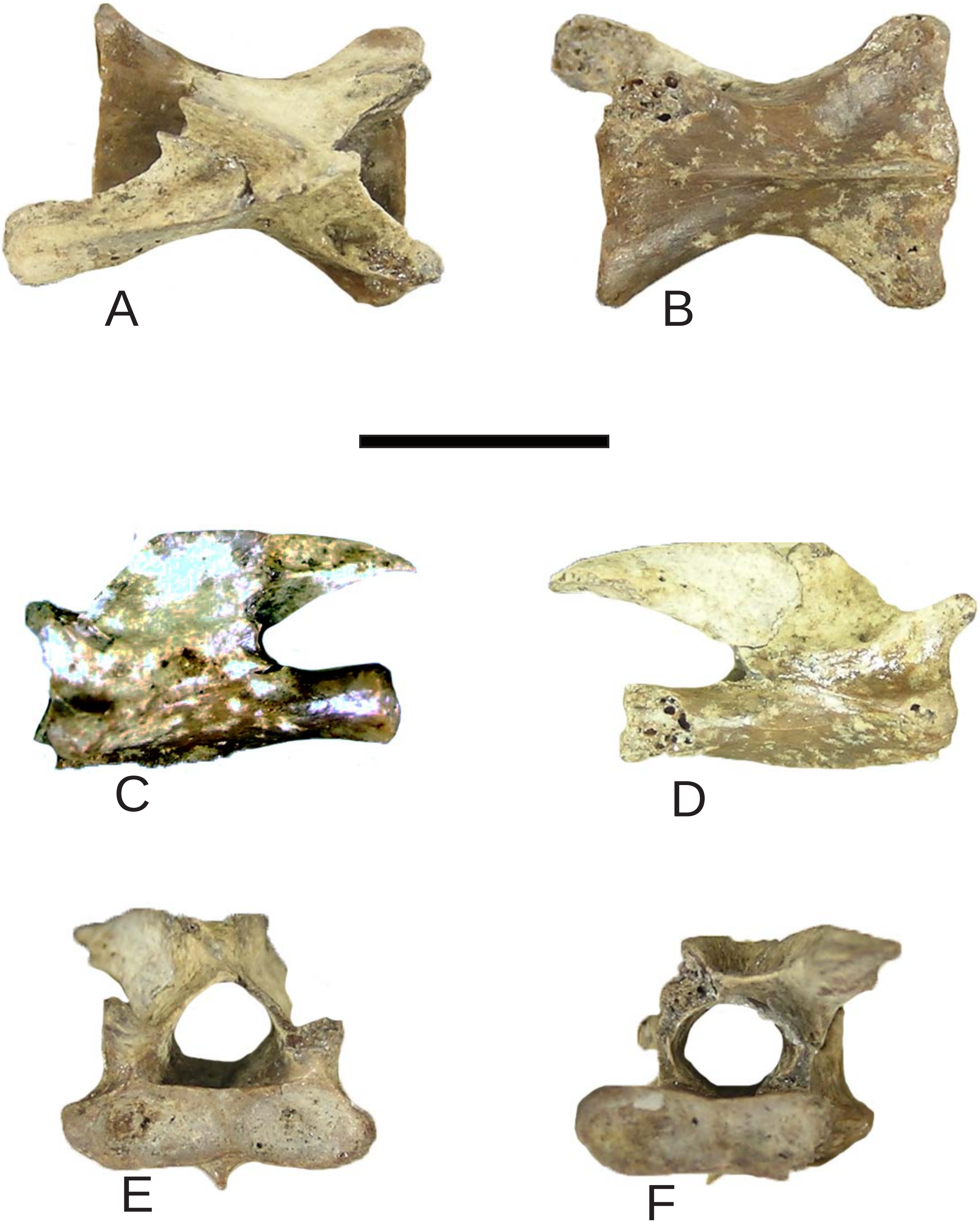

Figure S39. ***Trachemys haugrudi*, holotype nearly complete cervical vertebra VII (ETMNH–8549).**

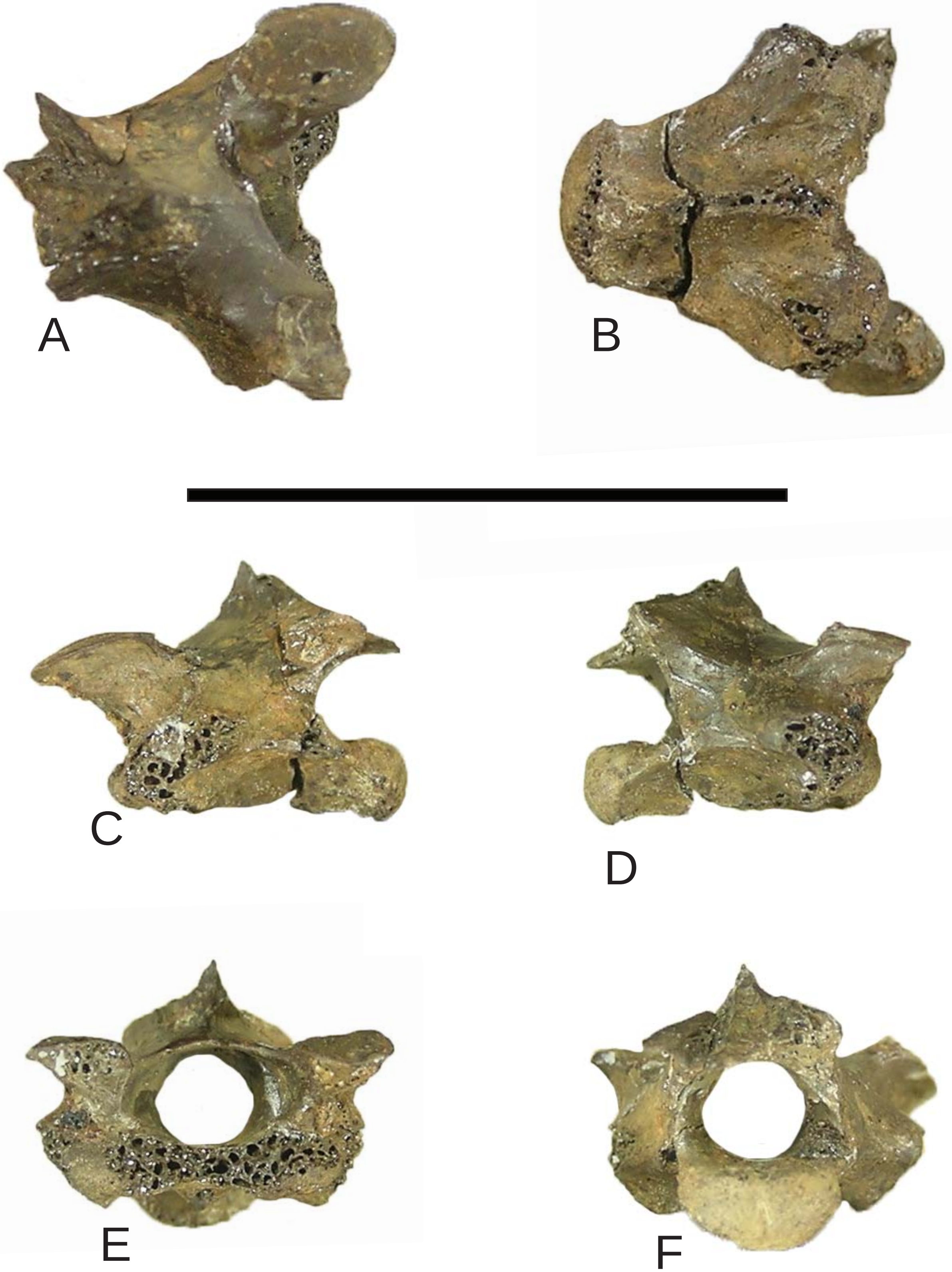

Figure S40. ***Trachemys haugrudi*, paratype nearly complete cervical vertebra VIII (ETMNH–12832).**

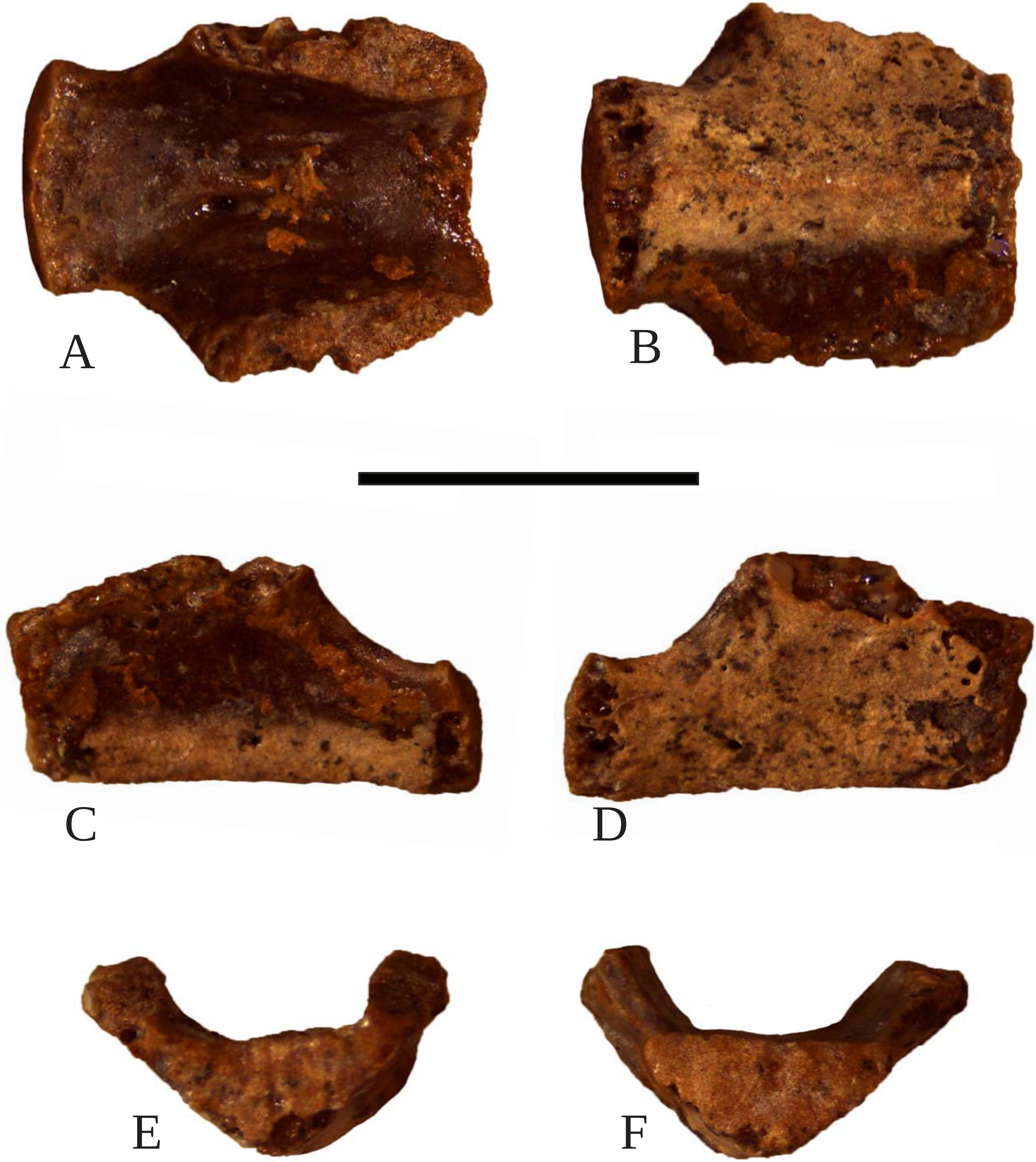

Figure S41. ***Trachemys haugrudi*, holotype anterior dorsal vertebra (ETMNH–8549).**

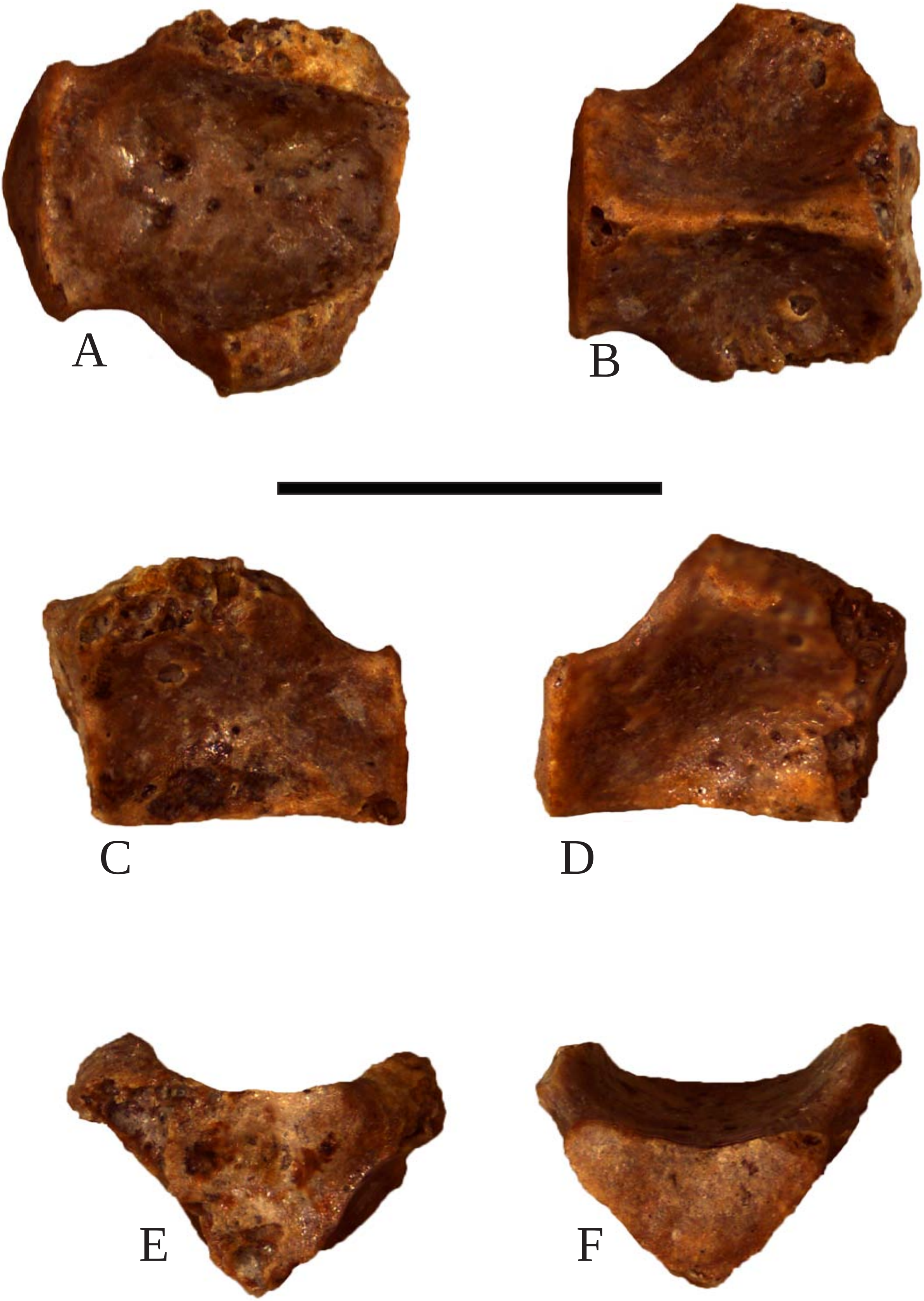

Figure S42. ***Trachemys haugrudi*, holotype posterior dorsal vertebra (ETMNH–8549).**

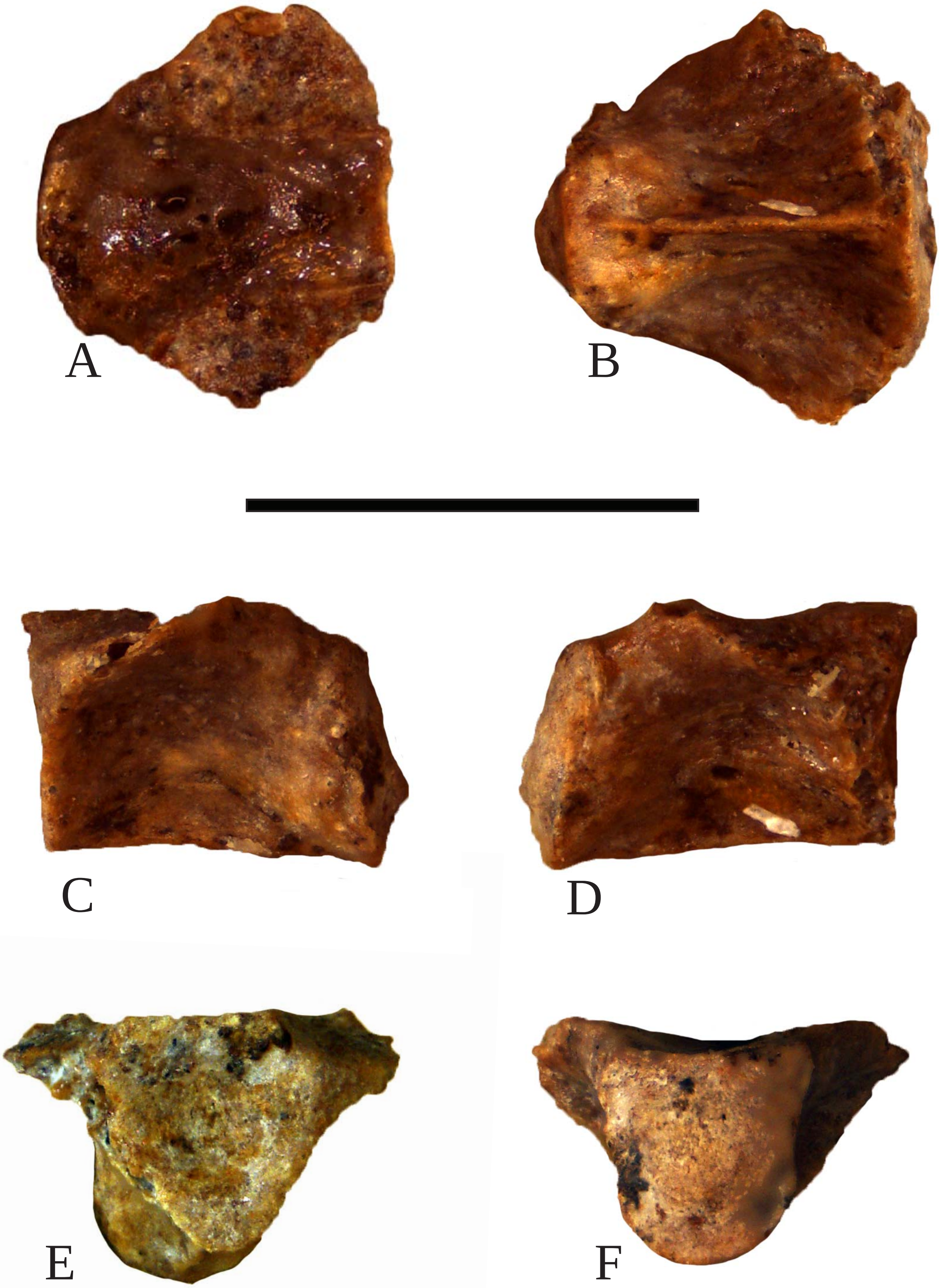

Figure S43. ***Trachemys haugrudi*****, holotype posterior dorsal vertebra (posterior to dorsal vertebra in Figure S42) (ETMNH–8549).**

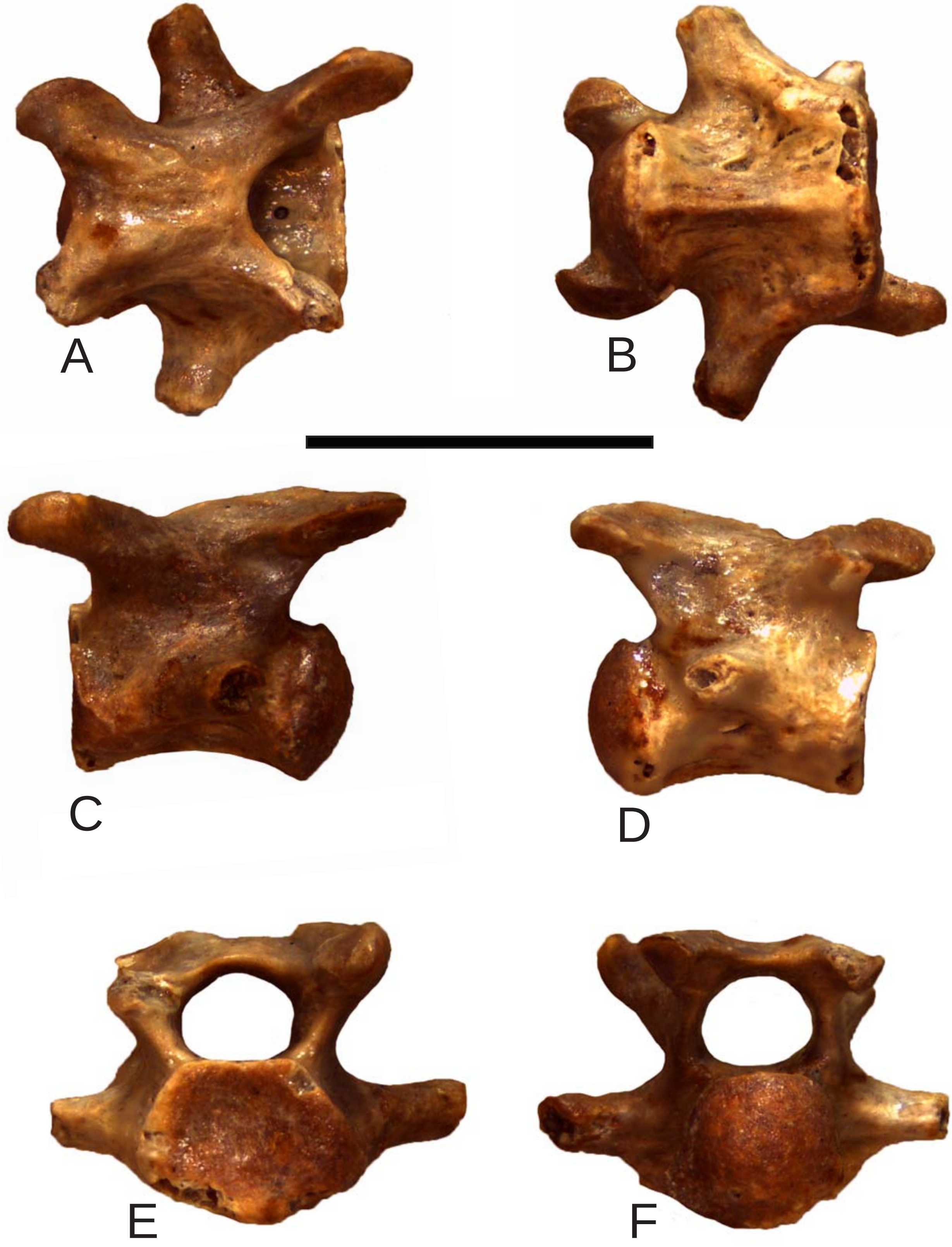

Figure S44. ***Trachemys haugrudi*****, holotype proximal caudal vertebra (ETMNH–8549).**

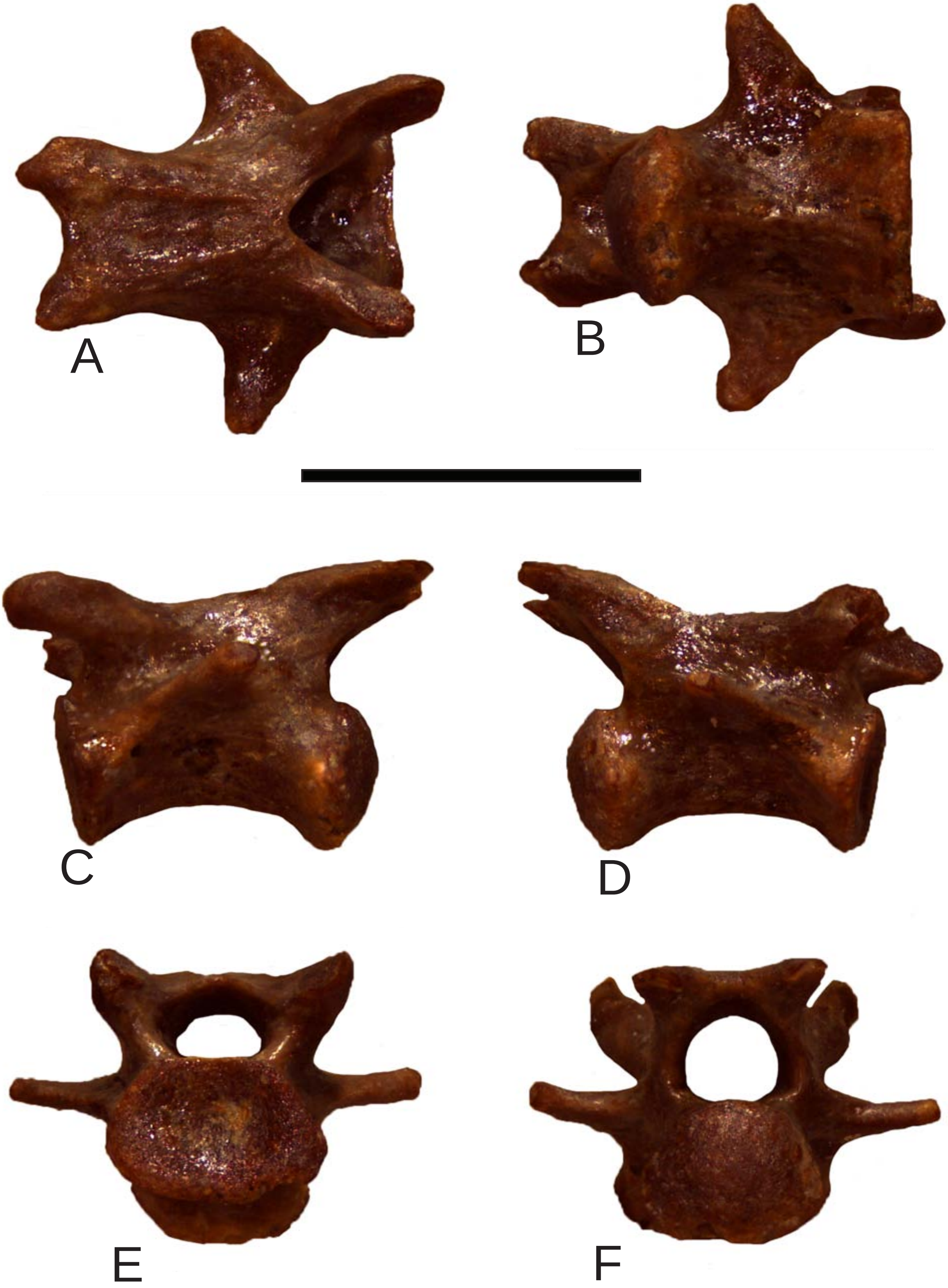

Figure S45. ***Trachemys haugrudi*, holotype medioproximal caudal vertebra (posterior to caudal vertebra in Figure S44) (ETMNH–8549).**

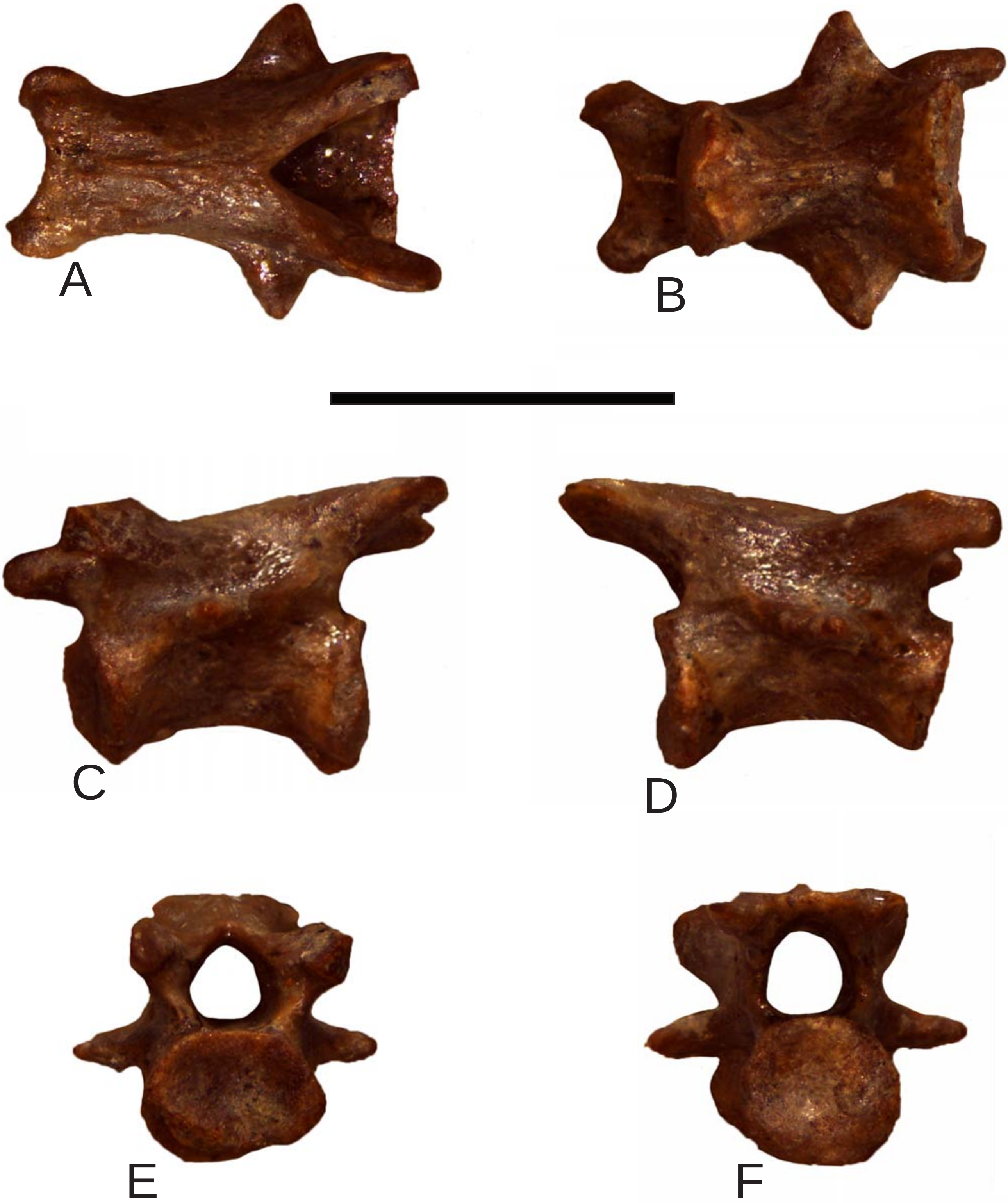

Figure S46. ***Trachemys haugrudi*, holotype median caudal vertebra (ETMNH–8549).**

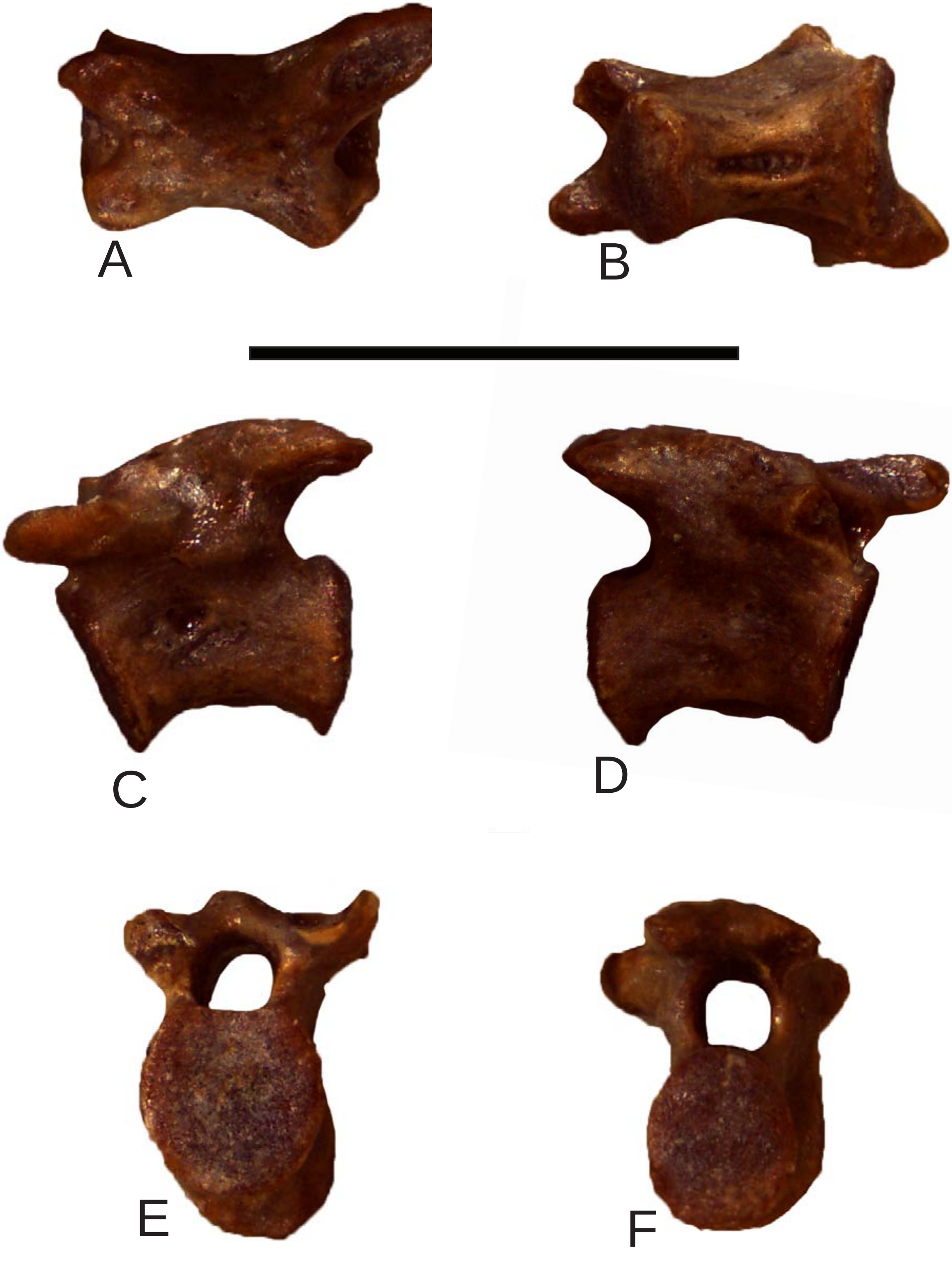

Figure S47. ***Trachemys haugrudi*****, holotype mediodistal caudal vertebra (ETMNH–8549).**

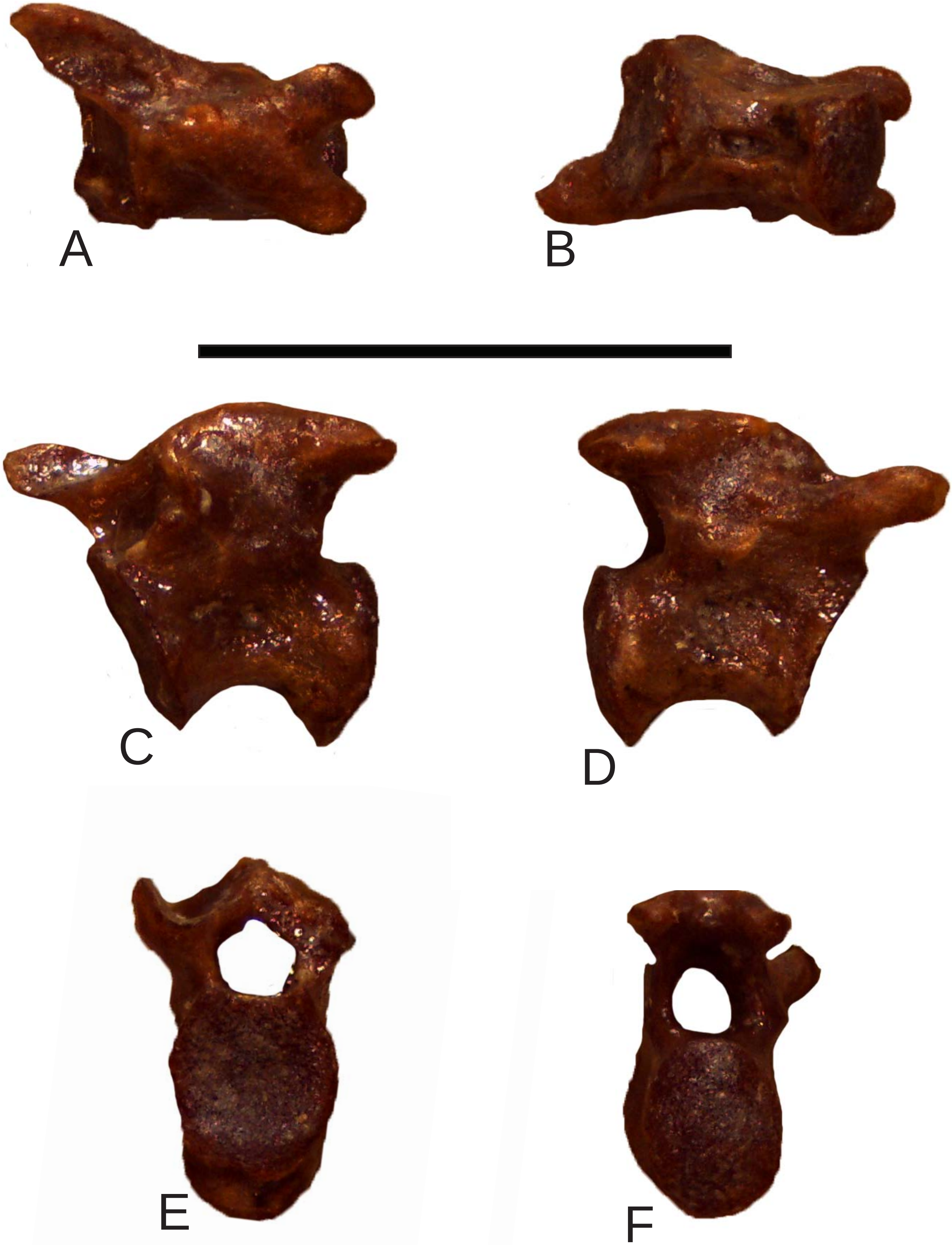

Figure S48. ***Trachemys haugrudi*****, holotype distal caudal vertebra (posterior to caudal vertebra in Figure S47) (ETMNH–8549).**

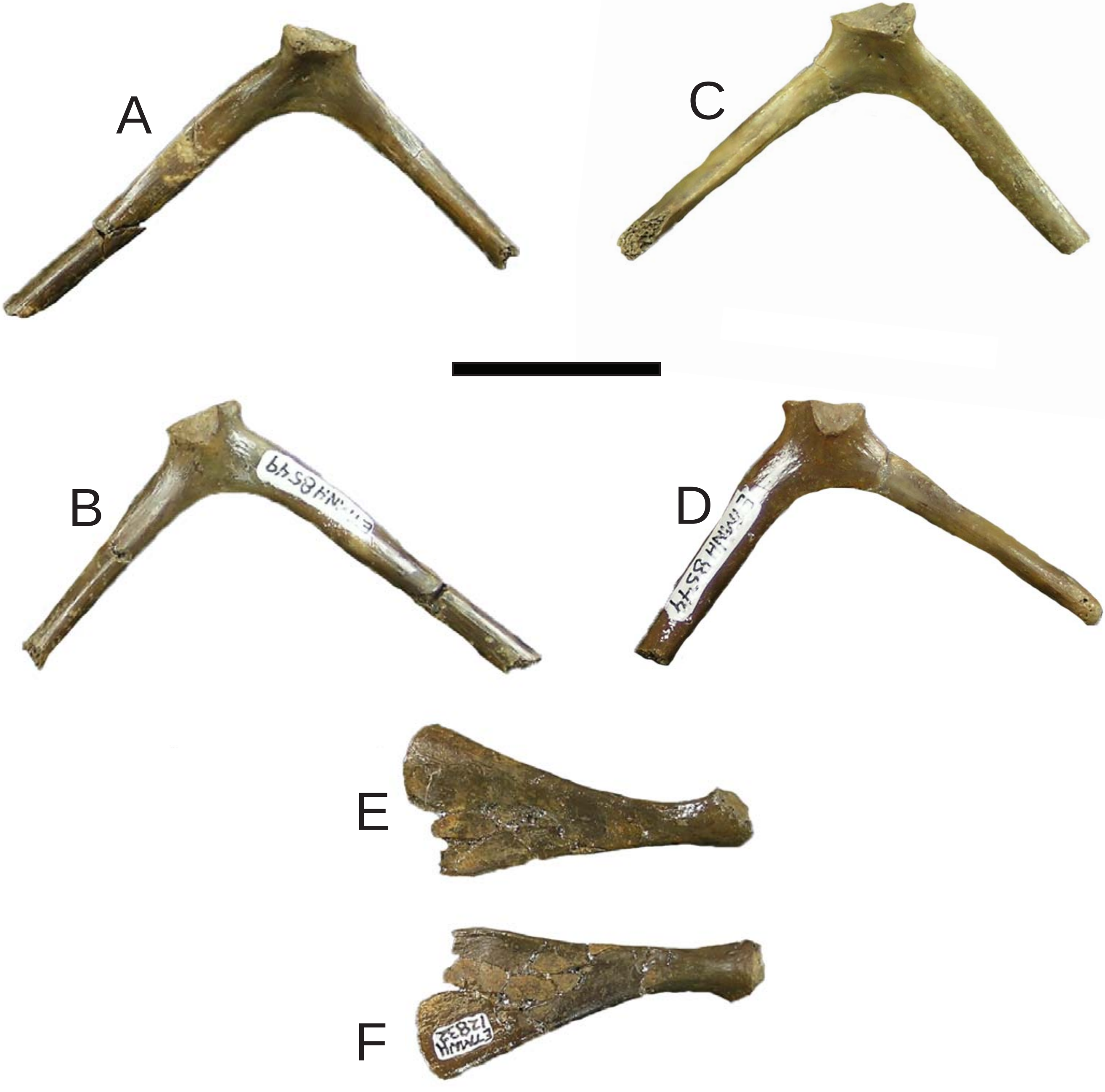

Figure S49. ***Trachemys haugrudi*****, scapula and coracoid.**

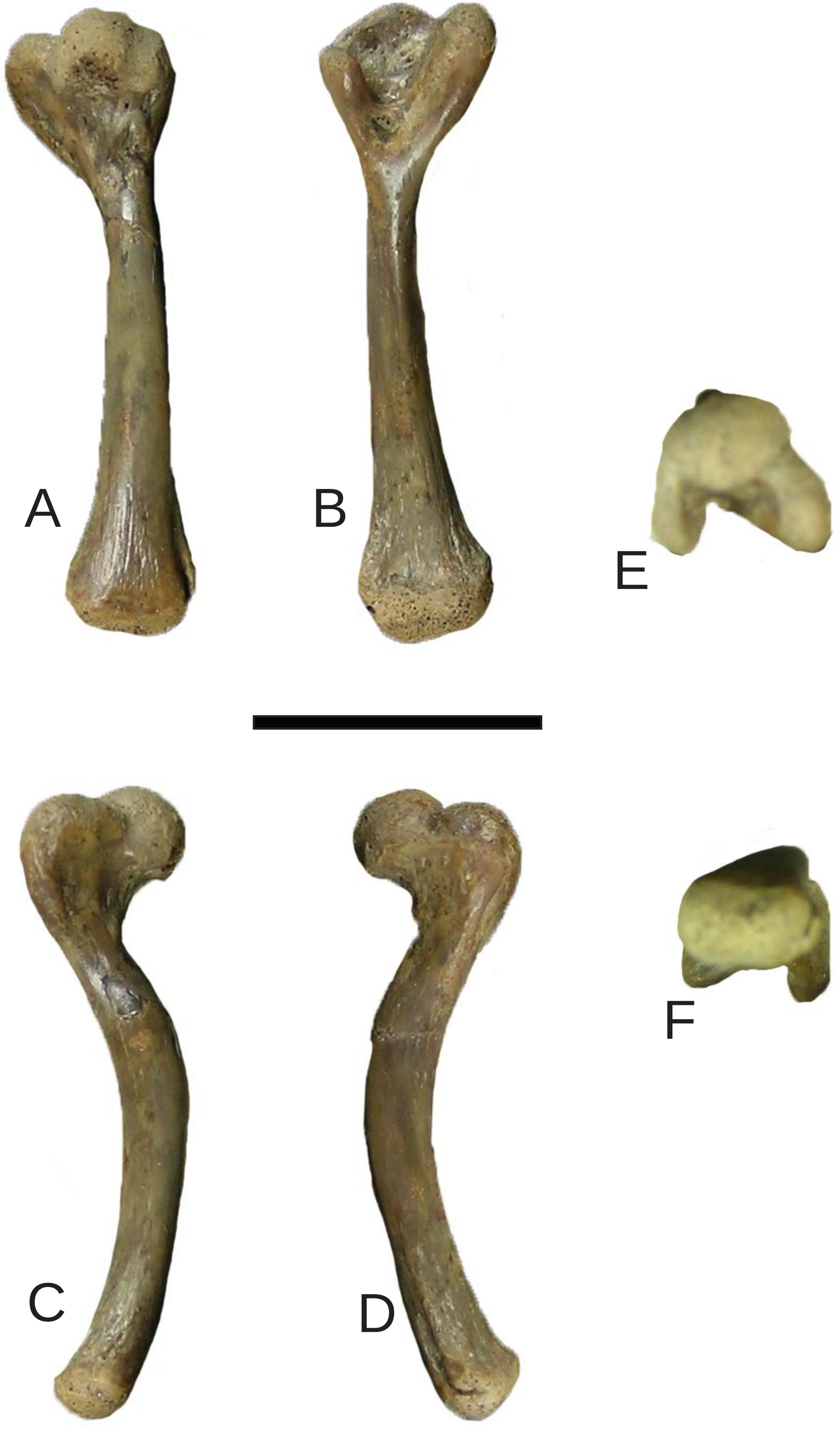

Figure S50. ***Trachemys haugrudi*, holotype complete right humerus (ETMNH–8549).**

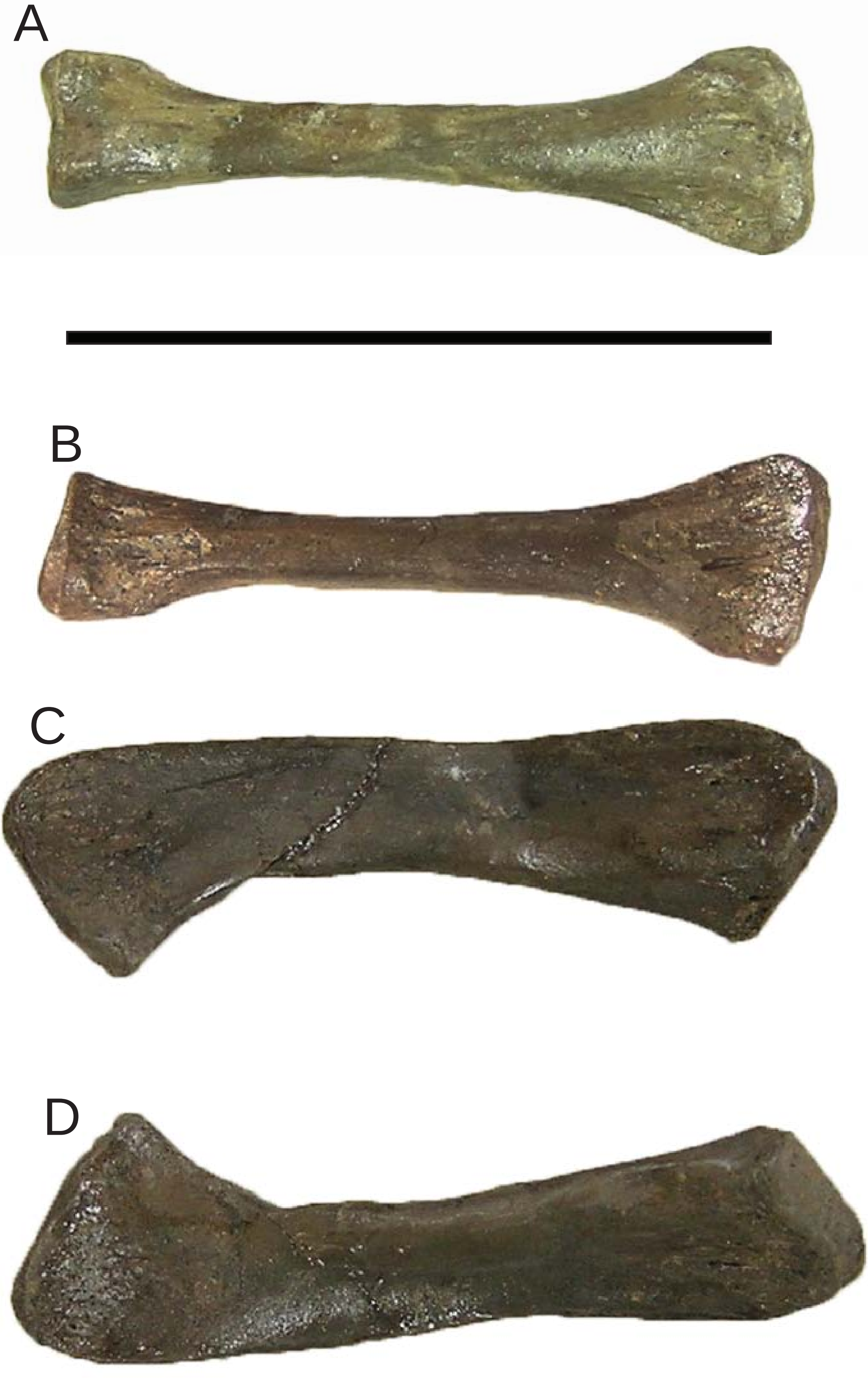

Figure S51. ***Trachemys haugrudi*****, radius and ulna.**

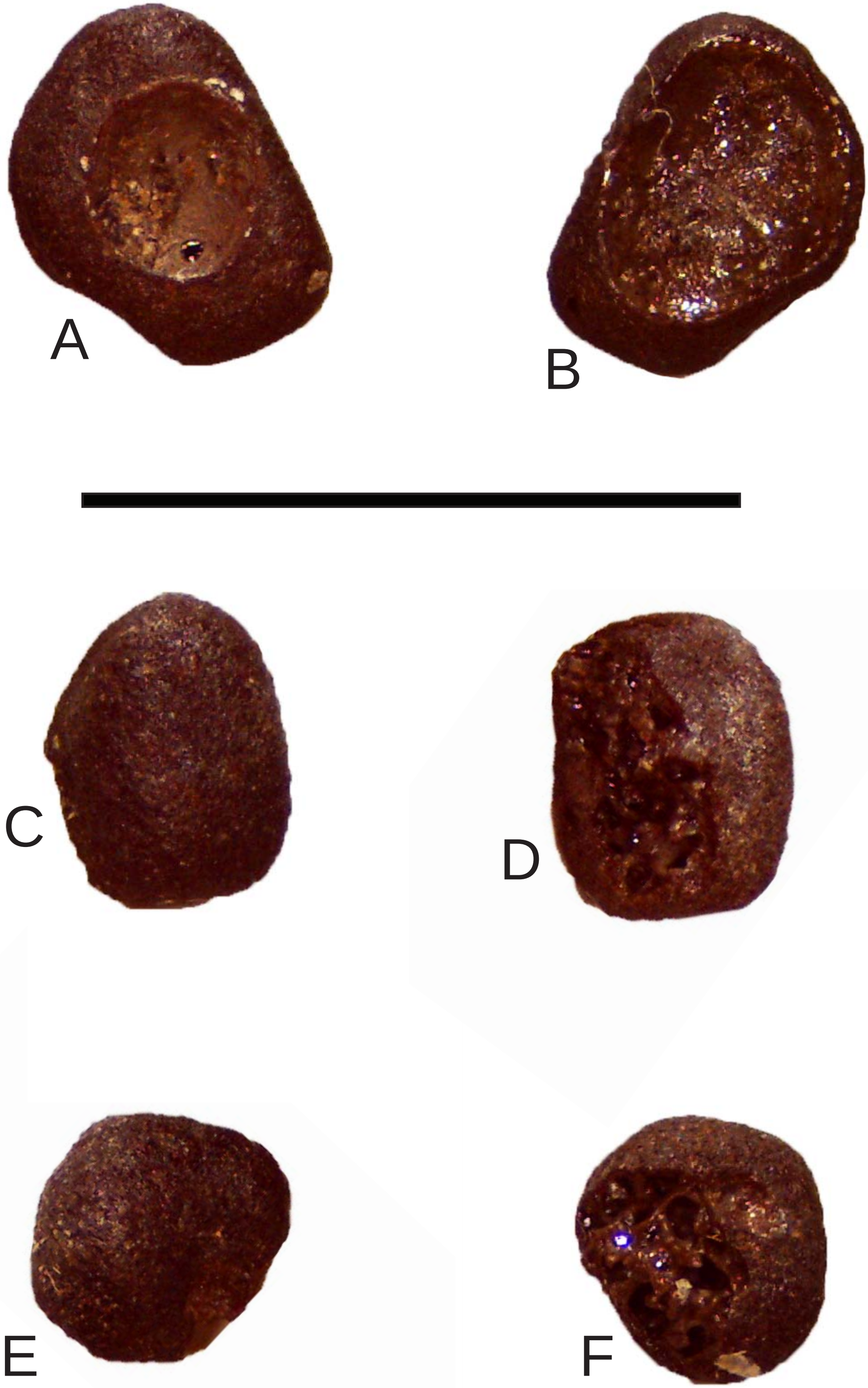

Figure S52. ***Trachemys haugrudi*****, carpals.**

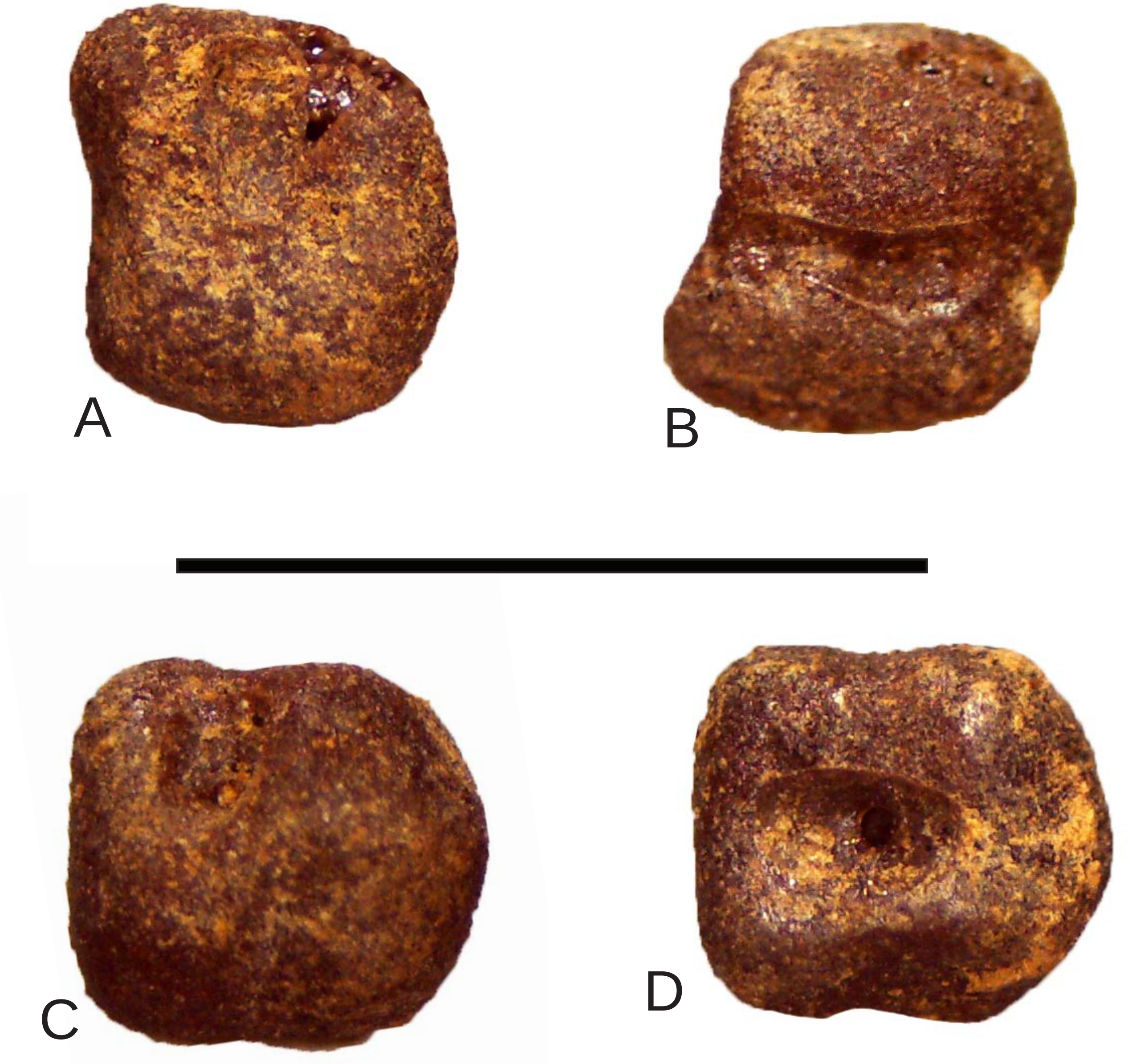

Figure S53. ***Trachemys haugrudi*****, distal carpals.**

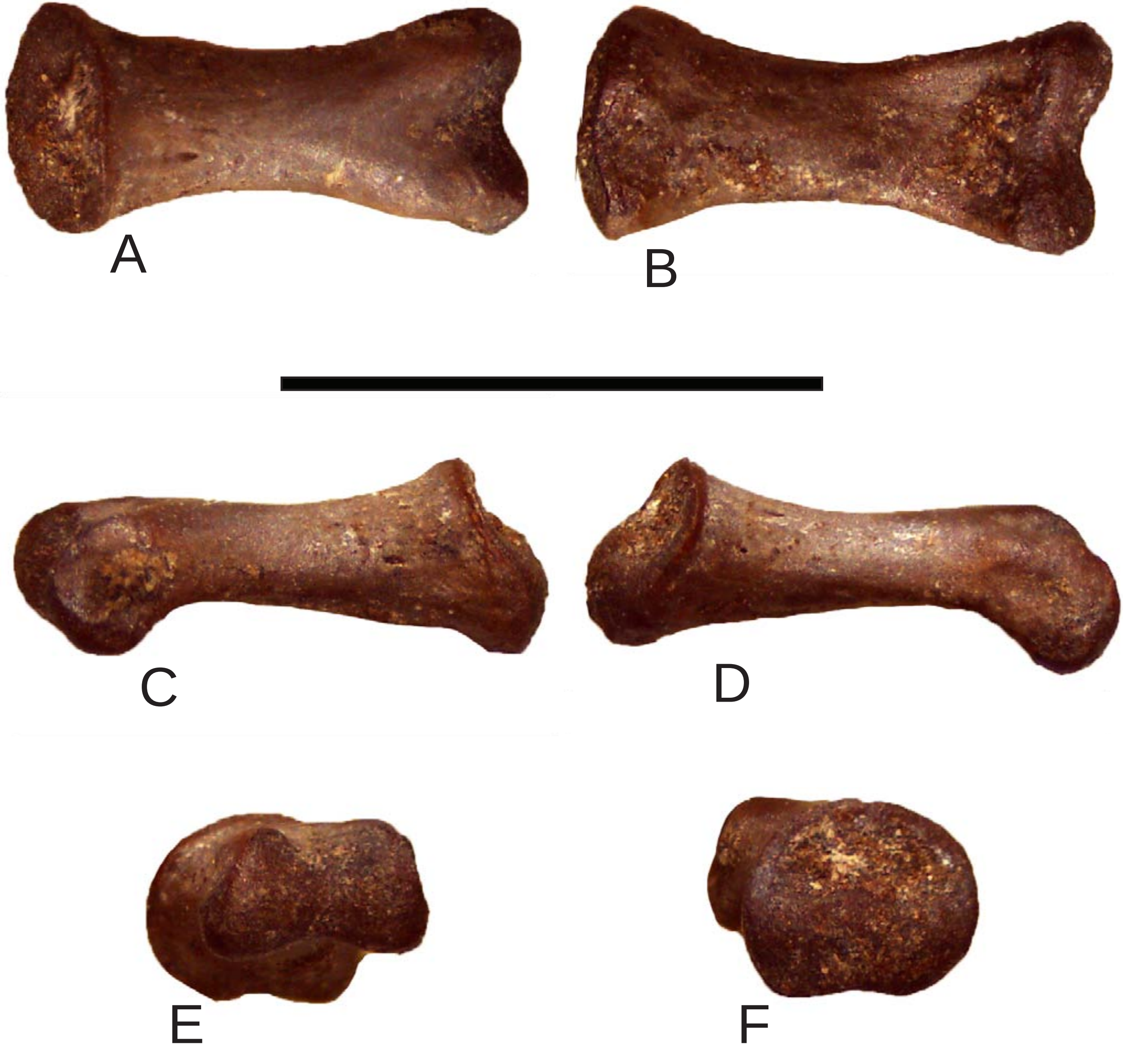

Figure S54. ***Trachemys haugrudi*, paratype right proximal phalanx ?IV (ETMNH–3558).**

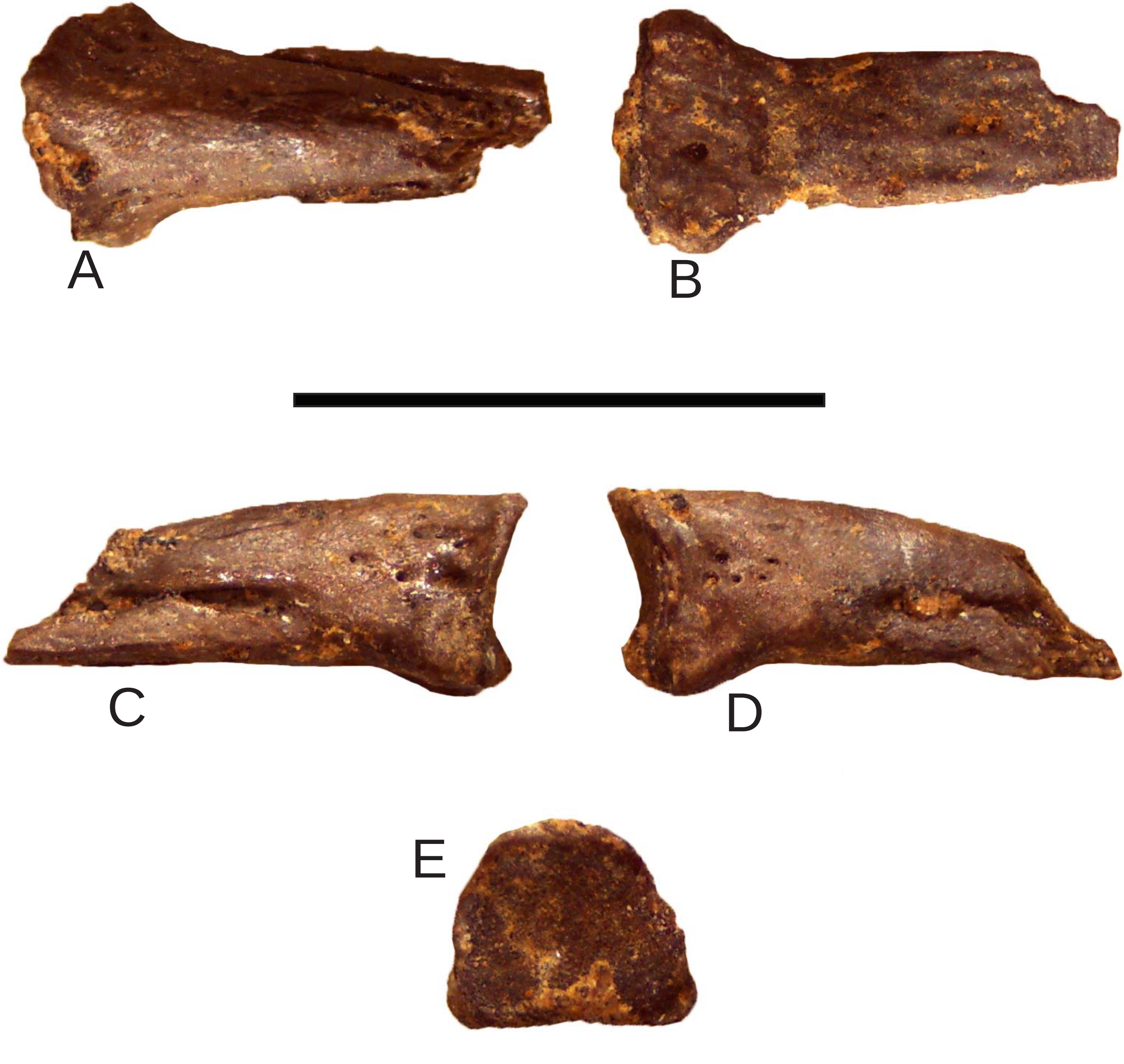

Figure S55. ***Trachemys haugrudi*, paratype right manual ungual ?I (ETMNH–3562).**

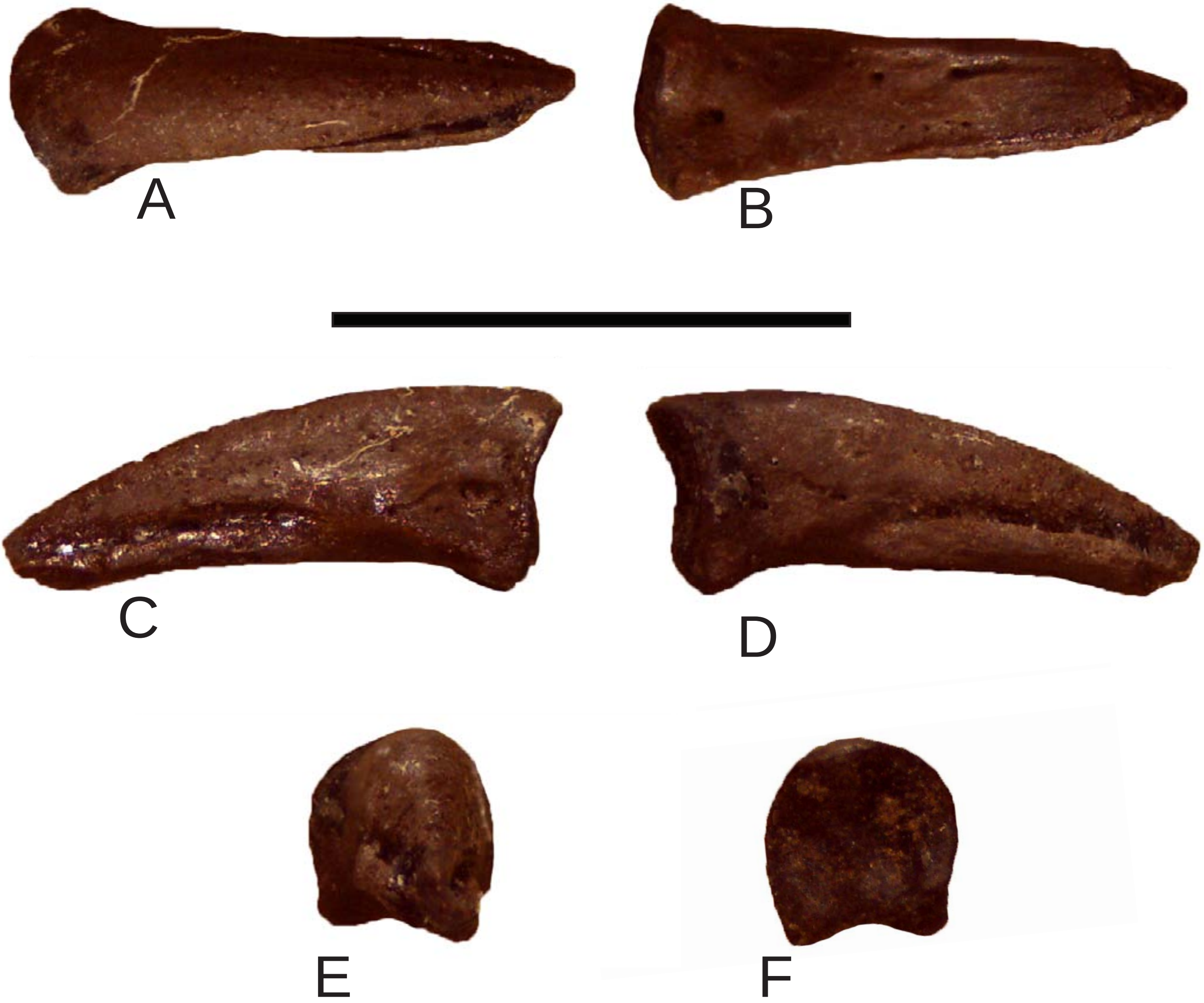

Figure S56. ***Trachemys haugrudi*, paratype right manual ungual ?II (ETMNH–3558).**

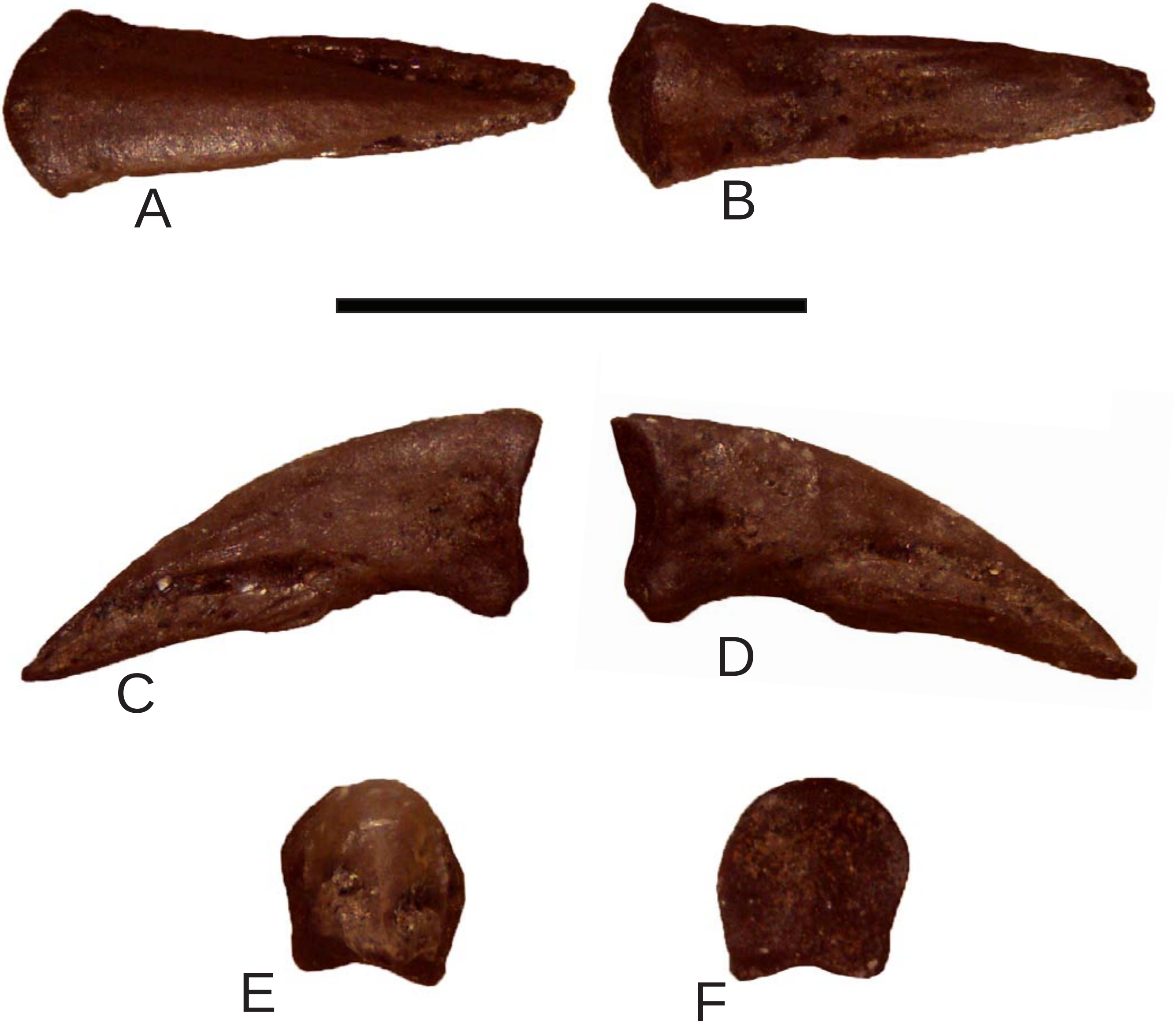

Figure S57. ***Trachemys haugrudi*****, paratype right manual ungual ?III (ETMNH–3558).**

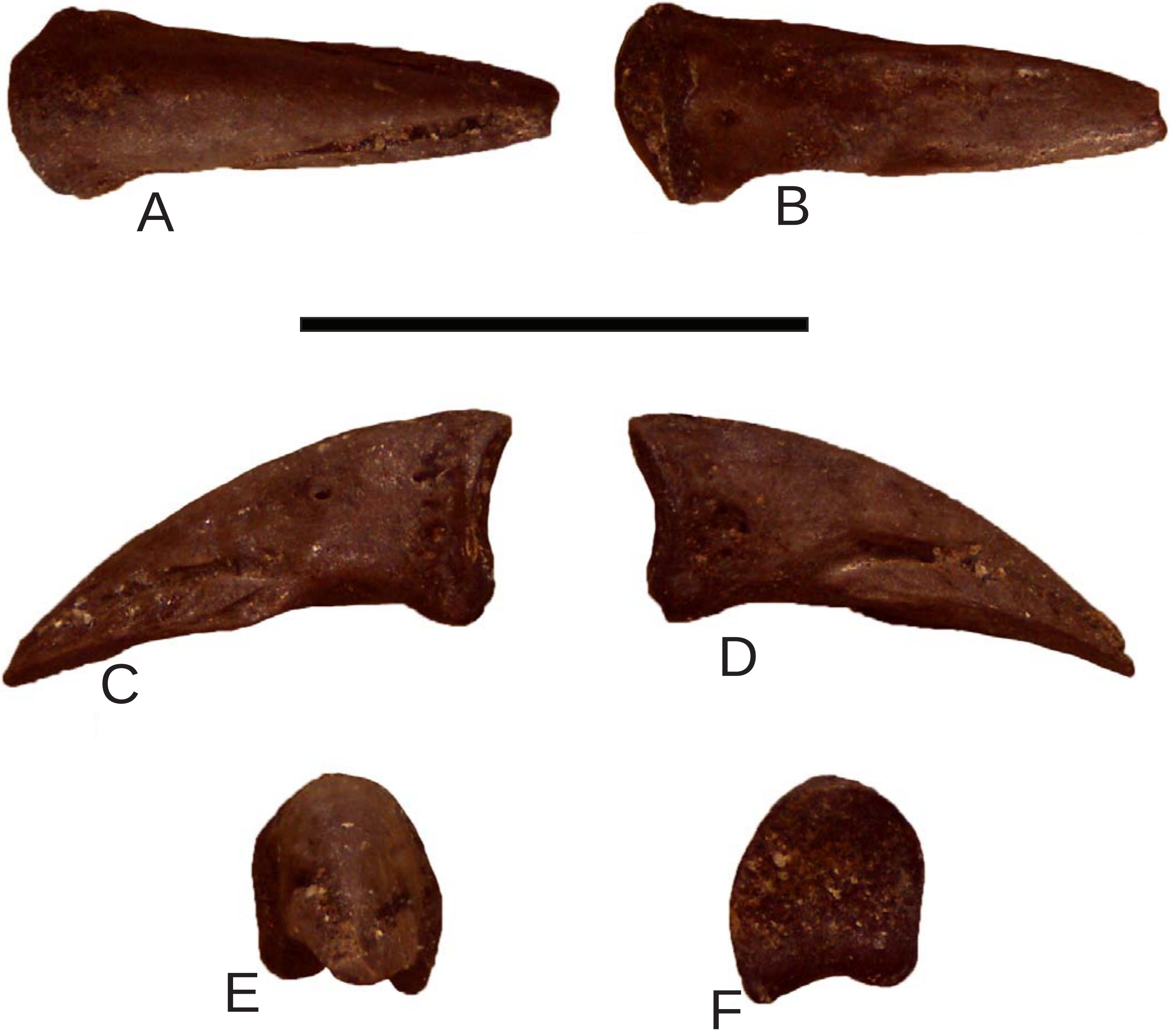

Figure S58. ***Trachemys haugrudi*, paratype right manual ungual ?IV (ETMNH–3558).**

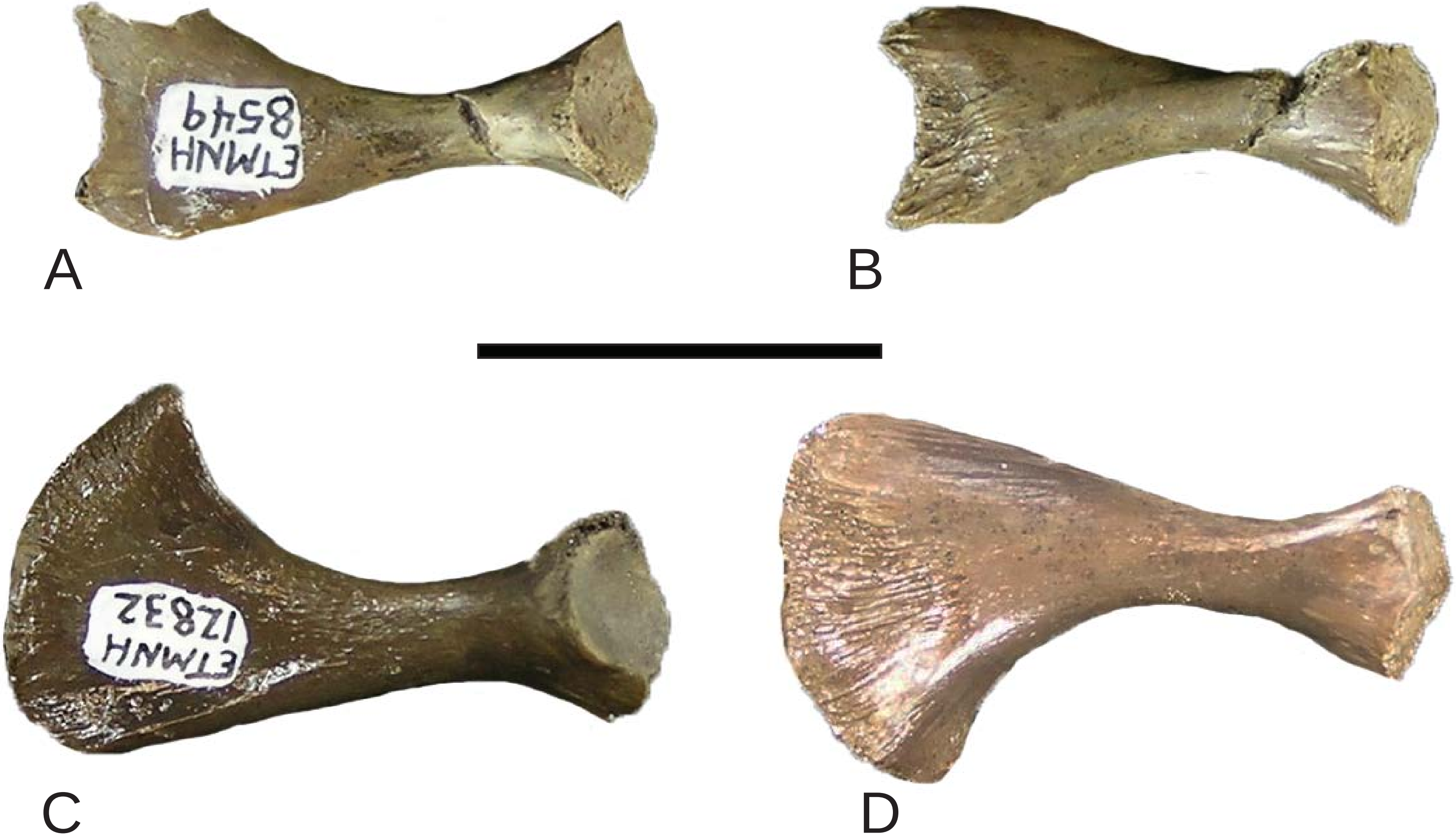

Figure S59. ***Trachemys haugrudi*****, holotype nearly complete left ilium (ETMNH–8549).**

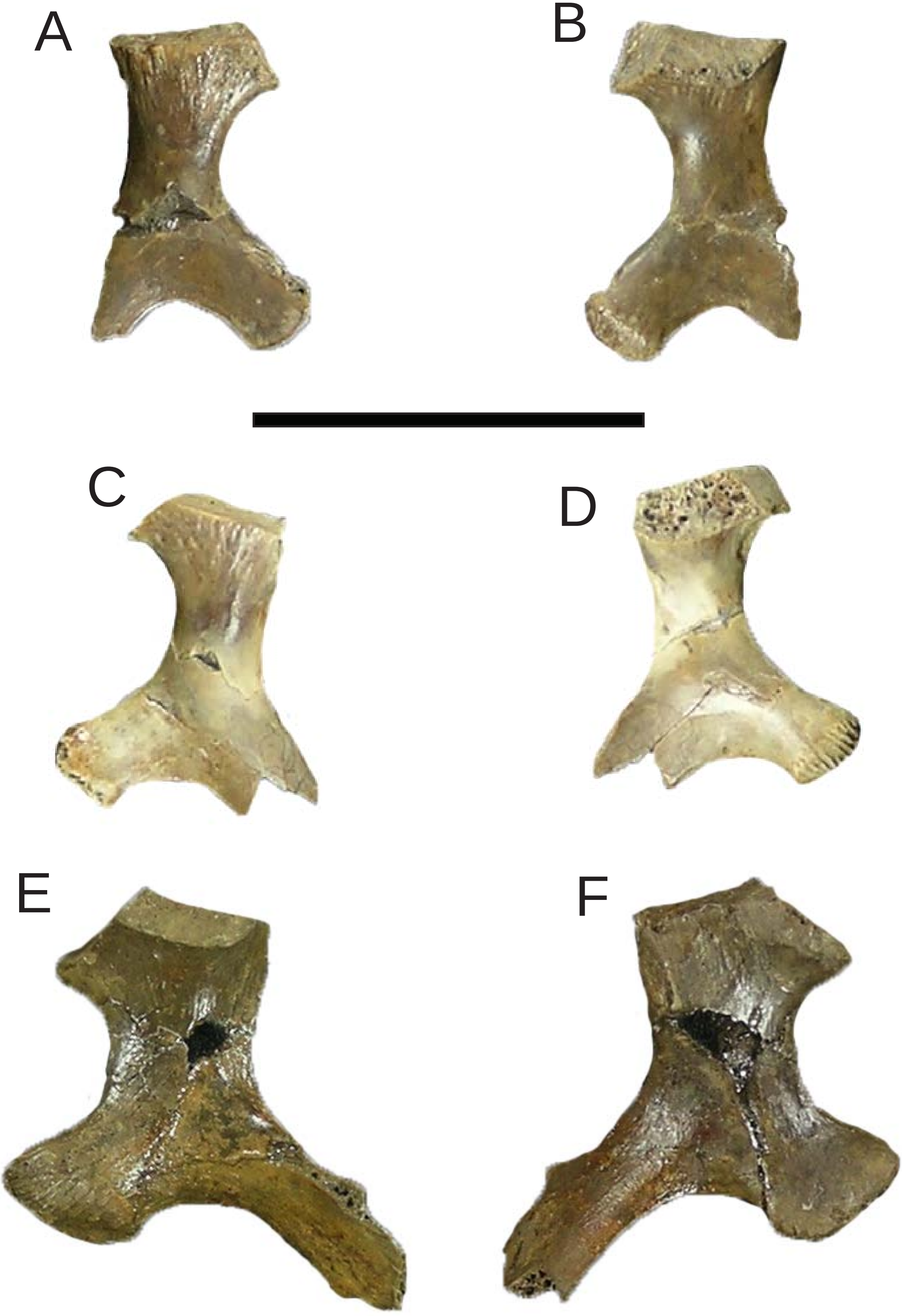

Figure S60. ***Trachemys haugrudi*, ischium.**

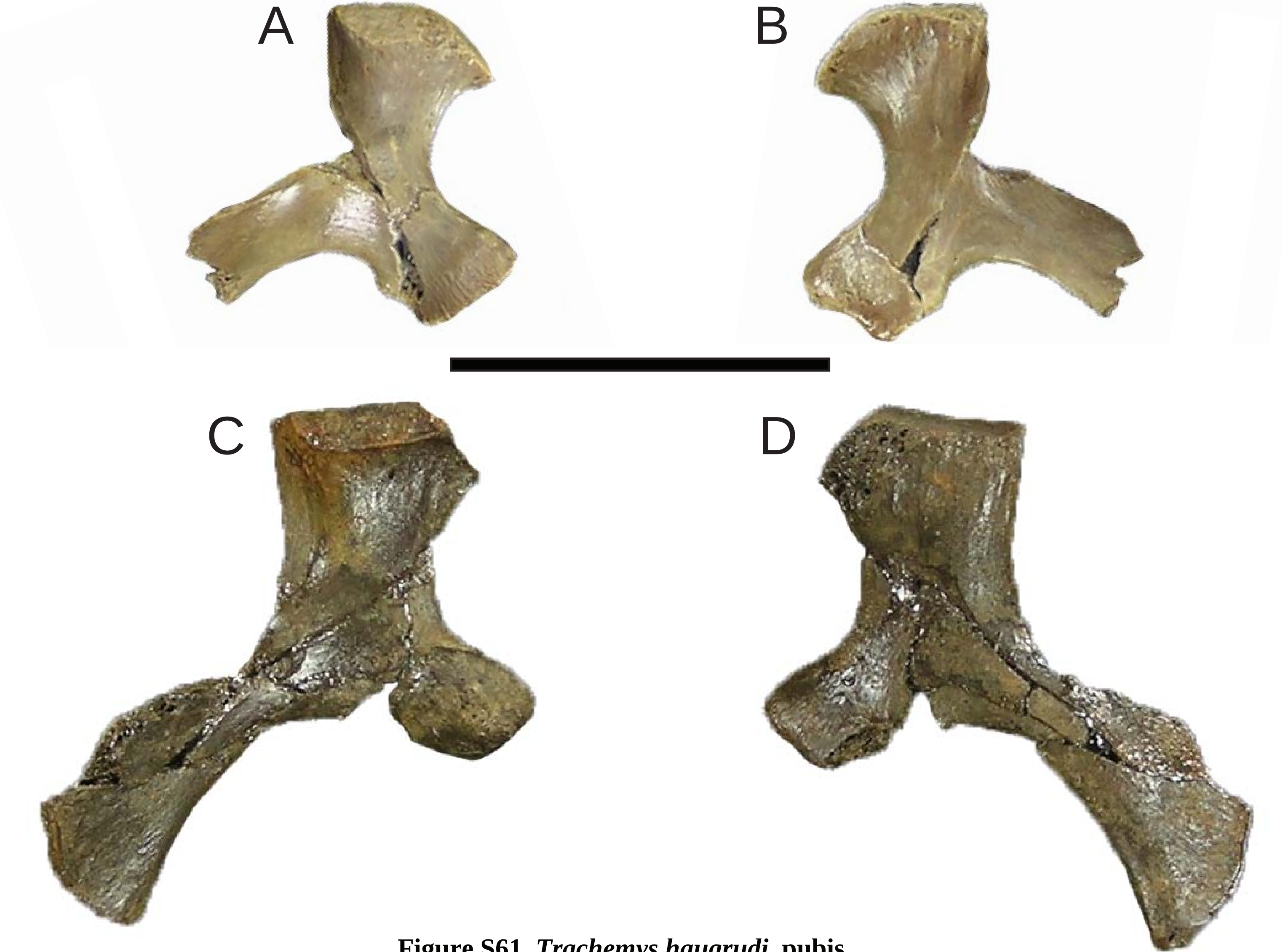

Figure S61. ***Trachemys haugrudi*, pubis.**

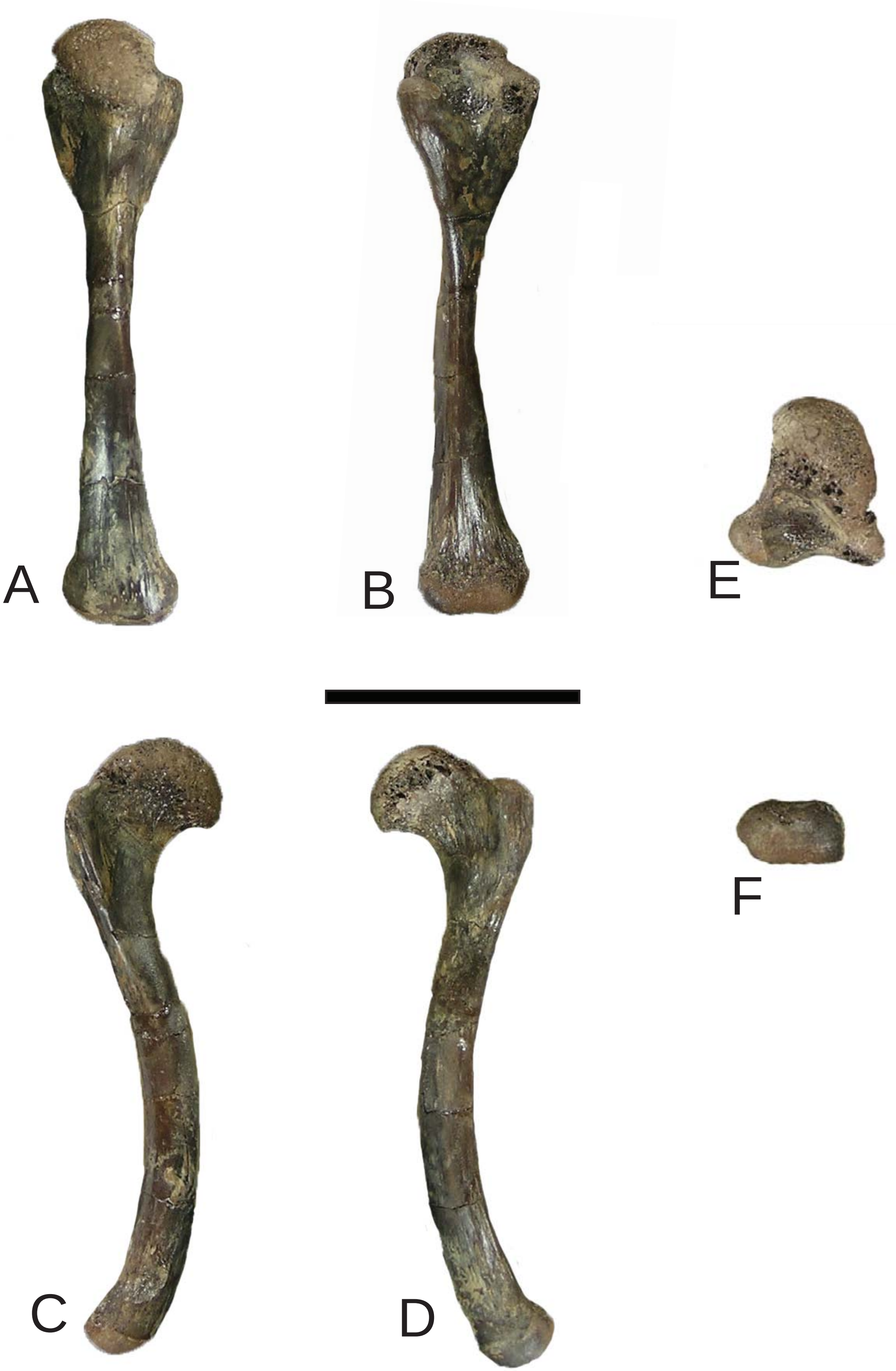

Figure S62. ***Trachemys haugrudi*****, referred complete right femur (ETMNH–12265).**

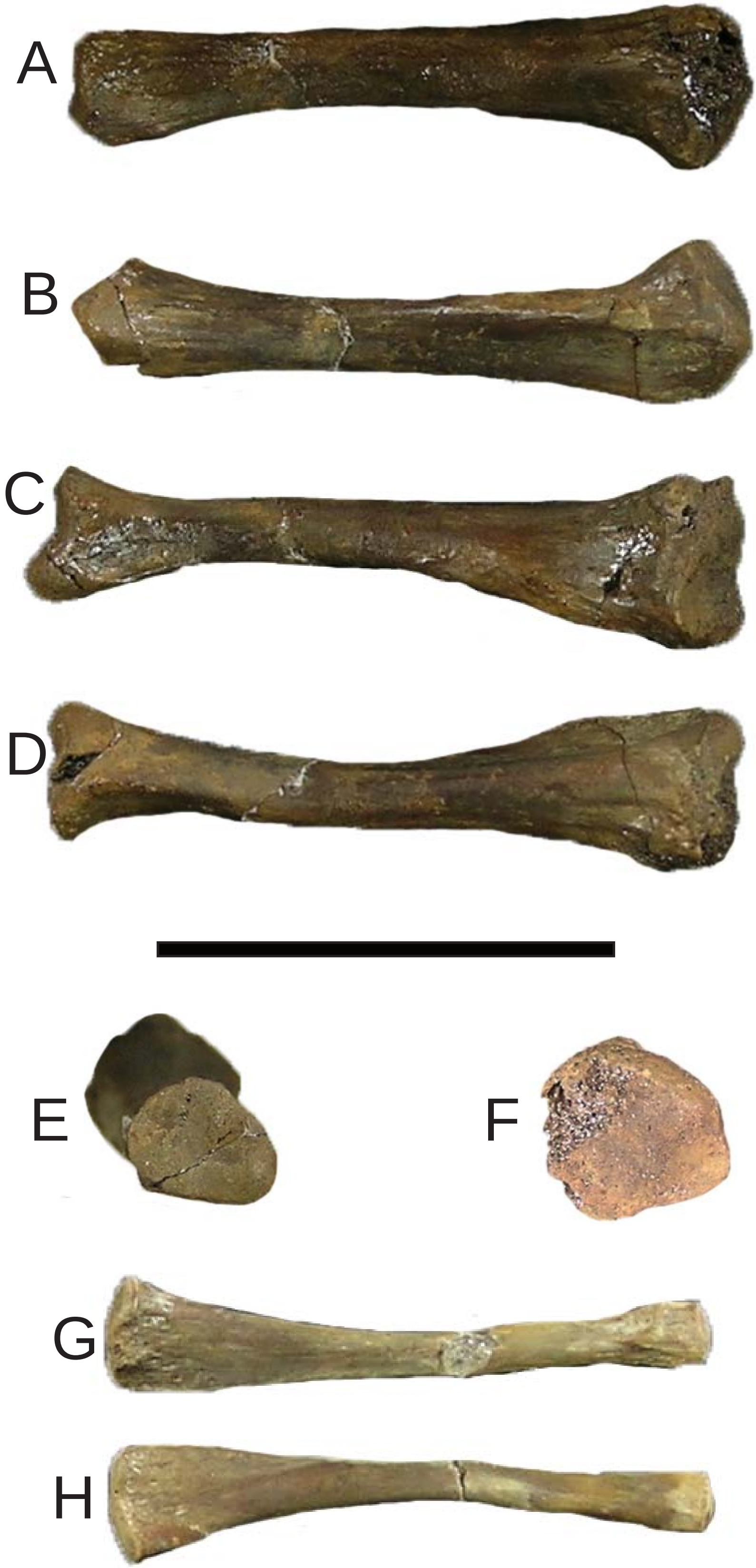

Figure S63. ***Trachemys haugrudi*, tibia and fibula.**

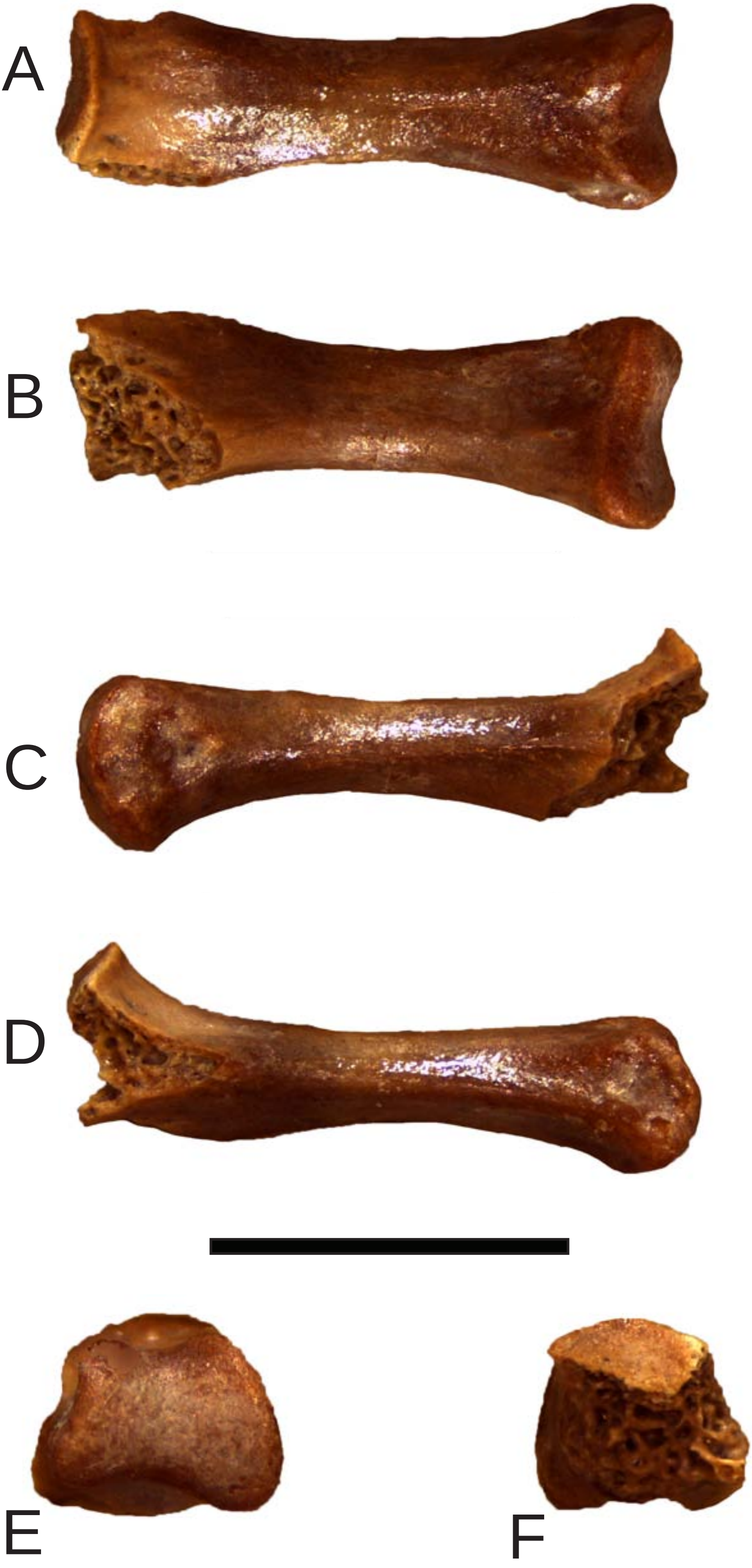

Figure S64. ***Trachemys haugrudi*, holotype right metatarsal ?II (ETMNH–8549).**

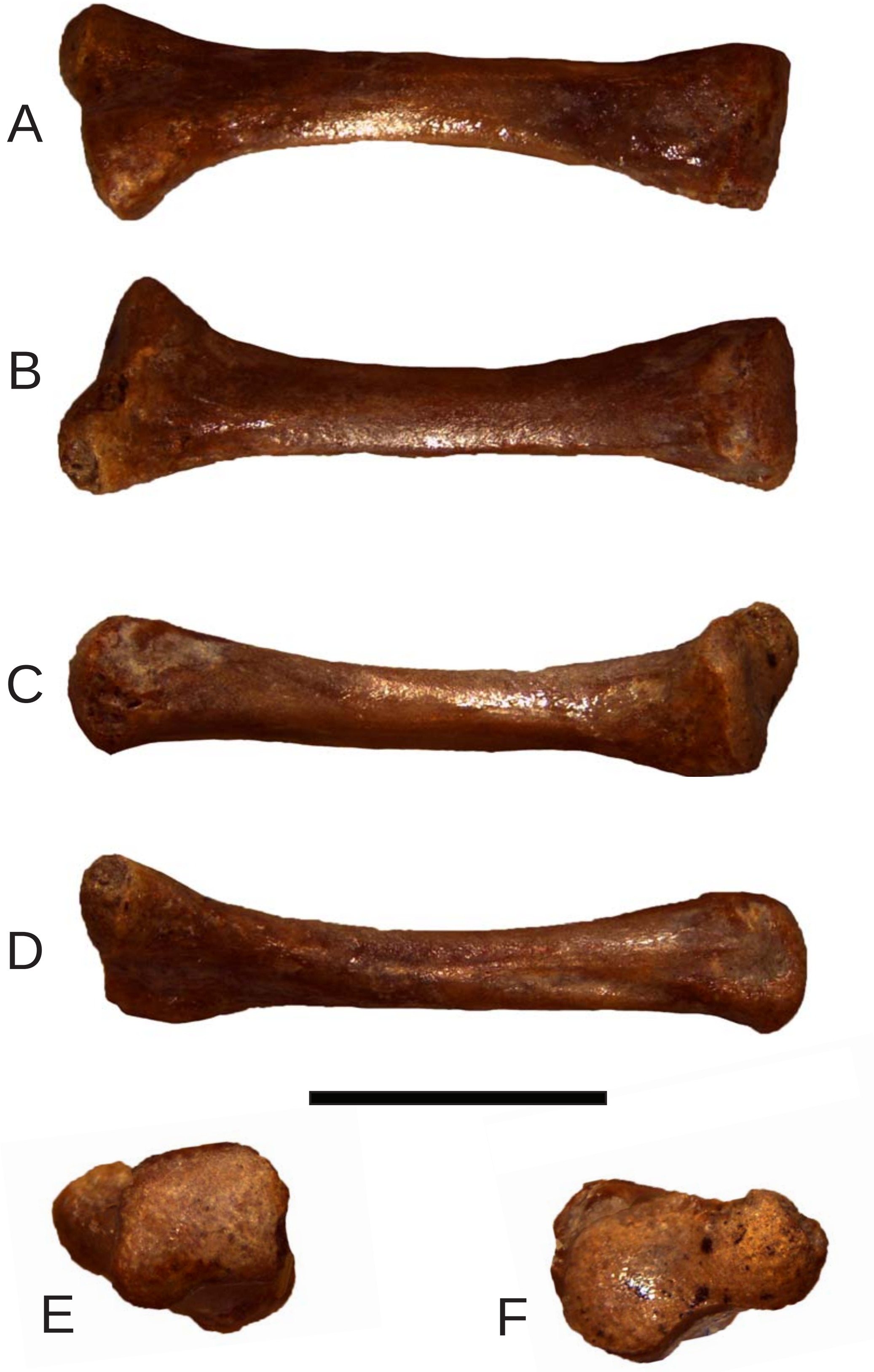

Figure S65. ***Trachemys haugrudi*, holotype right metatarsal III (ETMNH–8549).**

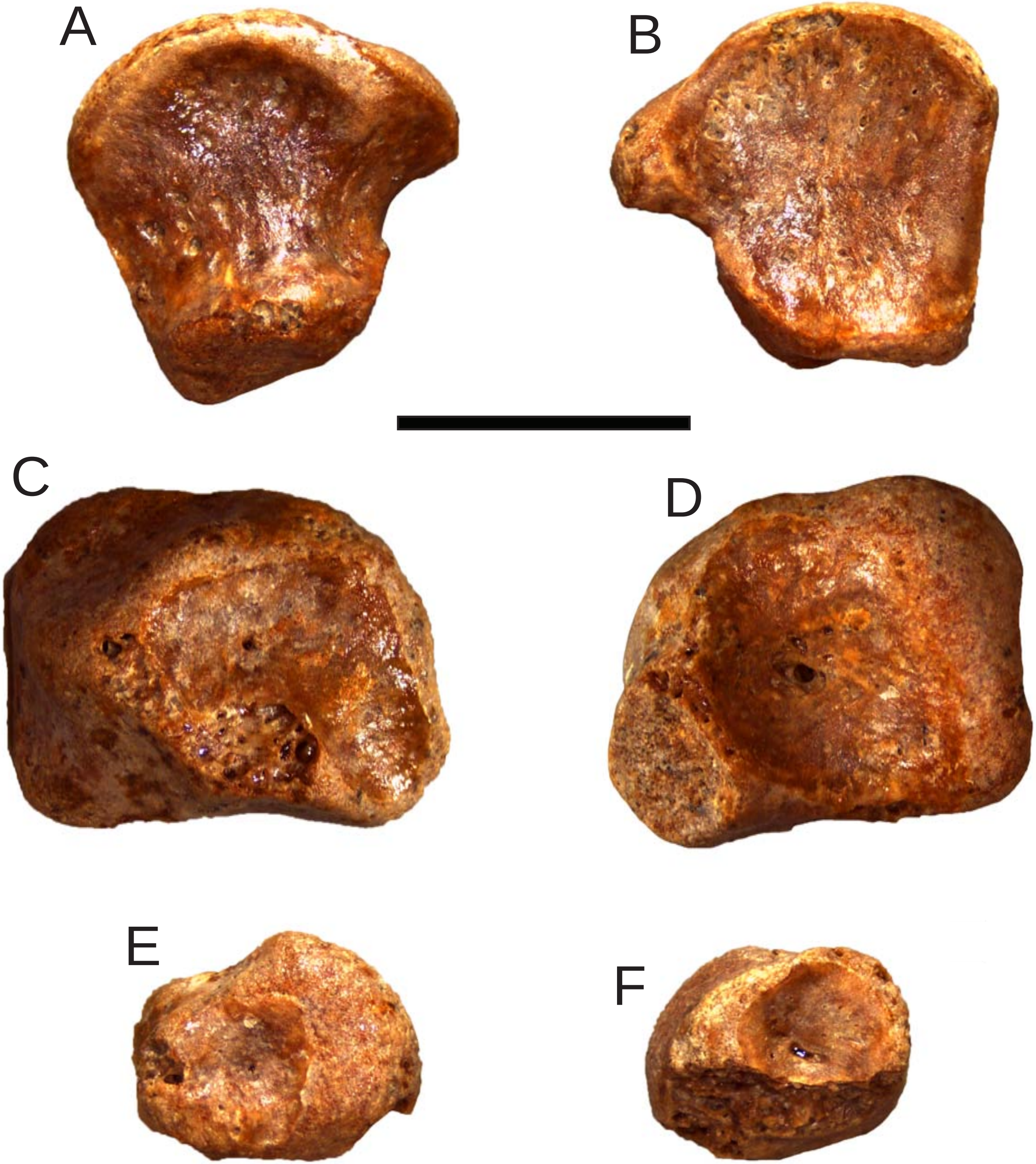

Figure S66. ***Trachemys haugrudi*, right metatarsal V, right astragalus, and incomplete right distal tarsal ?IV (ETMNH–8549).**

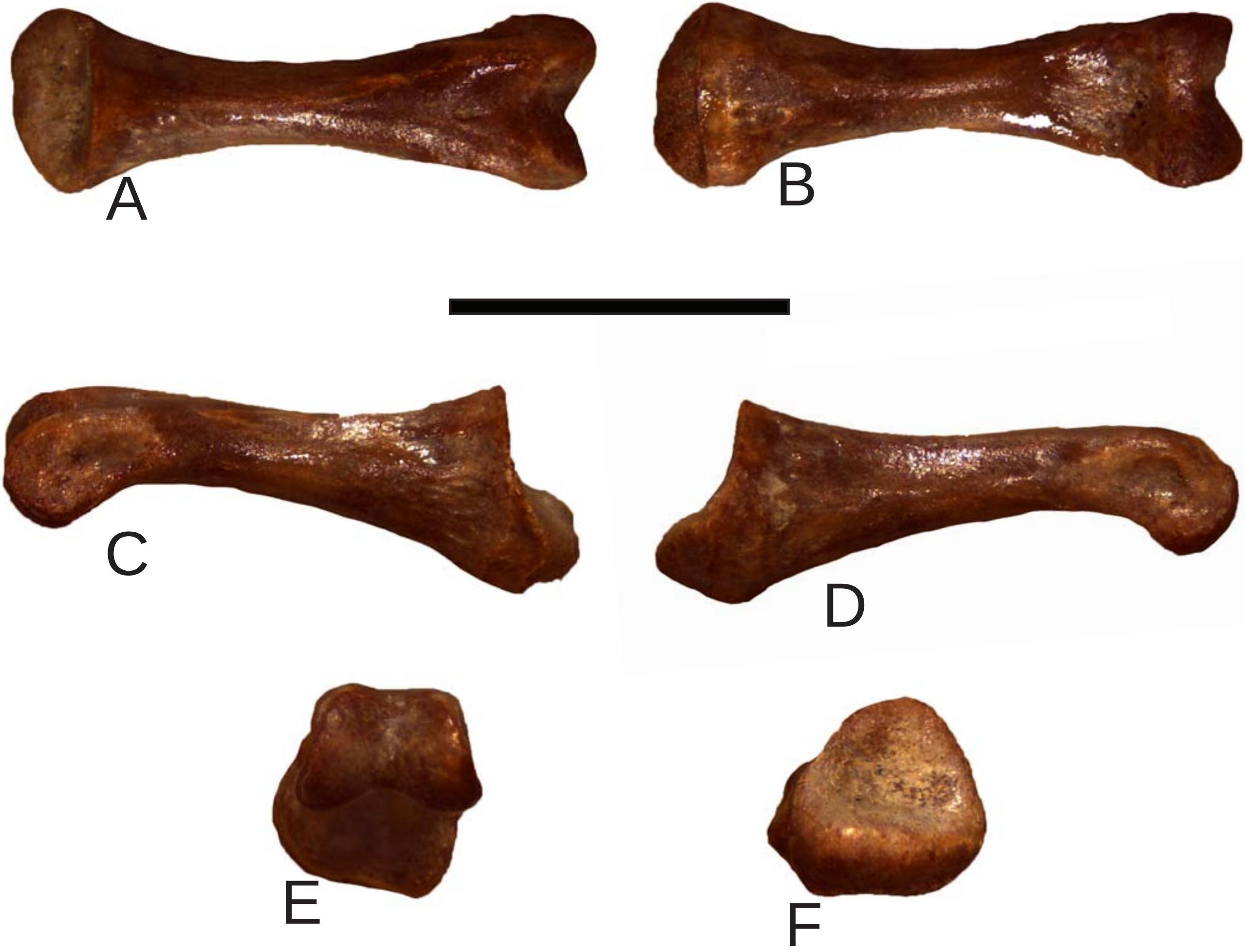

Figure S67. ***Trachemys haugrudi*****, holotype right proximal phalanx ?III (ETMNH–8549).**

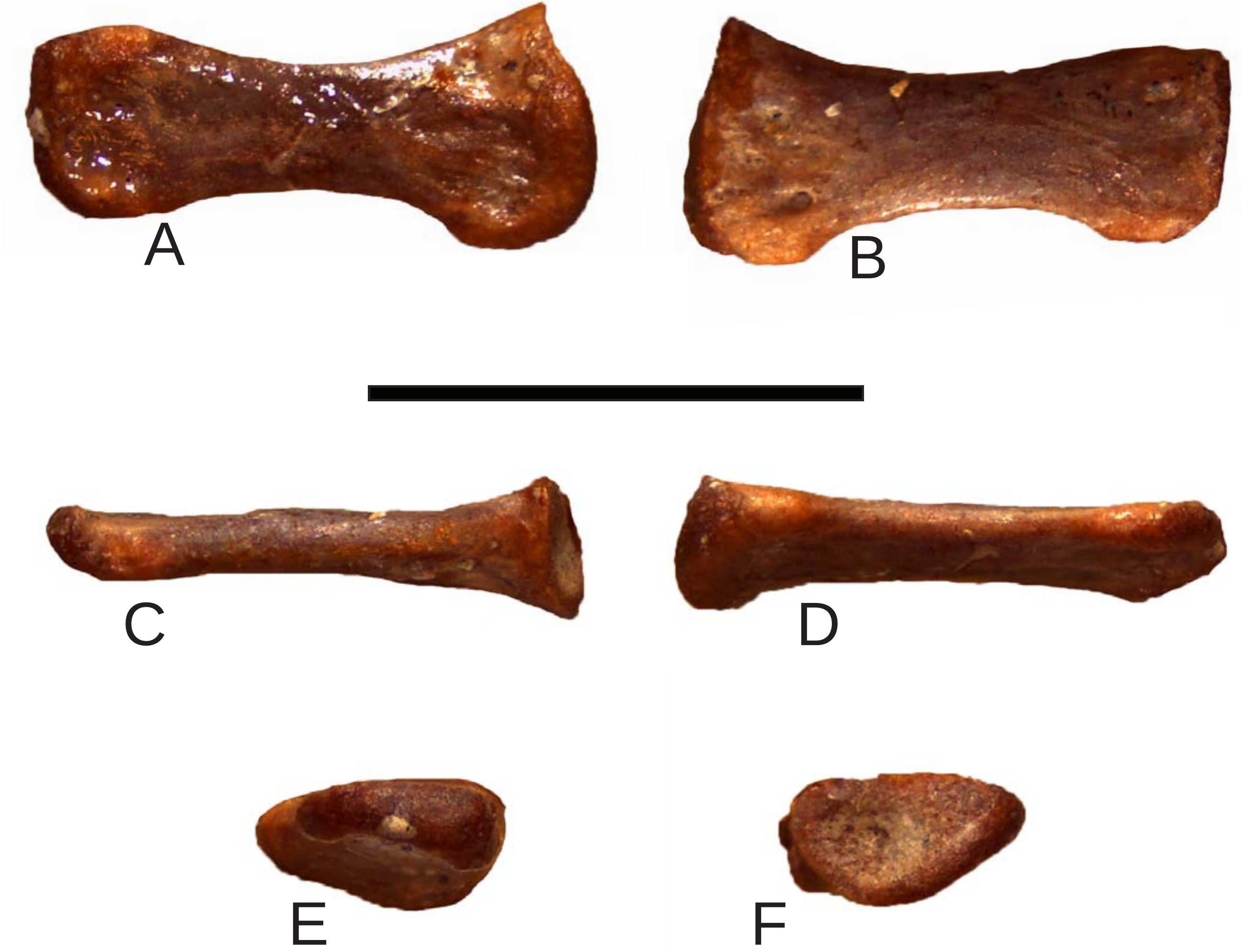

Figure S68. ***Trachemys haugrudi*****, holotype right proximal phalanx V (ETMNH–8549).**

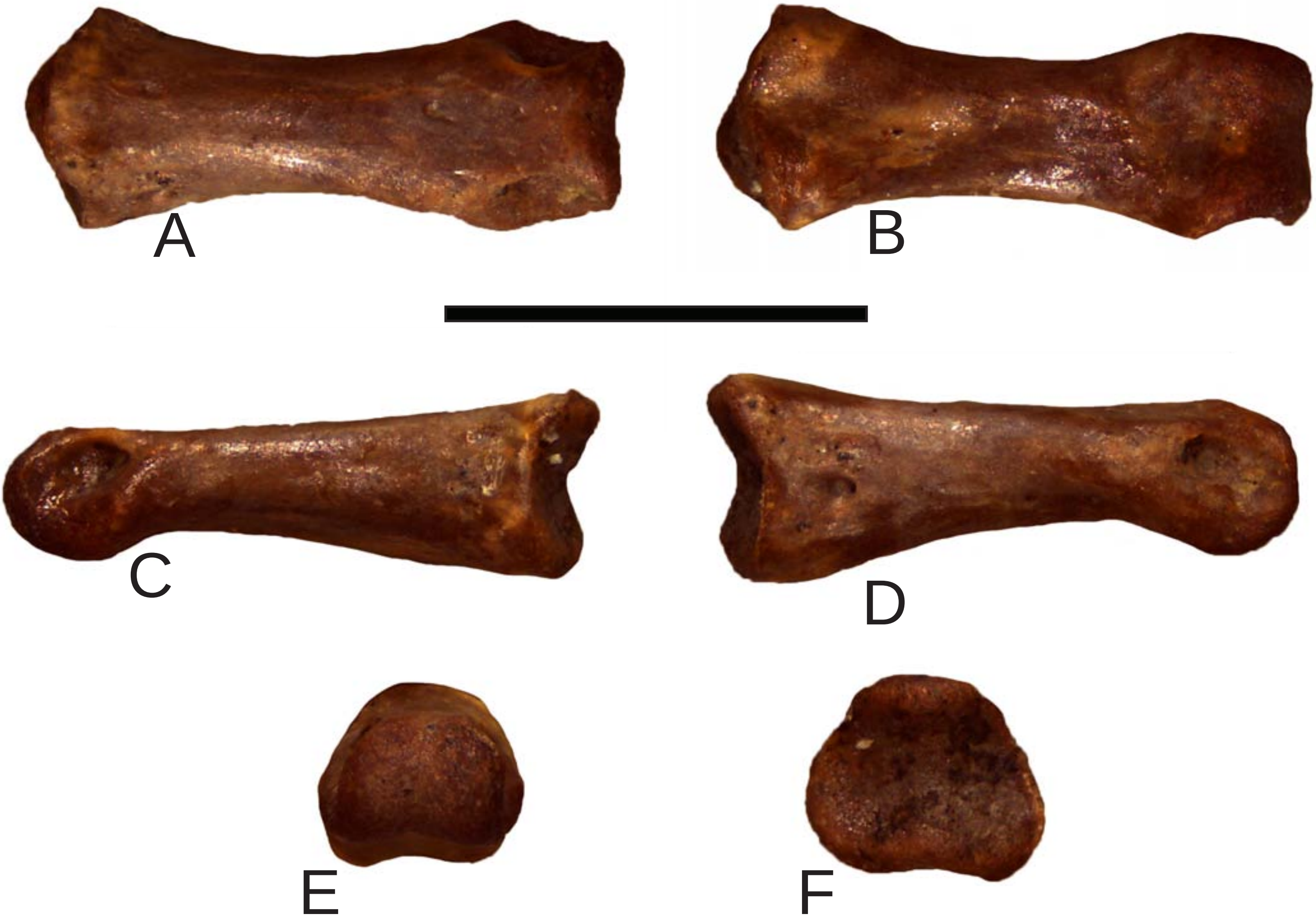

Figure S69. ***Trachemys haugrudi*, holotype right medial phalanx ?III (ETMNH–8549).**

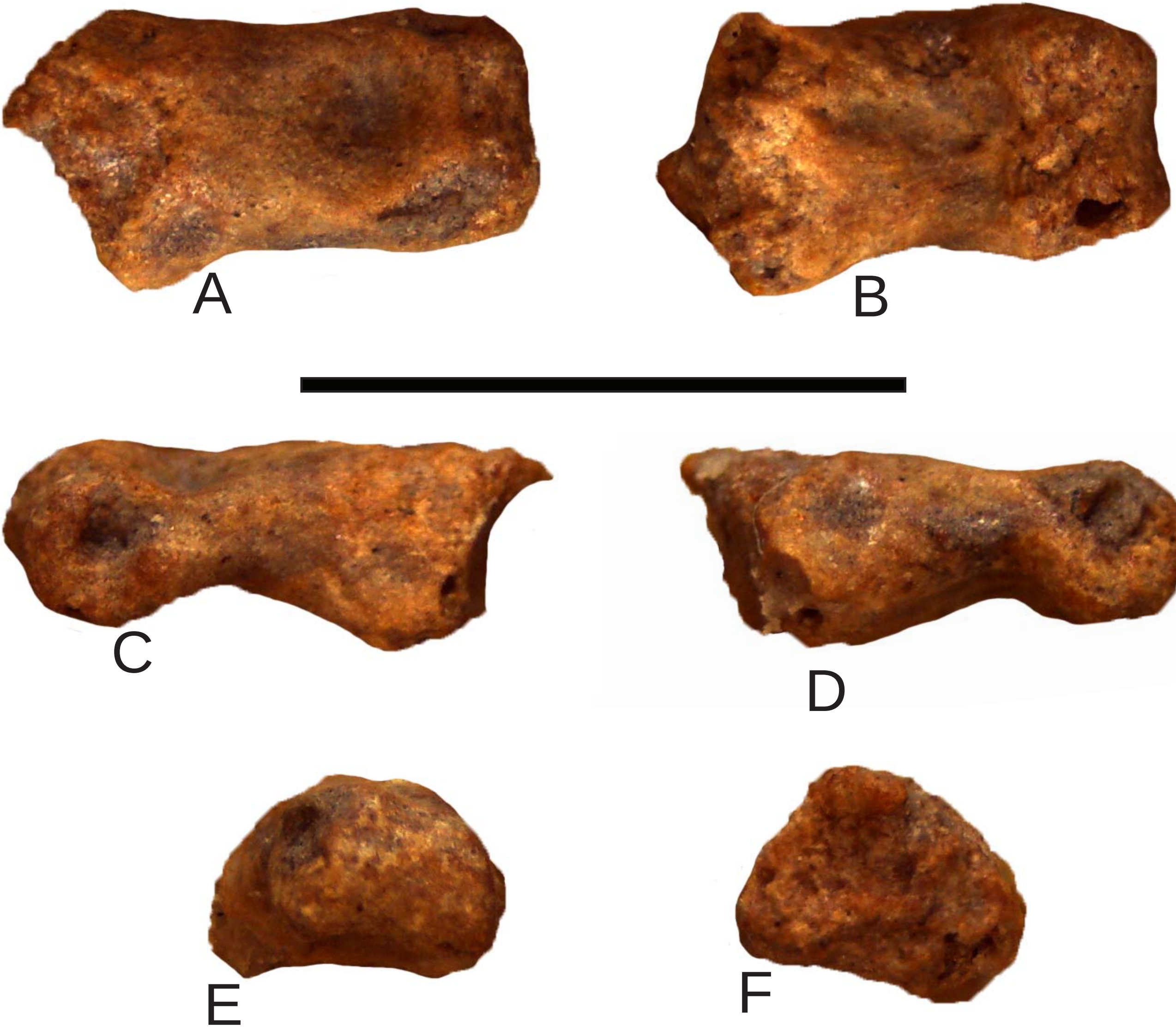

Figure S70. ***Trachemys haugrudi*****, holotype right distal phalanx ?II (ETMNH–8549).**

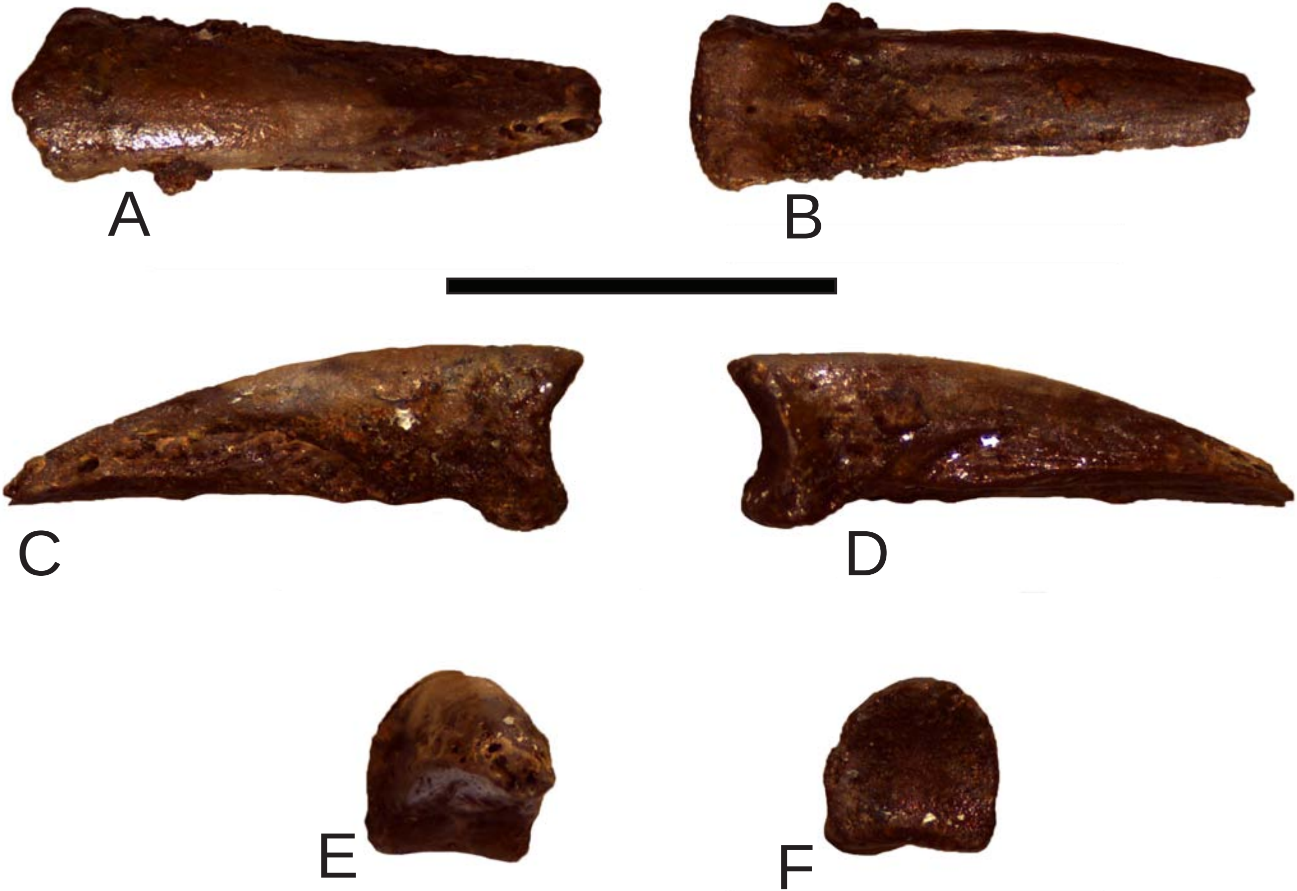

Figure S71. ***Trachemys haugrudi*, referred pedal ungual ?III (ETMNH–10547).**

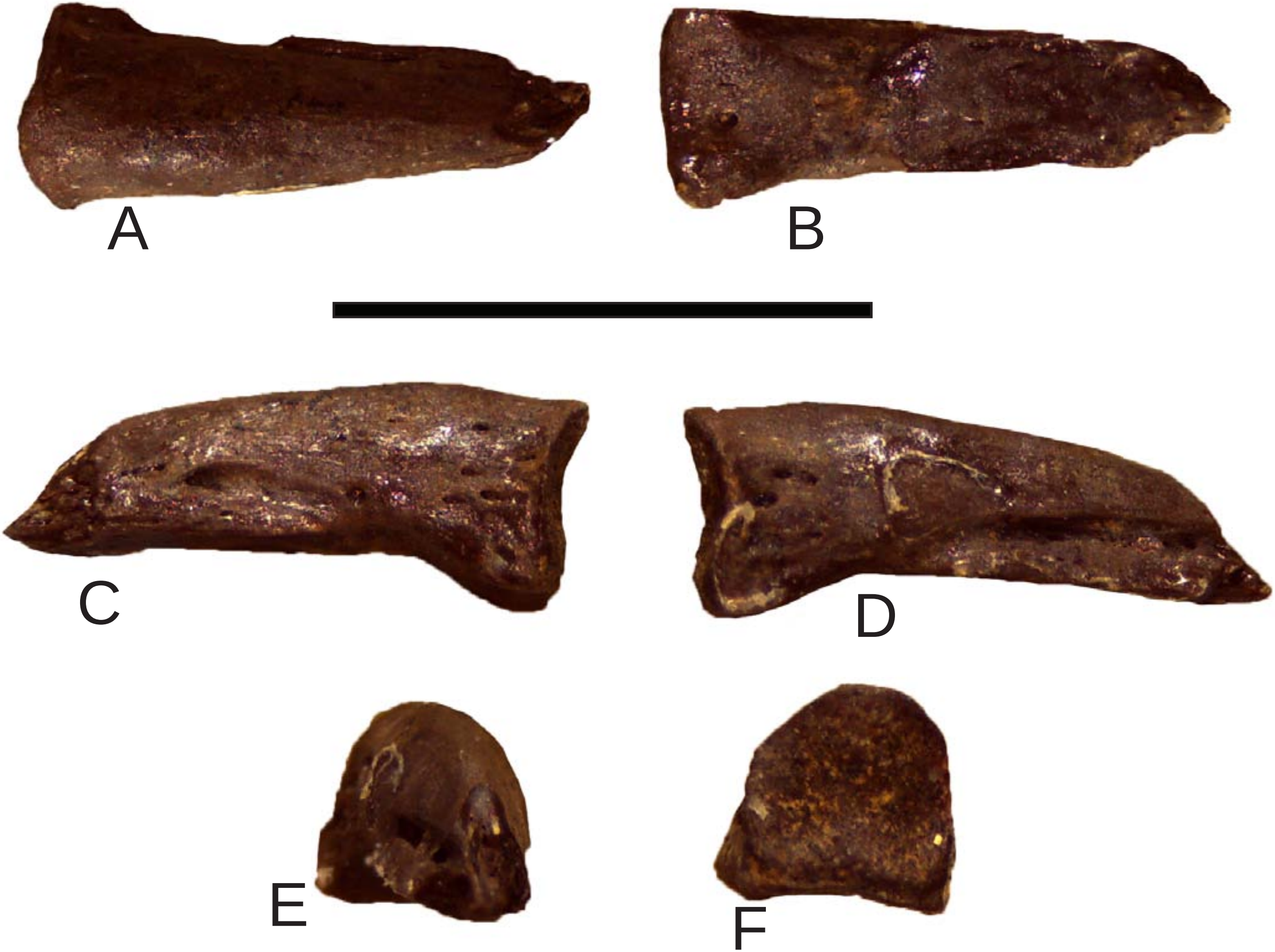

Figure S72. ***Trachemys haugrudi*, paratype pedal ungual ?IV (ETMNH–11642).**
