# Supplementary material for: A new slider turtle (Testudines: Emydidae: Deirochelyinae: Trachemys) from the late Hemphillian (late Miocene/early Pliocene) of eastern Tennessee and the evolution of the deirochelyines
Source: PeerJ. 2018 Feb 13;6:e4338. doi: 10.7717/peerj.4338 (PMC5815335; doi:10.7717/peerj.4338)
Supplement: Supplemental Information 8 [file peerj-06-4338-s008.pdf]

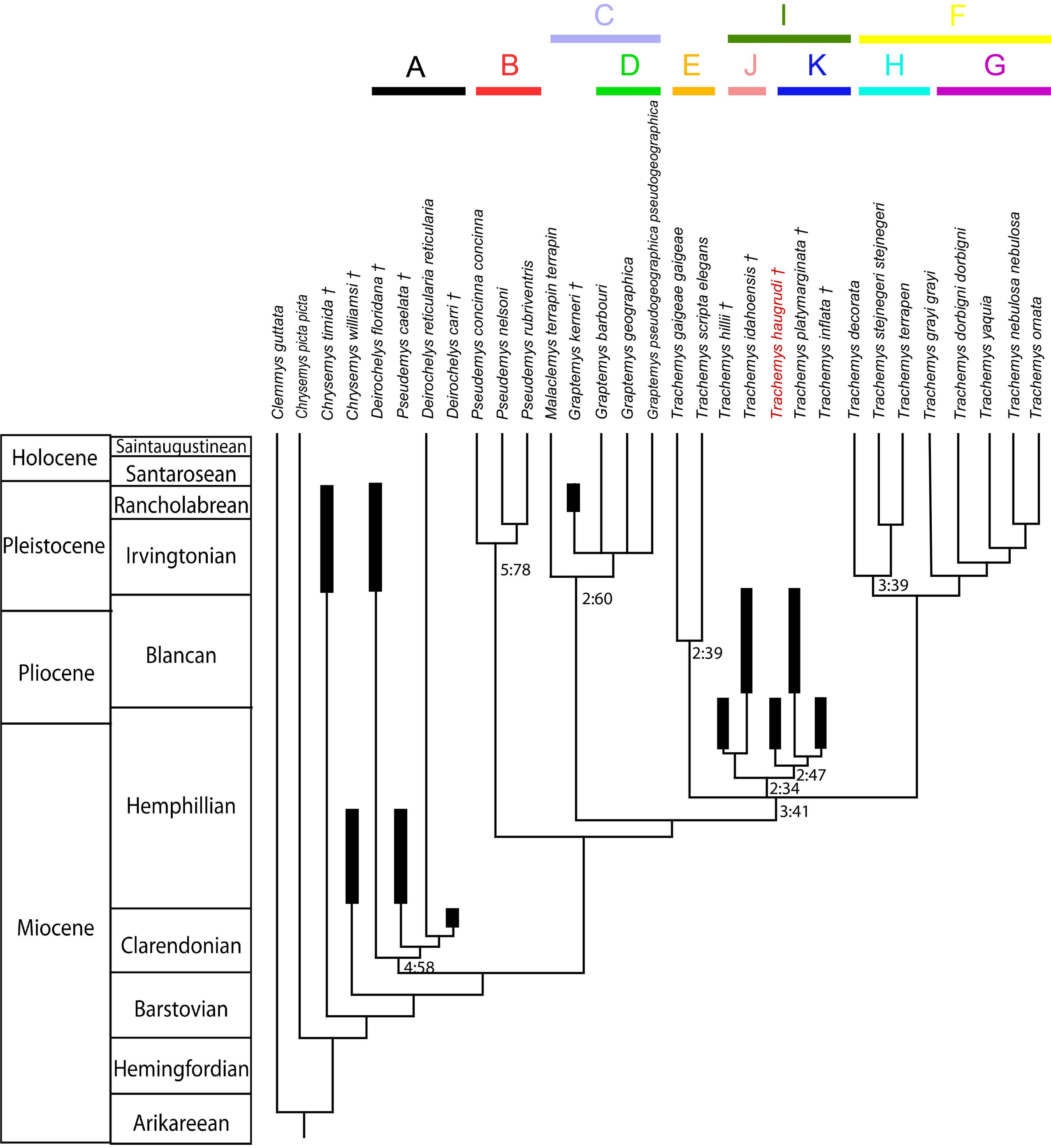

Figure S73. 50% majority rule consensus tree of the phylogenetic relationships of deirochelyine emydids supported by this study based on morphologic data.

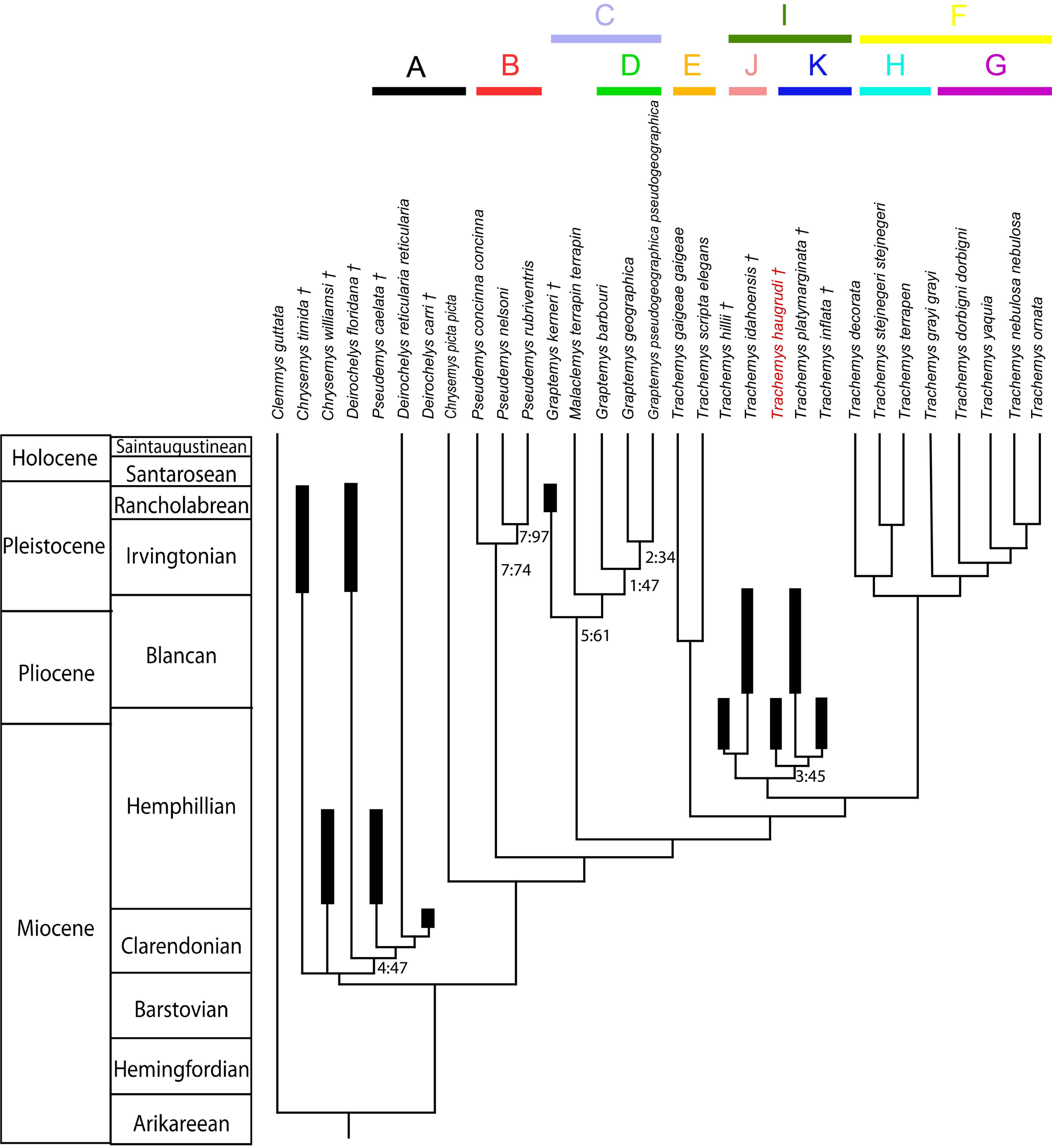

Figure S74. 50% majority rule consensus tree of the phylogenetic relationships of deirochelyine emydids, with constraints on modern genera, supported by this study based on morphologic data.
